# Supplementary material for: Nurture Early for Optimal Nutrition (NEON) participatory learning and action women’s groups to improve infant feeding and practices in South Asian infants: pilot randomised trial study protocol
Source: BMJ Open. 2023 Nov 29;13(11):e063885. doi: 10.1136/bmjopen-2022-063885 (PMC10689384; doi:10.1136/bmjopen-2022-063885)
Supplement: Supplementary data [file bmjopen-2022-063885supp001.pdf]

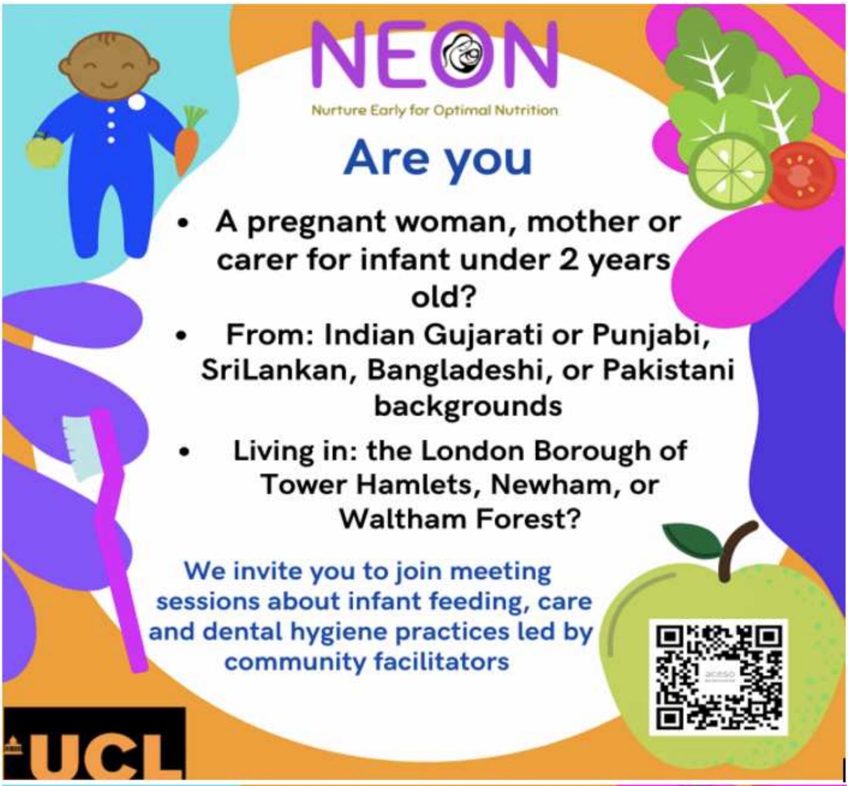
NEON
Nurture Early for Optimal Nutrition
Are you
• A pregnant woman, mother or carer for infant under 2 years old?
• From: Indian Gujarati or Punjabi, SriLankan, Bangladeshi, or Pakistani backgrounds
• Living in: the London Borough of Tower Hamlets, Newham, or Waltham Forest?
We invite you to join meeting sessions about infant feeding, care and dental hygiene practices led by community facilitators
UCL

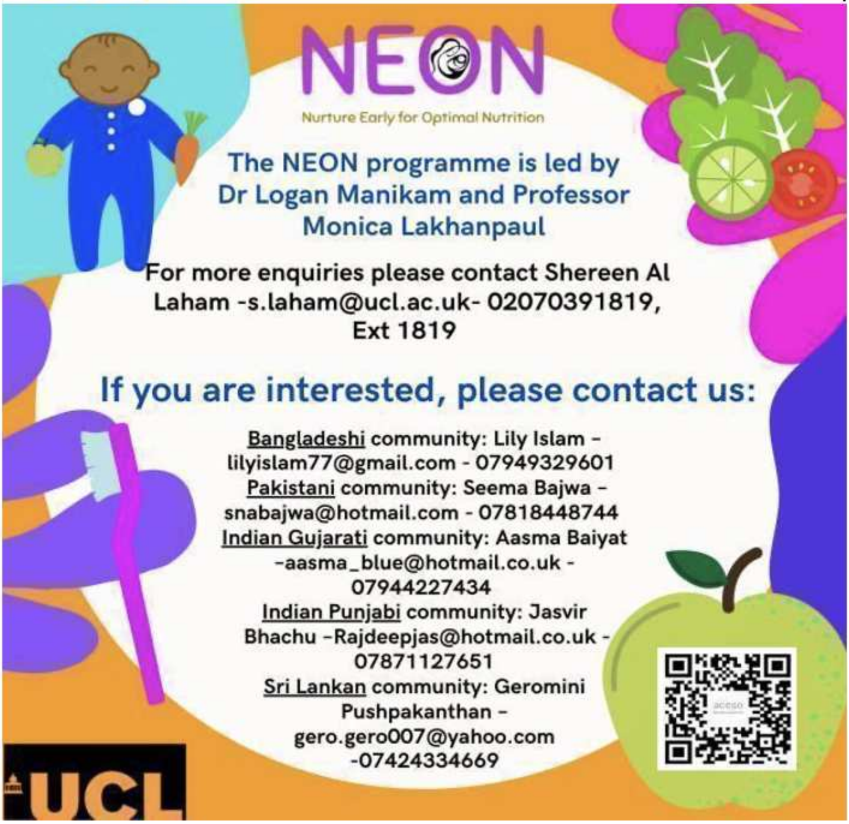
NEON
Nurture Early for Optimal Nutrition
The NEON programme is led by Dr Logan Manikam and Professor Monica Lakhanpaul
For more enquiries please contact Shereen Al Laham -s.laham@ucl.ac.uk- 02070391819, Ext 1819
If you are interested, please contact us:
Bangladeshi community: Lily Islam – lilyislam77@gmail.com - 07949329601
Pakistani community: Seema Bajwa – snabajwa@hotmail.com - 07818448744
Indian Gujarati community: Aasma Baiyat –aasma_blue@hotmail.co.uk - 07944227434
Indian Punjabi community: Jasvir Bhachu –Rajdeepjas@hotmail.co.uk - 07871127651
Sri Lankan community: Geromini Pushpakanthan – gero.gero007@yahoo.com -07424334669
UCL
