# Supplementary figures and images for: Nurture Early for Optimal Nutrition (NEON) participatory learning and action women’s groups to improve infant feeding and practices in South Asian infants: pilot randomised trial study protocol
Source: BMJ Open. 2023 Nov 29;13(11):e063885. doi: 10.1136/bmjopen-2022-063885 (PMC10689384; doi:10.1136/bmjopen-2022-063885)

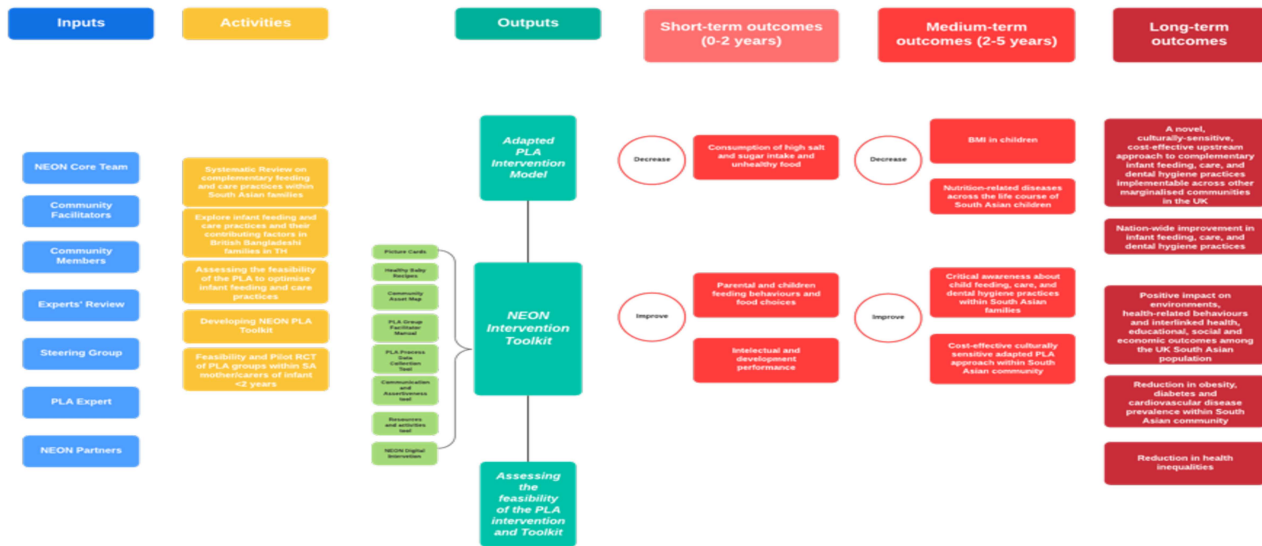

Supplement: Supplementary data [file bmjopen-2022-063885supp002.pdf]
