# Supplementary material for: Nurture Early for Optimal Nutrition (NEON) participatory learning and action women’s groups to improve infant feeding and practices in South Asian infants: pilot randomised trial study protocol
Source: BMJ Open. 2023 Nov 29;13(11):e063885. doi: 10.1136/bmjopen-2022-063885 (PMC10689384; doi:10.1136/bmjopen-2022-063885)

BMJ Publishing Group Limited (BMJ) disclaims all liability and responsibility arising from any reliance placed on this supplemental material which has been supplied by the author(s)

RESOURCES AND SERVICES SUPPORTING INFANT FEEDING, CARE AND DENTAL HYGIENE

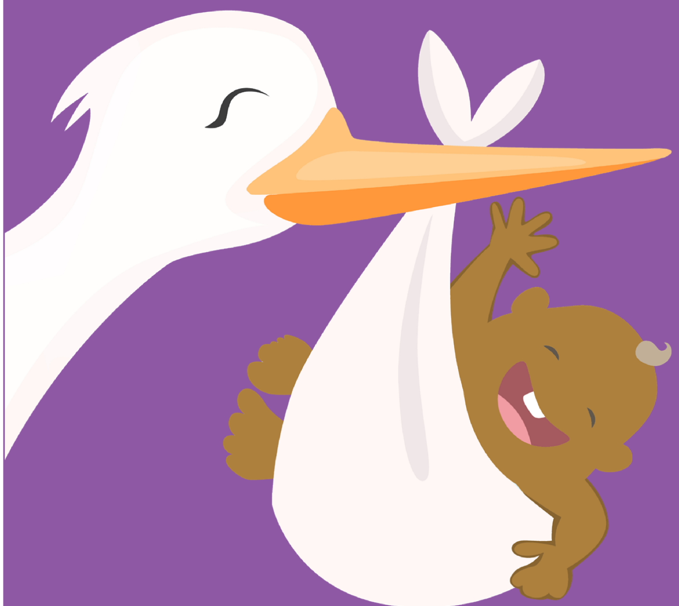

NEON
Nurture Early for Optimal Nutrition

Manikam L, *et al. BMJ Open* 2023; 13:e063885. doi: 10.1136/bmjopen-2022-063885

NHS AND COUNCILS ADVICE AND SUPPORT

1. NHS STAR4LIFE

https://www.nhs.uk/start4life

Website provides trusted NHS help and advice for parents and carers during pregnency, and birth and the early years.

2. NHS CHOICES

NHS Choices (www.nhs.uk) is the official website of the National Health Service in England and provides comprehensive health information service with thousands of articles, videos and tools. Key pages which give guidance on infant feeding and care include:

- **Your newborn:** *https://www.nhs.uk/conditions/pregnancy-and-baby/childrens-meal-ideas/?tab-name=your-newborn*
- **Babies and toddlers** *https://www.nhs.uk/conditions/pregnancy-and-baby/childrens-meal-ideas/?tabname=ba-bies-and-toddlers*

3.CHILDREN CENTRES AND LIBRARIES

Tower Hamlets has 12 children centres across the borough providing a range of services to infants, children and families to give them the support they need to be safe, healthy and happy, so that they are able to reach their full potential. This web page on the **Tower Hamlets Council** website gives locations and contact details of all the children's centres in the borough: *https://www.towerhamlets.gov.uk/lgnl/education_and_learning/childcare_and_early_years_educ/Children_centres/childrens_centres.aspx*

The borough of Newham offers support to its residents with children under the age of 5 through **Best Start in Life (BSiL)** Children's centres. These include play activities and childminder services, as well as supporting parents to improve their confidence, support their children with healthy eating and weaning. Libraries offer free courses and internet services for parents, and they offer devices and digital equipment to borrow.

Locations and details can be found on the Families Newham website: *https://families.newham.gov.uk/kb5/newham/directory/family.page?familychannel=3-2*

Waltham Forest offers to its families a similar service, with support for healthy development and access to financial support. They have implemented the 2 Year Old Partnership Pathway in which professionals empower and inform parents to assure the best start in life for their children. They offer advice on a wide range of issues including health, finance and cognitive/motor development. **Waltham Forest** offers support directed at healthy weight and eating through HARRY.

More information can be accessed via the following link: *https://www.walthamforest.gov.uk/content/2yo-partnership-pathway*

Further informal support that does not require formal registration can generally be accessed in public libraries

GUIDENCE DOCUMENTS, ONLINE RESOURCES AND APP'S

1. BABY FRIENDLY INITIATIVE

Published by the Department of Health and the Baby Friendly Initiative, the leaflets below provide mothers and health professionals with key information about breast feeding, introducing solid foods to babies, and evidence-based guidance around formula. The **Baby Friendly Initiative** is part of a global partnership between the World Health Organization (WHO) and UNICEF. The guidance below should be prioritised over other online resources (other than NHS Choices) as they the baby friendly initiative is less influenced by commercial interests, which can sometimes influence the advice offered to mothers and parents regarding infant feeding and care.

- **Leaflet on weaning and starting solid food:**
  https://www.unicef.org.uk/babyfriendly/baby-friendly-resources/leaflets-and-posters/weaning-starting-solid-food/

- **Leaflet on breastfeeding:**
  https://www.unicef.org.uk/babyfriendly/baby-friendly-resources/leaflets-and-posters/off-to-the-beststart/ -

- **Leaflet on infant formula:**
  https://www.unicef.org.uk/babyfriendly/baby-friendly-resources/leaflets-and-posters/simple-formula-guide-for-parents/

- **Leaflet on bottle feeding:**
  https://www.unicef.org.uk/babyfriendly/baby-friendly-resources/leaflets-and-posters/guide-to-bottle-feeding/

- **Baby friendly initiative resources in Bengali:**
  https://www.unicef.org.uk/babyfriendly/baby-friendly-resources/leaflets-and-posters/foreign-language-resources/bengali-resources/

- **Baby friendly initiative resources in Hindi:** बच्चे के अनुकूल पहल संसाधन
  https://www.unicef.org.uk/babyfriendly/baby-friendly-resources/foreign-language-resources/hindi-resources/

- **Baby friendly Initiative resources in Urdu:** ل ئ اس و ے ک)ت ش اده گ ن(ءام ن وش ن ئ ادت ب ا ی ک ے چ ب
  https://www.unicef.org.uk/babyfriendly/baby-friendly-resources/foreign-language-resources/urdu-resources/

Other Baby Friendly Initiative resources/leaflets:

https://www.unicef.org.uk/babyfriendly/baby-friendly-resources/leaflets-and-posters/

2. FIRST STEPS NUTRITION TRUST

https://www.firststepsnutrition.org

First steps nutrition can also be used as a trusted source of good information and guidance regarding nutrition and feeding in pregnancy, infants, and early years. **First Steps Nutrition Trust** is funded by research or charitable grants and donations, and does not take money from any commercial organisation or any organisation or individual linked to the sales of infant formula and other food or drink product or service which is associated with poor nutritional health of women and children.

First Steps Nutrition Trust offers some useful resources on meal choices and portion sizes, with helpful diagrams to illustrate portion size. Remember! Portion size is a maximum, not a minimum, never encourage a child to eat more when they indicate they have had enough.

- Eating well: the first year – guidance on introducing foods, food choices and meals for up to 7 months, 7-9 months, and 10-12 months: http://www.firststepsnutrition.org/newpages/Infants/first_year_of_life.html
- Eating well: Early years – guidance on food choices, meals, snacks and portion size http://www.firststepsnutrition.org/newpages/Early_Years/eating_well-early_years.html

3. BORN TO MOVE APP

Born to move is a free NHS app to help parents or carers with their newborn babies right up to pre-school, specifically relating to physical activity and play. It's packed with advice, tips and games that you can play with your child to support their development.

https://itunes.apple.com/gb/app/born-to-move/id976095022?mt=8Local%20initiatives/projects

https://play.google.com/store/apps/details?id=com.project.Born&hl=en_GB

4. EREDBOOK

ERedbook is an app based digital version of the paper Redbook. It provides solutions for parents that allows them to review their child's NHS records whilst providing health information and guidance from NHS and other sources. Additionally, it contains personalised information for new parents, including relevant videos and information from local sources & reminders and appointments updates. Parents can review the NHS child's health records on the app when they connect record to NHS. Also, The NEON participants will be able to review the NEON toolkit and receive correct information via the ERedbook app at any time and place.

https://www.eredbook.org.uk

https://apps.apple.com/gb/app/eredbook/id606443658

https://play.google.com/store/apps/details?id=com.sitekit.eRedBook&hl=en_GB

OTHER SOLUTION IDEAS TO SUPPORT PLAY AND ACTIVITY:

1. Develop activity and play schedules for you and your infant with guidance from our local children centre or health visitor and make time for these activities.

2. Download the 'born to move' app for access to information and guidance on infant activity and play.

3. Designate a play area (even if this needs to be set-up and put away at times of day) for your infant in the home.

4. Arrange play and physical activity sessions together as a group, or with other parents, extended family, carers and infants – this could help with support and confidence in exercising and conducting physical activity in public spaces.

OTHER LOCAL INITIATIVES, CHARITIES AND PROJECTS IN EAST LONDON

1. BREASTFEEDING NETWORK

Find your nearest breast feeding drop-in-group in Tower Hamlets
https://www.breastfeedingnetwork.org.uk

There are groups run at Shadwell Children's Centre, Chrisp Children's Centre, Overland Children's Centre, Collingwood Children's Centre, Wapping Children's Centre and many more. If you need information or support with breastfeeding in the evenings or at weekends please call:

National Breastfeeding Helpline: *0300 1000 212*

BfN Support in Bengali/Sylheti: *0300 456 2421*

Open from *9.30am - 9.30pm 365 days a year*

Newham: https://www.nct.org.uk/local-activities-meet-ups/region-london/newham/breastfeeding-support

Tower Hamlets: https://www.breastfeedingnetwork.org.uk/tower-hamlets/

Waltham Forest offers a team of infant feeding practitioners delivering 1:1 sessions, a helpline and at home visitors to support the breastfeeding family through HENRY.

For information:
Email: wfsupport@henry.org.uk
Telephone: 020 8496 5223

2. DENTAL SERVICES AND RESOURCES

Dentists across Tower Hamlets, Waltham Forest, and Newham offer free dental check-ups, which you can attend as soon as your child's teeth first appear.

To find your nearest dental practice in Tower Hamlets, please call 020 7364 5000.

HENRY has put together some tips for healthy teeth in young children and a list of Waltham Forest dentists currently accepting new patients from birth: https://www.henry.org.uk/sites/www.henry.org.uk/files/inline-files/Healthy%20Teeth%20Leaflet%20%28April%29_0.pdf

The Kent Community Health NHS Foundation Trust has compiled some online oral health promotion resources for parents here: https://www.kentcht.nhs.uk/service/dental-services/oral-health-promotion-resources/

The Kent Community Health NHS Foundation Trust has compiled some online oral health promotion resources for parents here: https://www.kentcht.nhs.uk/service/dental-services/oral-health-promotion-resources/

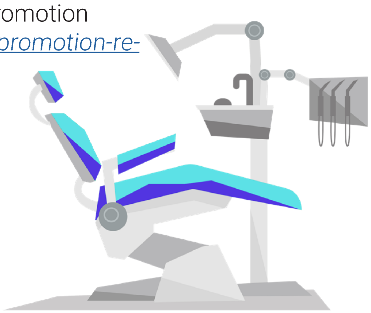

Here are examples of dental services by borough (to find out more, please see the community asset map):

Tower Hamlets

- Fresh Springs Dental Practice
  T: 020 3216 0077
  E: reception@freshspringsdentalpractice.co.uk

- Together Dental Whitechapel
  T: 020 7247 4600
  E: whitechapelreception@together.dental

Waltham Forest

- Abbey Dental Practice
  T: 020 8521 2816
  E: info@abbeydentalwalthamstow.co.uk

- Inspire Dental Walthamstow
  T: 020 8521 6656
  E: info@inspiredentalwalthamstow.co.uk

3. MATERNITY MATES

http://www.whfs.org.uk/index.php/what-we-do/maternity-mates

Maternity Mates™ is a project run by the charity Women's Health and Family Services which recruits, trains and match-ups volunteer Maternity Mates with pregnant women in need of extra support in Tower Hamlets. A Maternity Mate is a female volunteer trained by WHFS to provide practical and emotional support to women during pregnancy, childbirth and the early weeks of motherhood. Support can start any time from the 5th month of pregnancy, through to birth and up to either 6 or 12 weeks after the baby is born (6 weeks in Tower Hamlets and 12 weeks in Newham). **Maternity Mates** are recruited from the communities they serve, and where possible, will speak the same language as the mother-to-be.

For further information, please contact:

Jasmin Begum - Programme Manager
M: *07960 327 760*
T: *020 7377 9640*
E: *jasmin.begum@whfs.org.uk*

For information about the project in Tower Hamlets and Waltham Forest, please contact:

Rubi Rodriguez - Project Co-ordinator Tower Hamlets and Waltham Forest
M: 07496 764 881
E: rubi.rodriguez@whfs.org.uk

For information about the project in Newham, please contact:

Irantzu Perez Arribas - Project Co-ordinator Newham
M: 07436 139 235
E: irantzu.perez@whfs.org.uk

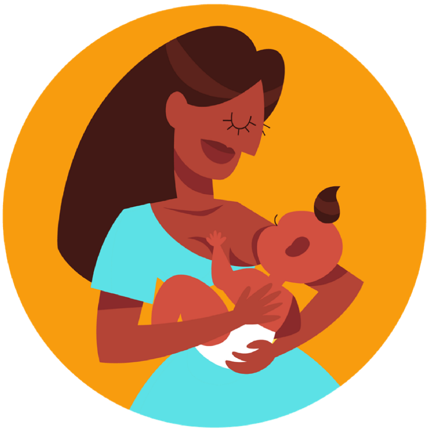

4. BOUNTY

https://www.bounty.com/about-bounty/bounty-packs/newborn-pack

Bounty provides mothers and pregnant women with free subscription packs including advice from Public Health England, as well as a variety of sample sized baby products (nappies, baby formula etc.) and coupons.

Every mother with a Newborn will receive a Newborn Pack at the hospital after delivery, and more freebies and advice are available through the Bounty app.

5. EMMA'S DIARY

https://www.emmasdiary.co.uk
Contact: 01628 535 483 Mon-Fri 9am to 4:30pm

Emma's Diary is a free to access resource created by a board of GPs and midwives that provide information ranging from trying to get pregnant from wellbeing information during and post-pregnancy. There's a free to download mobile app to allow you to access information more easily, tailored towards recent mothers and baby related advice and offers.

6. THE TOYHOUSE CENTRE

http://www.toyhouse.org.uk/

Phone: 0020 7987 7399
E-mail: info@toyhouse.org.uk

Toy House is a local charity in Tower Hamlets which runs projects and services aimed at support play and wellbeing of infants, children and families.

Toy Libraries
http://www.toyhouse.org.uk/toy-libraries/
The charity has its own toy library, and has information and links to over 19 toy libraries and stay and play sessions run across Tower Hamlets.

7.MEND - MIND, EXERCISE, NUTRITION...DO IT!

https://www.mytimeactive.co.uk/mend

MEND is a programme which empowers mums, children and adults to become fitter, healthier and happier and to reach or maintain a healthier weight. It's free to attend and run by local teams in local communities, after school or at weekends. MEND have a range of programmes for different ages run in different boroughs across London.

MEND Mums is designed for post-natal women with a baby up to 2 years and a BMI of 25 or above, **MEND** Mums is a fun and interactive weight management programme incorporating energy boosting workouts and great nutrition tips to help establish healthy habits for life.

For more information about any of the MEND programmes in Tower Hamlets call:
020 8323 1725

8. MIND

MIND is a mental health charity with a specific branch in Newham and Tower Hamlets that offers free mental health services talking therapies, COVID19 Support and Advocacy services

https://www.mithn.org.uk
Phone: 020 7510 1081
Email: info@mithn.org.uk

9. REBECCA CHEETHAM NURSERY & CHILDREN'S CENTRE

Website: *https://www.rebeccacheetham.newham.sch.uk*
Phone:
Email:

The Rebecca Cheetham Children's Centre offers free child-minding services as well as advice and support for families with young children. However they have temporarily stopped face to face sessions in light of the COVID pandemic. Limited online services are still running for examples: a Family Support Team that can refer you to foodbanks.

OTHER IDEAS AND SUGGESTIONS

- **Breakfast clubs** – these are available in most schools to give children a healthy start to the day. Speak to your local school. Due to COVID19 Restrictions, these breakfast clubs have moved to.
- Despite this, virtual coffee mornings are ongoing

- **Social support** – if you feel you need more support from other mothers, parents or people local to you, you can attend local coffee mornings, toddler groups and parent forums. E.g. One stop shop in Newham and Tower Hamlets. Father examples would be the "idea Store" which is based in Tower Hamlets and offers a range of free activities and educational services for children and families alike. *http://www.healthwatchnewham.co.uk/sites/default/files/newham_domestic_abuse_one-stop-shop.pdf*

- **Triple P – Positive Parenting Programme** – is an evidence based programme aimed at supporting parents develop relationships with their children.

  - Newham offers a Parenting Befriending service and bookable workshops accessible via the following website: *https://families.newham.gov.uk/kb5/newham/directory/family.page?family-channel=1-1*

  - Tower Hamlets offers a similar service: *https://www.towerhamlets.gov.uk/lgnl/educa tion_and_learning/parental_support/parenting_programmes.aspx*
    parenting@towerhamlets.gov.uk.

  - Waltham Forest offers a series of 6 sessions for parents with children aged 3 and over. *https://www.walthamforest.gov.uk/content/parenting-support*

    Call: *020 8496 2442*
    Email: *earlyhelpparenting@walthamforest.gov.uk*

- **Tunmarsh Centre**– support for parents with children with special needs
  The New Tunmarsh Centre based in Newham provides free education for children with ASD / DHD and support for their families. Parents are regularly invited for coffee mornings with speakers.

  E-mail: Faz.Mac@gmail.com

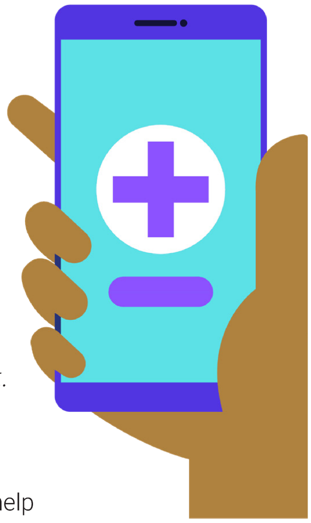

- **John Smith Children's Centre**

  Telephone: 0207 364 0537
  Contact: Deborah Wooding
  Email: deborah.wooding@towerhamlets.gov.uk
  Based in Tower Hamlets, The John Smith Children's Centre that offer childcare support.

- **The Trinity Centre**
  Website: www.thetrinitycentre.org/home/
  The Trinity Centre offers a day care nursery as well as independent groups and faith groups to help support parents form minority and marginalised communities

- **Around Poplar Children's Centre**

  Contact: Brenda Pascal
  T: 020 7364 0540
  E: Brenda.pascal@towerhamlets.gov.uk

  The Around Poplar Children's Centre offers childcare, family, health services, and early education support for families with children under 5 free of charge.

  For a list of similar children's centres in Tower Hamlets, see here:
  https://www.localoffertowerhamlets.co.uk/organisations?utf8=%E2%9C%93&search_organisation%5Bterm%5D=&search_organisation%5Blocation%5D=&search_organisation%5Blatitude%5D=&search_organisation%5Blongitude%5D=&search_organisation%5Bcategory_ids%5D%5B%5D=3308

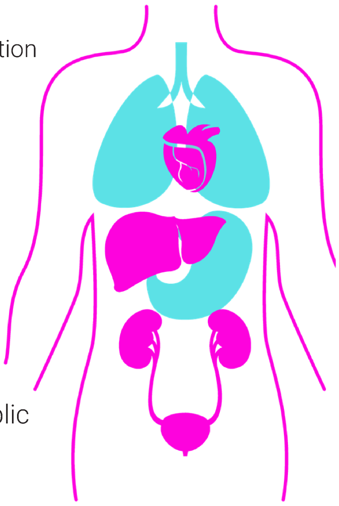

- **Idea Store**

  Website: www.ideastore.co.uk
  Branches of the Idea Store in Tower Hamlets offer library services and access to public computers for families and individuals free of charge.

- **Outreach centre** Outreach centres are open till 6 pm where you can drop your children there, and it is free for people who are entitled for public funds. They offer: Child minding, after school club, breakfast club for parents and children <3 years. Key examples would be Community Centres and Children's Centres.

EXAMPLES:

Newham Council: *https://www.newham.gov.uk/homepage/115/community-centres*
Tower Hamlets: Community and day centres (towerhamlets.gov.uk)

Plaistow Children's Centre
Website: *https://plaistow.newham.sch.uk/Plaistow-Children-s-Centre/*

The Plaistow Children's Centre is based in Newham and offers care and support for young children ranging from 0-5 years for 49 weeks within the year led by staff and volunteers. Other services range from nutrition webinars to overall family support and advice. The Children's Centre is still open during the pandemic and offers slightly modified services as a result.

OTHER SUPPORT

1. Request interpretation support for GP visits from Tower Hamlets GP care group.
2. Advocacy & Interpreting Service in Newham (https://www.gpcaregroup.org/section/455/Services/page/eb745e07-85ab-4cee-bc8f-c880a-d400a95/Advocacy-Interpreting)
3. Provide feedback on services or lodge a complaint via Health Watch Tower Hamlets. More information can be accessed here: https://www.healthwatchtowerhamlets.co.uk/

FOOD BANKS:

Suggested Food Bank locations: https://www.google.com/maps/d/edit?mid=1ZyqIE2-HgdQ-

NEON Community Asset Map: https://www.google.com/maps/d/edit?mid=1ZyqIE2-Hg-dQ-Hzow7KwhAiYsdtQnP_Zq&usp=sharing

- **Newham Food Alliance**

  Website: https://www.newham.gov.uk/newhamfoodalliance

  The Newham Food Alliance is a food bank based in Newham that can offer access to food for those who are struggling financially. There is also delivery support they can provide to those self-isolating due to COVID and providing food remotely during the pandemic regardless.

- **Church Food Banks:**

  Website: www.churchesfoodbank.org.uk/

  Food Banks located in the Borough of Newham. To access these you need to be referred from another agency such as a school, advice centres or your GP.

The 2 main sites are:

Woodgrange Baptist Church

345 Romford Road,Forest Gate,E7 8AA

0208 555 9880

St Paul's Church

65 Maryland Road,Stratford,E15 1JL

0208 534 1164

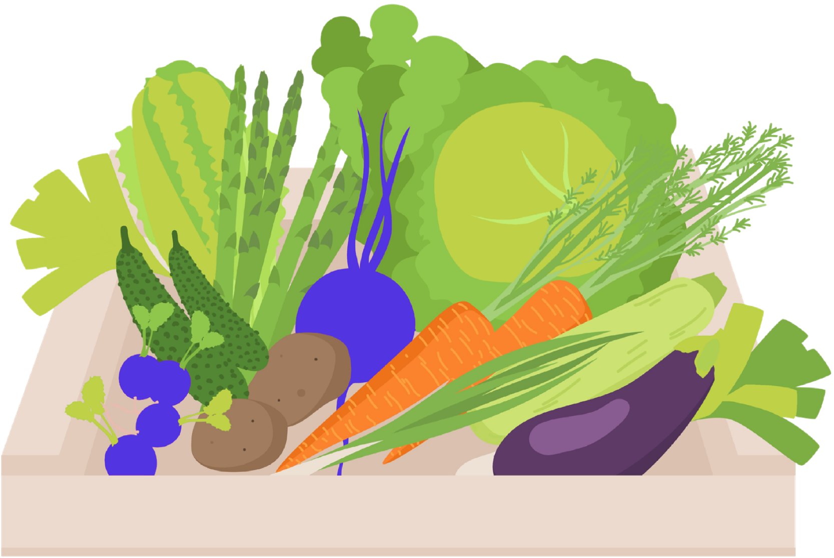

- **The Trussell Trust**

  Website: https://www.trusselltrust.org

  The Trussell Trust is a charity that provides nationwide network of food banks as well as emergency food for those in need. They also run regular campaigns to further increase support for people who need access to regular foods but cannot afford to do so. The website is useful as it flag foodbanks that are local to you.

- **First Love Foundation Foodbank**

  Website: www.firstlovefoundation.org.uk/

  The First Love Foundation coordinates the Tower Hamlets Foodbank. It provides daily necessities to families in need and alongside this, operates as a crisis service.

- **Eat or Heat - Waltham Forest Food Bank**

  Website: http://eatorheat.org

  Phone Number: 0800 772 0212

  Email: referrals@eatorheat.org

  A charity based in East London that supports local families. On the website, different professionals who can refer you to this service are listed.

- **Rukhsana Khan Foundation**

  Website: https://www.rukhsanakhanfoundation.org

  Phone Number: 07939 232 123

  Email: rukhsanakhanfoundation@outlook.com

  Rukshana Khan Foundation is a local charity that supports families in East London. They run food parcels and deliveries for those on benefits and those referred from Citizens Advice, NHS, Local Doctor's Surgery, Job Centre, Baby Food Bank, or the Council. They are also supporting those classed as clinically vulnerable. You can register for support on their website.

  They run a foodbank on Saturdays 11am-12pm at the William Morris Community Centre.

- **Waltham Forest Liberal Democrats**

  Website: https://www.walthamforestlibdems.org/local_support_for_families_in_need

  The Waltham Forest Liberal Democrats have collated a set of resources highlighting local foodbanks with Waltham Forest that local families can access

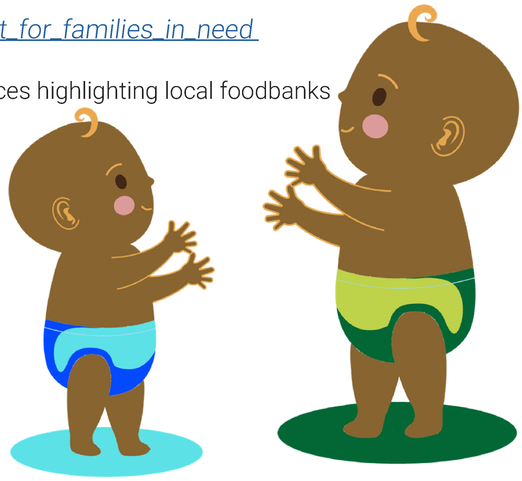

- **Eden Girls' School, Waltham Forest**

  A local food bank based at Eden Girls' School that provides food to local people from 1-3pm every Friday

  Contact Details:

  Eden Girls' School, Waltham Forest

  Silver Birch and Landmark Houses,

  Blackhorse Lane, Walthamstow

  London, E17 5SD

  Tel: 0208 523 1810

  Email: info@egwf.staracademies.org

- **Community Food Projects in Tower Hamlets**

  https://www.wen.org.uk/2020/03/30/https-www-wen-org-uk-2020-03-30-information-on-local-response-to-covid-19/

- **Healthwatch groups available in different boroughs:**

  Newham http://www.healthwatchnewham.co.uk/

  Tower Hamlets https://www.healthwatchtowerhamlets.co.uk/

  Redbridge http://www.healthwatchredbridge.co.uk/

  Waltham Forest https://www.healthwatchwalthamforest.co.uk/

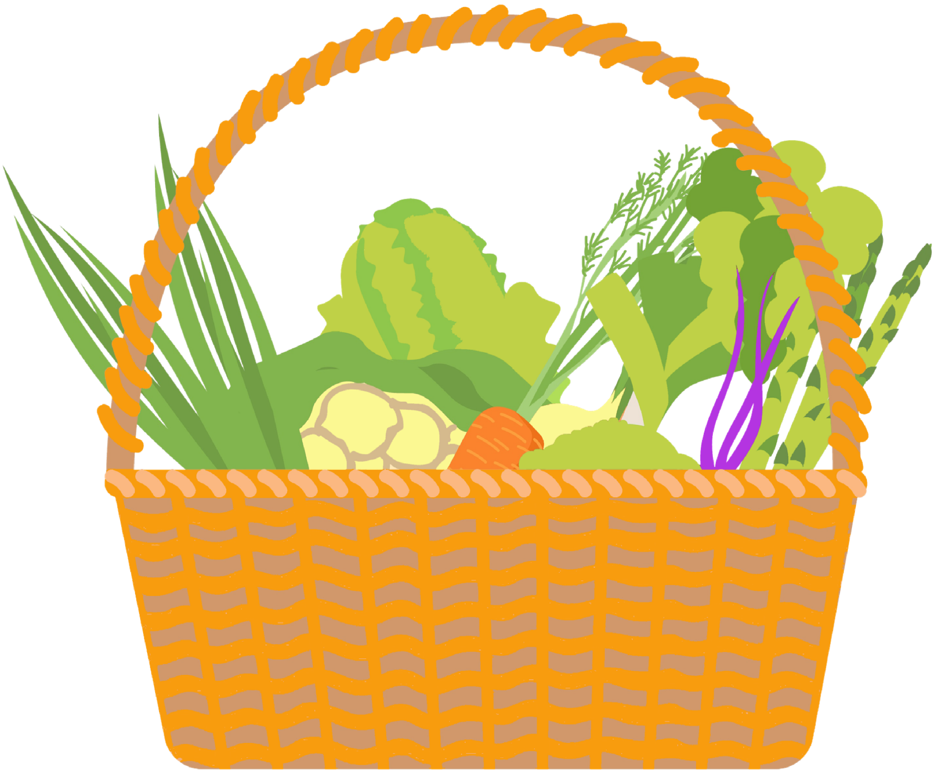

Supplement: Supplementary data [file bmjopen-2022-063885supp003.pdf]
