# Supplementary material for: Nurture Early for Optimal Nutrition (NEON) participatory learning and action women’s groups to improve infant feeding and practices in South Asian infants: pilot randomised trial study protocol
Source: BMJ Open. 2023 Nov 29;13(11):e063885. doi: 10.1136/bmjopen-2022-063885 (PMC10689384; doi:10.1136/bmjopen-2022-063885)
Supplement: Supplementary data [file bmjopen-2022-063885supp004.pdf]

Participants Feedback Questionnaire

1. What were your reasons for wanting to attend the group meetings?

2. What made you come back after attending a meeting? /or, what was your reason for not coming back to later meetings?

i. *What made you keep coming back to the meetings? /Or, what could we have done differently to motivate you to come back to the next meeting?*

3. Did the group manage to meet your expectations?

i. *how did it meet your expectations?*
ii. *If it did not, why was this the case?*

4. What could be improved if this project was run again?

i. *How could we improve the content (meeting exercises)*
ii. *How could we improve the tools (picture cards / infant feeding resources)*
iii. *How could we improve the tailoring to your needs as British Bangladeshi mother/pregnant women/or grandmother*
iv. Would it be beneficial to have picture cards and other project materials written in Bengali (with the picture on the front and Bengali text on the back)
v. Is there any additional information or resources you would have liked to have been offered in the meetings?

5. How involved did you feel in the learning process of the group?

i. Did you feel you worked together as a group during the meetings? How so?
ii. Were there any barriers you experienced to participating?
iii. Were you given opportunities to share experiences? Please give examples.

6. Are you in contact with any of the other participants outside of the group meetings?

i. Have you discussed any issues raised in the group meetings?
ii. Have you supported each other on any issues raised in the group meeting?

9. Have you found new people, groups or sources of information as a result of attending the group meetings? Please give examples.

10. Did you share any information from the group meetings with any other members of your family or community outside of the group?

11. If we were to implement a full project using this approach, the next stage would involve more group meetings (possibly up to 8 additional meetings) where by the group would support each other to implement some of the solutions you identified and then after implementing

solutions, evaluate their success. Do you think this is something you or Bangladeshi carers would be willing to undertake or partake in?

i. *Can you think of any barriers to using this approach we need to consider at this stage?*
ii. *How could additional meetings best support you to implement the solutions you identified, and introduce the recommended infant feeding and care practices? What should they focus on?*
iii. *What additional meetings would be acceptable to you and feasible to attend?*

12. Is there anything else you would like to share about your experience on the project?

Facilitator Report Form

**PLA GROUP FACILITATOR REPORT - PLA group meeting xx**					
Group location/name (XX children's centre group)		Meeting number		Meeting date	
Facilitator name					
Number of picture cards discussed					
Did you manage to go through all questions on the picture cards?<br>● *What does the picture show?*<br>● *Clarify which practice is being discussed*<br>● *Ask and explain how this affects infant growth and development*<br>● *Use info on the back of the card to ask questions about the practice and probe*<br>● *Ask if this occurs in the community? Is it common?*<br>Were there any barriers to a full discussion on the above questions?					
Were there any picture cards not discussed at all?					

Which exercises were completed during your meeting?	
What was the outcome of each exercise?	
How long did the meeting run for?	
Did anything go well/did anything not go well?	
GROUP FACILITATOR TO COMPLETE AFTER EACH MEETING CONDUCTED.	

**PLA GROUP FACILITATOR REPORT – PLA group meeting 3**					
Group location/name (XX children's centre group)		Meeting number		Me etin g dat e	
Facilitator name					
Number and type of picture cards discussed					
Did you manage to go through all the probing questions on the picture cards?<br>● *What does the picture show?*<br>● *Clarify what barrier is being discussed*<br>● *Ask and explain how this barrier may affect or influence behaviour relating to infant feeding and care*					

• *Ask which infant feeding and care practice the barrier may prevent parents from practicing?*<br><br>• *Ask if the barrier exists in the community*<br>Were there any barriers to a full discussion on the above questions?	
Were there any picture cards not discussed at all?	
Which exercises were completed during your meeting?	
What was the outcome of each exercise?<br>Which barrier cards were identified for prioritised practice 1?<br><br>Which barrier cards were identified for prioritised practice 2?<br><br>Which barrier cards were identified for prioritised practice 3?	
How long did the meeting run for?	
Did anything go well/did anything not go well?	
GROUP FACILITATOR TO COMPLETE AFTER EACH MEETING CONDUCTED.	

**PLA GROUP FACILITATOR REPORT – PLA group meeting 4**					
Group location/name (XX children's centre group)		Meeting number		Me etin g dat e	
Facilitator name					
Number and type of picture cards discussed					
Did you manage to go through all the probing questions on the picture cards?<br>● *What does the picture show?*<br>● *Ask participants to identify what solution they are discussing and what non-recommended infant feeding and care practice the solution helps to reduce or manage.*<br>● *Ask participants to suggest ideas on what activities the group (or they, as individuals) could undertake to achieve this solution. Use the information on the back of the card to suggest ideas on activities which could be undertaken. Ask if the barrier exists in the community*<br>● Were there any barriers to a full discussion on the above questions?					
Were there any picture cards not discussed at all?					
Which exercises were completed during your meeting?					

What was the outcome of each exercise?<br><br>Which barrier solution cards were identified for prioritised practice 1<br><br>Which management solution cards were identified for prioritised practice 1<br><br>Which barrier solution cards were identified for prioritised practice 2<br><br>Which management solution cards were identified for prioritised practice 2<br><br>Which barrier solution cards were identified for prioritised practice 2<br><br>Which management solution cards were identified for prioritised practice 2	
How long did the meeting run for?	
Did anything go well/did anything not go well?	
GROUP FACILITATOR TO COMPLETE AFTER EACH MEETING CONDUCTED.	

Sustainability assessment

1. PARTICIPATION

**Criteria for assessment**	***Are many people participating? Are they active? Is anyone excluded from participation?***
**Score / Average score assigned**	
**Identified successes**	
**Identified concerns / areas for improvement**	
**Actions agreed**	
**Additional notes**	

2. LEADERSHIP

**Criteria for assessment**	***Do groups have leaders, are they helping or hindering the group? Do they have the capacity to lead groups well?***
**Score / Average score assigned**	
**Identified successes**	
**Identified concerns / areas for improvement**	
**Actions agreed**	
**Additional notes**	

WOMEN AND CHILDREN FIRST (UK)

3. ORGANISATIONAL STRUCTURE

**Criteria for assessment**	***Are the organisational structures, including the committee and taskforces, set up in the groups? If so, what is their purpose and how are they functioning?***
**Score / Average score assigned**	
**Identified successes**	
**Identified concerns / areas for improvement**	
**Actions agreed**	
**Additional notes**	

4. NEEDS / PROBLEM ASSESSMENT

**Criteria for assessment**	***Are the problems identified in the groups important and relevant to their respective community?***
**Score / Average score assigned**	
**Identified successes**	
**Identified concerns / areas for improvement**	
**Actions agreed**	
**Additional notes**	

WOMEN CHILDREN FIRST (UK)

5. RESOURCE MOBILISATION

**Criteria for assessment**	***Are the groups able to mobilise resources to implement their solutions? Are they able to mobilise resources from within the groups and/or outside the groups?***
**Score / Average score assigned**	
**Identified successes**	
**Identified concerns / areas for improvement**	
**Actions agreed**	
**Additional notes**	

6. ABILITY TO ASK "WHY"?

**Criteria for assessment**	***Do groups try to address root causes of the problems they face? If so, how well? By root causes we are referring to meeting re: contributing factors – the broader causes of ill health such as poverty.***
**Score / Average score assigned**	
**Identified successes**	
**Identified concerns / areas for improvement**	
**Actions agreed**	
**Additional notes**	

WOMEN AND CHILDREN FIRST (UK)

7. LINKS TO OTHER ORGANISATIONS / PEOPLE

**Criteria for assessment**	***Are they linking to other stakeholders (people, groups, organisations) who can help them solve identified issues? If so, who are they linking with and why?***
**Score / Average score assigned**	
**Identified successes**	
**Identified concerns / areas for improvement**	
**Actions agreed**	
**Additional notes**	

8. RELATIONSHIP WITH OUTSIDE AGENT

**Criteria for assessment**	***Is the external agent – Ministry of Health counterparts – involved and supporting the groups, building their capacity?***
**Score / Average score assigned**	
**Identified successes**	
**Identified concerns / areas for improvement**	
**Actions agreed**	
**Additional notes**	

WOMEN AND CHILDREN FIRST (UK)

9. PROGRAMME MANAGEMENT

**Criteria for assessment**	***Do groups feel they own the groups and their work? Are they capable of continuing the activities once the project comes to an end? Is the NGO implementing partner building group capacity to solve health problems and strengthening them to be sustainable?***
**Score / Average score assigned**	
**Identified successes**	
**Identified concerns / areas for improvement**	
**Actions agreed**	
**Additional notes**	

Direct observation of intervention delivery and CF performance Form:

Did the facilitator deliver the components of the sessions according to the manual?
(Please rate this on scale of 1-4 scale i.e. 1 = no elements of the programme delivered;
2 = some elements of the programme, others missed or inserted;
3 = majority elements of the programme delivered;
4 = all elements of the programme delivered as per handbook).

What are your thoughts about the overall session?

Did you think there are areas for improvement and what where they?

Would you have done anything differently while facilitating the sessions and how?

What advice do you have for the community facilitator?
