# Supplementary material for: Nurture Early for Optimal Nutrition (NEON) participatory learning and action women’s groups to improve infant feeding and practices in South Asian infants: pilot randomised trial study protocol
Source: BMJ Open. 2023 Nov 29;13(11):e063885. doi: 10.1136/bmjopen-2022-063885 (PMC10689384; doi:10.1136/bmjopen-2022-063885)
Supplement: Supplementary data [file bmjopen-2022-063885supp005.pdf]

Development performance of children – (ASQ-3)

Improving lives

NHS
Oxleas
NHS Foundation Trust

Parents of Shiv LOGAN
3 Abbey Terrace
Abbey Wood
London
SE2 9EY

380 D

Oxleas 0 – 19 Years Public Health Nursing Service
Central Administration Team
Tel: 020 8836 8621
Email: oxl-tr.cat@nhs.net

11 Nov 2019

Dear Parents of Shiv,

9 – 12 month review   Appointment confirmation

This letter is to confirm your appointment to review your child's development.

At: Gallions Reach Health Centre Bentham Road Thamesmead London SE28 8BE

On: Tuesday 10 Dec 2019 10:30

Enclosed with this letter are the 'Ages and Stages' questionnaires that are used for this developmental review. Please complete these forms and try out the activities with your baby before the appointment.

It is important that you complete the questionnaires with your child before your appointment and bring the completed questionnaires and Red Book with you on the day.

If you require any help completing this questionnaire or you have any queries about your appointment please contact us on **020 8836 8621**

To find out about Healthy Start benefits search online for the Healthy Start website

Visit our website which was designed by Oxleas Health Care professionals, service users and families to provide you with information about your health and wellbeing and how to access our services.

www.Oxleas.nhs.uk/CYP

Follow us on Twitter @oxleasCYPGre

There is a drop in Health Advice Session open every day from Monday to Friday across Greenwich. For times and venues see Health Visitor Timetable in the link above.

Yours sincerely
Public Health Nursing Service 0 -19 Years

ASQ3 **10 Month Questionnaire** *page 3 of 6*

GROSS MOTOR *(continued)*

		YES	SOMETIMES	NOT YET	
3.	When you stand your baby next to furniture or the cot rail, does she hold on without leaning her chest against the furniture for support? 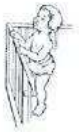	O	O	O	___
4.	While holding onto furniture, does your baby bend down and pick up a toy from the floor and then return to a standing position? 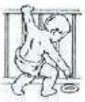	O	O	O	___
5.	While holding onto furniture, does your baby lower himself with control (without falling or flopping down)?	O	O	O	___
6.	Does your baby walk beside furniture while holding on with only one hand?	O	O	O	___
			GROSS MOTOR TOTAL		___

FINE MOTOR

		YES	SOMETIMES	NOT YET	
1.	Does your baby pick up a small toy with only one hand? 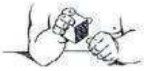	O	O	O	___
2.	Does your baby *successfully* pick up a small cube of bread by using her thumb and all of her fingers in a raking motion? (*If she already picks up a small cube of bread, mark "yes" for this item.*) 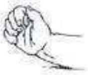	O	O	O	___
3.	Does your baby pick up a small toy with the *tips* of his thumb and fingers? (*You should see a space between the toy and his palm.*) 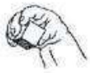	O	O	O	___
4.	After one or two tries, does your baby pick up a piece of string with her first finger and thumb? (*The string may be attached to a toy.*) 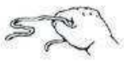	O	O	O	___
5.	Does your baby pick up a small cube of bread with the tips of his thumb and a finger? (*He may rest his arm or hand on the table while doing it.*) 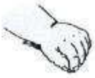	O	O	O	___ *
6.	Does your baby put a small toy down, without dropping it, and then take her hand off the toy?	O	O	O	___
			FINE MOTOR TOTAL		___

* If Fine Motor Item 5 is marked "yes" or "sometimes," mark Fine Motor Item 2 "yes."

BE Av. 1 1.15

Ages & Stages Questionnaires®, Third Edition (ASQ-3™), Squires & Bricker
© 2009-2015 Paul H. Brookes Publishing Co. All rights reserved.
Adaptation into British English prepared with the Department of Health.

OVERALL

Parents and providers may use the space below for additional comments.

1. Does your baby use both hands and both legs equally well? If no, explain: ○ YES ○ NO

2. When you help your baby stand, are his feet flat on the surface most of the time? If no, explain: ○ YES ○ NO

3. Do you have concerns that your baby is too quiet or does not make sounds like other babies? If yes, explain: ○ YES ○ NO

4. Does either parent have a family history of childhood deafness or hearing problems? If yes, explain: ○ YES ○ NO

5. Do you have concerns about your baby's eyesight? If yes, explain: ○ YES ○ NO

6. Has your baby had any medical or health-related problems in the last few months? If yes, explain: ○ YES ○ NO

2999611W1PC0067363/0000690404/

BE Av. 1 1.15

Ages & Stages Questionnaires®, Third Edition (ASQ-3™), Squires & Bricker
© 2009-2015 Paul H. Brookes Publishing Co. All rights reserved.
Adaptation into British English prepared with the Department of Health.

4-day Food Diary

DAY 1

Day 1:		Date:		
Time	Where? With Whom? TV on? At table?	Food/Drink description & preparation	Brand Name	Portion size or quantity eaten
How to describe what you had and how much you had can be found on pages 16 - 21				
6am to 9am				
9am to 12 noon				

Time	Where? With Whom? TV on? At table?	Food/Drink description & preparation	Brand Name	Portion size or quantity eaten
12 noon to 2pm				
2pm to 5pm				

Time	Where? With Whom? TV on? At table?	Food/Drink description & preparation	Brand Name	Portion size or quantity eaten
		5pm to 8pm		
		8pm to 10pm		
		10pm to 6am		

Was the amount of **food** that you had today about what you usually have, less than usual, or more than usual?

Yes, usual ☐ No, **less** than usual ☐ No, **more** than usual ☐

Please tell us why you had less than usual

Please tell us why you had more than usual

Was the amount you had to **drink** today, including water, tea, coffee and soft drinks [and alcohol], about what you usually have, less than usual, or more than usual?

Yes, usual ☐ No, **less** than usual ☐ No, **more** than usual ☐

Please tell us why you had less than usual

Please tell us why you had more than usual

Did you **finish all the food and drink** that you recorded in the diary today?

Yes ☐ No ☐

If no, please **go back to the diary and make a note of any leftovers**

Did you take any **vitamins, minerals or other food supplements** today?

Yes ☐ No ☐

If yes, **please describe the supplements you took below**

Brand	Name (in full) including strength	Number of pills, capsules, teaspoons

Please record on the next pages details of any recipes or (if not already described) ingredients of made up dishes or take-away dishes.

**Write in recipes or ingredients of made up dishes or take-away dishes**			
NAME OF DISH:		***Serves:***	
Ingredients	***Amount***	***Ingredients***	***Amount***
Brief description of cooking method			

Write in recipes or ingredients of made up dishes or take-away dishes			
NAME OF DISH:		*Serves:*	
Ingredients	*Amount*	*Ingredients*	*Amount*
Brief description of cooking method			

DAY 2

Day 2:		Date:		
Time	Where?<br>With Whom?<br>TV on?<br>At table?	Food/Drink description & preparation	Brand Name	Portion size or quantity eaten
How to describe what you had and how much you had can be found on pages 16 - 21				
6am to 9am				
9am to 12 noon				

Time	Where?<br>With Whom?<br>TV on?<br>At table?	Food/Drink description & preparation	Brand Name	Portion size or quantity eaten
		12 noon to 2pm		
		2pm to 5pm		

Time	Where?<br>With Whom?<br>TV on?<br>At table?	Food/Drink description & preparation	Brand Name	Portion size or quantity eaten
		5pm to 8pm		
		8pm to 10pm		
		10pm to 6am		

Was the amount of **food** that you had today about what you usually have, less than usual, or more than usual?

Yes, usual ☐ No, **less** than usual ☐ No, **more** than usual ☐

Please tell us why you had less than usual

Please tell us why you had more than usual

Was the amount you had to **drink** today, including water, tea, coffee and soft drinks [and alcohol], about what you usually have, less than usual, or more than usual?

Yes, usual ☐ No, **less** than usual ☐ No, **more** than usual ☐

Please tell us why you had less than usual

Please tell us why you had more than usual

Did you **finish all the food and drink** that you recorded in the diary today?

Yes ☐ No ☐

If no, please **go back to the diary and make a note of any leftovers**

Did you take any **vitamins, minerals or other food supplements** today?

Yes ☐ No ☐

If yes, **please describe the supplements you took below**

Brand	Name (in full) including strength	Number of pills, capsules, teaspoons

Please record on the next pages details of any recipes or (if not already described) ingredients of made up dishes or take-away dishes.

Write in recipes or ingredients of made up dishes or take-away dishes			
NAME OF DISH:		***Serves:***	
Ingredients	***Amount***	***Ingredients***	***Amount***
Brief description of cooking method			

Write in recipes or ingredients of made up dishes or take-away dishes			
NAME OF DISH:		***Serves:***	
Ingredients	***Amount***	***Ingredients***	***Amount***
Brief description of cooking method			

DAY 3

Day 3:		Date:		
Time	Where? With Whom? TV on? At table?	Food/Drink description & preparation	Brand Name	Portion size or quantity eaten
How to describe what you had and how much you had can be found on pages 16 - 21				
6am to 9am				
9am to 12 noon				

Time	Where? With Whom? TV on? At table?	Food/Drink description & preparation	Brand Name	Portion size or quantity eaten
12 noon to 2pm				
2pm to 5pm				

Time	Where? With Whom? TV on? At table?	Food/Drink description & preparation	Brand Name	Portion size or quantity eaten
		5pm to 8pm		
		8pm to 10pm		
		10pm to 6am		

Was the amount of **food** that you had today about what you usually have, less than usual, or more than usual?

Yes, usual ☐ No, **less** than usual ☐ No, **more** than usual ☐

Please tell us why you had less than usual

Please tell us why you had more than usual

Was the amount you had to **drink** today, including water, tea, coffee and soft drinks [and alcohol], about what you usually have, less than usual, or more than usual?

Yes, usual ☐ No, **less** than usual ☐ No, **more** than usual ☐

Please tell us why you had less than usual

Please tell us why you had more than usual

Did you **finish all the food and drink** that you recorded in the diary today?

Yes ☐ No ☐

If no, please **go back to the diary and make a note of any leftovers**

Did you take any **vitamins, minerals or other food supplements** today?

Yes ☐ No ☐

If yes, **please describe the supplements you took below**

Brand	Name (in full) including strength	Number of pills, capsules, teaspoons

Please record on the next pages details of any recipes or (if not already described) ingredients of made up dishes or take-away dishes.

Write in recipes or ingredients of made up dishes or take-away dishes			
NAME OF DISH:		***Serves:***	
Ingredients	***Amount***	***Ingredients***	***Amount***
Brief description of cooking method			

Write in recipes or ingredients of made up dishes or take-away dishes			
NAME OF DISH:		***Serves:***	
Ingredients	***Amount***	***Ingredients***	***Amount***
Brief description of cooking method			

DAY 4

Please remember to complete the general questions on pages 61-66!

**Day 4:**		**Date:**		
**Time**	**Where?<br>With Whom?<br>TV on?<br>At table?**	**Food/Drink description & preparation**	**Brand Name**	**Portion size or quantity eaten**
How to describe what you had and how much you had can be found on pages 16 - 21				
6am to 9am				
9am to 12 noon				

Time	Where? With Whom? TV on? At table?	Food/Drink description & preparation	Brand Name	Portion size or quantity eaten
		12 noon to 2pm		
		2pm to 5pm		

Time	Where? With Whom? TV on? At table?	Food/Drink description & preparation	Brand Name	Portion size or quantity eaten
		5pm to 8pm		
		8pm to 10pm		
		10pm to 6am		

Was the amount of **food** that you had today about what you usually have, less than usual, or more than usual?

Yes, usual ☐ No, **less** than usual ☐ No, **more** than usual ☐

Please tell us why you had less than usual

Please tell us why you had more than usual

Was the amount you had to **drink** today, including water, tea, coffee and soft drinks [and alcohol], about what you usually have, less than usual, or more than usual?

Yes, usual ☐ No, **less** than usual ☐ No, **more** than usual ☐

Please tell us why you had less than usual

Please tell us why you had more than usual

Did you **finish all the food and drink** that you recorded in the diary today?

Yes ☐ No ☐

If no, please **go back to the diary and make a note of any leftovers**

Did you take any **vitamins, minerals or other food supplements** today?

Yes ☐ No ☐

If yes, **please describe the supplements you took below**

Brand	Name (in full) including strength	Number of pills, capsules, teaspoons

Please record on the next pages details of any recipes or (if not already described) ingredients of made up dishes or take-away dishes.

Write in recipes or ingredients of made up dishes or take-away dishes			
NAME OF DISH:		***Serves:***	
Ingredients	***Amount***	***Ingredients***	***Amount***
Brief description of cooking method			

Write in recipes or ingredients of made up dishes or take-away dishes			
NAME OF DISH:		***Serves:***	
Ingredients	***Amount***	***Ingredients***	***Amount***
Brief description of cooking method			

General questions about your food/ drink during the recording period.

Special diet

1. Did you follow a special diet during the recording period e.g. vegetarian, cholesterol lowering, weight reducing?

Yes ☐ *Please specify* No ☐

Milk

2. Which type of milk did you use most often during the recording period?

Whole, fresh, pasteurised ☐ Semi-skimmed fresh, pasteurised ☐ Skimmed (fat free) fresh, pasteurised ☐ 1% fat milk, pasteurised ☐

Dried ☐ *Type* Soya ☐ *Type*

Other ☐ *Type* Did not use ☐

Tea and coffee

3. How much milk did you usually have in coffee/ tea?

Coffee A lot ☐ Some ☐ A little ☐ None/did not drink ☐

Tea A lot ☐ Some ☐ A little ☐ None/did not drink ☐

4. Did you usually sweeten your coffee/ tea with sugar?

Coffee Yes ☐ How many teaspoons in a mug/cup? ☐ No/did not drink ☐

Tea Yes ☐ How many teaspoons in a mug/cup? ☐ No/did not drink ☐

5. Did you usually sweeten your coffee/ tea with artificial sweetener?

Coffee Yes ☐ How many tablets or teaspoons in a mug/cup? ☐ No/did not drink ☐

Tea Yes ☐ How many tablets or teaspoons in a mug/cup? ☐ No/did not drink ☐

6. Did you drink decaffeinated coffee/ tea during the recording period?

Coffee Always ☐ Sometimes ☐ Never ☐

Tea Always ☐ Sometimes ☐ Never ☐

Manikam L, *et al. BMJ Open* 2023; 13:e063885. doi: 10.1136/bmjopen-2022-063885

Breakfast cereals

7. How much milk did you usually have on breakfast cereal?

Drowned ☐ Average ☐ Damp ☐ None/did not eat ☐

8. How did you usually make your porridge?

With all water ☐ With all milk ☐ With milk and water ☐ Did not eat ☐

9. Did you usually sweeten or salt your porridge?

With sugar ☐ With honey ☐ With salt ☐ Neither/did not eat ☐

10. How did you usually make your instant oat cereal?

With all water ☐ With all milk ☐ With milk and water ☐ Did not eat ☐

11. Did you usually sweeten or salt your instant oat cereal?

With sugar ☐ With honey ☐ With salt ☐ Neither/did not eat ☐

Fats for spreading and cooking

12. Which type of butter, margarine or other fat spread did you use most often during the recording period? Please record the full product name and fat content

Name:

None ☐

e.g. Flora Omega 3 plus, low fat spread, 38% fat, polyunsaturated

13. How thickly did you spread butter, margarine on bread, crackers etc?

Thick ☐ Medium ☐ Thin ☐ N/A ☐

14. Which type of cooking fat/oil did your household use most often over the recording period? Please record the full product name e.g. *Sainsbury's sunflower oil*

Name:

None ☐

Bread

15. Which type of bread did you eat most often during the recording period?

White ☐ Granary ☐ Wholemeal ☐ Brown ☐

50/50 bread e.g. Hovis Best of Both ☐ Other ☐ *Type* Did not eat ☐

16. Was it a large loaf or a small loaf?

Large ☐ Small ☐

17. If the bread was shop bought, how was it sliced?

Thick ☐ Medium ☐ Thin ☐ Unsliced ☐ N/A ☐

Meat

18. If you ate meat during the recording period, did you eat the visible fat?

Always ☐ Sometimes ☐ Never ☐ Did not eat meat ☐

19. If you ate poultry (e.g. chicken, turkey) during the recording period, did you eat the skin?

Always ☐ Sometimes ☐ Never ☐ Did not eat poultry ☐

Fruit and vegetables

20. If you ate apples during the recording period, did you eat the skin?

Always ☐ Sometimes ☐ Never ☐ Did not eat ☐

21. If you ate pears during the recording period, did you eat the skin?

Always ☐ Sometimes ☐ Never ☐ Did not eat ☐

22. If you ate new potatoes during the recording period, did you eat the skin?

Always ☐ Sometimes ☐ Never ☐ Did not eat ☐

23. If you ate baked/jacket potatoes during the recording period, did you eat the skin?

Always ☐ Sometimes ☐ Never ☐ Did not eat ☐

Salt

24. Do you add salt to your food at the table?

Always ☐ Sometimes ☐ Never ☐

25. Do you add salt substitute to your food at the table? *e.g. LoSalt*

Always ☐ Sometimes ☐ Never ☐

Water

26. Which type of water did you drink most often during the recording period?

Tap ☐ Filtered ☐ Bottled ☐ *brand* ______ Did not drink ☐

Thank you for completing this diary.

Children Feeding Behaviour

ID:

Child Eating Behaviour Questionnaire (CEBQ)

Please read the following statements and tick the boxes most appropriate to your child's eating behaviour.

	Never	Rarely	Some-times	Often	Always
My child loves food	☐	☐	☐	☐	☐
My child eats more when worried	☐	☐	☐	☐	☐
My child has a big appetite	☐	☐	☐	☐	☐
My child finishes his/her meal quickly	☐	☐	☐	☐	☐
My child is interested in food	☐	☐	☐	☐	☐
My child is always asking for a drink	☐	☐	☐	☐	☐
My child refuses new foods at first	☐	☐	☐	☐	☐
My child eats slowly	☐	☐	☐	☐	☐
My child eats less when angry	☐	☐	☐	☐	☐
My child enjoys tasting new foods	☐	☐	☐	☐	☐
My child eats less when s/he is tired	☐	☐	☐	☐	☐
My child is always asking for food	☐	☐	☐	☐	☐
My child eats more when annoyed	☐	☐	☐	☐	☐
If allowed to, my child would eat too much	☐	☐	☐	☐	☐
My child eats more when anxious	☐	☐	☐	☐	☐
My child enjoys a wide variety of foods	☐	☐	☐	☐	☐
My child leaves food on his/her plate at the end of a meal	☐	☐	☐	☐	☐
My child takes more than 30 minutes to finish a meal	☐	☐	☐	☐	☐

	Never	Rarely	Some-times	Often	Always
Given the choice, my child would eat most of the time	☐	☐	☐	☐	☐
My child looks forward to mealtimes	☐	☐	☐	☐	☐
My child gets full before his/her meal is finished	☐	☐	☐	☐	☐
My child enjoys eating	☐	☐	☐	☐	☐
My child eats more when she is happy	☐	☐	☐	☐	☐
My child is difficult to please with meals	☐	☐	☐	☐	☐
My child eats less when upset	☐	☐	☐	☐	☐
My child gets full up easily	☐	☐	☐	☐	☐
My child eats more when s/he has nothing else to do	☐	☐	☐	☐	☐
Even if my child is full up s/he finds room to eat his/her favourite food	☐	☐	☐	☐	☐
If given the chance, my child would drink continuously throughout the day	☐	☐	☐	☐	☐
My child cannot eat a meal if s/he has had a snack just before	☐	☐	☐	☐	☐
If given the chance, my child would always be having a drink	☐	☐	☐	☐	☐
My child is interested in tasting food s/he hasn't tasted before	☐	☐	☐	☐	☐
My child decides that s/he doesn't like a food, even without tasting it	☐	☐	☐	☐	☐
If given the chance, my child would always have food in his/her mouth	☐	☐	☐	☐	☐
My child eats more and more slowly during the course of a meal	☐	☐	☐	☐	☐

Parental Feeding Style

Parental Feeding Style Questionnaire

Please read the following statements and tick the appropriate boxes to show how you deal with feeding your child. It is important to remember that there are no right or wrong answers to these questions, we are interested in what parents really feel and do.

ID

	Never	Rarely	Some-times	Often	Always
I allow my child to choose which foods to have for meals	☐	☐	☐	☐	☐
I give my child something to eat to make him/her feel better when s/he is feeling upset	☐	☐	☐	☐	☐
I encourage my child to look forward to the meal	☐	☐	☐	☐	☐
I praise my child if s/he eats what I give him/her	☐	☐	☐	☐	☐
I decide how many snacks my child should have	☐	☐	☐	☐	☐
I encourage my child to eat a wide variety of foods	☐	☐	☐	☐	☐
In order to get my child to behave him/herself I promise him/her something to eat	☐	☐	☐	☐	☐
I present food in an attractive way to my child	☐	☐	☐	☐	☐
If my child misbehaves I withhold his/her favourite food	☐	☐	☐	☐	☐
I encourage my child to taste each of the foods I serve at mealtimes	☐	☐	☐	☐	☐
I allow my child to wander around during a meal	☐	☐	☐	☐	☐
I encourage my child to try foods that s/he hasn't tasted before	☐	☐	☐	☐	☐
I give my child something to eat to make him/her feel better when s/he has been hurt	☐	☐	☐	☐	☐
I let my child decide when s/he would like to have her meal	☐	☐	☐	☐	☐
I give my child something to eat if s/he is feeling bored	☐	☐	☐	☐	☐
I allow my child to decide when s/he has had enough snacks to eat	☐	☐	☐	☐	☐
I decide when it is time for my child to have a snack	☐	☐	☐	☐	☐
I use puddings as a bribe to get my child to eat his/her main course	☐	☐	☐	☐	☐
I encourage my child to enjoy his/her food	☐	☐	☐	☐	☐

PTO

Network Diffusion Form

(To be completed by participants at the end of each PLA session and at 6 months from baseline)

1. How many people did you share your material/information from the PLA sessions with?..

2. How did you share this information/material (verbal, social media, whatsapp etc)?..

3. Please fill the table below with details of the people you shared the information with -

Serial number	Relation to you	Age/Gender	Family size	How did you share (verbal, social media, whatsapp)

Equality Impact Assessment

Equality Impact Assessment- NEON

Question	Response
**1. Name of intervention being assessed**	NEON Intervention (PLA Cycle)
**2. Summary of aims and objectives of the intervention**	To improve infant feeding, care and dental hygiene practices amongst the South Asian Communities of Tower Hamlets, Newham, and Waltham Forest
**3. What involvement and consultation has been done in relation to this intervention?** *(e.g., with relevant groups and stakeholders)*	
**4. Who is affected by the intervention?**	Infants (aged<24 months)<br>Mothers<br>Pregnant women<br>Carers- Grandmother etc.
**5. What are the arrangements for monitoring and reviewing the actual impact of the intervention?**	

Protected Characteristic Group	Is there a potential for positive or negative impact?	Please explain and give examples of any evidence/data used	Action to address negative impact (e.g. adjustment to the intervention)
**Disability**			
**Gender reassignment**			
**Marriage or civil partnership**			
**Pregnancy and maternity**			
**Race**			
**Religion or belief**			
**Sexual orientation**			
**Sex (gender)**			
**Age**			

Evaluation:

Question	Explanation / justification
Is it possible the proposed intervention could discriminate or unfairly disadvantage people?	

Final Decision:	Tick the relevant box	Include any explanation / justification required
1. No barriers identified, therefore activity will **proceed**.		
2. You can decide to **stop** the intervention at some point because the data shows bias towards one or more groups		
3. You can **adapt or change** the intervention in a way which you think will eliminate the bias		
4. Barriers and impact identified, however having considered all available options carefully, there appear to be no other proportionate ways to achieve the aim of the intervention (e.g., in extreme cases or where positive action is taken). Therefore, you are going to **proceed with caution** with this intervention knowing that it may favour some people less than others, providing justification for this decision.		

**Date completed:**	
**Completed by whom (Borough/individual):**	

Staff time questionnaire

Measurement of Time Use for staff involved in *NEON*

This questionnaire is for collecting information on how staff from the study team who are contributing to the Nurture Early for Optimal Nutrition (*NEON*) divide their time between the different trial components. We aim to collect this information from all our partners staff involved in *NEON* over the duration of the 3-year of the study.

The information you provide will only be used for research purposes only. It ***will not*** be used to monitor performance or to plan future activities. The information you provide will help us to divide the total *NEON* cost into the following components: intervention, M&E, process evaluation, and research. This will allow us to report the cost effectiveness of the *NEON* intervention. We also want to separate out the setting up cost from the implementation cost, to inform others who may wish to implement these interventions.

Your name will not be used in the analysis but you will be identifiable through your answers. If you do not want to proceed, or wish to stop at any time, you can do so. If you have any questions, please discuss with Shereen Al Laham (*s.laham@ucl.ac.uk*).

==

Name of Staff Member: ....

Job title / role: ....

Institution: ....

Date: ....

---

1. When did your involvement with *NEON* first begin? .... (Month/Year)
2. Since you started, have you been involved in any project other than *NEON*?

   ☐ Yes

   ☐ No. **Go to question 6.**

3. If Yes, how many days did you spend on *NEON* in the last 30 days?

   .... Days OR .... Percent of time

4. Was this a typical month for you, since you started work on *NEON*?

   ☐ Yes. **Go to question 6.**

   ☐ No

5. If No, please tell me how you have divided your time between *NEON* and any other project since you first started on *NEON*.

   ....

   ....

   (E.g: On average, 2 days per week from January to May, then full-time since June. Answer can be in percentage, days, or weeks – whatever you think most accurately represents your actual time use.)

6. Thinking now about the **last 30 days**, and only those days you worked on *NEON*, how have you in practice divided your time between the following *NEON* activities?

Page **1** of **2**

a) I spent .... days OR .... percent time on Monitoring and Evaluation.
b) I spent .... days OR .... percent time on study delivery and implementation.
c) I spent .... days OR .... percent time on Research Work, that is, working on the study design, reading the literature, or writing up or presenting the research findings.
d) I spent .... days OR .... percent time on Joint Work, that is, it is not possible to say it was one of the above. For example, general management or support functions.

7. Is this how you usually divide your time?

☐ Yes. **Go to question 9.**

☐ No

8. If No, please tell me how you have divided your time between the different *NEON* activities since you first started on *NEON*. *(Use the categories in question 6.)*

....

....

....

(E.g: 50% Joint work for the first 6 months, then 1 day per week Joint work but otherwise only NEON. Answer can be in percentage, days, or weeks – whatever you think most accurately represents your actual time use.)

9. Finally, I want to ask you about the division of time between **setting up** and **implementation** of the *NEON* study. Examples of setting up activities are: recruiting and training; developing and testing the intervention material. These activities may still be ongoing.
Please could you tell us when you start to work on implementation, and do setting up activities still continue

	**Activity**	**Start date of implementation** *(Month/Year)*	**Time spend on SETTING UP activities since start date** *(days per week or percentage of time)*
a	NEON intervention implementation		
b	Monitoring and Evaluation implementation		
c	Process Evaluation implementation		

== Thank You ==
