# Supplementary material for: Nurture Early for Optimal Nutrition (NEON) participatory learning and action women’s groups to improve infant feeding and practices in South Asian infants: pilot randomised trial study protocol
Source: BMJ Open. 2023 Nov 29;13(11):e063885. doi: 10.1136/bmjopen-2022-063885 (PMC10689384; doi:10.1136/bmjopen-2022-063885)
Supplement: Author's manuscript [file bmjopen-2022-063885.draft_revisions.pdf]

BMJ Open

BMJ Open is committed to open peer review. As part of this commitment we make the peer review history of every article we publish publicly available.

When an article is published we post the peer reviewers' comments and the authors' responses online. We also post the versions of the paper that were used during peer review. These are the versions that the peer review comments apply to.

The versions of the paper that follow are the versions that were submitted during the peer review process. They are not the versions of record or the final published versions. They should not be cited or distributed as the published version of this manuscript.

BMJ Open is an open access journal and the full, final, typeset and author-corrected version of record of the manuscript is available on our site with no access controls, subscription charges or pay-per-view fees (http://bmjopen.bmj.com).

If you have any questions on BMJ Open's open peer review process please email info.bmjopen@bmj.com

BMJ Open

Nurture Early for Optimal Nutrition (NEON) Pilot Feasibility Randomised Controlled Trial: Community facilitator led participatory learning and action (PLA) women's groups to improve infant feeding, care and dental hygiene practices in South Asian infants aged < 2 years in East London

Journal:	*BMJ Open*
Manuscript ID	bmjopen-2022-063885
Article Type:	Protocol
Date Submitted by the Author:	28-Apr-2022

Complete List of Authors:	Manikam, Logan; UCL Institute of Epidemiology and Health Care, Epidemiology and Public Health; Aceso Global Health Consultants<br>Allaham, Shereen ; UCL Institute of Epidemiology and Health Care, Department of Epidemiology and Public Health; Aceso Global Health Consultants Ltd,<br>Ong, Zhen Ling; Aceso Global Health Consultants Limited<br>Demel, Isabel-Cathérine; King's College London Faculty of Life Sciences and Medicine, Guy's, King's and St.Thomas' School of Medicine<br>Heys, Michelle ; University College London Institute of Child Health, Population, Policy and Practice<br>Batura, Neha; University College London, Institute for Global Health<br>Llewellyn, Clare; University College London Department of Behavioural Science and Health<br>Hayward, Andrew; University College London, Epidemiology and Public Health<br>Lakshman, Rajalakshmi; Cambridgeshire County Council, Public Health Directorate; University of Cambridge, Medical Research Council Epidemiology Unit<br>Gilmour, Jenny; Mile End Hospital, Tower Hamlets GP Care Group<br>Webb-Martin, Kelley; London Borough of Newham, Children's Health 0-19 Service<br>Irish, Carol; London Borough of Newham, Children's Health 0-19 Service<br>Archibong, Mfon; London Borough of Newham, Children's Health 0-19 Service<br>Clarkson, Corinne; London Borough of Waltham Forest, Council, Public Health Strategist for Maternal & Child Health<br>Delceta, Daley; Chingford Medical Practice, Operational Lead<br>Islam, Lily; London Borough of Tower Hamlets, Community Facilitator<br>Bajwa, Seema; London Borough of Newham, Community Facilitator<br>Malek, Sabiha; London Borough of Newham, Community Facilitator<br>Bhachu, Jasvir; London Borough of Newham, Community Facilitator<br>Pushpakanthan, Geromini; London Borough of Newham, Community Facilitator<br>Lakhanpaul, Monica; UCL Great Ormond Street Institute of Child Health Population Policy and Practice, Institute of Child Health; Whittington Health NHS Trust

Keywords:	Community child health < PAEDIATRICS, NUTRITION & DIETETICS, PAEDIATRICS, PUBLIC HEALTH

SCHOLARONE™ Manuscripts

Nurture Early for Optimal Nutrition (NEON) Pilot Feasibility Randomised Controlled Trial: Community facilitator led participatory learning and action (PLA) women's groups to improve infant feeding, care and dental hygiene practices in South Asian infants aged < 2 years in East London

Short Title/ Acronym: Nurture Early for Optimal Nutrition (NEON) Pilot Feasibility Randomised Controlled Trial

Authors:

[1]Dr Logan Manikam, NIHR Advanced Fellow & Honorary Consultant in Public Health Medicine[1,2]

Shereen Allaham, Research Assistant & consultant [1,2]

Zhen Ling Ong, Associate[2]

Isabel-Cathérine Demel, Medical Student[2,3]

Dr Michelle Heys, Associate Professor of Community and Population Child Health, Consultant Paediatrician [4,5]

Dr Neha Batura, Associate Professor in Health Economics[6]

Dr Clare Llewellyn, Associate Professor in Behavioural Obesity Research[7]

Prof Andrew Hayward, Professor of Infectious Disease Epidemiology and Inclusion Health Research[1]

Dr Rajalakshmi Lakshman, Consultant in Public Health Medicine[8]

Jenny Gilmour, 0-19 Service Development Lead THGPCG[9]

Kelley Webb Martin, Director of Delivery, Compliance & Transformation[10]

Carol Irish, Head of Children's Health 0-19 Service[10]

Mfon Archibong, Specialist Health Visiting Research Lead[10]

Corinne Clarkson, Public Health Strategist for Maternal & Child Health[11]

Daley Delceta, Operational Lead[12]

Lily Islam, Tower Hamlets community facilitator

Seema Bajwa, Newham community facilitator

Sabiha Malek, Newham community facilitator

Jasvir Bhachu, Newham community facilitator

Geromini Pushpakanthan, Newham community facilitator

[1] Logan Manikam and Shereen Al Laham are joint first authors

For peer review only

[2]Prof Monica Lakhanpaul, Professor of Integrated Community Child Health[13,14]

Affiliations:

1. Department of Epidemiology and Public Health, University College London Institute of Epidemiology and Health Care, London, UK
2. Aceso Global Health Consultants Limited, London, UK
3. Department of Life Sciences & Medicine, King's College London GKT School of Medical Education, London, UK
4. Specialist Children and Young People's Services, East London NHS Foundation Trust, 84 West Ham Lane, London E15 4PT.
5. Population, Policy and Practice, UCL Great Ormond Street Institute of Child Health, 30 Guildford Street, London WC1N 1EH, UK
6. Institute for Global Health, University College London, 30 Guilford Street, London WC1N 1EH.
7. Department of Behavioural Sciences & Health, UCL IEHC, 1-19 Torrington Pl, Fitzrovia, London WC1E 7HB.
8. MRC Epidemiology Unit, University of Cambridge
9. Tower Hamlets GP Care Group, Mile End Hospital, London, UK.
10. Children's Health 0-19 Service, London Borough of Newham, 1000 Dockside Road E16 2QU
11. Waltham Forest Council, London, UK
12. Chingford Health Centre Waltham Forest, London, UK
13. Population, Policy and Practice, UCL Great Ormond Street Institute of Child Health, University College London, London, WC1N 1EH, UK
14. Whittington Health NHS Trust, London, UK, N19 5NF

Corresponding author: Dr Logan Manikam

Email: logan.manikam.10@ucl.ac.uk

Telephone: +44 7868721152

Key Words: Community-based intervention; infant; child health; nutrition; feeding practices; clinical trial

Word Count: 4000

[2] Monica Lakhanpaul is the senior author

For peer review only

ABSTRACT

Introduction: Feeding practices developed in early life can impact a child's nutrition, growth, dental health, cognitive development and lifetime risk of chronic diseases. Substantial evidence suggests ethnic health inequalities, and non-recommended complementary infant feeding practices among UK's South Asian(SA) population. NEON aims to use Women's group Participatory Learning and Action(PLA) cycles to optimise infant feeding, care and dental hygiene practices in SA infants <2 years in East London.

Methods and analysis: A three-arm pilot feasibility cluster randomised controlled trial RCT) will assess feasibility, acceptability, costs and explore preliminary effectiveness for proposed primary outcome(BMI z-score). Multilingual SA community facilitators(CFs) will deliver the intervention, the NEON Women's Group PLA Cycle, to mothers/carers of infants in respective ethnic/language groups. 18 wards are randomised to face-to-face PLA, online PLA and usual care arms in 1:1:1 ratio. Primary outcomes are feasibility and process measures(i.e. recruitment, retention rates, intervention support, acceptability, fidelity, adherence, sustainability) for assessment against Go/Stop criteria. Secondary outcomes are proposed individual-, cluster-level and economic outcomes for the definitive trial(i.e. child BMI z-scores, eating behaviour, parental feeding practices, network diffusion, children development performance, level of dental caries, GP utilisation, costs, staff time). Outcomes are measured at baseline, every two weeks during intervention, 14-weeks(end of PLA cycle),6-months and 12-months by blinded outcome assessors where possible.

This study will use concurrent mixed-methods evaluation. Quantitative analyses include descriptive summary with 95% confidence interval(CI) and sample size calculation for the definitive trial. The intervention effect with CI will be estimated for child BMI z-score. Implementation will be evaluated qualitatively using thematic framework analysis.

Ethics and dissemination: Ethics approval will be obtained from UCL and NHS. Results will be published in peer-reviewed journals, presented at scientific conferences and workshops with commissioners, partners and participating communities. Plain language summaries will be disseminated through community groups, websites and social media.

Word count: 299/300

Trial registration: This trial will be registered with the International Standard Randomised Controlled Trial Number Register (ISRCTN).

For peer review only

ARTICLE SUMMARY

Strengths

- This study will assess the feasibility of delivering culturally-tailored early life interventions, developed through participatory approaches, in a real-world setting.
- This pilot feasibility study will inform a definitive trial to compare the approach of utilising community assets and champions to usual care as an innovative, scalable health service model to reduce ethnic health inequalities.
- Assessing a range of quantitative and qualitative process, economic, individual- and cluster-level outcome measurements will provide rich contextual data to improve and address issues that arise during trial implementation.

Limitations

- Triangulating data from participant self-report, implementers' report, structured observation by independent observers and routine data could improve outcome measurement.
- Due to the nature of the intervention, neither participants nor all research staff will be fully masked to allocation, which may introduce the risk of performance or measurement bias.

For peer review only

INTRODUCTION

The first 1,000 days of life is important for growth and brain development. There is mounting evidence that influences during pregnancy and infancy may alter lifetime risk of nutrition and dental related diseases [1]. Feeding practices developed during this period can impact children's nutrition, growth, dental health, cognitive development, and may lead eventually to heart disease, obesity, and diabetes [2].

Britain's ethnically diverse population is mostly disadvantaged across a range of socioeconomic outcomes, forming fundamental causes of ethnic health inequalities in the UK [3]. Some of the widest differences have been observed in the South Asian (SA) population, more so for Pakistani and Bangladeshi communities compared to Indians [4].

Systematic reviews assessing complementary feeding practices (CFP) and the sociocultural beliefs underpinning them in children <2 years within SA families in the UK were explored. Despite the adoption of the WHO Infant and Young Children Feeding Guidelines, there remains substantial evidence of non-recommended complementary feeding practices being followed [5-7]. Contributing factors that persisted post-migration included bicultural issues or low acculturation levels and conflicting information among health professionals, extended family, and religious and community leaders.

Effective early life interventions tailored to different ethnic groups have great potential to reduce the development of short- and long-term conditions and, thereby, lifetime inequalities. At present, however, few of such interventions exist [8,9]. Traditionally, UK health services provide unidirectional information based on guidelines and NHS recommendations. Specific ethnic groups may be marginalised by this approach as most advice is not tailored to their cultural practices. Based on Lakhanpaul et al.'s MARKS (Motivation Awareness Resources Knowledge and Skills) model [10], current practice lacks the space to understand parents' motivation and is unable to support families in skills development, all of which is key to providing optimal infant feeding, care, and dental hygiene support. Considering the resource constraints of the NHS and its 10 Year Forward Plan which aims to shift the emphasis to the community, highlights a need for interventions that utilise community assets.

The Participatory Learning and Action (PLA) cycle is an iterative process led by multilingual facilitators through a four-stage cycle of identifying and prioritising contextual issues, designing problem-solving strategies, implementing these strategies and a post-implementation evaluation. The PLA approach is a low-cost, community-based, culturally sensitive intervention that can be adapted from Low- and Middle-income Countries (LMICs) to High-income Countries (HICs), as well as different population groups and topic areas. Community mobilisation through facilitated Women's Group PLA Cycles is a strategy recommended by the WHO to improve maternal and infant survival [11]. A meta-analysis of seven cluster randomised controlled trials (RCTs) utilising PLA in LMICs showed reduced maternal and neonatal mortality by 37% and 23% respectively [12]. An RCT and controlled before-and-after study have demonstrated reduced maternal depression, increased exclusive breastfeeding rates, and decreased under-5 morbidity [13]. A Mumbai-based RCT also showed improvements in maternal practices and care-seeking behaviour [14]. Recognising the success of PLA in LMICs and its official recommendation by the WHO, this approach was adopted for the NEON programme.

For peer review only

In NEON Phase 1 (Intervention Development), the NEON Women's Group PLA Cycle intervention was co-developed with the SA community facilitators (CF) (now community researchers/independent observers in NEON Phase 2), community members, research team and independent experts (i.e. health visitors, dentists, dieticians, Bengali nutritional advocates, GPs, and midwives). This followed the MRC complex intervention framework starting from the use of best available evidence and appropriate theory during the Intervention Development phase. This adaptation involved using formative research and prior trials as evidence-based factors; micro-adaptations involved adjusting language, literacy levels for materials and using picture cards, face-to-face, and interactive learning delivery methods.

This protocol describes NEON Phase 2: Pilot Feasibility Cluster RCT.

AIMS AND OBJECTIVES

This pilot feasibility study aims to evaluate the feasibility and inform the design and conduct of a definitive cluster RCT comparing NEON Women's Group PLA Cycle versus usual care to optimise infant feeding, care, and dental hygiene practices in SA infants aged <2 years in East London.

Primary objectives:

- To assess the feasibility of proceeding to a definitive trial against predetermined Go/Stop criteria (i.e. recruitment, retention rates, intervention support, acceptability)
- To assess intervention fidelity and participants' adherence
- To assess the implementation of face-to-face versus online intervention arms

Secondary objectives:

- To assess feasibility, completeness, acceptability and adequacy of blinding when collecting proposed outcomes for a definitive trial to establish optimal outcomes and data collection procedures
- To assess the time needed to competently deliver both versions of NEON intervention
- To assess the mean, standard deviation and intervention effect with 95% confidence interval (CI) of the proposed primary outcome, child BMI z-score

METHODS AND ANALYSIS

Design

This three-arm pilot feasibility cluster RCT study is expected to run from July 2021-May 2023 in 3 East London boroughs. 18 clusters defined as borough wards will be randomised with 1:1:1 allocation to 2 intervention arms (face-to-face or online NEON Women's Group PLA Cycle) and 1 control arm (usual care). An online arm enables the trial to continue under Covid-19 restrictions; usual care was selected as control to avoid depriving participants of essential and available infant and maternal care services. Multilingual CFs will deliver interventions in respective ethnic/language groups. Ward-level randomisation is a trade-off between minimising contamination and ensuring maximum SA population representation given the diversity in the ethnic make up, i.e. SA sub-groups, of the aforementioned boroughs.

For peer review only

Patient and public involvement

The research has been co-designed with SA CFs involved in all stages of developing and evaluating the intervention to ensure relevant research questions and an acceptable study design. 5 CFs from the Intervention Development phase, representing different SA communities, are now recruited as Community Researchers (CRs) to support;

- protocol development and ethics application by ensuring participants-facing documents are clear, appropriate and sensitively-worded;
- topic guide and questionnaire development;
- strategy development and troubleshooting (e.g. recruitment);
- interpretation of findings into appropriate and attainable recommendations for practice;
- review and revision of draft academic papers;
- dissemination activities and development of plain language summaries.

Setting

This study will run sequentially in the London Boroughs of Tower Hamlets (TH), Newham (NH) and Waltham Forest (WF), all of which are considered to be among 5% most deprived in England [15]. The boroughs were analysed in terms of ethnic make-up; in each borough, the 6 wards with the highest density of Asian population were selected based on census data [16]. The most common SA ethnic/language groups will be targeted. Figure 1 shows the number of wards and PLA groups by boroughs. Figure 2 shows the overview of participant flow throughout the trial.

Figure 1. Overview schematic detailing number of wards & PLA groups by East London boroughs

Figure 2. Overview of the NEON pilot feasibility RCT design and participant flow

Randomisation

The research assistant (RA) will randomly allocate clusters in 1:1:1 allocation to face-to-face, online and control arms using Research Randomizer software [17] for the 18 wards stratified by borough and hold the randomisation list. Ward-level randomisation will occur before staff (CF) and participant recruitment. Once sufficient numbers of participants are recruited in an ethnic/language group then that Women's Group PLA cycle can start.

Eligibility Criteria

Participants

Inclusion

- Mothers or female carers of an infant <24 months;
- Indian, Pakistani, Sri Lankan or Bangladeshi background;
- Resident in a randomised study ward in TH, NH and WF;
- Willing and able to provide informed consent

For peer review only

Exclusion

- <18 years old;
- Anticipating moving out of *a priori* defined geographical areas before or after delivery;
- Current or recent participation in another study within 4 weeks of trial commencement.

Staff (Community Facilitator) Eligibility

- Female
- Have at least one child, preferably <24 months
- From the SA community in TH, NH, or WF
- Able to read and write
- Fluent in English and another local language (Hindi, Bengali, Sylheti, Urdu, or Tamil)
- Understand SA social norms, values and culture within study boroughs
- Known and respected by their local community
- Motivated to address issues related to infant growth and development
- Able to manage a group and have some leadership qualities

Sample size

With communication and cultural sensitivity as key drivers of the NEON intervention, the sample size will partly depend on the coverage of ethnic and language groupings. The choice of ethnic/language groups were informed by the 2011 ONS Census [16] and input from CFs stressing that, while language is important for communication, people's practices are embedded in different ethnicities.

The main ethnic/language group(s) identified are: 1 group in TH (Bangladeshi/ Bengali & Sylleti); 4 groups in NH (Indian/Gujrati, Indian/Punjabi, Pakistani/Urdu, and Sri Lankan/Tamil); 3 groups in WF (Indian/Punjabi, Pakistani/Urdu, Sri Lankan/Tamil). In each borough, 1 Women's Group PLA Cycle will run per ethnic/language group per ward.

We aim to run 20 to 32 Women's Group PLA Cycle (with equal numbers of face-to-face and online PLA groups) at 6-8 participants per PLA group. Including control groups totals to 288-384 participants. Assuming an 80% recruitment or retention rate, this enables us to be 85% confident that the true population rate will fall between 0.76%-0.84% (precision=0.04). This was estimated using a geometric mean cluster size=16 participants, cluster size coefficient of variation (COV)=0.7 and Intra-Cluster Correlation Coefficient (ICC)=0.02.

For peer review only

Recruitment

Participants

Due to the diversity of the target population, multiple recruitment methods are needed to maximise reach and minimise inequalities or non-representative samples [18] [19]. For instance, relying solely on online recruitment may be ineffective due to digital poverty. Thus, other mediums including leveraging on existing social networks (snowballing), identifying and fostering collaborations with community leaders, and creating clear and succinct recruitment materials [20] were employed. All study promotional materials (e.g. participants information sheets, posters) will be available in English and local languages. To standardise the recruitment process, we will provide the same recruitment script and study materials to all recruiters. Potential participants will contact the RA, who will confirm eligibility, consent and register them into the study.

Participant recruitment will follow three strategies with regular advice by CRs to maximise reach:

1. *Recruitment through CFs:* CFs will share study materials with their network to invite eligible participants with subsequent snowballing. We will provide training to standardise CF recruitment.
2. *Recruitment through Health Visitor (HVs), GPs, and Midwifery teams*: HVs, GPs, and midwives who have been identified based on their geographical proximity to study wards will share study materials to eligible participants. Details of those interested will be given to the RA.
3. *Online recruitment:* Online social media campaigns on Facebook and Instagram will be utilised. See Supplementary Material-1 for advertisement materials.

Staff

We will recruit CFs from our local stakeholders' network. Recruitment via CFs is particularly useful for studies with underserved or traditionally hard-to-reach populations [21]. CFs are leaders/champions in their local community who, with training and support, help improve the health and wellbeing of their communities. In line with NIHR's "INVOLVE" guideline, CFs will be paid £30 for each PLA meeting and additional £5 to cover travel costs for face-to-face meetings.

HV, GP and midwives are identified and recruited by our study partners at each study ward. Payments will be supported by NHS support costs and decided with the relevant clinical lead.

Allocation concealment and blinding

Firstly, treatment allocation will be concealed from participants and recruiters during participant recruitment to reduce recruitment bias. After recruitment has been completed for that ethnic/ language group in the borough, the RA will reveal ward allocation to participants and CFs via text or email, listing postcodes by trial arms to allow CFs to effectively organise and deliver the intervention. Whether, or not, CFs were in fact unblinded will be verified in the context of a debrief.

For peer review only

Secondly, outcome assessors (CRs) will be blinded where possible to reduce measurement bias. This will be achieved by CRs logging the data collected onto an electronic platform that automatically links participant ID to maintain blinding.

Intervention

NEON Women's Group PLA Cycle

The co-adapted NEON Women's Group PLA Cycle tailored to ethnic/language groups will be delivered face-to-face at community/children's centre or online over 14 weeks (1 session per 2 weeks). HVs, GP, and midwives' teams will be invited to 1-2 sessions to provide evidence-based information upon participants' request. To reduce study contamination, participants may not switch wards (i.e. switch arms) after treatment allocation is revealed. Figure 3 illustrates the aim of each PLA session.

Figure 3. NEON Women's Group PLA Cycle

A culturally-sensitive intervention package has been co-developed consisting of:

1. PLA group facilitator manual
2. Picture cards with recommended and non-recommended infant feeding, care and dental hygiene practices, and facilitators/barriers to uptake
3. Healthy baby food recipes
4. Community asset maps (e.g. low-cost fruit and vegetable shops)
5. List of relevant resources and services.

Online arm participants may access this toolkit through the eRedbook platform and receive NEON information the via the app or website. A Logic Model (Supplementary Material-2) shows the relationship between these activities and resources and their intended short-, medium- and long-term effects.

Control (Usual Care)

In all wards, HV teams have regular mandatory postnatal visits for all families of new-borns and infants. These visits are immediately after birth, 6-8 weeks, 12-16 weeks, 1 year, and 2-2.5 years. One optional prenatal visit is conducted by HVs between 28-32 weeks of pregnancy. Supplementary Material-3 lists other initiatives and resources available in each study borough as part of usual care.

For peer review only

Outcome

Primary outcomes: Feasibility and process outcome measures
We will collect quantitative and qualitative data to assess Go/Stop criteria (Table 1) on recruitment and retention rates, intervention support, acceptability, fidelity and participant adherence using:

1. ***Study record:*** An enrolment log will record all eligible participants, total enrolled, reasons for non-participation, number followed-up on child BMI z-score, and the date on how many people responded to adverts/invitations. Pre-screen failure logs will record those eligible but not enrolled, with reasons. PLA session audio/video recordings will be collected using secured Dictaphone, video cameras, CCTV, and/or Zoom recording.

2. *Process measurements:* These include Participants' Feedback Questionnaire, Facilitator's Feedback Form, PLA Cycle Meeting Register, direct observation of intervention delivery and CF performance by CRs, and Sustainability Assessment Tool (Supplementary Material-4).

Definite Go	Definite Stop
>=50% of eligible participants consenting to pilot feasibility study	<40% of eligible participants consenting to pilot feasibility trial
>=80% of mothers attend >=60% of planned sessions in the intervention arm.	<20% of mothers attend >=60% of sessions as planned in each intervention arm
Retention of >=70% of consented participants for primary outcome data collection	Retention of <50% of participants at 12 months
High intervention support with respect to content, frequency, duration, and quality	Low support of Intervention Procedures
Intervention is perceived as acceptable	Intervention perceived as unacceptable

Table 1: Go/ Stop criteria for the definitive trial[3]

Secondary outcomes: Proposed outcomes for the definitive trial
All other individual-level, cluster-level and economic outcome measures proposed for the definitive trial will be assessed on the response, completion rates, acceptability and the adequacy of blinding for outcome assessors during data collection. Table 2 summarises outcome measures and data collection timing. See Supplementary Material-5 for collection tools.

[3] The study will have to meet the Definite Go criteria in order for the study to be feasible and to be able to proceed. However, if any one of the Definite Stop criteria is met then the study stops. The 'Go/Stop' indicates that it is either feasible or not feasible to proceed to a definitive trial. The progression rules have been approved by the steering and data management team. Should any of the progression rules not be met, assessments and adjustments of the NEON pilot feasibility RCT will be negotiated before proceeding to a definitive trial.

For peer review only - http://bmjopen.bmj.com/site/about/guidelines.xhtml

Outcome	Outcome measures	Data collection	Timing				
			Baseline	Every 2 wks (end of each PLA meeting)	After 14 wks (end of PLA cycle)	6 mths	12 mths
**Proposed primary individual-level outcome**	Individual Child BMI z-score	RA, CRs	✓		✓	✓	
**Proposed secondary individual-level outcome**	Children feeding behaviour[4]	RA, CRs	✓		✓	✓	
	Parental feeding style[4]	RA, CRs	✓		✓	✓	
	Audio/Video recording of child eating behaviours & parental feeding practices	RA	✓		✓		
	4-day food diary[4]	RA, CRs	✓		✓	✓	
	Network diffusion†[4]	RA, CRs		✓		✓	
**Proposed secondary cluster-level outcome**	Children's development performance	RA, HRs	✓			✓	
	Level of dental caries	RA	✓			✓	
	GP healthcare utilisation	RA	✓			✓	
**Economic outcome**	Cost tool	RA	✓			✓	
	Partner's time Questionnaire	RA					✓
**Process outcome†**	Participants Feedback[4]	RA, CRs		✓	✓		
	Facilitator Report[4]	RA, CRs		✓	✓		
	PLA cycle meeting register[4]	RA, CRs		✓	✓		
	Direct observation	RA, CRs		✓	✓		
	Sustainability assessment	RA, CRs		✓	✓		

Table 2. Schedule of Outcome Assessments

[4] Community Facilitators will assist in administering or completing these data collection tools before sending them to RA/CRs during the intervention period; †For intervention groups only; CR= Community Researchers; RA = Research Assistant

Primary individual-level outcome

1. *Child BMI z-score:* CRs will measure infants' length/height, head circumference and weight. The RA will refer to the WHO 2006 growth standard [2], using age- and gender-adjusted length or height (m)/weight $(kg)^2$ to provide a comparison on the cluster-level.

Secondary individual-level outcomes

1. *Children's feeding behaviour* will be measured using 6 of 8 domains adapted from the validated Children's Eating Behaviour Questionnaire (CEBQ) deemed suitable for infants: food responsiveness; enjoyment of food; emotional overeating; desire to drink; satiety responsiveness; slowness in eating; emotional undereating; and food fussiness [7, 8, 22]. Obesity risk could be linked with external eating, emotional overeating, and food fussiness [7].

2. *Parental feeding style* will be assessed with a validated self-reported Parental Feeding Style Questionnaire (PFSQ) with four scales: emotional feeding; instrumental feeding; encouragement to eat; and control overeating [23].

3. *Audio/video recording of child eating behaviour and parental feeding practices* [24] involves asking participants about the meal (e.g. name, ingredients) and creating annotations that link to behaviour codes identified from NEON formative study [25]. Before-and-after audio/video recordings will be thematically analysed using Elan Software [26] to measure intervention effect.

4. *Child food intake*: The 4-day food diary [7] is a self-reported, prospective, open-ended survey that collects detailed quantitative estimates of food consumption of infants between 4-18 months [6]. It assesses nutrient intake (e.g. total fat, total carbohydrate, salt, sugar) by comparing with age/sex-specific UK dietary reference values [27]. Dietary data will be presented by age group (e.g. 4-6 months, 7-9 months, 12-18 months).

5. *Network diffusion of study materials between participants and their communities*: CRs will track the number of downloads using the eRedbook platform. Participants will also complete a questionnaire on the number of people they shared the material with, their family size, age, gender, relationship to the participant and platform used.

6. *Equality impact assessment (EIA)*: This trial should reflect the diversity of the target population by enabling equal access while accommodating different needs. An EIA tool will systematically assess likely effects of the intervention on people (e.g. with respect to disability, gender, ethnicity, age, sexual orientation, religion/belief etc.), mitigate adverse impacts and identify active steps to address existing disadvantage and promote equality.

Secondary cluster-level routine outcomes

Although this intervention acts on the individual-level, we expect a social diffusion of good infant feeding and dental hygiene awareness and practices in the medium- to long-term (Supplementary Material-2, Logic Model). While immediate cluster-level changes are not expected, we would like to pilot cluster-level routine data collection.

For peer review only

1. *Development performance of children*: The Ages and Stages questionnaire (ASQ-3) include communication, gross motor, fine motor, problem-solving, and personal-social domains [5]. Three versions are available: 24-months, 27-months and 30-months. ASQ-3 data collected by HVs during mandated visits are stored in EMIS/ RIO databases.

2. *GP healthcare utilisation* data is available in participants' medical record.

3. *Level of dental caries* and other signs of tooth decay is available in NHS dental services records for registered patients beginning at 6-months of age and approximately every half-year following initial visit [17,28,29].

Economic outcomes

1. *Cost*: The RA will use a spreadsheet will capture the cost of the NEON Intervention Development and pilot feasibility RCT phases every 6 months.

2. *Partners' time used*: A questionnaire will capture time spent for the delivery of NEON-associated programmes.

Data Collection and Management

Outcome data will be collected at baseline, every 2-weeks during intervention (for process measures), 14-weeks immediately post-intervention, 6-months and 12-months follow-up (for economic data). During the intervention period, several outcome measurements will be administered by CFs to participants or completed by CFs and sent to CRs/RA. Other outcomes and post-intervention outcomes will be assessed directly by CRs/RA (Table 2). Each case will be assigned an ID. Data collected digitally or in paper format will be stored in UCL S: Drive and/or UCL cabinets. We will use RedCap electronic data management system [30]; the RedCap API allows flexible and straightforward data import and export to data analysis software.

We will form a data sharing agreement with study partners for the RA to extract and link routine cluster-level data to trial participants. To guarantee confidentiality, pseudonymised participant data will be stored on encrypted, password-protected computer or UCL S:Drive accessible only to study staff and authorised personnel. Password-protected personal data will be stored separately from trial data on secure UCL computers. Only pseudonymised quotes or data from audio/video recording may be published. Personal data and audio/video recordings will be stored for 3-years post-study and destroyed; research data will be stored for 20-years post-study.

Analysis

Overall, quantitative and qualitative data will be analysed concurrently at multiple time points to identify early problems that are rectified as the trial progresses. Quantitative data will be descriptively summarised using mean and standard deviation (SD) for continuous variables, number with percentages for categorical variables, and 95% CI with a breakdown of participants by trial arm, ethnic/language group and borough where relevant. Results will be collectively assessed against the

For peer review only

Go/Stop criteria. Before progression to the definitive trial, we will systematically and rigorously assess areas of improvement using a structured discussion tool: A process for Decision-making after Pilot and feasibility Trials (ADePT) [31]. This three-step process involves deciding the type of problem, identifying all solutions, and systematically appraising these solutions.

Sample Characteristics

Participant demographic characteristics will be descriptively summarised by trial arm. We will check for recruitment bias across different arms at baseline and compare later recruits who are prone to unblinded recruitment.

Feasibility and Process Measures

Across the three arms, we will check for any differential recruitment or retention rates and any consequent systematic differences in participant characteristics over time, as control arm participants may be less keen to participate or remain in the study.

For the two intervention arms, we will conduct a mixed-methods implementation evaluation to explore the possibility of a blended approach in the definitive trial as some features of each delivery mode might be particularly salient. Qualitative data will be analysed thematically using framework analysis that include participants' motivation for engagement, expectations, experiences, intervention acceptability, implementation barriers, and suggestions for improvement. Negative 'deviant' cases will inform interpretation.

Outcome measures for the definitive trial

1. *Feasibility of collecting outcome measures:* To assess completeness, degrees of missingness at the individual item level and the entire outcome measure will be reported overall, by time-point and trial arms. Other feasibility measures would be captured by analyses of process measures above.

2. *Intervention effect for proposed primary outcome (BMI z-score):* Conditioned upon low recruitment bias and sufficient adherence to trial protocol, we will estimate the difference in BMI z-score between each intervention arm versus the control arm. We will consider methods to pool data across age groups depending on the actual sample age breakdown.

3. *Sample size and power calculation for the definitive trial:* Data from the pilot feasibility trial will enable us to determine (i) the smallest difference of clinical importance (through multiple testing of several key endpoints for the proposed primary outcome), (ii) the clinically justifiable power-significance level or scientifically acceptable probability of "false positive" (type I error) results, and (iii) whether we need to adjust the calculated sample size for expected level of non-compliance/ drop-in and drop-outs. We will report the ICC.

4. *Economic Evaluation:* This will be conducted from a provider and user perspective which includes direct cost (e.g. time, resources spent on training and participation of CF, participants' overall household consumption and childcare costs). We will estimate the total cost, average annual cost, cost-effectiveness, equity impact analysis and compare across three arms.

For peer review only

Monitoring

The Principal Investigator (PI) will be responsible for day-to-day monitoring and study management.

The NEON Steering and Data Management Team will ensure adherence to all relevant regulations and principles of good clinical practice. The team consists of multidisciplinary experts in child health, practitioners, PLA expert, SA independent observers from NEON 1, and Queen Mary University of London Pragmatic Clinical Trials Unit. Committee meetings will be held every 3 months to review the Go/Stop criteria (Table 1) and interim analyses. The team will agree to the trial protocol and facilitate any necessary protocol amendments; provide independent expert advice (including on project management governance); monitor progress; ensure adequate deadlines are set and met; monitor and advise NEON Core Team (Trial Management Group) on strategic decisions in light of new evidence; and ensure successful delivery by meeting and reporting on study progress with the Core Team biannually. The PI and other Core Team members will attend all necessary meetings and report on the study progress.

Any adverse events or safety concerns will be captured in the Participant Feedback Questionnaire and Facilitator Report Form (every 2-weeks during intervention period) and reported to the steering and safety monitoring team.

The UCLH/UCL Joint Research Office, on behalf of the Sponsor (UCL), may conduct random audits in accordance with the UK Policy Framework for Health and Social Care Research and UCL's policies and procedures.

ETHICS AND DISSEMINATION

Ethical approval will be obtained from UCL Research Ethics Committee and NHS Health Research Authority. The participant information sheet will be provided in English or participants' native language in-writing and/or verbally by community facilitators for informed consent. The RA or CRs will obtain written or audio-recorded consent ≥24 hours later [32]. Participation will be voluntary and they may withdraw anytime without prejudicing treatment or usual care. No incentives will be provided except for small reimbursements (e.g. childcare at the local centre where PLA meetings take place).

UCL will provide insurance cover and we will also signpost any vulnerable participants who require protection from harm to relevant safeguarding organisations.

Regular progress updates will be shared via UCL or NIHR websites and patient group newsletters. Study findings will be submitted for 3-5 publications in high impact factor peer-reviewed journals and presented at national and international conferences. Researchers and community members from the steering team will contribute to confirming analysis and write-up, adhering to authorship guidelines. To reach wider audiences, we will (i) disseminate plain language summaries for each academic paper through community groups, social media and You Tube; (ii) present findings at lay-person meetings at community or children centres through the NIHR CLAHRC network and (iii) organise 2-3 annual workshops with commissioners, partner organisations and NEON community facilitators to share key findings and recommendations.

For peer review only

Acknowledgements

The authors would like to thank the South Asian community facilitators in the NEON Intervention Development phase: Lily Islam, Seema Bajwa, Aasma Baiyat, Jasvir Bhachu, Geromini Pushpakanthan , Sabiha Malek, Cllr Lakmini Shah; and community members of the London Boroughs of Tower Hamlet, Newham, and Waltham Forest for their important contribution and engagement to this research project. Furthermore, the authors would like to thank the 'Conversation Café' initiative led by Cllr Lakmini Shah for her significant help in the recruitment of the community facilitators and members, and in identifying the community centres to carry out the intervention development meetings. The authors would also like to acknowledge the contribution of the NEON core team, steering team and all health experts in validating intervention NEON toolkit. We would like to thank the Women & Children First Charity and First Steps Nutrition Trust for their valuable contributions and guidance throughout the study.

Author's Contributions

ML, LM, MH, NB, CL, AH, SA conceived the original concept of the study and designed the research methodology. SA carried out the Intervention Development meetings, workshops, co-development of the intervention toolkit, wrote and devised the paper. LM, ML, SA, NB, CL, AH, RL, JG, KWM, CI, MA, CE, CC, MM, DD and AN validated the study and revised the manuscript critically for important intellectual content. ICD was involved in drafting materials for the NEON meetings and workshops of the Intervention Development Phase. UB and ICD contributed to the manuscript writing, and prepared it for submission. LM, SA, ML had primary responsibility for the final content. All authors read and contributed to reviewing the study data, the designing of the manuscript, and the approval of the final manuscript.

Funding

Logan Manikam and Shereen Al Laham are funded via a National Institute for Health Research (NIHR) Advanced Fellowship (Ref: NIHR300020) to undertake the Pilot Feasibility Cluster Randomised Controlled Trial of the *NEON* programme in East London. Prof Monica Lakhanpaul was funded by the NIHR Collaboration for Leadership in Applied Health Research and Care (CLAHRC) North Thames.

Disclaimer

The views expressed in the publication are those of the author(s) and not necessarily those of the sponsor (UCL), funder (NIHR), study partners (Tower Hamlets GP Care Group, London Borough of Newham Council, Waltham Forest Council).

Conflicts of Interest

The authors declared no potential conflicts of interest with respect to the research, authorship and/or publication of this paper.

For peer review only

Participant consent for publication

Participant information sheets and consent forms have been provided to community members and those expressing interest. Community participants have agreed to participate and given audio/video consent prior to their participation in the workshops. Verbal consent was witnessed and formally recorded. All participants have been informed of their right to freely withdraw from the study at any time. Confidentiality of personal data has been ensured through the use of anonymisation techniques as stated in the Data Protection Act (1998) and in line with the General Data Protection Regulation (2018). All participant data has been anonymised and stored on an encrypted password protected computer. Data can only be accessed by the authorised research personnel.

Ethics approval

This study has obtained ethical approval from UCL Research Ethics Committee [Ethics ID 17269/001], Sponsor reference number: 142600, Funding Reference: NIHR300020 and IRAS number: 296259, Ref: 21/SW/0142. Study protocols and relevant documents will be reviewed by UCL Research Ethics Committee, NHS Health Research Authority (HRA) and study partners involved in establishing data sharing agreement for linking participants' routine data (Tower Hamlets GP Care Group, London Borough of Newham Council, Waltham Forest Council).

Provenance and Peer Review

Not commissioned; peer reviewed for ethical and funding approval prior to submission.

Data Sharing Statement

The data supporting the findings of this study is available upon reasonable request from the corresponding author.

For peer review only

REFERENCES

[1] World Health Organization (WHO), *Postnatal care for mothers and newborns: Highlights from the World Health Organization 2013 Guidelines.* Available from: http://www. who. int/maternal_child_adolescent/publications/WHOMCA-PNC-2014-Briefer_ A, 2015. **4**.

[2] Group, W.M.G.R.S., *WHO Child Growth Standards based on length/height, weight and age.* Acta paediatrica (Oslo, Norway: 1992). Supplement, 2006. **450**: p. 76.

[3] Evandrou M, Falkingham J, Feng Z*, et al* Ethnic inequalities in limiting health and self-reported health in later life revisited *J Epidemiol Community Health* 2016;**70:**653-662.

[4] Brown MS. Religion and economic activity in the South Asian population. Ethnic and Racial Studies. 2000;23(6):1035-61

[5] ASQ-3. *The Ages & Stages Questionnaires, Third Edition*. [cited 2019 30 November]; Available from: https://agesandstages.com/products-pricing/asq3/.

[6] Lennox, A., et al., *Diet and nutrition survey of infants and young children, 2011.* A survey carried out on behalf of the Department of Health and Food Standards Agency, 2013.

[7] *NATIONAL DIET AND NUTRITION SURVEY: Food and Drink Diary*. 2010; Available from: https://webarchive.nationalarchives.gov.uk/20130413115649/http://media.dh.gov.uk/network/261/files/2012/07/Appendix-E_All-docs-combined-v2.pdf.

[8] Wardle, J., et al., *Parental feeding style and the inter-generational transmission of obesity risk.* Obesity research, 2002. **10**(6): p. 453-462.

[9] 2011. *Early Years Interventions To Address Health Inequalities In London – The Economic Case*. [ebook] London: Greater London Authority. Available at: <https://www.london.gov.uk/sites/default/files/gla_migrate_files_destination/Early%20Years%20report%20OPT.pdf> [Accessed 5 January 2021].

[10] Lakhanpaul, M., 2019. *PANCHSHEEEL - MRC HEEE STUDY*. [online] Ucl.ac.uk. Available at: <https://www.ucl.ac.uk/child-health/sites/child-health/files/newsletter_3_0.pdf

[11] WHO. WHO recommendation on facilitated participatory learning and action cycles with women`s groups during pregnancy: The WHO Reproductive Health Library 2018 [Available from: https://extranet.who.int/rhl/topics/improving-health-system-performance/implementation-strategies/who-recommendation-facilitated-participatory-learning-and-action-cycles-womens-groups-during.

[12] Seward N, Neuman M, Colbourn T, Osrin D, Lewycka S, Azad K, et al. Effects of women's groups practising participatory learning and action on preventive and care-seeking behaviours to reduce neonatal mortality: A meta-analysis of cluster-randomised trials. PLoS medicine. 2017;14(12):e1002467.

[13] Tripathy P, Nair N, Barnett S, Mahapatra R, Borghi J, Rath S, et al. Effect of a participatory intervention with women's groups on birth outcomes and maternal depression in Jharkhand and Orissa, India: a cluster-randomised controlled trial. The Lancet. 2010;375(9721):1182-92

For peer review only - http://bmjopen.bmj.com/site/about/guidelines.xhtml

[14] Prost A, Colbourn T, Seward N, Azad K, Coomarasamy A, Copas A, et al. Women's groups practising participatory learning and action to improve maternal and newborn health in low-resource settings: a systematic review and meta-analysis. The Lancet. 2013;381(9879):1736-46

[15] Mayor of London. London's socio-economic issues. 2016; Available at: https://www.london.gov.uk/sites/default/files/chapter10-economic-evidence-base-2016.pdf. Accessed 08/06/, 2020.

[16] ONS. 2011 Census Data. 2011; Available at: https://www.ons.gov.uk/census/2011census.

[17] Faculty of Dental Surgery. 2015. The state of children's oral health in England. [PDF]

[18] Newington L, Metcalfe A. Factors influencing recruitment to research: qualitative study of the experiences and perceptions of research teams. *BMC Med Res Methodol*. 2014;14:10. Published 2014 Jan 23. doi:10.1186/1471-2288-14-10

[19] Office for National Statistics. 2020. *Domestic Abuse During The Coronavirus (COVID-19) Pandemic, England And Wales - Office For National Statistics*. [online] Available at: <https://www.ons.gov.uk/peoplepopulationandcommunity/crimeandjustice/articles/domesticabuseduringthecoronaviruscovid19pandemicenglandandwales/november2020> [Accessed 10 January 2021].

[20] Joseph RP, Keller C, Ainsworth BE. Recruiting Participants into Pilot Trials: Techniques for Researchers with Shoestring Budgets. *Calif J Health Promot*. 2016;14(2):81-89.

[21] An innovative approach to recruiting homebound older adults. *Crawford Shearer NB, Fleury JD, Belyea MRes Gerontol Nurs. 2010 Jan; 3(1):11-8.*

[22] Llewellyn, C.H., et al., *Development and factor structure of the Baby Eating Behaviour Questionnaire in the Gemini birth cohort.* Appetite, 2011. **57**(2): p. 388-396.

[23] Daniels, L.A., et al., *Outcomes of an early feeding practices intervention to prevent childhood obesity.* Pediatrics, 2013. **132**(1): p. e109-e118.

[24] Fogel, A., et al., *Prospective associations between parental feeding practices and children's oral processing behaviours.* Maternal & child nutrition, 2019. **15**(1): p. e12635.

[25] Lakhanpaul M, Benton L, Lloyd-Houldey O, et al. *Nurture Early for Optimal Nutrition (NEON) programme: qualitative study of drivers of infant feeding and care practices in a British-Bangladeshi population*. BMJ Open 2020;**10**:e035347. doi:10.1136/ bmjopen-2019-035347

[26] Tla.mpi.nl. 2020. *ELAN | The Language Archive*. [online] Available at: http://tla.mpi.nl/tools/tla-tools/elan

[27] Health Do. *Infant feeding recommendation*. Department of Health London, England; 2003.

[28] 2019. Dental Record Keeping Standards: a consensus approach. [PDF] NHS England and NHS Improvement.

[29] Faculty of General Dental Practice, 2012. 7. Collecting Data. Available at: <https://www.fgdp.org.uk/research-primary-dental-care/7-collecting-data> [Accessed April 15, 2021].

[30] Lawrence, C.E. et al. A REDCap-based model for electronic consent (eConsent): Moving toward a more personalized consent. Journal of Clinical and Translational Science. 2020; 4(4):345–353.

[31] Bugge C, et al. A process for Decision-making after Pilot and feasibility Trials (ADePT): Development following a feasibility study of a complex intervention for pelvic organ prolapse. Trials. 2013 Oct 25;14(1):353.

[32] Health Research Authority. 2018. *HRA And MHRA Publish Joint Statement On Seeking And Documenting Consent Using Electronic Methods (Econsent)*. [online] Available at: <https://s3.eu-west-2.amazonaws.com/www.hra.nhs.uk/media/documents/hra-mhra-econsent-statement-sept-18.pdf> [Accessed 8 January 2021]

For peer review only

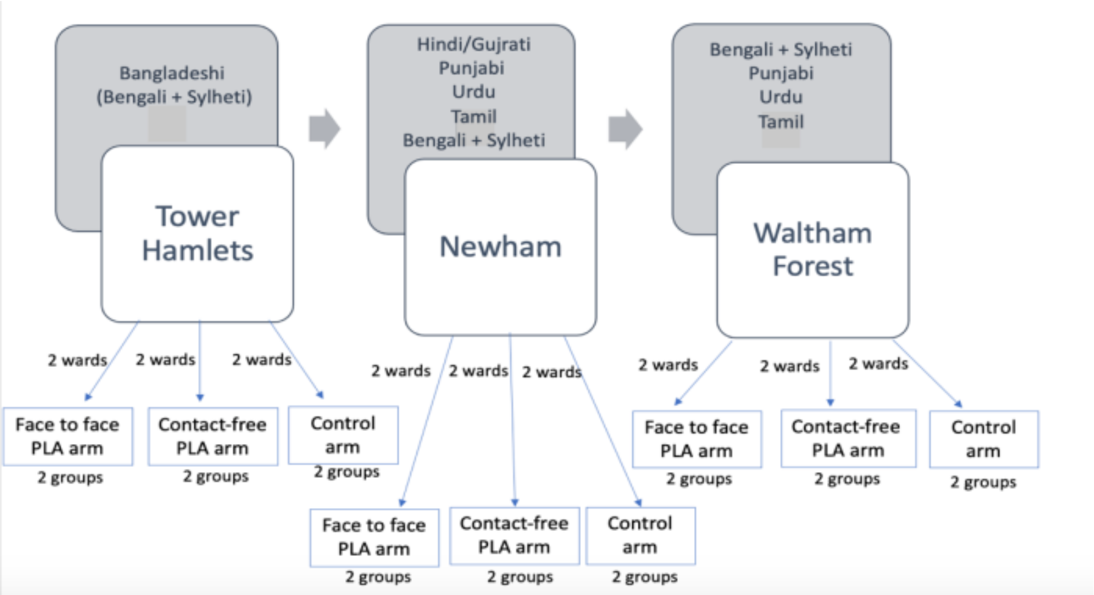

Figure 1. Overview schematic detailing number of wards & PLA groups by East London boroughs

Footnote: To illustrate, TH will have 2 face-to-face Bangladeshi/ Bengali & Sylheti PLA groups (1 per ward), 2 groups of Bangladeshi/ Bengali & Sylheti online PLA group (1 per ward), and 2 Bangladeshi/ Bengali & Sylheti control groups (1 per ward). The more the ethnic/ language group in the borough, the more the intervention and control groups.

For peer review only

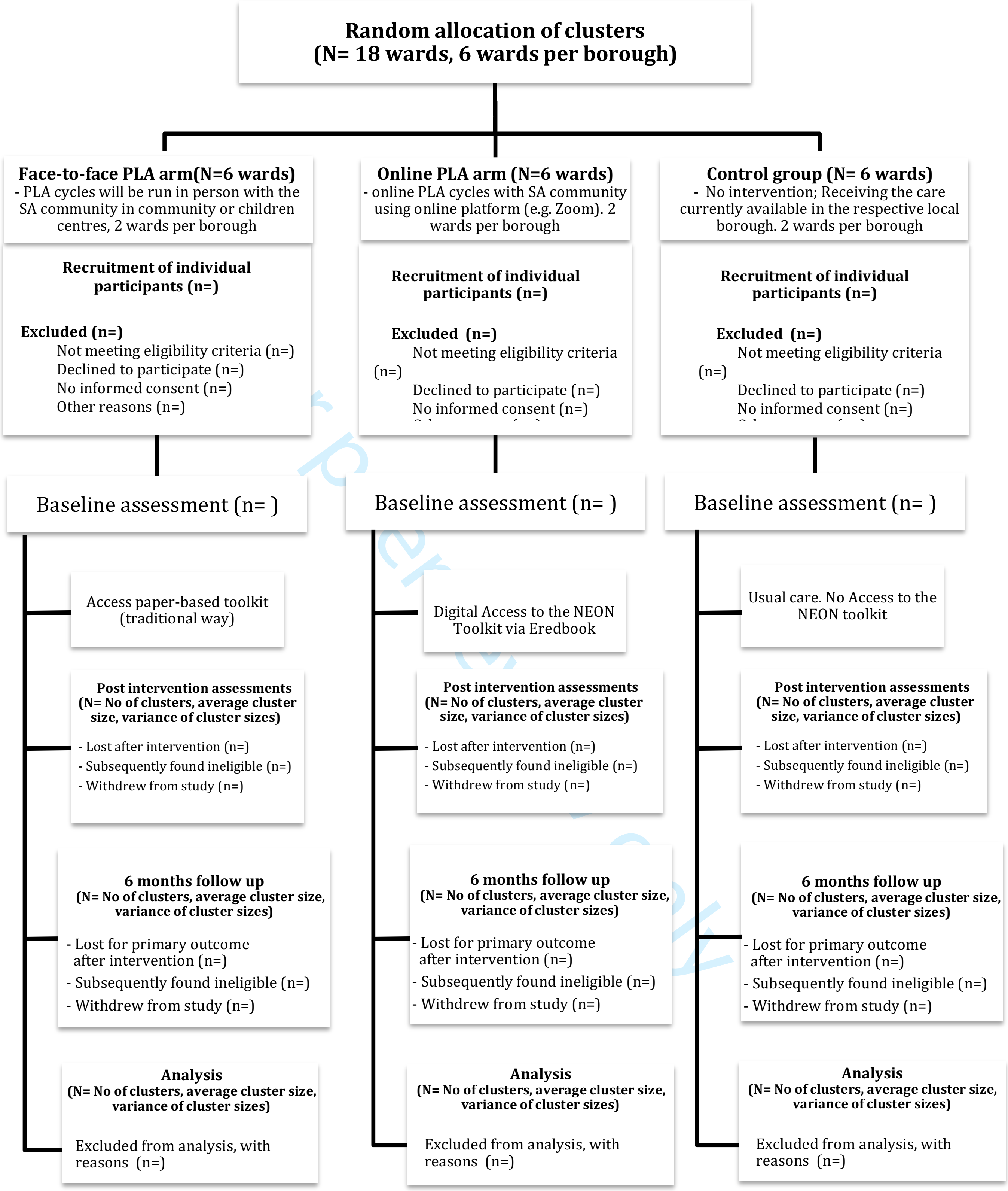

Figure 2. Overview of the NEON pilot feasibility RCT design and participant flow

For peer review only - http://bmjopen.bmj.com/site/about/guidelines.xhtml

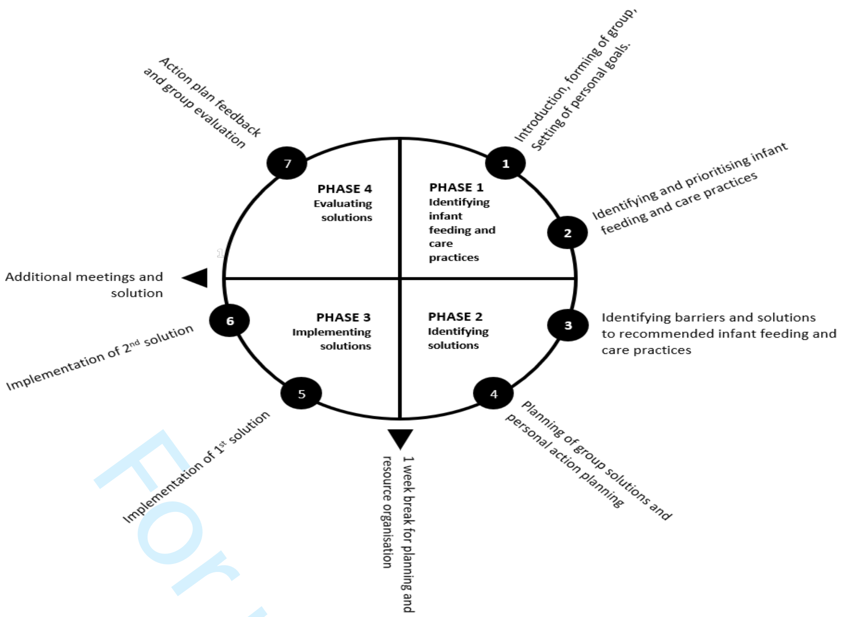

Figure 3. NEON Women's Group PLA Cycle

For peer review only

SPIRIT 2013 Checklist: Recommended items to address in a clinical trial protocol and related documents*

Section/ item	Item No	Description	Page
**Administrative information**			
Title	1	Descriptive title identifying the study design, population, interventions, and, if applicable, trial acronym	1
Trial registration	2a	Trial identifier and registry name. If not yet registered, name of intended registry	3, 17, 18
	2b	All items from the World Health Organization Trial Registration Data Set	N/A
Protocol version	3	Date and version identifier	17, 18
Funding	4	Sources and types of financial, material, and other support	17
Roles and responsibilities	5a	Names, affiliations, and roles of protocol contributors	1, 17
	5b	Name and contact information for the trial sponsor	17
	5c	Role of study sponsor and funders, if any, in study design; collection, management, analysis, and interpretation of data; writing of the report; and the decision to submit the report for publication, including whether they will have ultimate authority over any of these activities	17, 18
	5d	Composition, roles, and responsibilities of the coordinating centre, steering committee, endpoint adjudication committee, data management team, and other individuals or groups overseeing the trial, if applicable (see Item 21a for data monitoring committee)	11, 16
**Introduction**			
Background and rationale	6a	Description of research question and justification for undertaking the trial, including summary of relevant studies (published and unpublished) examining benefits and harms for each intervention	3, 5
	6b	Explanation for choice of comparators	6, 7
Objectives	7	Specific objectives or hypotheses	6
Trial design	8	Description of trial design including type of trial (eg, parallel group, crossover, factorial, single group), allocation ratio, and framework (eg, superiority, equivalence, noninferiority, exploratory)	6, 7

For peer review only - http://bmjopen.bmj.com/site/about/guidelines.xhtml

Methods: Participants, interventions, and outcomes

Study setting	9	Description of study settings (eg, community clinic, academic hospital) and list of countries where data will be collected. Reference to where list of study sites can be obtained	7
Eligibility criteria	10	Inclusion and exclusion criteria for participants. If applicable, eligibility criteria for study centres and individuals who will perform the interventions (eg, surgeons, psychotherapists)	7, 8
Interventions	11a	Interventions for each group with sufficient detail to allow replication, including how and when they will be administered	6, 7, 10
	11b	Criteria for discontinuing or modifying allocated interventions for a given trial participant (eg, drug dose change in response to harms, participant request, or improving/worsening disease)	N/A
	11c	Strategies to improve adherence to intervention protocols, and any procedures for monitoring adherence (eg, drug tablet return, laboratory tests)	N/A
	11d	Relevant concomitant care and interventions that are permitted or prohibited during the trial	N/A
Outcomes	12	Primary, secondary, and other outcomes, including the specific measurement variable (eg, systolic blood pressure), analysis metric (eg, change from baseline, final value, time to event), method of aggregation (eg, median, proportion), and time point for each outcome. Explanation of the clinical relevance of chosen efficacy and harm outcomes is strongly recommended	11, 12
Participant timeline	13	Time schedule of enrolment, interventions (including any run-ins and washouts), assessments, and visits for participants. A schematic diagram is highly recommended (see Figure)	9
Sample size	14	Estimated number of participants needed to achieve study objectives and how it was determined, including clinical and statistical assumptions supporting any sample size calculations	8
Recruitment	15	Strategies for achieving adequate participant enrolment to reach target sample size	8, 9

Methods: Assignment of interventions (for controlled trials)

Allocation:

Sequence generation	16a	Method of generating the allocation sequence (eg, computer-generated random numbers), and list of any factors for stratification. To reduce	9, 10

		predictability of a random sequence, details of any planned restriction (eg, blocking) should be provided in a separate document that is unavailable to those who enrol participants or assign interventions	
Allocation concealment mechanism	16b	Mechanism of implementing the allocation sequence (eg, central telephone; sequentially numbered, opaque, sealed envelopes), describing any steps to conceal the sequence until interventions are assigned	9, 10
Implementation	16c	Who will generate the allocation sequence, who will enrol participants, and who will assign participants to interventions	9, 10
Blinding (masking)	17a	Who will be blinded after assignment to interventions (eg, trial participants, care providers, outcome assessors, data analysts), and how	9, 10
	17b	If blinded, circumstances under which unblinding is permissible, and procedure for revealing a participant's allocated intervention during the trial	9, 10
**Methods: Data collection, management, and analysis**			
Data collection methods	18a	Plans for assessment and collection of outcome, baseline, and other trial data, including any related processes to promote data quality (eg, duplicate measurements, training of assessors) and a description of study instruments (eg, questionnaires, laboratory tests) along with their reliability and validity, if known. Reference to where data collection forms can be found, if not in the protocol	11, 12, 13
	18b	Plans to promote participant retention and complete follow-up, including list of any outcome data to be collected for participants who discontinue or deviate from intervention protocols	14, 16
Data management	19	Plans for data entry, coding, security, and storage, including any related processes to promote data quality (eg, double data entry; range checks for data values). Reference to where details of data management procedures can be found, if not in the protocol	14
Statistical methods	20a	Statistical methods for analysing primary and secondary outcomes. Reference to where other details of the statistical analysis plan can be found, if not in the protocol	14, 15
	20b	Methods for any additional analyses (eg, subgroup and adjusted analyses)	N/A
	20c	Definition of analysis population relating to protocol non-adherence (eg, as randomised analysis), and any statistical methods to handle missing data (eg, multiple imputation)	N/A
**Methods: Monitoring**			
Data monitoring	21a	Composition of data monitoring committee (DMC); summary of its role and reporting structure; statement of whether it is independent from the sponsor and competing interests; and reference to where further details	16

For peer review only - http://bmjopen.bmj.com/site/about/guidelines.xhtml

		about its charter can be found, if not in the protocol. Alternatively, an explanation of why a DMC is not needed	
	21b	Description of any interim analyses and stopping guidelines, including who will have access to these interim results and make the final decision to terminate the trial	16
/Harms	22	Plans for collecting, assessing, reporting, and managing solicited and spontaneously reported adverse events and other unintended effects of trial interventions or trial conduct	N/A
Auditing	23	Frequency and procedures for auditing trial conduct, if any, and whether the process will be independent from investigators and the sponsor	N/A
**Ethics and dissemination**			
Research ethics approval	24	Plans for seeking research ethics committee/institutional review board (REC/IRB) approval	2, 16, 18
Protocol amendments	25	Plans for communicating important protocol modifications (eg, changes to eligibility criteria, outcomes, analyses) to relevant parties (eg, investigators, REC/IRBs, trial participants, trial registries, journals, regulators)	16, 17
Consent or assent	26a	Who will obtain informed consent or assent from potential trial participants or authorised surrogates, and how (see Item 32)	18
	26b	Additional consent provisions for collection and use of participant data and biological specimens in ancillary studies, if applicable	N/A
Confidentiality	27	How personal information about potential and enrolled participants will be collected, shared, and maintained in order to protect confidentiality before, during, and after the trial	17, 18
Declaration of interests	28	Financial and other competing interests for principal investigators for the overall trial and each study site	17, 18
Access to data	29	Statement of who will have access to the final trial dataset, and disclosure of contractual agreements that limit such access for investigators	18
Ancillary and post-trial care	30	Provisions, if any, for ancillary and post-trial care, and for compensation to those who suffer harm from trial participation	N/A
Dissemination policy	31a	Plans for investigators and sponsor to communicate trial results to participants, healthcare professionals, the public, and other relevant groups (eg, via publication, reporting in results databases, or other data sharing arrangements), including any publication restrictions	18
	31b	Authorship eligibility guidelines and any intended use of professional writers	N/A

For peer review only - http://bmjopen.bmj.com/site/about/guidelines.xhtml

	31c	Plans, if any, for granting public access to the full protocol, participant-level dataset, and statistical code	18
**Appendices**			
Informed consent materials	32	Model consent form and other related documentation given to participants and authorised surrogates	Suppl. material
Biological specimens	33	Plans for collection, laboratory evaluation, and storage of biological specimens for genetic or molecular analysis in the current trial and for future use in ancillary studies, if applicable	N/A

*It is strongly recommended that this checklist be read in conjunction with the SPIRIT 2013 Explanation & Elaboration for important clarification on the items. Amendments to the protocol should be tracked and dated.
The SPIRIT checklist is copyrighted by the SPIRIT Group under the Creative Commons "Attribution-NonCommercial-NoDerivs 3.0 Unported" license.

For peer review only

BMJ Open

Nurture Early for Optimal Nutrition (NEON) Pilot Feasibility Randomised Controlled Trial Study Protocol Community facilitator led participatory learning and action (PLA) women's groups to improve infant feeding, care and dental hygiene practices in South Asian infants aged < 2 years in East London

Journal:	*BMJ Open*
Manuscript ID	bmjopen-2022-063885.R1
Article Type:	Protocol
Date Submitted by the Author:	27-Jan-2023

Complete List of Authors:	Manikam, Logan; UCL Institute of Epidemiology and Health Care, Epidemiology and Public Health; Aceso Global Health Consultants<br>Patil, Priyanka; University College London Institute of Epidemiology and Health Care; Aceso Global Health Consultants Pte Limited<br>Naman, Maryan; Aceso Global Health Consultants Pte Limited<br>Allaham, Shereen ; UCL Institute of Epidemiology and Health Care, Department of Epidemiology and Public Health; Aceso Global Health Consultants Ltd,<br>Ong, Zhen Ling; Aceso Global Health Consultants Limited<br>Demel, Isabel-Cathérine; King's College London Faculty of Life Sciences and Medicine, Guy's, King's and St.Thomas' School of Medicine<br>Batura, Neha; University College London, Institute for Global Health<br>Llewellyn, Clare; University College London Department of Behavioural Science and Health<br>Hayward, Andrew; University College London, Epidemiology and Public Health<br>Lakshman, Rajalakshmi; Cambridgeshire County Council, Public Health Directorate; University of Cambridge, Medical Research Council Epidemiology Unit<br>Gilmour, Jenny; Mile End Hospital, Tower Hamlets GP Care Group<br>Webb-Martin, Kelley; London Borough of Newham, Children's Health 0-19 Service<br>Irish, Carol; London Borough of Newham, Children's Health 0-19 Service<br>Archibong, Mfon; London Borough of Newham, Children's Health 0-19 Service<br>Clarkson, Corinne; London Borough of Waltham Forest, Council, Public Health Strategist for Maternal & Child Health<br>Delceta, Daley; Chingford Medical Practice, Operational Lead<br>Islam, Lily; London Borough of Tower Hamlets, Community Facilitator<br>Bajwa, Seema; London Borough of Newham, Community Facilitator<br>Malek, Sabiha; London Borough of Newham, Community Facilitator<br>Bhachu, Jasvir; London Borough of Newham, Community Facilitator<br>Pushpakanthan, Geromini; London Borough of Newham, Community Facilitator<br>Heys, Michelle; University College London Institute of Child Health,

	University College London Institute of Child Health; Specialist Children and Young People’s Services, East London NHS Foundation Trust Lakhanpaul, Monica; UCL Great Ormond Street Institute of Child Health Population Policy and Practice, Institute of Child Health; Whittington Health NHS Trust
**Primary Subject Heading**:	Public health
Secondary Subject Heading:	Epidemiology, Evidence based practice, Global health, Nutrition and metabolism, Public health
Keywords:	Community child health < PAEDIATRICS, NUTRITION & DIETETICS, PAEDIATRICS, PUBLIC HEALTH

SCHOLARONE™
Manuscripts

Nurture Early for Optimal Nutrition (NEON) Pilot Feasibility Randomised Controlled Trial Study Protocol Community facilitator led participatory learning and action (PLA) women's groups to improve infant feeding, care and dental hygiene practices in South Asian infants aged < 2 years in East London

Short Title/ Acronym: Nurture Early for Optimal Nutrition (NEON) Pilot Feasibility Randomised Controlled Trial

Authors:
[1]Dr Logan Manikam, NIHR Advanced Fellow & Honorary Consultant in Public Health Medicine[1,2]
Priyanka Patil, Research Assistant & Consultant[1,2]
Maryan Naman, Consultant[2]
Shereen Allaham, Research Assistant & Consultant[1,2]
Zhen Ling Ong, Associate[2]
Isabel-Cathérine Demel, Medical Student[2,3]
Dr Neha Batura, Associate Professor in Health Economics[6]
Dr Clare Llewellyn, Associate Professor in Behavioural Obesity Research[7]
Prof Andrew Hayward, Professor of Infectious Disease Epidemiology and Inclusion Health Research[1]
Dr Rajalakshmi Lakshman, Consultant in Public Health Medicine[8]
Jenny Gilmour, 0-19 Service Development Lead THGPCG[9]
Kelley Webb Martin, Director of Delivery, Compliance & Transformation[10]
Carol Irish, Head of Children's Health 0-19 Service[10]
Mfon Archibong, Specialist Health Visiting Research Lead[10]
Corinne Clarkson, Public Health Strategist for Maternal & Child Health[11]
Daley Delceta, Operational Lead[12]
Lily Islam, Tower Hamlets community facilitator
Seema Bajwa, Newham community facilitator
Sabiha Malek, Newham community facilitator
Jasvir Bhachu, Newham community facilitator
Geromini Pushpakanthan, Newham community facilitator
[2]Dr Michelle Heys, Associate Professor of Community and Population Child Health, Consultant Paediatrician[4,5]
Monica Lakhanpaul Professor of Integrated Community Child Health[13,14]

---

[1] Logan Manikam and Priyanka Patil are joint first authors

[2] Dr Michelle Heys and Professor Monica Lakhanpaul are joint senior authors

For peer review only

Affiliations:

1. Department of Epidemiology and Public Health, University College London Institute of Epidemiology and Health Care, London, UK
2. Aceso Global Health Consultants Pte Limited, Singapore
3. Department of Life Sciences & Medicine, King's College London GKT School of Medical Education, London, UK
4. Specialist Children and Young People's Services, East London NHS Foundation Trust, 84 West Ham Lane, London E15 4PT.
5. Population, Policy and Practice, UCL Great Ormond Street Institute of Child Health, 30 Guildford Street, London WC1N 1EH, UK
6. Institute for Global Health, University College London, 30 Guilford Street, London WC1N 1EH.
7. Department of Behavioural Sciences & Health, UCL IEHC, 1-19 Torrington Pl, Fitzrovia, London WC1E 7HB.
8. MRC Epidemiology Unit, University of Cambridge
9. Tower Hamlets GP Care Group, Mile End Hospital, London, UK
10. Children's Health 0-19 Service, London Borough of Newham, 1000 Dockside Road E16 2QU
11. Waltham Forest Council, London, UK
12. Chingford Health Centre Waltham Forest, London, UK
13. Population, Policy and Practice, UCL Great Ormond Street Institute of Child Health, University College London, London, WC1N 1EH, UK
14. Whittington Health NHS Trust, London, UK, N19 5NF

Corresponding author: Dr Logan Manikam

Email: logan.manikam.10@ucl.ac.uk
Telephone: +44 7868721152

Key Words: Community-based intervention; infant; child health; nutrition; feeding practices; clinical trial

Word Count: 3999

For peer review only

ABSTRACT

Introduction: Feeding practices developed in early life can impact a child's nutrition, growth, dental health, cognitive development and lifetime risk of chronic diseases. Substantial evidence suggests ethnic health inequalities, and non-recommended complementary infant feeding practices among UK's South Asian (SA) population. Nurture Early for Optimal Nutrition (NEON) aims to use Women's group Participatory Learning and Action (PLA) cycles to optimise infant feeding, care and dental hygiene practices in SA infants <2 years in East London.

Methods and analysis: A three-arm pilot feasibility cluster randomised controlled trial will assess feasibility, acceptability, costs and explore preliminary effectiveness for proposed primary outcome (i.e. reporting on BMI z-score). Multilingual SA community facilitators (CFs) will deliver the intervention, Group PLA Cycle, to mothers/carers in respective ethnic/language groups. 12 wards are randomised to face-to-face PLA, online PLA and usual care arms in 1:1:1 ratio. Primary outcomes are feasibility and process measures (i.e. BMI z-score, study records, feedback questionnaires, direct observation of intervention, and sustainability) for assessment against Go/Stop criteria. Secondary outcomes are cluster-level and economic outcomes (i.e. eating behaviour, parental feeding practices, network diffusion, children development performance, level of dental caries, GP utilisation, costs, staff time). Outcomes are measured at baseline, every two weeks during intervention, 14-weeks and at 6-months by blinded outcome assessors where possible. This study will use concurrent mixed-methods evaluation. Quantitative analyses include descriptive summary with 95% confidence interval (CI) and sample size calculation for the definitive trial. The intervention effect with CI will be estimated for child BMI z-score. Implementation will be evaluated qualitatively using thematic framework analysis.

Ethics and dissemination: Ethics approval was obtained from UCL, NHS (HRA and HRCW). Results will be published in peer-reviewed journals, presented at scientific conferences/workshops with commissioners, partners and participating communities. Plain language summaries will be disseminated through community groups, websites and social media.

Word Count: 299/300

Trial registration: This trial will be registered with the International Standard Randomised Controlled Trial Number Register:IRAS-ID - 296259

For peer review only

ARTICLE SUMMARY

Strengths

- Delivers guidance on execution of similar interventions in different fields involving participatory learning within communities
- Compares the approach of utilising community assets and champions to usual care as an innovative, scalable health service model to reduce ethnic health inequalities
- Provides rich contextual data to improve and address issues that arise during trial implementation through assessing a range of qualitative and quantitative outcome measures.

Limitations

- Triangulating data from participant self-report, implementers' report, structured observation by independent observers and routine data could improve outcome measurement.
- Due to the nature of the intervention, neither participants nor all research staff will be fully masked to allocation, which may introduce the risk of performance or measurement bias.

For peer review only

For peer review only - http://bmjopen.bmj.com/site/about/guidelines.xhtml

INTRODUCTION

The first 1,000 days of life is important for growth and brain development. There is mounting evidence that influences during pregnancy and infancy may alter lifetime risk of nutrition and dental related diseases [1]. Feeding practices developed during this period can impact children's nutrition, growth, dental health, cognitive development, and may lead eventually to heart disease, obesity, and diabetes [2].

Britain's ethnically diverse population is mostly disadvantaged across a range of socioeconomic outcomes, forming fundamental causes of ethnic health inequalities in the UK [3]. Some of the widest differences have been observed in the South Asian (SA) population, more so for Pakistani and Bangladeshi communities compared to Indians [4].

Systematic reviews assessing complementary feeding practices and the sociocultural beliefs underpinning them in children <2 years within SA families in the UK were explored. Despite the adoption of the WHO Infant and Young Children Feeding Guidelines, there remains substantial evidence of non-recommended complementary feeding practices such as early introduction of solids, introduction of minimum dietary diversity or minimum frequency of meals being followed [5-7]. Contributing factors that persisted post-migration included bicultural issues or low acculturation levels and conflicting information among health professionals, extended family, and religious and community leaders.

Effective early life interventions tailored to different ethnic groups have great potential to reduce the development of short- and long-term conditions and, thereby, lifetime inequalities. At present, however, few of such interventions exist [8]. Traditionally, UK health services provide unidirectional information based on guidelines and NHS recommendations. Specific ethnic groups may be marginalised by this approach as most advice is not tailored to their cultural practices. There is a need for interventions that target these communities through a top-down unidirectional approach and bring a change originating from within the communities. Therefore, it is important to work in partnership with communities thereby building their capacity to work closely with local authorities/stakeholders. The asset-based community development approach (ABCD) has been successful in community development as this helps communities identify their assets through asset mapping and mobilize them to bring along the desired change [9].

Based on Lakhanpaul et al.'s MARKS (Motivation Awareness Resources Knowledge and Skills) model [10], current practice lacks the space to understand parents' motivation and ability to support families in skills development, all of which is key to providing optimal infant feeding, care, and dental hygiene support. Considering the resource constraints of the NHS and its 10 Year Forward Plan which aims to shift the emphasis to the community, highlighting a need for interventions that enable communities to utilise their available community assets. The Participatory Learning and Action (PLA) cycle is an iterative process led by multilingual facilitators through a four-stage cycle of identifying and prioritising contextual issues, designing problem-solving strategies, implementing these strategies and a post-implementation evaluation. The PLA approach is a low-cost, community-based, culturally

For peer review only - http://bmjopen.bmj.com/site/about/guidelines.xhtml

sensitive intervention that can be adapted from Low- and Middle-income Countries (LMICs) to High-income Countries (HICs), as well as to different population groups and topic areas. This strategy is also recommended by the WHO to improve maternal and infant survival [11]. A meta-analysis of seven cluster randomised controlled trials (RCTs) utilising PLA in LMICs showed reduced maternal and neonatal mortality by 37% and 23% respectively [12].
An RCT and controlled before-and-after study have demonstrated reduced maternal depression, increased exclusive breastfeeding rates, and decreased under-5 morbidity [13]. A Mumbai-based RCT also showed improvements in maternal practices and care-seeking behaviour [14]. Recognising WHO recommendations and the success of PLA in LMICs, this approach was adopted for the Nurture Early for Optimal Nutrition (NEON) programme.

In NEON Phase 1 (Intervention Development) was co-developed with the SA community facilitators (CF) (now community researchers/independent observers in NEON Phase 2), community members, research team and independent experts (i.e. health visitors, dentists, dieticians, nutritionists, GPs, and midwives). This followed the MRC complex intervention framework , which involved using formative research and prior trials as evidence-based factors; micro-adaptations involved adjusting language, literacy levels for materials, using picture cards, face-to-face, and interactive learning delivery methods. This protocol describes NEON Phase 2: Pilot Feasibility Cluster RCT.

AIMS AND OBJECTIVES

This pilot feasibility study aims to evaluate the feasibility and inform the design and conduct of a definitive cluster RCT comparing NEON Women's Group PLA Cycle versus usual care to optimise infant feeding, care, and dental hygiene practices in SA infants aged <2 years in East London.

Primary objectives:

Assess: 1) the feasibility of proceeding to a definitive trial against predetermined Go/Stop criteria (i.e. recruitment, retention rates, intervention support, acceptability),2) intervention fidelity and participant' adherence, 3) implementation of face-to-face versus online intervention arms

Secondary objectives:

Assess: 1) feasibility, completeness, acceptability and adequacy of blinding when collecting proposed outcomes to establish optimal outcomes and data collection procedures, 2) time needed to competently deliver both versions of NEON intervention, 3)the mean, standard deviation and intervention effect with 95% confidence interval (CI) of the proposed primary outcome, child BMI z-score

For peer review only

METHODA AND ANALYSIS

Design

This three-arm pilot feasibility cluster RCT study is expected to run from July 2021-May 2023 in 3 East London boroughs. 18 clusters defined as borough wards will be randomised with 1:1:1 allocation to 2 intervention arms (face-to-face or online NEON Women's Group PLA Cycle) and 1 control arm (usual care). An online arm enables the trial to continue under Covid-19 restrictions; usual care was selected as control to avoid depriving participants of essential and available infant and maternal care services. Multilingual CFs will deliver interventions in respective ethnic/language groups. Ward-level randomisation is a trade-off between minimising contamination and ensuring maximum SA population representation given the diversity in the ethnic makeup, i.e. SA sub-groups, of the aforementioned boroughs.

Patient and Public Involvement

The research has been co-designed with SA CFs involved in all stages of developing and evaluating the intervention to ensure relevant research questions and an acceptable study design. Development phase, representing different SA communities, are now recruited as Community Researchers (CRs) to support; protocol development and ethics application by ensuring participants-facing documents are clear, appropriate and sensitively-worded; topic guide and questionnaire development; strategy development and troubleshooting (e.g. recruitment); interpretation of findings into appropriate and attainable recommendations for practice; review and revision of draft academic papers; dissemination activities and development of plain language summaries.

Setting

This study will run sequentially in the London Boroughs of Tower Hamlets (TH), Newham (NH) and Waltham Forest (WF), all of which are considered to be among 5% most deprived in England [15]. The boroughs were analysed in terms of ethnic make-up; in each borough, the 6 wards with the highest density of Asian population were selected based on census data [16]. The most common SA ethnic/language groups will be targeted. Figure 1 shows the number of wards and PLA groups by boroughs. Figure 2 shows the overview of participant flow throughout the trial.

Figure 1. Overview schematic detailing number of wards & PLA groups by East London boroughs

Figure 2. Overview of the NEON pilot feasibility RCT design and participant flow

For peer review only - http://bmjopen.bmj.com/site/about/guidelines.xhtml

Ranomisation

Randomization of wards will be done before the recruitment by a separate member at UCL using the Research Randomizer software for the 18 wards stratified by borough [17]. The randomised wards will be shared with the RA at the end of recruitment.Once sufficient numbers are recruited in an ethnic/language group,the RA will assign participants to their respective arms. Recruitment will be performed by NHS personnel who will not be aware of the randomisation. CFs/CRs will be made aware of the participant allocation before the PLA sessions start to make contact and invite participants

Eligibility Criteria

Participants

Inclusion

- Mothers or female carers of an infant <24 months;
- Indian, Pakistani, Sri Lankan or Bangladeshi background;
- Resident in a randomised study ward in TH, NH and WF;
- Willing and able to provide informed consent

Exclusion

- <18 years old;
- Anticipating moving out of *a priori* defined geographical areas before or after delivery
- Current or recent participation in another study within 4 weeks of trial commencement.

Staff (Community Facilitator) Eligibility

- Female
- Have at least one child, preferably <24 months
- From the SA community in TH, NH, or WF
- Able to read and write
- Fluent in English and another local language (Hindi, Bengali, Sylheti, Urdu, or Tamil)
- Understand SA social norms, values and culture within study boroughs
- Known and respected by their local community
- Motivated to address issues related to infant growth and development
- Able to manage a group and have some leadership qualities

For peer review only - http://bmjopen.bmj.com/site/about/guidelines.xhtml

Sample size

With communication and cultural sensitivity as key drivers of the NEON intervention, the sample size will partly depend on the coverage of ethnic and language groupings. The choice of ethnic/language groups were informed by the 2011 ONS Census [16] and input from CFs stressing that, while language is important for communication, people's practices are embedded in different ethnicities.

The main ethnic/language group(s) identified are: 1 group in TH (Bangladeshi/ Bengali & Sylleti); 4 groups in NH (Indian/Gujrati, Indian/Punjabi, Pakistani/Urdu, and Sri Lankan/Tamil); 3 groups in WF (Indian/Punjabi, Pakistani/Urdu, Sri Lankan/Tamil). In each borough, 1 Women's Group PLA Cycle will run per ethnic/language group per ward.

We aim to run 20 to 32 Women's Group PLA Cycle (with equal numbers of face-to-face and online PLA groups) at 6-8 participants per PLA group. Including control groups totals to 288-384 participants. Assuming an 80% recruitment or retention rate, this enables us to be 85% confident that the true population rate will fall between 0.76%-0.84% (precision=0.04). This was estimated using a geometric mean cluster size=16 participants, cluster size coefficient of variation (COV)=0.7 and Intra-Cluster Correlation Coefficient (ICC)=0.02.

Recruitment

Participants

Due to the diversity of the target population, multiple recruitment methods are needed to maximise reach and minimise inequalities or non-representative samples [18] [19]. For instance, relying solely on online recruitment may be ineffective due to digital poverty. Thus, other mediums including leveraging on existing social networks (snowballing), identifying and fostering collaborations with community leaders, and creating clear and succinct recruitment materials [20] were employed. All study promotional materials (e.g. participants information sheets, posters) will be available in English and local languages. To standardise the recruitment process, we will provide the same recruitment script and study materials to all recruiters. Potential participants will contact the RA, who will confirm eligibility, consent and register them into the study.

Participant recruitment will follow three strategies with regular advice by CRs to maximise reach:
1)Recruitment through CFs: CFs will share study materials with their network to invite eligible participants with subsequent snowballing. We will provide training to standardise CF recruitment. 2)Recruitment through Health Visitor (HVs), GPs, and Midwifery teams: HVs, GPs, and midwives who have been identified based on their geographical proximity to study wards will share study materials to eligible participants. Details of those interested will be given to the RA. 3)Online recruitment: Online social media campaigns on Facebook and Instagram will be utilised. See Supplementary Material-1 for advertisement materials. Recruitment bias will

For peer review only - http://bmjopen.bmj.com/site/about/guidelines.xhtml

be limited due to the above-mentioned multiple channels of recruitment and all recruiters will be blinded thereby not knowing the allocation details at the time of recruitment.

Staff

We will recruit CFs from our local stakeholders' network. Recruitment via CFs is particularly useful for studies with underserved or traditionally hard-to-reach populations [21]. CFs are leaders/champions in their local community who, with training and support, help improve the health and wellbeing of their communities. In line with NIHR's "INVOLVE" guideline, CFs will be paid £30 for each PLA meeting and additional £5 to cover travel costs for face-to-face meetings. A comprehensive manual for the CFs will be developed with PLA experts. A tool kit will be provided to support the learnings. The CFs will attend a three-day comprehensive workshop and additional sessions will be conducted in between the 8-meeting cycle as a refresher and address any challenges. Biweekly meetings with the RA will be conducted to discuss learnings from the sessions. The CFs will be paid through vouchers for their time similar to a zero-hour contract. HV, GP and midwives would be identified and recruited by our study partners at each study ward. Payments will be supported by NHS support costs and decided with the relevant clinical lead.

Allocation concealment and blinding

Firstly, treatment allocation will be concealed from participants and recruiters during participant recruitment to reduce recruitment bias. After recruitment has been completed for that ethnic/language group in the borough, the RA will reveal ward allocation to participants and CFs via text or email, listing postcodes by trial arms to allow CFs to effectively organise and deliver the intervention.

Secondly, outcome assessors (CRs) will be blinded where possible to reduce measurement bias. This will be achieved by CRs logging the data collected onto an electronic platform that automatically links participant ID to maintain blinding. Baseline measurement of BMI will be done by health visitors in each borough who will be blinded.

Intervention

NEON Women's Group PLA Cycle

The co-adapted NEON Women's Group PLA Cycle tailored to ethnic/language groups will be delivered face-to-face at community/children's centre or online over 14 weeks (1 session per 2 weeks). HVs, GP, and midwives' teams will be invited to 1-2 sessions to provide evidence-based information upon participants' request. To reduce study contamination, participants may not switch wards (i.e. switch arms) after treatment allocation is revealed. Figure 3 illustrates the aim of each PLA session.

Figure 3. NEON Women's Group PLA Cycle

A culturally-sensitive intervention package has been co-developed consisting of:

1. PLA group facilitator manual
2. Picture cards with recommended and non-recommended infant feeding, care and dental hygiene practices, and facilitators/barriers to uptake
3. Healthy baby food recipes

For peer review only

4. Community asset maps (e.g. low-cost fruit/vegetable shops)
5. List of relevant resources and services.

Online arm participants may access this toolkit through the eRedbook platform and receive NEON information the via the app or website. A Logic Model (Supplementary Material-2) shows the relationship between these activities and resources and their intended short-, medium- and long-term effects.

Control (Usual Care)

In all wards, HV teams have regular mandatory postnatal visits for all families of new-borns and infants. These visits are immediately after birth, 6-8 weeks, 12-16 weeks, 1 year, and 2-2.5 years. One optional prenatal visit is conducted by HVs between 28-32 weeks of pregnancy. Supplementary Material-3 lists other initiatives and resources available in each study borough as part of usual care.

Outcome

Primary outcomes: Feasibility and process outcome measures
We will collect quantitative and qualitative data to assess Go/Stop criteria (Table 1) on recruitment and retention rates, intervention support, acceptability, fidelity and participant adherence using:

1. *Study record:* An enrolment log will record all eligible participants, total enrolled, reasons for non-participation, number followed-up on child BMI z-score, and the date on how many people responded to adverts/invitations. Pre-screen failure logs will record those eligible but not enrolled, with reasons. PLA session audio/video recordings will be collected using secured Dictaphone, video cameras, CCTV, and/or Zoom recording.

2. *Process measurements:* These include Participants' Feedback Questionnaire, Facilitator's Feedback Form, PLA Cycle Meeting Register, direct observation of intervention delivery and CF performance by CRs, and Sustainability Assessment Tool (Supplementary Material-4).

Definite Go	Definite Stop
>=50% of eligible participants consenting to pilot feasibility study	<40% of eligible participants consenting to pilot feasibility trial
>=80% of mothers attend >=60% of planned sessions in the intervention arm.	<20% of mothers attend >=60% of sessions as planned in each intervention arm
Retention of >=70% of consented participants for primary outcome data collection	Retention of <50% of participants at 6 months
High intervention support with respect to content, frequency, duration, and quality	Low support of Intervention Procedures

For peer review only - http://bmjopen.bmj.com/site/about/guidelines.xhtml

Intervention is perceived as acceptable	Intervention perceived as unacceptable

Table 1: Go/ Stop criteria for the definitive trial[3]

Secondary outcomes: Proposed outcomes for the definitive trial

All other individual-level, cluster-level and economic outcome measures proposed for the definitive trial will be assessed on the response, completion rates, acceptability and the adequacy of blinding for outcome assessors during data collection. Table 2 summarises outcome measures and data collection timing. See Supplementary Material-5 for collection tools.

Outcome	Outcome Measures	Data Collection	Timing			
			Baseline	Every 2 weeks (end of each PLA meeting)	14 weeks (end of PLA cycle)	6 months
**Proposed primary individual-level outcome**	Individual Child BMI z-score	RA, CRs	✓		✓	✓
**Proposed secondary individual-level outcome**	Children feeding behaviour[3]	RA, CRs	✓		✓	✓
	Parental feeding style[3]	RA, CRs	✓		✓	✓
	Audio/Video recording of child eating behaviours & parental feeding practices	RA	✓		✓	✓
	4-day food diary[4]	RA, CRs	✓		✓	✓
	Network diffusion†[3]	RA, CRs	✓			✓
**Proposed secondary cluster-level outcome**	Children's development performance	RA, CRs		✓		✓
	Level of dental caries	RA	✓			✓

[3] The study will have to meet the Definite Go criteria in order for the study to be feasible and to be able to proceed. However, if any one of the Definite Stop criteria is met then the study stops. The 'Go/Stop' indicates that it is either feasible or not feasible to proceed to a definitive trial. The progression rules have been approved by the steering and data management team. Should any of the progression rules not be met, assessments and adjustments of the NEON pilot feasibility RCT will be negotiated before proceeding to a definitive trial.

[4] Community Facilitators will assist in administering or completing these data collection tools before sending them to RA/ CRs during the intervention period; †For intervention groups only; CR= Community Researchers; RA = Research Assistant

For peer review only - http://bmjopen.bmj.com/site/about/guidelines.xhtml

	GP healthcare utilisation	RA	✓			✓
**Economic outcome**	Cost tool	RA	✓			✓
	Partners time questionnaire	RA				✓
**Process outcome†**	Participants Feedback[3]	RA, CRs		✓		
	PLA cycle meeting register[3]	RA, CRs		✓		
	Direct observation	RA, CRs		✓		
	Sustainability assessment	RA, CRs		✓		

Table 2. Schedule of Outcome Assessments

Primary individual-level outcome

1. *Child BMI z-score:* Health visitors will measure infants' length/height, head circumference and weight. The RA will refer to the WHO 2006 growth standard [2], using age- and gender-adjusted length or height(m)/weight $(kg)^2$ to provide a comparison on the cluster-level.

Secondary individual-level outcomes

1. *Children's feeding behaviour* will be measured using 6 of 8 domains adapted from the validated Children's Eating Behaviour Questionnaire (CEBQ) deemed suitable for infants: food responsiveness; enjoyment of food; emotional overeating; desire to drink; satiety responsiveness; slowness in eating; emotional undereating; and food fussiness [7, 8, 22]. Obesity risk could be linked with external eating, emotional overeating, and food fussiness [7].

2. *Parental feeding style* will be assessed with a validated self-reported Parental Feeding Style Questionnaire (PFSQ) with four scales: emotional feeding; instrumental feeding; encouragement to eat; and control overeating [23].

3. *Audio/video recording of child eating behaviour and parental feeding practices* [24] involves asking participants about the meal (e.g. name, ingredients) and creating annotations that link to behaviour codes identified from NEON formative study [25]. Before-and-after audio/video recordings will be thematically analysed using Elan Software [26] to measure intervention effect.

4. *Child food intake*: The 4-day food diary [7] is a self-reported, prospective, open-ended survey that collects detailed quantitative estimates of food consumption of infants between 4-18 months [6]. It assesses nutrient intake (e.g. total fat, total carbohydrate, salt, sugar) by comparing with age/sex specific UK dietary reference values [27]. Dietary data will be presented by age group (e.g. 4-6 months, 7-9 months, 12-18 months).

5. *Network diffusion of study materials between participants and their communities*: CRs will track the number of downloads using the eRedbook platform. Participants will also complete a questionnaire on the number of people they shared the material with, their family size, age, gender, relationship to the participant and platform used.

6. *Equality impact assessment (EIA)*: This trial should reflect the diversity of the target population by enabling equal access while accommodating different needs. An EIA tool will systematically assess likely effects of the intervention on people (e.g. with respect to disability,

For peer review only - http://bmjopen.bmj.com/site/about/guidelines.xhtml

gender, ethnicity, age, sexual orientation, religion/belief etc.), mitigate adverse impacts and identify active steps to address existing disadvantage and promote equality.

Secondary cluster-level routine outcomes

Although this intervention acts on the individual-level, we expect a social diffusion of good infant feeding and dental hygiene awareness and practices in the medium- to long-term (Supplementary Material-2, Logic Model). While immediate cluster-level changes are not expected, we would like to pilot cluster-level routine data collection.

1. Development performance of children: The Ages and Stages questionnaire (ASQ-3) include communication, gross motor, fine motor, problem-solving, and personal-social domains [5]. Three versions are available: 24-months, 27-months and 30-months. ASQ-3 data collected by HVs during mandated visits are stored in EMIS/ RIO databases.
2. GP healthcare utilisation data is available in participants' medical record.
3. Level of dental caries and other signs of tooth decay is available in NHS dental services records for registered patients beginning at 6-months of age and approximately every half-year following initial visit [17,28,29].

Economic outcomes

1. Cost: The RA will use a spreadsheet will capture the cost of the NEON Intervention Development
and pilot feasibility RCT phases every 6 months.
2. Partners' time used: A questionnaire will capture time spent for the delivery of NEON-associated programmes.

Data Collection and Management

Outcome data will be collected at baseline, every 2-weeks during intervention (for process measures), 14-weeks immediately post-intervention, and 6-months follow-up (for economic data). During the intervention period, several outcome measurements will be administered by CFs to participants or completed by CFs and sent to CRs/RA. Other outcomes and post-intervention outcomes will be assessed directly by CRs/RA (Table 2). Each case will be assigned an ID. Data collected digitally or in paper format will be stored in UCL S: Drive and/or UCL cabinets. We will use RedCap electronic data management system [30]; the RedCap API allows flexible and straightforward data import and export to data analysis software.
We will form a data sharing agreement with study partners for the RA to extract and link routine cluster-level data to trial participants. To guarantee confidentiality, pseudonymised participant data will be stored on encrypted, password-protected computer or UCL S:Drive accessible only to study staff and authorised personnel. Password-protected personal data will be stored separately from trial data on secure UCL computers. Only pseudonymised quotes or data from audio/video recording may be published. Personal data and audio/video recordings will be stored for 3-years post-study and destroyed; research data will be stored for 20-years post-study.

Analysis

For peer review only - http://bmjopen.bmj.com/site/about/guidelines.xhtml

Overall, quantitative and qualitative data will be analysed concurrently at multiple time points to identify early problems that are rectified as the trial progresses. Quantitative data such as the BMI z-score will be descriptively summarised using mean and standard deviation (SD) for continuous variables, number with percentages for categorical variables, and 95% CI with a breakdown of participants by trial arm, ethnic/language group and borough where relevant. An intention to treat analysis will also be considered. Results will be collectively assessed against the Go/Stop criteria. Before progression to the definitive trial, we will systematically and rigorously assess areas of improvement using a structured discussion tool: A process for Decision-making after Pilot and feasibility Trials (ADePT) [31]. All statistical analysis will be done by an independent statistician who would be blinded.

Sample Characteristics

Participant demographic characteristics will be descriptively summarised by trial arm. We will check for recruitment bias across different arms at baseline and compare later recruits who are prone to unblinded recruitment.

Feasibility and Process Measures

Across the three arms, we will check for any differential recruitment or retention rates and any consequent systematic differences in participant characteristics over time, as control arm participants may be less keen to participate or remain in the study. For the two intervention arms, we will conduct a mixed-methods implementation evaluation to explore the possibility of a blended approach in the definitive trial as some features of each delivery mode might be particularly salient. Qualitative data will be analysed thematically using framework analysis that include participants' motivation for engagement, expectations, experiences, intervention acceptability, implementation barriers, and suggestions for improvement. Negative 'deviant' cases will inform interpretation.

Outcome measures for the definitive trial

1. *Feasibility of collecting outcome measures:* To assess completeness, degrees of missingness at the individual item level and the entire outcome measure will be reported overall, by time-point and trial arms. Other feasibility measures would be captured by analyses of process measures above.

2. *Intervention effect for proposed primary outcome (BMI z-score):* Conditioned upon low recruitment bias and sufficient adherence to trial protocol, we will estimate the difference in BMI z-score between each intervention arm versus the control arm. We will consider methods to pool data across age groups depending on the actual sample age breakdown.

3. *Sample size and power calculation for the definitive trial:* Data from the pilot feasibility trial will enable us to determine (i) the smallest difference of clinical importance (through multiple testing of several key endpoints for the proposed primary outcome), (ii) the clinically justifiable power-significance level or scientifically acceptable probability of "false positive" (type I error) results, and (iii) whether we need to adjust the calculated sample size for expected level of non-compliance/ drop-in and drop-outs. We will report the ICC.

4. *Economic Evaluation:* This will be conducted from a provider and user perspective which includes direct cost (e.g. time, resources spent on training and participation of CF, participants' overall household consumption and childcare costs). We will estimate the total cost, average annual cost, cost-effectiveness, equity impact analysis and compare across three arms.

Monitoring

The Principal Investigator (PI) will be responsible for day-to-day monitoring and study management.

For peer review only - http://bmjopen.bmj.com/site/about/guidelines.xhtml

For peer review only

The NEON Steering and Data Management Team will ensure adherence to all relevant regulations and principles of good clinical practice. The team consists of multidisciplinary experts in child health, practitioners, PLA expert, SA independent observers from NEON 1, and Queen Mary University of London Pragmatic Clinical Trials Unit. Committee meetings will be held every 3 months to review the Go/Stop criteria (Table 1) and interim analyses. The team will agree to the trial protocol and facilitate any necessary protocol amendments; provide independent expert advice (including on project management governance); monitor progress; ensure adequate deadlines are set and met; monitor and advise NEON Core Team (Trial Management Group) on strategic decisions in light of new evidence; and ensure successful delivery by meeting and reporting on study progress with the Core Team biannually. The PI and other Core Team members will attend all necessary meetings and report on the study progress.

Any adverse events or safety concerns will be captured in the Participant Feedback Questionnaire and Facilitator Report Form (every 2-weeks during intervention period) and reported to the steering and safety monitoring team.

The UCLH/UCL Joint Research Office, on behalf of the Sponsor (UCL), may conduct random audits in accordance with the UK Policy Framework for Health and Social Care Research and UCL's policies and procedures.

ETHICS AND DISSEMINATION

Ethical approval will be obtained from UCL Research Ethics Committee and NHS Health Research Authority. The participant information sheet will be provided in English or participants' native language in-writing and/or verbally by community facilitators for informed consent. The RA or CRs will obtain written or audio-recorded consent ≥24 hours later [32]. Participation will be voluntary and they may withdraw anytime without prejudicing treatment or usual care. No incentives will be provided except for small reimbursements (e.g. childcare at the local centre where PLA meetings take place).

UCL will provide insurance cover and we will also signpost any vulnerable participants who require protection from harm to relevant safeguarding organisations.

Regular progress updates will be shared via UCL or NIHR websites and patient group newsletters. Study findings will be submitted for 3-5 publications in high impact factor peer-reviewed journals and presented at national and international conferences. Researchers and community members from the steering team will contribute to confirming analysis and write-up, adhering to authorship guidelines. To reach wider audiences, we will (i) disseminate plain language summaries for each academic paper through community groups, social media and You Tube; (ii)present findings at lay-person meetings at community or children centres through the NIHR CLAHRC network and (iii)organise 2-3 annual workshops with commissioners, partner organisations and NEON community facilitators to share key findings and recommendations.

Acknowledgements

The authors would like to thank the South Asian community facilitators in the NEON Intervention Development phase: Lily Islam, Seema Bajwa, Aasma Baiyat, Jasvir Bhachu, Geromini Pushpakanthan, Sabiha Malek, Cllr Lakmini Shah; and community members of the London Boroughs of Tower Hamlet, Newham, and Waltham Forest for their important contribution and engagement to this research project. Furthermore, the authors would like to thank the 'Conversation Café'

For peer review only - http://bmjopen.bmj.com/site/about/guidelines.xhtml

initiative led by Cllr Lakmini Shah for her significant help in the recruitment of the community facilitators and members, and in identifying the community centres to carry out the intervention development meetings. The authors would also like to acknowledge the contribution of the NEON core team, steering team and all health experts in validating intervention NEON toolkit. We would like to thank the Women & Children First Charity and First Steps Nutrition Trust for their valuable contributions and guidance throughout the study.

Author's Contributions

ML, LM, MH, NB, CL, AH, SA conceived the original concept of the study and designed the research methodology. SA carried out the Intervention Development meetings, workshops, co-development of the intervention toolkit, wrote and devised the paper along with LI, SB, SM, JB and GP. LM, ML, SA, PP, MN, ZO, NB, CL, AH, RL, JG, KWM, CI, MA, CC, DD validated the study and revised the manuscript critically for important intellectual content. ICD was involved in drafting materials for the NEON meetings and workshops of the Intervention Development Phase. MN and ICD contributed to the manuscript writing, and prepared it for submission. LM, SA, ML had primary responsibility for the final content. All authors read and contributed to reviewing the study data, the designing of the manuscript, and the approval of the final manuscript

Funding

Logan Manikam and Shereen Al Laham are funded via a National Institute for Health Research (NIHR) Advanced Fellowship (Ref: NIHR300020) to undertake the Pilot Feasibility Cluster Randomised Controlled Trial of the *NEON* programme in East London. Prof Monica Lakhanpaul was funded by the NIHR Collaboration for Leadership in Applied Health Research and Care (CLAHRC) North Thames.

Disclaimer

The views expressed in the publication are those of the author(s) and not necessarily those of the sponsor (UCL), funder (NIHR), study partners (Tower Hamlets GP Care Group, London Borough of Newham Council, Waltham Forest Council).

Conflicts of Interest

The authors declared no potential conflicts of interest with respect to the research, authorship and/or publication of this paper.

Participant consent for publication

Participant information sheets and consent forms have been provided to community members and those expressing interest. Community participants have agreed to participate and given audio/video consent prior to their participation in the workshops. Verbal consent was witnessed and formally recorded. All participants have been informed of their right to freely withdraw from the study at any time. Confidentiality of personal data has been ensured through the use of anonymisation techniques as stated in the Data Protection Act (1998) and in line with the General Data Protection Regulation (2018). All participant data has been anonymised and stored on an encrypted password protected computer. Data can only be accessed by the authorised research personnel.

For peer review only - http://bmjopen.bmj.com/site/about/guidelines.xhtml

Ethics approval

This study has obtained ethical approval from UCL Research Ethics Committee [Ethics ID 17269/001], Sponsor reference number: 142600, Funding Reference: NIHR300020 and IRAS number: 296259, Ref: 21/SW/0142. Study protocols and relevant documents will be reviewed by UCL Research Ethics Committee, NHS Health Research Authority (HRA) and study partners involved in establishing data sharing agreement for linking participants' routine data (Tower Hamlets GP Care Group, London Borough of Newham Council, Waltham Forest Council).

Provenance and Peer Review

Not commissioned; peer reviewed for ethical and funding approval prior to submission.

Data Sharing Statement

The data supporting the findings of this study is available upon reasonable request from the corresponding author.

CURRENT STUDY STATUS (AS OF JAN 2023)

The study is currently in the intervention delivery phase.
There has been an overall delay in the timeline of the study due to the COVID - 19 pandemic, lockdown and NHS staff shortage in our study wards.

Recruitment for the study commenced in March 2022 and was completed in Sep 2022. Following recruitment, we launched our first PLA meeting in Tower Hamlets in Oct 2022 and then in Newham in the week following. Waltham Forest dropped out of the study due to lack of supporting staff in the recruitment phase.

We have completed 5 out of the 8 PLA meetings and the PLA meeting cycle will conclude in Feb 2023. Following this we will initiate data analysis and reporting of study findings. We will collect follow-up data in April 2023 which will be the last data collection time point for this trial.

REFERENCES

[1] World Health Organization (WHO), Postnatal care for mothers and newborns: Highlights from the World Health Organization 2013 Guidelines. Available from: http://www.who.int/maternal_child_adolescent/publications/WHOMCA-PNC-2014-Briefer_A, 2015. 4.

[2] Group, W.M.G.R.S., WHO Child Growth Standards based on length/height, weight and age. Actapaediatrica (Oslo, Norway: 1992). Supplement, 2006. 450: p. 76.

For peer review only

[3] Evandrou M, Falkingham J, Feng Z, et al Ethnic inequalities in limiting health and self-reported health in later life revisited J Epidemiol Community Health 2016;70:653-662.

[4] Brown MS. Religion and economic activity in the South Asian population. Ethnic and Racial Studies. 2000;23(6):1035-61

[5] ASQ-3. The Ages & Stages Questionnaires, Third Edition. [cited 2019 30 November]; Available from: https://agesandstages.com/products-pricing/asq3/.

[6] Lennox, A., et al., Diet and nutrition survey of infants and young children, 2011. A survey carried out on behalf of the Department of Health and Food Standards Agency, 2013.

[7] NATIONAL DIET AND NUTRITION SURVEY: Food and Drink Diary. 2010; Available from: https://webarchive.nationalarchives.gov.uk/20130413115649/http://media.dh.gov.uk/network/261/files/2012/07/Appendix-E_All-docs-combined-v2.pdf.

[8] 2011. Early Years Interventions To Address Health Inequalities In London – The Economic Case.[ebook] London: Greater London Authority. Available at:<https://www.london.gov.uk/sites/default/files/gla_migrate_files_destination/Early%20Years%20report%20OPT.pdf> [Accessed 5 January 2021].

[9] High S, Mckie I, Mackay M, et alO-09 Beyond traditional approaches to community engagement: using asset based community development to discover, connect and mobilise communities to support each other right to the end of lifeBMJ Supportive & Palliative Care 2022;12:A4.

[10] Lakhanpaul, M., 2019. PANCHSHEEEL - MRC HEEE STUDY. [online] Ucl.ac.uk. Available <https://www.ucl.ac.uk/child-health/sites/child-health/files/newsletter_3_0.pdf

[11] WHO. WHO recommendation on facilitated participatory learning and action cycles with women`s groups during pregnancy: The WHO Reproductive Health Library 2018 [from: https://extranet.who.int/rhl/topics/improving-health-system-performance/implementation-strategies/who-recommendation-facilitated-participatory-learning-and-action-cycles-womens-groups-during.

[12] Seward N, Neuman M, Colbourn T, Osrin D, Lewycka S, Azad K, et al. Effects of women's groups practising participatory learning and action on preventive and care-seeking behaviours to reduce neonatal mortality: A meta-analysis of cluster-randomised trials. PLoS medicine. 2017;14(12):e1002467.

[13] Tripathy P, Nair N, Barnett S, Mahapatra R, Borghi J, Rath S, et al. Effect of a participatory intervention with women's groups on birth outcomes and maternal depression

For peer review only - http://bmjopen.bmj.com/site/about/guidelines.xhtml

in Jharkhand and Orissa, India: a cluster-randomised controlled trial. The Lancet. 2010;375(9721):1182-92

[14] Prost A, Colbourn T, Seward N, Azad K, Coomarasamy A, Copas A, et al. Women's groups practising participatory learning and action to improve maternal and newborn health in low-resource settings: a systematic review and meta-analysis. The Lancet. 2013;381(9879):1736-46

[15] Mayor of London. London’s socio-economic issues. 2016; Available at:https://www.london.gov.uk/sites/default/files/chapter10-economic-evidence-base-2016.pdf.Accessed 08/06/, 2020.

[16] ONS. 2011 Census Data. 2011; Available at: https://www.ons.gov.uk/census/2011census.

[17] Faculty of Dental Surgery. 2015. The state of children’s oral health in England. [PDF]

[18] Newington L, Metcalfe A. Factors influencing recruitment to research: qualitative study of the experiences and perceptions of research teams. BMC Med Res Methodol. 2014;14:10. Published 2014 Jan 23. doi:10.1186/1471-2288-14-10

[19] Office for National Statistics. 2020. Domestic Abuse During The Coronavirus (COVID-19) Pandemic, England And Wales - Office For National Statistics. [online] Available at: <https://www.ons.gov.uk/peoplepopulationandcommunity/crimeandjustice/articles/domesticabuse duringthecoronaviruscovid19pandemicenglandandwales/november2020> [Accessed 10 January 2021].

[20] Joseph RP, Keller C, Ainsworth BE. Recruiting Participants into Pilot Trials: Techniques Researchers with Shoestring Budgets. Calif J Health Promot. 2016;14(2):81-89.

[21] An innovative approach to recruiting homebound older adults. Crawford Shearer NB, Fleury JD, Belyea MRes Gerontol Nurs. 2010 Jan; 3(1):11-8.

[22] Llewellyn, C.H., et al., Development and factor structure of the Baby Eating Behaviour Questionnaire in the Gemini birth cohort. Appetite, 2011. 57(2): p. 388-396.

[23] Daniels, L.A., et al., Outcomes of an early feeding practices intervention to prevent childhood obesity. Pediatrics, 2013. 132(1): p. e109-e118.

[24] Fogel, A., et al., Prospective associations between parental feeding practices and children's oral processing behaviours. Maternal & child nutrition, 2019. 15(1): p. e12635.

For peer review only

For peer review only - http://bmjopen.bmj.com/site/about/guidelines.xhtml

[25] Lakhanpaul M, Benton L, Lloyd-Houldey O, et al. Nurture Early for Optimal Nutrition (NEON) programme: qualitative study of drivers of infant feeding and care practices in a British-Bangladeshi population. BMJ Open 2020;10:e035347. doi:10.1136/ bmjopen-2019-035347

[26] Tla.mpi.nl. 2020. ELAN | The Language Archive. [online] Available at: http://tla.mpi.nl/tools/tla-tools/elan

[27] Health Do. Infant feeding recommendation. Department of Health London, England; 2003.

[28] 2019. Dental Record Keeping Standards: a consensus approach. [PDF] NHS England and NHS Improvement.

[29] Faculty of General Dental Practice, 2012. 7. Collecting Data. Available at: <https://www.fgdp.org.uk/research-primary-dental-care/7-collecting-data> [Accessed April 15, 2021].

[30] Lawrence, C.E. et al. A REDCap-based model for electronic consent (eConsent): Moving toward a more personalized consent. Journal of Clinical and Translational Science. 2020; 4(4):345–353.

[31] Bugge C, et al. A process for Decision-making after Pilot and feasibility Trials (ADePT): Development following a feasibility study of a complex intervention for pelvic organ prolapse. Trials.2013 Oct 25;14(1):353.

[32] Health Research Authority. 2018. HRA And MHRA Publish Joint Statement On Seeking And Documenting Consent Using Electronic Methods (Econsent). [online] Available at: <https://s3.eu-west-2.amazonaws.com/www.hra.nhs.uk/media/documents/hra-mhra-econsent-statement-sept-18.pdf> [Accessed 8 January 2021]

For peer review only

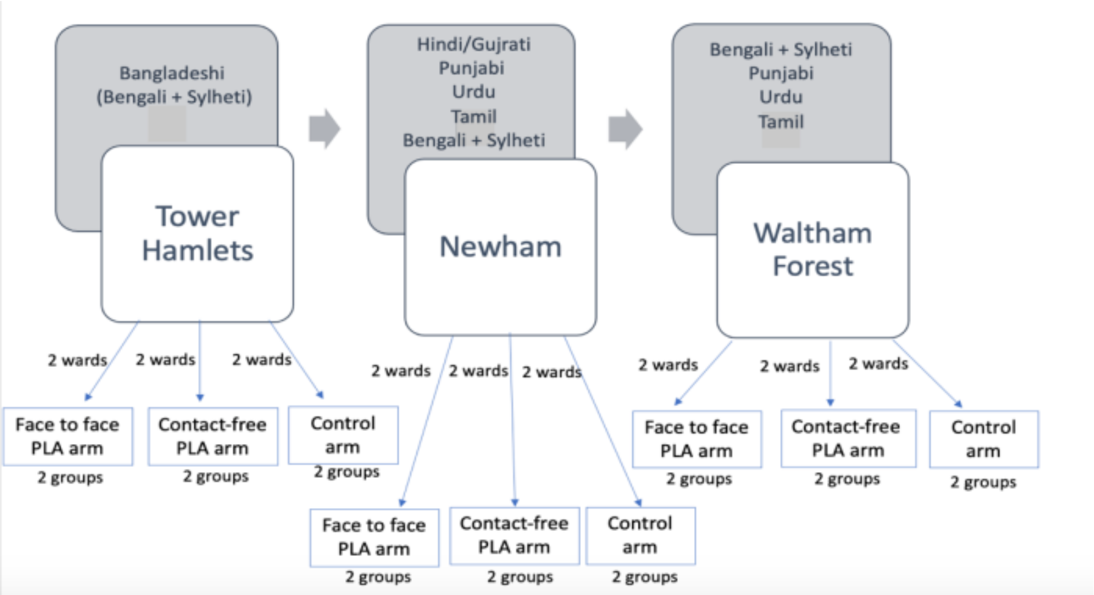

Figure 1. Overview schematic detailing number of wards & PLA groups by East London boroughs

Footnote: To illustrate, TH will have 2 face-to-face Bangladeshi/ Bengali & Sylheti PLA groups (1 per ward), 2 groups of Bangladeshi/ Bengali & Sylheti online PLA group (1 per ward), and 2 Bangladeshi/ Bengali & Sylheti control groups (1 per ward). The more the ethnic/ language group in the borough, the more the intervention and control groups.

For peer review only

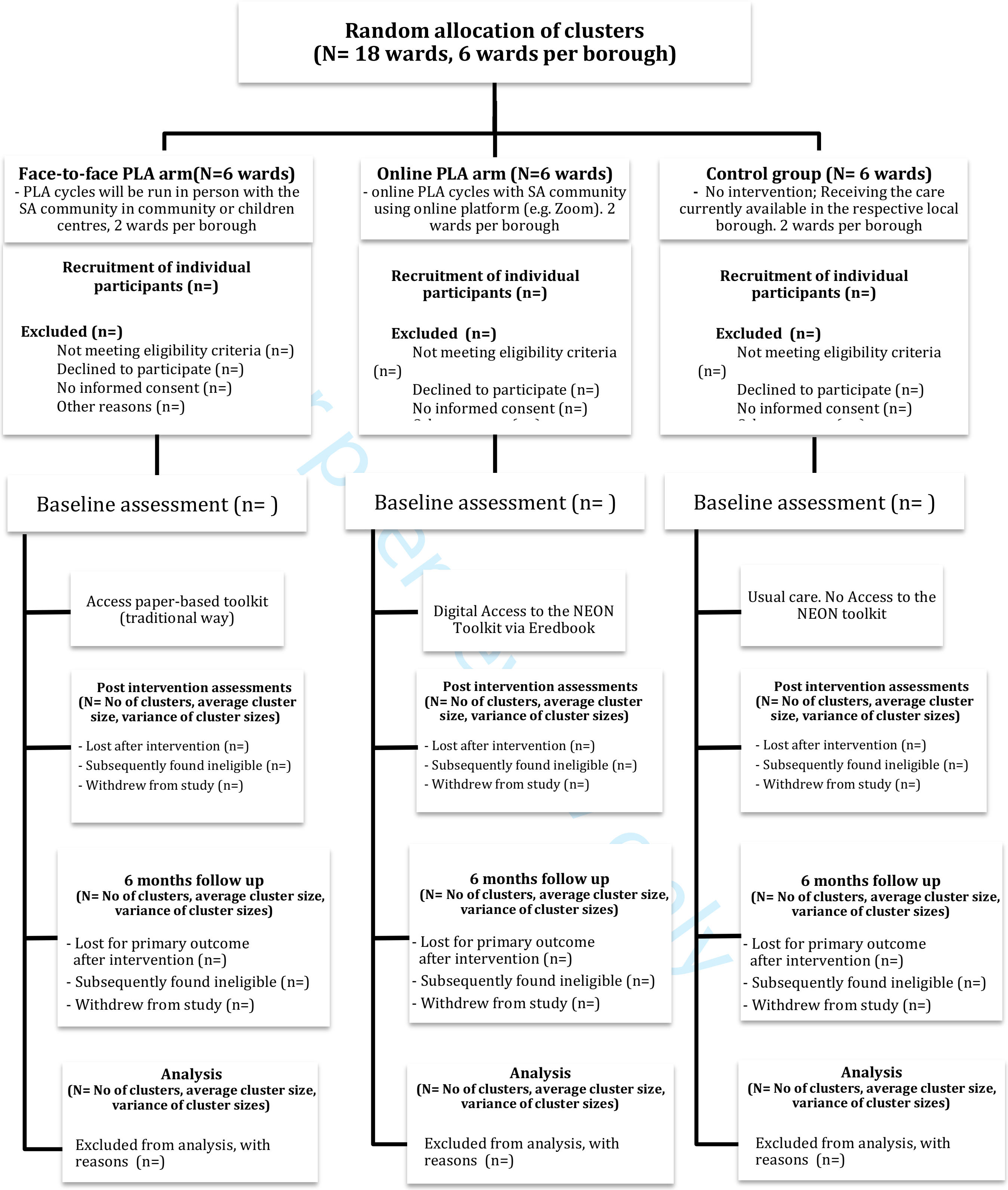

Figure 2. Overview of the NEON pilot feasibility RCT design and participant flow

For peer review only - http://bmjopen.bmj.com/site/about/guidelines.xhtml

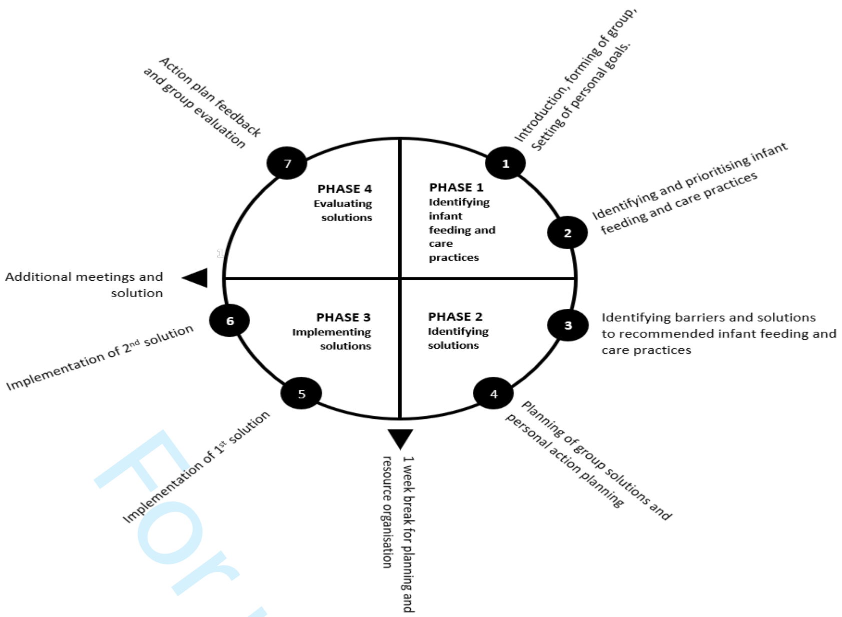

Figure 3. NEON Women's Group PLA Cycle

For peer review only

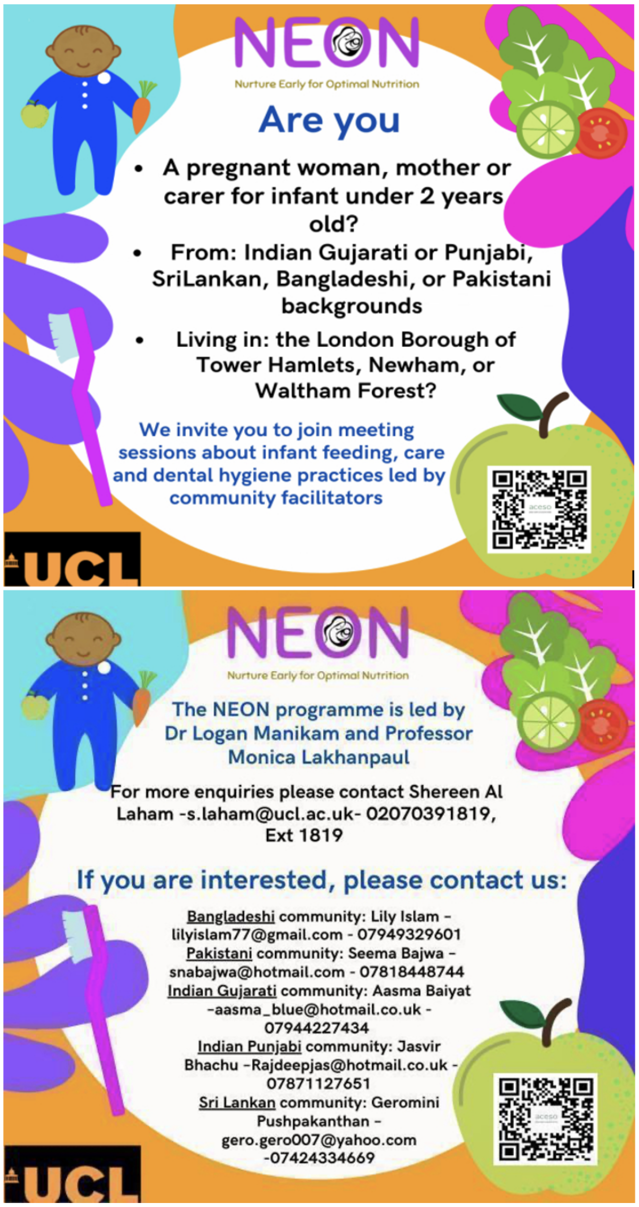

125x234mm (144 x 144 DPI)

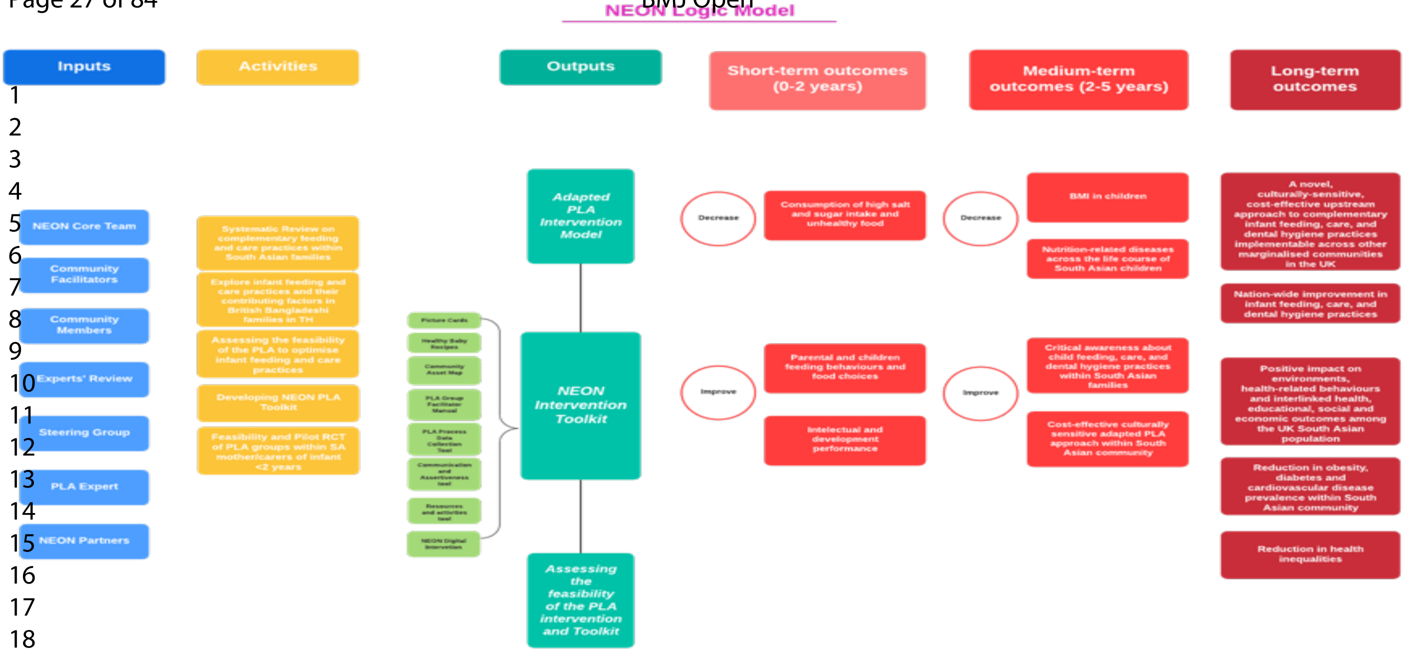
NEON Logic Model
Inputs
Activities
Outputs
Short-term outcomes (0-2 years)
Medium-term outcomes (2-5 years)
Long-term outcomes
NEON Core Team
Community Facilitators
Community Members
Experts' Review
Steering Group
PLA Expert
NEON Partners
Systematic Review on complementary feeding and care practices within South Asian families
Explore infant feeding and care practices and their contributing factors in British Bangladeshi families in TH
Assessing the feasibility of the PLA to optimise infant feeding and care practices
Developing NEON PLA Toolkit
Feasibility and Pilot RCT of PLA groups within SA mother/carers of infant <2 years
Picture Cards
Healthy Baby Recipes
Community Asset Map
PLA Group Facilitator Manual
PLA Process Data Collection Tool
Communication and Assertiveness tool
Resources and activities tool
NEON Digital Intervention
Adapted PLA Intervention Model
NEON Intervention Toolkit
Assessing the feasibility of the PLA intervention and Toolkit
Decrease
Consumption of high salt and sugar intake and unhealthy food
Improve
Parental and children feeding behaviours and food choices
Intellectual and development performance
Decrease
BMI in children
Nutrition-related diseases across the life course of South Asian children
Improve
Critical awareness about child feeding, care, and dental hygiene practices within South Asian families
Cost-effective culturally sensitive adapted PLA approach within South Asian community
A novel, culturally-sensitive, cost-effective upstream approach to complementary infant feeding, care, and dental hygiene practices implementable across other marginalised communities in the UK
Nation-wide improvement in infant feeding, care, and dental hygiene practices
Positive impact on environments, health-related behaviours and interlinked health, educational, social and economic outcomes among the UK South Asian population
Reduction in obesity, diabetes and cardiovascular disease prevalence within South Asian community
Reduction in health inequalities

RESOURCES AND SERVICES SUPPORTING INFANT FEEDING, CARE AND DENTAL HYGIENE

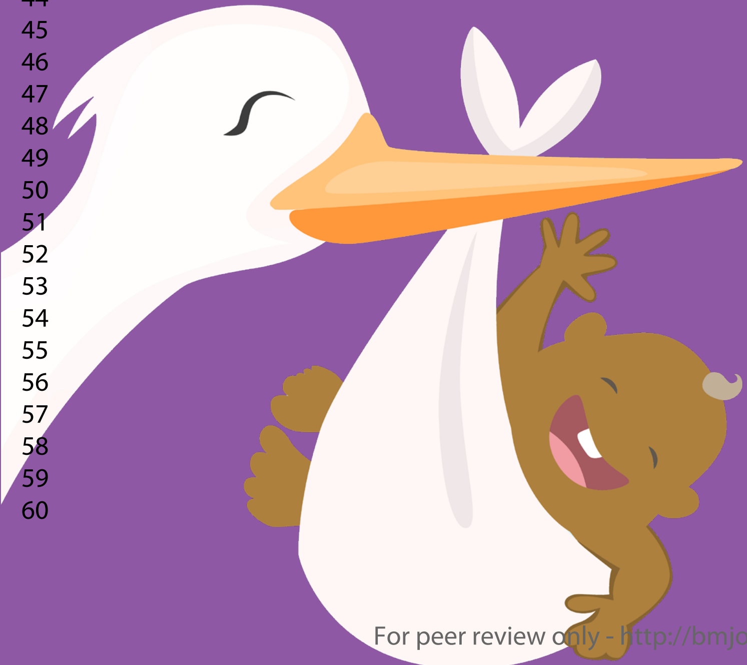

NEON
Nurture Early for Optimal Nutrition

NHS AND COUNCILS ADVICE AND SUPPORT

1. NHS STAR4LIFE

https://www.nhs.uk/start4life

Website provides trusted NHS help and advice for parents and carers during pregnency, and birth and the early years.

2. NHS CHOICES

NHS Choices (www.nhs.uk) is the official website of the National Health Service in England and provides comprehensive health information service with thousands of articles, videos and tools. Key pages which give guidance on infant feeding and care include:

- **Your newborn:** *https://www.nhs.uk/conditions/pregnancy-and-baby/childrens-meal-ideas/?tab-name=your-newborn*
- **Babies and toddlers** *https://www.nhs.uk/conditions/pregnancy-and-baby/childrens-meal-ideas/?tabname=ba-bies-and-toddlers*

3.CHILDREN CENTRES AND LIBRARIES

Tower Hamlets has 12 children centres across the borough providing a range of services to infants, children and families to give them the support they need to be safe, healthy and happy, so that they are able to reach their full potential. This web page on the **Tower Hamlets Council** website gives locations and contact details of all the children's centres in the borough: *https://www.towerhamlets.gov.uk/lgnl/education_and_learning/childcare_and_early_years_educ/Children_centres/childrens_centres.aspx*

The borough of Newham offers support to its residents with children under the age of 5 through **Best Start in Life (BSiL)** Children's centres. These include play activities and childminder services, as well as supporting parents to improve their confidence, support their children with healthy eating and weaning. Libraries offer free courses and internet services for parents, and they offer devices and digital equipment to borrow.

Locations and details can be found on the Families Newham website: *https://families.newham.gov.uk/kb5/newham/directory/family.page?familychannel=3-2*

Waltham Forest offers to its families a similar service, with support for healthy development and access to financial support. They have implemented the 2 Year Old Partnership Pathway in which professionals empower and inform parents to assure the best start in life for their children. They offer advice on a wide range of issues including health, finance and cognitive/motor development. **Waltham Forest** offers support directed at healthy weight and eating through HARRY.

More information can be accessed via the following link: *https://www.walthamforest.gov.uk/content/2yo-partnership-pathway*

Further informal support that does not require formal registration can generally be accessed in public libraries

For peer review only

GUIDENCE DOCUMENTS, ONLINE RESOURCES AND APP'S

1. BABY FRIENDLY INITIATIVE

Published by the Department of Health and the Baby Friendly Initiative, the leaflets below provide mothers and health professionals with key information about breast feeding, introducing solid foods to babies, and evidence-based guidance around formula. The **Baby Friendly Initiative** is part of a global partnership between the World Health Organization (WHO) and UNICEF. The guidance below should be prioritised over other online resources (other than NHS Choices) as they the baby friendly initiative is less influenced by commercial interests, which can sometimes influence the advice offered to mothers and parents regarding infant feeding and care.

- **Leaflet on weaning and starting solid food:**
  https://www.unicef.org.uk/babyfriendly/baby-friendly-resources/leaflets-and-posters/weaning-starting-solid-food/

- **Leaflet on breastfeeding:**
  https://www.unicef.org.uk/babyfriendly/baby-friendly-resources/leaflets-and-posters/off-to-the-beststart/ -

- **Leaflet on infant formula:**
  https://www.unicef.org.uk/babyfriendly/baby-friendly-resources/leaflets-and-posters/simple-formula-guide-for-parents/

- **Leaflet on bottle feeding:**
  https://www.unicef.org.uk/babyfriendly/baby-friendly-resources/leaflets-and-posters/guide-to-bottle-feeding/

- **Baby friendly initiative resources in Bengali:**
  https://www.unicef.org.uk/babyfriendly/baby-friendly-resources/leaflets-and-posters/foreign-language-resources/bengali-resources/

- **Baby friendly initiative resources in Hindi:** बच्चे के अनुकूल पहल संसाधन
  https://www.unicef.org.uk/babyfriendly/baby-friendly-resources/foreign-language-resources/hindi-resources/

- **Baby friendly Initiative resources in Urdu:** لئیاس وےک)تشادہگن(ءامنوشنئیادتبایک ےچب
  https://www.unicef.org.uk/babyfriendly/baby-friendly-resources/foreign-language-resources/urdu-resources/

Other Baby Friendly Initiative resources/leaflets:

https://www.unicef.org.uk/babyfriendly/baby-friendly-resources/leaflets-and-posters/

2. FIRST STEPS NUTRITION TRUST

https://www.firststepsnutrition.org

First steps nutrition can also be used as a trusted source of good information and guidance regarding nutrition and feeding in pregnancy, infants, and early years. **First Steps Nutrition Trust** is funded by research or charitable grants and donations, and does not take money from any commercial organisation or any organisation or individual linked to the sales of infant formula and other food or drink product or service which is associated with poor nutritional health of women and children.

For peer review only

First Steps Nutrition Trust offers some useful resources on meal choices and portion sizes, with helpful diagrams to illustrate portion size. Remember! Portion size is a maximum, not a minimum, never encourage a child to eat more when they indicate they have had enough.

- Eating well: the first year – guidance on introducing foods, food choices and meals for up to 7 months, 7-9 months, and 10-12 months: http://www.firststepsnutrition.org/newpages/Infants/first_year_of_life.html
- Eating well: Early years – guidance on food choices, meals, snacks and portion size http://www.firststepsnutrition.org/newpages/Early_Years/eating_well-early_years.html

3. BORN TO MOVE APP

Born to move is a free NHS app to help parents or carers with their newborn babies right up to pre-school, specifically relating to physical activity and play. It's packed with advice, tips and games that you can play with your child to support their development.

https://itunes.apple.com/gb/app/born-to-move/id976095022?mt=8Local%20initiatives/pro-jects

https://play.google.com/store/apps/details?id=com.project.Born&hl=en_GB

4. EREDBOOK

ERedbook is an app based digital version of the paper Redbook. It provides solutions for parents that allows them to review their child's NHS records whilst providing health information and guidance from NHS and other sources. Additionally, it contains personalised information for new parents, including relevant videos and information from local sources & reminders and appointments updates. Parents can review the NHS child's health records on the app when they connect record to NHS. Also, The NEON participants will be able to review the NEON toolkit and receive correct information via the ERedbook app at any time and place.

https://www.eredbook.org.uk

https://apps.apple.com/gb/app/eredbook/id606443658

https://play.google.com/store/apps/details?id=com.sitekit.eRedBook&hl=en_GB

OTHER SOLUTION IDEAS TO SUPPORT PLAY AND ACTIVITY:

1. Develop activity and play schedules for you and your infant with guidance from our local children centre or health visitor and make time for these activities.

2. Download the 'born to move' app for access to information and guidance on infant activity and play.

3. Designate a play area (even if this needs to be set-up and put away at times of day) for your infant in the home.

4. Arrange play and physical activity sessions together as a group, or with other parents, extended family, carers and infants – this could help with support and confidence in exercising and conducting physical activity in public spaces.

For peer review only

OTHER LOCAL INITIATIVES, CHARITIES AND PROJECTS IN EAST LONDON

1. BREASTFEEDING NETWORK

Find your nearest breast feeding drop-in-group in Tower Hamlets
https://www.breastfeedingnetwork.org.uk

There are groups run at Shadwell Children's Centre, Chrisp Children's Centre, Overland Children's Centre, Collingwood Children's Centre, Wapping Children's Centre and many more. If you need information or support with breastfeeding in the evenings or at weekends please call:

National Breastfeeding Helpline: *0300 1000 212*

BfN Support in Bengali/Sylheti: *0300 456 2421*

Open from *9.30am - 9.30pm 365 days a year*

Newham: https://www.nct.org.uk/local-activities-meet-ups/region-london/newham/breastfeeding-support

Tower Hamlets: https://www.breastfeedingnetwork.org.uk/tower-hamlets/

Waltham Forest offers a team of infant feeding practitioners delivering 1:1 sessions, a helpline and at home visitors to support the breastfeeding family through HENRY.

For information:
Email: wfsupport@henry.org.uk
Telephone: 020 8496 5223

2. DENTAL SERVICES AND RESOURCES

Dentists across Tower Hamlets, Waltham Forest, and Newham offer free dental check-ups, which you can attend as soon as your child's teeth first appear.

To find your nearest dental practice in Tower Hamlets, please call 020 7364 5000.

HENRY has put together some tips for healthy teeth in young children and a list of Waltham Forest dentists currently accepting new patients from birth: https://www.henry.org.uk/sites/www.henry.org.uk/files/inline-files/Healthy%20Teeth%20Leaflet%20%28April%29_0.pdf

The Kent Community Health NHS Foundation Trust has compiled some online oral health promotion resources for parents here: https://www.kentcht.nhs.uk/service/dental-services/oral-health-promotion-resources/

The Kent Community Health NHS Foundation Trust has compiled some online oral health promotion resources for parents here: https://www.kentcht.nhs.uk/service/dental-services/oral-health-promotion-resources/

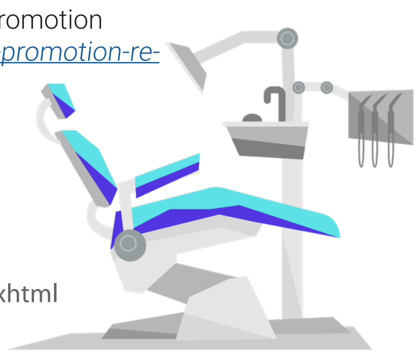

For peer review only

Here are examples of dental services by borough (to find out more, please see the community asset map):

Tower Hamlets

- Fresh Springs Dental Practice
  T: 020 3216 0077
  E: reception@freshspringsdentalpractice.co.uk

- Together Dental Whitechapel
  T: 020 7247 4600
  E: whitechapelreception@together.dental

Waltham Forest

- Abbey Dental Practice
  T: 020 8521 2816
  E: info@abbeydentalwalthamstow.co.uk

- Inspire Dental Walthamstow
  T: 020 8521 6656
  E: info@inspiredentalwalthamstow.co.uk

3. MATERNITY MATES

http://www.whfs.org.uk/index.php/what-we-do/maternity-mates

Maternity Mates™ is a project run by the charity Women's Health and Family Services which recruits, trains and match-ups volunteer Maternity Mates with pregnant women in need of extra support in Tower Hamlets. A Maternity Mate is a female volunteer trained by WHFS to provide practical and emotional support to women during pregnancy, childbirth and the early weeks of motherhood. Support can start any time from the 5th month of pregnancy, through to birth and up to either 6 or 12 weeks after the baby is born (6 weeks in Tower Hamlets and 12 weeks in Newham). **Maternity Mates** are recruited from the communities they serve, and where possible, will speak the same language as the mother-to-be.

For further information, please contact:

Jasmin Begum - Programme Manager
M: *07960 327 760*
T: *020 7377 9640*
E: *jasmin.begum@whfs.org.uk*

For information about the project in Tower Hamlets and Waltham Forest, please contact:

Rubi Rodriguez - Project Co-ordinator Tower Hamlets and Waltham Forest
M: 07496 764 881
E: rubi.rodriguez@whfs.org.uk

For information about the project in Newham, please contact:

Irantzu Perez Arribas - Project Co-ordinator Newham
M: 07436 139 235
E: irantzu.perez@whfs.org.uk

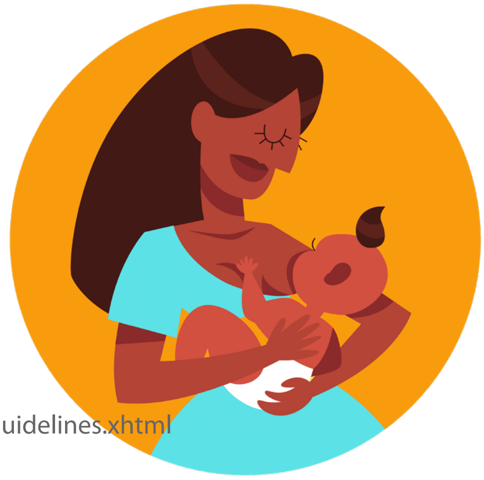

For peer review only

4. BOUNTY

https://www.bounty.com/about-bounty/bounty-packs/newborn-pack

Bounty provides mothers and pregnant women with free subscription packs including advice from Public Health England, as well as a variety of sample sized baby products (nappies, baby formula etc.) and coupons.

Every mother with a Newborn will receive a Newborn Pack at the hospital after delivery, and more freebies and advice are available through the Bounty app.

5. EMMA'S DIARY

https://www.emmasdiary.co.uk
Contact: 01628 535 483 Mon-Fri 9am to 4:30pm

Emma's Diary is a free to access resource created by a board of GPs and midwives that provide information ranging from trying to get pregnant from wellbeing information during and post-pregnancy. There's a free to download mobile app to allow you to access information more easily, tailored towards recent mothers and baby related advice and offers.

6. THE TOYHOUSE CENTRE

http://www.toyhouse.org.uk/

Phone: 0020 7987 7399
E-mail: info@toyhouse.org.uk

Toy House is a local charity in Tower Hamlets which runs projects and services aimed at support play and wellbeing of infants, children and families.

Toy Libraries
http://www.toyhouse.org.uk/toy-libraries/
The charity has its own toy library, and has information and links to over 19 toy libraries and stay and play sessions run across Tower Hamlets.

7.MEND - MIND, EXERCISE, NUTRITION...DO IT!

https://www.mytimeactive.co.uk/mend

MEND is a programme which empowers mums, children and adults to become fitter, healthier and happier and to reach or maintain a healthier weight. It's free to attend and run by local teams in local communities, after school or at weekends. MEND have a range of programmes for different ages run in different boroughs across London.

MEND Mums is designed for post-natal women with a baby up to 2 years and a BMI of 25 or above, **MEND** Mums is a fun and interactive weight management programme incorporating energy boosting workouts and great nutrition tips to help establish healthy habits for life.

For more information about any of the MEND programmes in Tower Hamlets call:
020 8323 1725

For peer review only

8. MIND

MIND is a mental health charity with a specific branch in Newham and Tower Hamlets that offers free mental health services talking therapies, COVID19 Support and Advocacy services

https://www.mithn.org.uk
Phone: 020 7510 1081
Email: info@mithn.org.uk

9. REBECCA CHEETHAM NURSERY & CHILDREN'S CENTRE

Website: https://www.rebeccacheetham.newham.sch.uk
Phone:
Email:

The Rebecca Cheetham Children's Centre offers free child-minding services as well as advice and support for families with young children. However they have temporarily stopped face to face sessions in light of the COVID pandemic. Limited online services are still running for examples: a Family Support Team that can refer you to foodbanks.

For peer review only

OTHER IDEAS AND SUGGESTIONS

- **Breakfast clubs** – these are available in most schools to give children a healthy start to the day. Speak to your local school. Due to COVID19 Restrictions, these breakfast clubs have moved to.
- Despite this, virtual coffee mornings are ongoing

- **Social support** – if you feel you need more support from other mothers, parents or people local to you, you can attend local coffee mornings, toddler groups and parent forums. E.g. One stop shop in Newham and Tower Hamlets. Father examples would be the "idea Store" which is based in Tower Hamlets and offers a range of free activities and educational services for children and families alike. http://www.healthwatchnewham.co.uk/sites/default/files/newham_domestic_abuse_one-stop-shop.pdf

- **Triple P – Positive Parenting Programme** – is an evidence based programme aimed at supporting parents develop relationships with their children.

  - Newham offers a Parenting Befriending service and bookable workshops accessible via the following website: https://families.newham.gov.uk/kb5/newham/directory/family.page?family-channel=1-1

  - Tower Hamlets offers a similar service: https://www.towerhamlets.gov.uk/lgnl/educa tion_and_learning/parental_support/parenting_programmes.aspx
    parenting@towerhamlets.gov.uk.

  - Waltham Forest offers a series of 6 sessions for parents with children aged 3 and over. https://www.walthamforest.gov.uk/content/parenting-support

    Call: *020 8496 2442*
    Email: *earlyhelpparenting@walthamforest.gov.uk*

- **Tunmarsh Centre**– support for parents with children with special needs
  The New Tunmarsh Centre based in Newham provides free education for children with ASD / DHD and support for their families. Parents are regularly invited for coffee mornings with speakers.

  E-mail: Faz.Mac@gmail.com

For peer review only - http://bmjopen.bmj.com/site/about/guidelines.xhtml

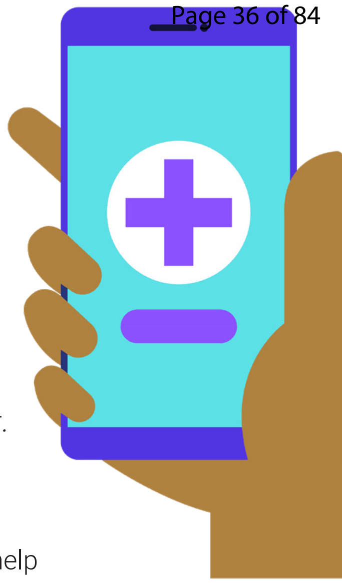

- **John Smith Children's Centre**

  Telephone: 0207 364 0537
  Contact: Deborah Wooding
  Email: deborah.wooding@towerhamlets.gov.uk
  Based in Tower Hamlets, The John Smith Children's Centre that offer childcare support.

- **The Trinity Centre**
  Website: *www.thetrinitycentre.org/home/*
  The Trinity Centre offers a day care nursery as well as independent groups and faith groups to help support parents form minority and marginalised communities

- **Around Poplar Children's Centre**

  Contact: Brenda Pascal
  T: 020 7364 0540
  E: Brenda.pascal@towerhamlets.gov.uk

  The Around Poplar Children's Centre offers childcare, family, health services, and early education support for families with children under 5 free of charge.

  For a list of similar children's centres in Tower Hamlets, see here:
  https://www.localoffertowerhamlets.co.uk/organisations?utf8=%E2%9C%93&search_organisation%5Bterm%5D=&search_organisation%5Blocation%5D=&search_organisation%5Blatitude%5D=&search_organisation%5Blongitude%5D=&search_organisation%5Bcategory_ids%5D%5B%5D=3308

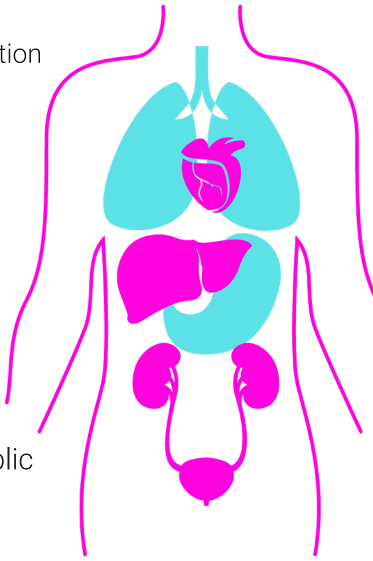

- **Idea Store**

  Website: www.ideastore.co.uk
  Branches of the Idea Store in Tower Hamlets offer library services and access to public computers for families and individuals free of charge.

- **Outreach centre** Outreach centres are open till 6 pm where you can drop your children there, and it is free for people who are entitled for public funds. They offer: Child minding, after school club, breakfast club for parents and children <3 years. Key examples would be Community Centres and Children's Centres.

EXAMPLES:

Newham Council: *https://www.newham.gov.uk/homepage/115/community-centres*
Tower Hamlets: Community and day centres (towerhamlets.gov.uk)

Plaistow Children's Centre
Website: *https://plaistow.newham.sch.uk/Plaistow-Children-s-Centre/*

The Plaistow Children's Centre is based in Newham and offers care and support for young children ranging from 0-5 years for 49 weeks within the year led by staff and volunteers. Other services range from nutrition webinars to overall family support and advice. The Children's Centre is still open during the pandemic and offers slightly modified services as a result.

For peer review only

OTHER SUPPORT

1. Request interpretation support for GP visits from Tower Hamlets GP care group.
2. Advocacy & Interpreting Service in Newham (https://www.gpcaregroup.org/section/455/Services/page/eb745e07-85ab-4cee-bc8f-c880a-d400a95/Advocacy-Interpreting)
3. Provide feedback on services or lodge a complaint via Health Watch Tower Hamlets. More information can be accessed here: https://www.healthwatchtowerhamlets.co.uk/

FOOD BANKS:

Suggested Food Bank locations: https://www.google.com/maps/d/edit?mid=1ZyqIE2-HgdQ-

NEON Community Asset Map: https://www.google.com/maps/d/edit?mid=1ZyqIE2-Hg-dQ-Hzow7KwhAiYsdtQnP_Zq&usp=sharing

- **Newham Food Alliance**

  Website: https://www.newham.gov.uk/newhamfoodalliance

  The Newham Food Alliance is a food bank based in Newham that can offer access to food for those who are struggling financially. There is also delivery support they can provide to those self-isolating due to COVID and providing food remotely during the pandemic regardless.

- **Church Food Banks:**

  Website: www.churchesfoodbank.org.uk/

  Food Banks located in the Borough of Newham. To access these you need to be referred from another agency such as a school, advice centres or your GP.

The 2 main sites are:

Woodgrange Baptist Church

345 Romford Road,Forest Gate,E7 8AA

0208 555 9880

St Paul's Church

65 Maryland Road,Stratford,E15 1JL

0208 534 1164

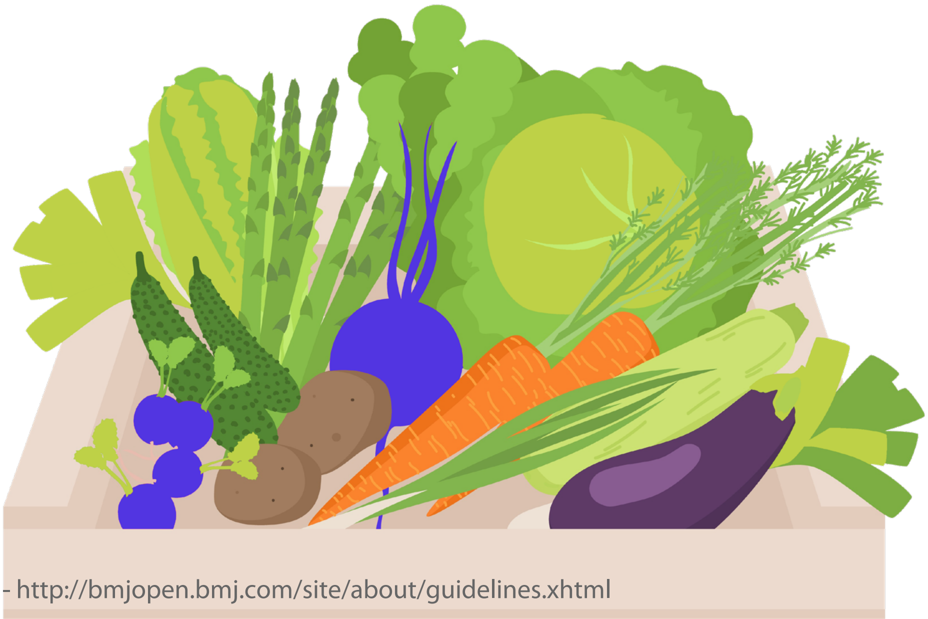

For peer review only

- **The Trussell Trust**

  Website: https://www.trusselltrust.org

  The Trussell Trust is a charity that provides nationwide network of food banks as well as emergency food for those in need. They also run regular campaigns to further increase support for people who need access to regular foods but cannot afford to do so. The website is useful as it flag foodbanks that are local to you.

- **First Love Foundation Foodbank**

  Website: www.firstlovefoundation.org.uk/

  The First Love Foundation coordinates the Tower Hamlets Foodbank. It provides daily necessities to families in need and alongside this, operates as a crisis service.

- **Eat or Heat - Waltham Forest Food Bank**

  Website: http://eatorheat.org

  Phone Number: 0800 772 0212

  Email: referrals@eatorheat.org

  A charity based in East London that supports local families. On the website, different professionals who can refer you to this service are listed.

- **Rukhsana Khan Foundation**

  Website: https://www.rukhsanakhanfoundation.org

  Phone Number: 07939 232 123

  Email: rukhsanakhanfoundation@outlook.com

  Rukshana Khan Foundation is a local charity that supports families in East London. They run food parcels and deliveries for those on benefits and those referred from Citizens Advice, NHS, Local Doctor's Surgery, Job Centre, Baby Food Bank, or the Council. They are also supporting those classed as clinically vulnerable. You can register for support on their website.

  They run a foodbank on Saturdays 11am-12pm at the William Morris Community Centre.

- **Waltham Forest Liberal Democrats**

  Website: https://www.walthamforestlibdems.org/local_support_for_families_in_need

  The Waltham Forest Liberal Democrats have collated a set of resources highlighting local foodbanks with Waltham Forest that local families can access

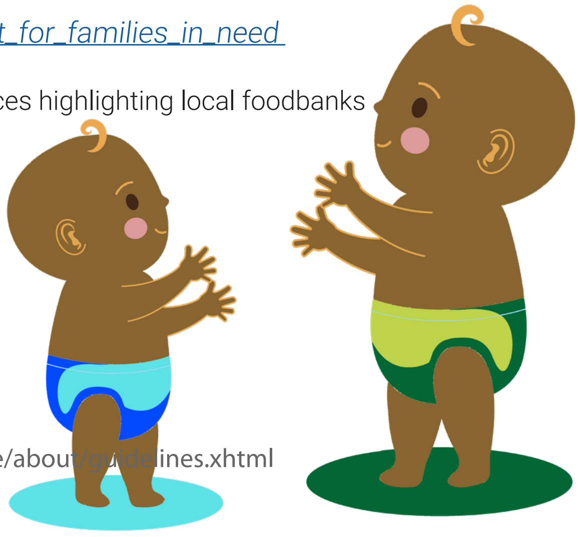

For peer review only

- **Eden Girls' School, Waltham Forest**

A local food bank based at Eden Girls' School that provides food to local people from 1-3pm every Friday

Contact Details:

Eden Girls' School, Waltham Forest

Silver Birch and Landmark Houses,

Blackhorse Lane, Walthamstow

London, E17 5SD

Tel: 0208 523 1810

Email: info@egwf.staracademies.org

- **Community Food Projects in Tower Hamlets**

https://www.wen.org.uk/2020/03/30/https-www-wen-org-uk-2020-03-30-information-on-local-response-to-covid-19/

- **Healthwatch groups available in different boroughs:**

Newham *http://www.healthwatchnewham.co.uk/*

Tower Hamlets *https://www.healthwatchtowerhamlets.co.uk/*

Redbridge *http://www.healthwatchredbridge.co.uk/*

Waltham Forest *https://www.healthwatchwalthamforest.co.uk/*

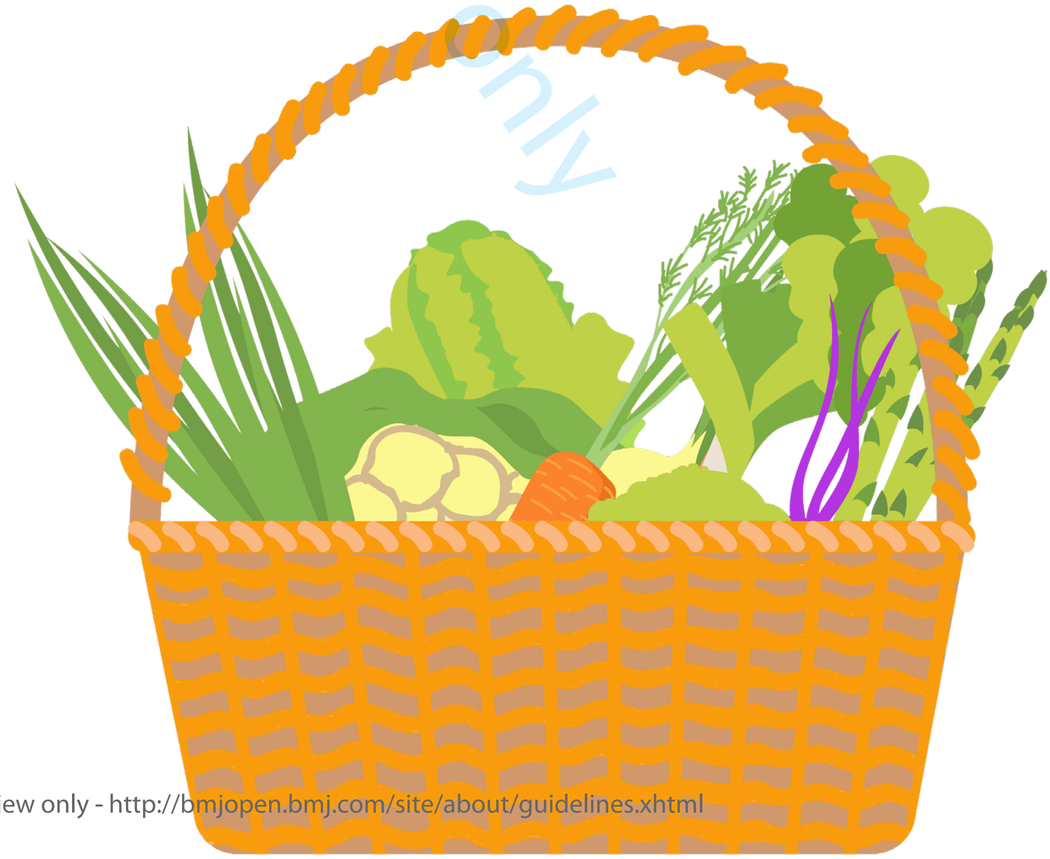

For peer review only

For peer review only - http://bmjopen.bmj.com/site/about/guidelines.xhtml

Participants Feedback Questionnaire

1. What were your reasons for wanting to attend the group meetings?

2. What made you come back after attending a meeting? /or, what was your reason for not coming back to later meetings?

i. *What made you keep coming back to the meetings? /Or, what could we have done differently to motivate you to come back to the next meeting?*

3. Did the group manage to meet your expectations?

i. *how did it meet your expectations?*
ii. *If it did not, why was this the case?*

4. What could be improved if this project was run again?

i. *How could we improve the content (meeting exercises)*
ii. *How could we improve the tools (picture cards / infant feeding resources)*
iii. *How could we improve the tailoring to your needs as British Bangladeshi mother/pregnant women/or grandmother*
iv. *Would it be beneficial to have picture cards and other project materials written in Bengali (with the picture on the front and Bengali text on the back)*
v. *Is there any additional information or resources you would have liked to have been offered in the meetings?*

5. How involved did you feel in the learning process of the group?

i. Did you feel you worked together as a group during the meetings? How so?
ii. Were there any barriers you experienced to participating?
iii. Were you given opportunities to share experiences? Please give examples.

6. Are you in contact with any of the other participants outside of the group meetings?

i. Have you discussed any issues raised in the group meetings?
ii. Have you supported each other on any issues raised in the group meeting?

9. Have you found new people, groups or sources of information as a result of attending the group meetings? Please give examples.

10. Did you share any information from the group meetings with any other members of your family or community outside of the group?

11. If we were to implement a full project using this approach, the next stage would involve more group meetings (possibly up to 8 additional meetings) where by the group would support each other to implement some of the solutions you identified and then after implementing

For peer review only - http://bmjopen.bmj.com/site/about/guidelines.xhtml

solutions, evaluate their success. Do you think this is something you or Bangladeshi carers would be willing to undertake or partake in?

i. *Can you think of any barriers to using this approach we need to consider at this stage?*
ii. *How could additional meetings best support you to implement the solutions you identified, and introduce the recommended infant feeding and care practices? What should they focus on?*
iii. *What additional meetings would be acceptable to you and feasible to attend?*

12. Is there anything else you would like to share about your experience on the project?

For peer review only

Facilitator Report Form

<table>
<tr><th colspan="6">PLA GROUP FACILITATOR REPORT - PLA group meeting xx</th></tr>
<tr><td>Group location/name (XX children’s centre group)</td><td></td><td rowspan="2">Meeting number</td><td rowspan="2"></td><td rowspan="2">Meeting date</td><td rowspan="2"></td></tr>
<tr><td>Facilitator name</td><td></td></tr>
<tr><td>Number of picture cards discussed</td><td colspan="5"></td></tr>
<tr><td>Did you manage to go through all questions on the picture cards?
● What does the picture show?
● Clarify which practice is being discussed
● Ask and explain how this affects infant growth and development
● Use info on the back of the card to ask questions about the practice and probe
● Ask if this occurs in the community? Is it common?
Were there any barriers to a full discussion on the above questions?</td><td colspan="5"></td></tr>
<tr><td>Were there any picture cards not discussed at all?</td><td colspan="5"></td></tr>
</table>

For peer review only

Which exercises were completed during your meeting?	
What was the outcome of each exercise?	
How long did the meeting run for?	
Did anything go well/did anything not go well?	
GROUP FACILITATOR TO COMPLETE AFTER EACH MEETING CONDUCTED.	

peer review only

**PLA GROUP FACILITATOR REPORT – PLA group meeting 3**					
Group location/name (XX children's centre group)		Meeting number		Meeting date	
Facilitator name					
Number and type of picture cards discussed					
Did you manage to go through all the probing questions on the picture cards?<br>● *What does the picture show?*<br>● *Clarify what barrier is being discussed*<br>● *Ask and explain how this barrier may affect or influence behaviour relating to infant feeding and care*					

• *Ask which infant feeding and care practice the barrier may prevent parents from practicing?*<br><br>• *Ask if the barrier exists in the community*<br>Were there any barriers to a full discussion on the above questions?	
Were there any picture cards not discussed at all?	
Which exercises were completed during your meeting?	
What was the outcome of each exercise?<br><br>Which barrier cards were identified for prioritised practice 1?<br><br>Which barrier cards were identified for prioritised practice 2?<br><br>Which barrier cards were identified for prioritised practice 3?	
How long did the meeting run for?	
Did anything go well/did anything not go well?	
GROUP FACILITATOR TO COMPLETE AFTER EACH MEETING CONDUCTED.	

For peer review only - http://bmjopen.bmj.com/site/about/guidelines.xhtml

**PLA GROUP FACILITATOR REPORT – PLA group meeting 4**					
Group location/name (XX children's centre group)		Meeting number		Meeting date	
Facilitator name					
Number and type of picture cards discussed					
Did you manage to go through all the probing questions on the picture cards?<br>● *What does the picture show?*<br>● *Ask participants to identify what solution they are discussing and what non-recommended infant feeding and care practice the solution helps to reduce or manage.*<br>● *Ask participants to suggest ideas on what activities the group (or they, as individuals) could undertake to achieve this solution. Use the information on the back of the card to suggest ideas on activities which could be undertaken. Ask if the barrier exists in the community*<br>● Were there any barriers to a full discussion on the above questions?					
Were there any picture cards not discussed at all?					
Which exercises were completed during your meeting?					

For peer review only

For peer review only - http://bmjopen.bmj.com/site/about/guidelines.xhtml

What was the outcome of each exercise?<br><br>Which barrier solution cards were identified for prioritised practice 1<br><br>Which management solution cards were identified for prioritised practice 1<br><br>Which barrier solution cards were identified for prioritised practice 2<br><br>Which management solution cards were identified for prioritised practice 2<br><br>Which barrier solution cards were identified for prioritised practice 2<br><br>Which management solution cards were identified for prioritised practice 2	
How long did the meeting run for?	
Did anything go well/did anything not go well?	
GROUP FACILITATOR TO COMPLETE AFTER EACH MEETING CONDUCTED.	

For peer review only

Sustainability assessment

1. PARTICIPATION

Criteria for assessment	*Are many people participating? Are they active? Is anyone excluded from participation?*
Score / Average score assigned	
Identified successes	
Identified concerns / areas for improvement	
Actions agreed	
Additional notes	

2. LEADERSHIP

Criteria for assessment	*Do groups have leaders, are they helping or hindering the group? Do they have the capacity to lead groups well?*
Score / Average score assigned	
Identified successes	
Identified concerns / areas for improvement	
Actions agreed	
Additional notes	

For peer review only - http://bmjopen.bmj.com/site/about/guidelines.xhtml

WOMEN AND CHILDREN FIRST (UK)

3. ORGANISATIONAL STRUCTURE

Criteria for assessment	*Are the organisational structures, including the committee and taskforces, set up in the groups? If so, what is their purpose and how are they functioning?*
Score / Average score assigned	
Identified successes	
Identified concerns / areas for improvement	
Actions agreed	
Additional notes	

4. NEEDS / PROBLEM ASSESSMENT

Criteria for assessment	*Are the problems identified in the groups important and relevant to their respective community?*
Score / Average score assigned	
Identified successes	
Identified concerns / areas for improvement	
Actions agreed	
Additional notes	

5. RESOURCE MOBILISATION

Criteria for assessment	***Are the groups able to mobilise resources to implement their solutions? Are they able to mobilise resources from within the groups and/or outside the groups?***
Score / Average score assigned	
Identified successes	
Identified concerns / areas for improvement	
Actions agreed	
Additional notes	

6. ABILITY TO ASK "WHY"?

Criteria for assessment	***Do groups try to address root causes of the problems they face? If so, how well? By root causes we are referring to meeting re: contributing factors – the broader causes of ill health such as poverty.***
Score / Average score assigned	
Identified successes	
Identified concerns / areas for improvement	
Actions agreed	
Additional notes	

For peer review only - http://bmjopen.bmj.com/site/about/guidelines.xhtml

WOMEN AND CHILDREN FIRST (UK)

7. LINKS TO OTHER ORGANISATIONS / PEOPLE

Criteria for assessment	*Are they linking to other stakeholders (people, groups, organisations) who can help them solve identified issues? If so, who are they linking with and why?*
Score / Average score assigned	
Identified successes	
Identified concerns / areas for improvement	
Actions agreed	
Additional notes	

8. RELATIONSHIP WITH OUTSIDE AGENT

Criteria for assessment	*Is the external agent – Ministry of Health counterparts – involved and supporting the groups, building their capacity?*
Score / Average score assigned	
Identified successes	
Identified concerns / areas for improvement	
Actions agreed	
Additional notes	

For peer review only - http://bmjopen.bmj.com/site/about/guidelines.xhtml

WOMEN AND CHILDREN FIRST (UK)

9. PROGRAMME MANAGEMENT

**Criteria for assessment**	***Do groups feel they own the groups and their work? Are they capable of continuing the activities once the project comes to an end? Is the NGO implementing partner building group capacity to solve health problems and strengthening them to be sustainable?***
**Score / Average score assigned**	
**Identified successes**	
**Identified concerns / areas for improvement**	
**Actions agreed**	
**Additional notes**	

For peer review only - http://bmjopen.bmj.com/site/about/guidelines.xhtml

Direct observation of intervention delivery and CF performance Form:

Did the facilitator deliver the components of the sessions according to the manual?
(Please rate this on scale of 1-4 scale i.e. 1 = no elements of the programme delivered;
2 = some elements of the programme, others missed or inserted;
3 = majority elements of the programme delivered;
4 = all elements of the programme delivered as per handbook).

What are your thoughts about the overall session?

Did you think there are areas for improvement and what where they?

Would you have done anything differently while facilitating the sessions and how?

What advice do you have for the community facilitator?

For peer review only

For peer review only - http://bmjopen.bmj.com/site/about/guidelines.xhtml

Development performance of children – (ASQ-3)

Improving lives

NHS
Oxleas
NHS Foundation Trust

Parents of Shiv LOGAN
3 Abbey Terrace
Abbey Wood
London
SE2 9EY

380 D

Oxleas 0 – 19 Years Public Health Nursing Service
Central Administration Team
Tel: 020 8836 8621
Email: oxl-tr.cat@nhs.net

11 Nov 2019

Dear Parents of Shiv,

9 – 12 month review Appointment confirmation

This letter is to confirm your appointment to review your child's development.

At: Gallions Reach Health Centre Bentham Road Thamesmead London SE28 8BE

On: Tuesday 10 Dec 2019 10:30

Enclosed with this letter are the 'Ages and Stages' questionnaires that are used for this developmental review. Please complete these forms and try out the activities with your baby before the appointment.

It is important that you complete the questionnaires with your child before your appointment and bring the completed questionnaires and Red Book with you on the day.

If you require any help completing this questionnaire or you have any queries about your appointment please contact us on **020 8836 8621**

To find out about Healthy Start benefits search online for the Healthy Start website

Visit our website which was designed by Oxleas Health Care professionals, service users and families to provide you with information about your health and wellbeing and how to access our services.

www.Oxleas.nhs.uk/CYP

Follow us on Twitter @oxleasCYPGre

There is a drop in Health Advice Session open every day from Monday to Friday across Greenwich. For times and venues see Health Visitor Timetable in the link above.

Yours sincerely
Public Health Nursing Service 0 -19 Years

For peer review only - http://bmjopen.bmj.com/site/about/guidelines.xhtml

ASQ-3 **10 Month Questionnaire** *page 3 of 6*

GROSS MOTOR *(continued)*

		YES	SOMETIMES	NOT YET	
3. When you stand your baby next to furniture or the cot rail, does she hold on without leaning her chest against the furniture for support?	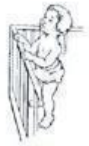	○	○	○	___
4. While holding onto furniture, does your baby bend down and pick up a toy from the floor and then return to a standing position?	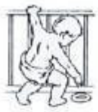	○	○	○	___
5. While holding onto furniture, does your baby lower himself with control (without falling or flopping down)?		○	○	○	___
6. Does your baby walk beside furniture while holding on with only one hand?		○	○	○	___
			GROSS MOTOR TOTAL		___

FINE MOTOR

		YES	SOMETIMES	NOT YET	
1. Does your baby pick up a small toy with only one hand?	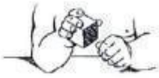	○	○	○	___
2. Does your baby *successfully* pick up a small cube of bread by using her thumb and all of her fingers in a raking motion? (*If she already picks up a small cube of bread, mark "yes" for this item.*)	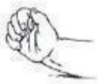	○	○	○	___
3. Does your baby pick up a small toy with the *tips* of his thumb and fingers? (*You should see a space between the toy and his palm.*)	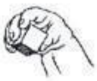	○	○	○	___
4. After one or two tries, does your baby pick up a piece of string with her first finger and thumb? (*The string may be attached to a toy.*)	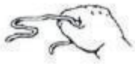	○	○	○	___
5. Does your baby pick up a small cube of bread with the tips of his thumb and a finger? (*He may rest his arm or hand on the table while doing it.*)	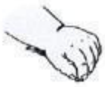	○	○	○	___ *
6. Does your baby put a small toy down, without dropping it, and then take her hand off the toy?		○	○	○	___
			FINE MOTOR TOTAL		___

* If Fine Motor Item 5 is marked "yes" or "sometimes," mark Fine Motor Item 2 "yes."

BE Av. 1 1.15

Ages & Stages Questionnaires®, Third Edition (ASQ-3™), Squires & Bricker
© 2009-2015 Paul H. Brookes Publishing Co. All rights reserved.
Adaptation into British English prepared with the Department of Health.

ASQ-3 **10 Month Questionnaire** *page 5 of 6*

OVERALL

Parents and providers may use the space below for additional comments.

1. Does your baby use both hands and both legs equally well? If no, explain: ○ YES ○ NO

2. When you help your baby stand, are his feet flat on the surface most of the time? If no, explain: ○ YES ○ NO

3. Do you have concerns that your baby is too quiet or does not make sounds like other babies? If yes, explain: ○ YES ○ NO

4. Does either parent have a family history of childhood deafness or hearing problems? If yes, explain: ○ YES ○ NO

5. Do you have concerns about your baby's eyesight? If yes, explain: ○ YES ○ NO

6. Has your baby had any medical or health-related problems in the last few months? If yes, explain: ○ YES ○ NO

BE Av. 1 1.15

Ages & Stages Questionnaires®, Third Edition (ASQ-3™), Squires & Bricker
© 2009-2015 Paul H. Brookes Publishing Co. All rights reserved.
Adaptation into British English prepared with the Department of Health.

For peer review only - http://bmjopen.bmj.com/site/about/guidelines.xhtml

4-day Food Diary

DAY 1

Day 1:		Date:		
**Time**	**Where? With Whom? TV on? At table?**	**Food/Drink description & preparation**	**Brand Name**	**Portion size or quantity eaten**
How to describe what you had and how much you had can be found on pages 16 - 21				
6am to 9am				
9am to 12 noon				

Time	Where? With Whom? TV on? At table?	Food/Drink description & preparation	Brand Name	Portion size or quantity eaten
12 noon to 2pm				
2pm to 5pm				

For peer review only - http://bmjopen.bmj.com/site/about/guidelines.xhtml

Time	Where?<br>With Whom?<br>TV on?<br>At table?	Food/Drink description & preparation	Brand Name	Portion size or quantity eaten
		5pm to 8pm		
		8pm to 10pm		
		10pm to 6am		

Was the amount of **food** that you had today about what you usually have, less than usual, or more than usual?

Yes, usual ☐ No, **less** than usual ☐ No, **more** than usual ☐

Please tell us why you had less than usual

Please tell us why you had more than usual

Was the amount you had to **drink** today, including water, tea, coffee and soft drinks [and alcohol], about what you usually have, less than usual, or more than usual?

Yes, usual ☐ No, **less** than usual ☐ No, **more** than usual ☐

Please tell us why you had less than usual

Please tell us why you had more than usual

Did you **finish all the food and drink** that you recorded in the diary today?

Yes ☐ No ☐

If no, please **go back to the diary and make a note of any leftovers**

Did you take any **vitamins, minerals or other food supplements** today?

Yes ☐ No ☐

If yes, **please describe the supplements you took below**

Brand	Name (in full) including strength	Number of pills, capsules, teaspoons

Please record on the next pages details of any recipes or (if not already described) ingredients of made up dishes or take-away dishes.

Write in recipes or ingredients of made up dishes or take-away dishes			
NAME OF DISH:		***Serves:***	
Ingredients	***Amount***	***Ingredients***	***Amount***
Brief description of cooking method			

For peer review only - http://bmjopen.bmj.com/site/about/guidelines.xhtml

Write in recipes or ingredients of made up dishes or take-away dishes			
NAME OF DISH:		***Serves:***	
Ingredients	***Amount***	***Ingredients***	***Amount***
Brief description of cooking method			

DAY 2

Day 2:		Date:		
Time	Where? With Whom? TV on? At table?	Food/Drink description & preparation	Brand Name	Portion size or quantity eaten
How to describe what you had and how much you had can be found on pages 16 - 21				
6am to 9am				
9am to 12 noon				

For peer review only - http://bmjopen.bmj.com/site/about/guidelines.xhtml

Time	Where?<br>With Whom?<br>TV on?<br>At table?	Food/Drink description & preparation	Brand Name	Portion size or quantity eaten
		12 noon to 2pm		
		2pm to 5pm		

Time	Where?<br>With Whom?<br>TV on?<br>At table?	Food/Drink description & preparation	Brand Name	Portion size or quantity eaten
		5pm to 8pm		
		8pm to 10pm		
		10pm to 6am		

For peer review only - http://bmjopen.bmj.com/site/about/guidelines.xhtml

Was the amount of **food** that you had today about what you usually have, less than usual, or more than usual?

Yes, usual ☐ No, **less** than usual ☐ No, **more** than usual ☐

Please tell us why you had less than usual

Please tell us why you had more than usual

Was the amount you had to **drink** today, including water, tea, coffee and soft drinks [and alcohol], about what you usually have, less than usual, or more than usual?

Yes, usual ☐ No, **less** than usual ☐ No, **more** than usual ☐

Please tell us why you had less than usual

Please tell us why you had more than usual

Did you **finish all the food and drink** that you recorded in the diary today?

Yes ☐ No ☐

If no, please **go back to the diary and make a note of any leftovers**

Did you take any **vitamins, minerals or other food supplements** today?

Yes ☐ No ☐

If yes, **please describe the supplements you took below**

Brand	Name (in full) including strength	Number of pills, capsules, teaspoons

Please record on the next pages details of any recipes or (if not already described) ingredients of made up dishes or take-away dishes.

For peer review only - http://bmjopen.bmj.com/site/about/guidelines.xhtml

Write in recipes or ingredients of made up dishes or take-away dishes			
NAME OF DISH:		***Serves:***	
Ingredients	***Amount***	***Ingredients***	***Amount***
Brief description of cooking method			

Write in recipes or ingredients of made up dishes or take-away dishes			
NAME OF DISH:		***Serves:***	
Ingredients	***Amount***	***Ingredients***	***Amount***
Brief description of cooking method			

DAY 3

Day 3:		Date:		
Time	Where? With Whom? TV on? At table?	Food/Drink description & preparation	Brand Name	Portion size or quantity eaten
How to describe what you had and how much you had can be found on pages 16 - 21				
6am to 9am				
9am to 12 noon				

Time	Where? With Whom? TV on? At table?	Food/Drink description & preparation	Brand Name	Portion size or quantity eaten
12 noon to 2pm				
2pm to 5pm				

For peer review only - http://bmjopen.bmj.com/site/about/guidelines.xhtml

Time	Where?<br>With Whom?<br>TV on?<br>At table?	Food/Drink description & preparation	Brand Name	Portion size or quantity eaten
		5pm to 8pm		
		8pm to 10pm		
		10pm to 6am		

Was the amount of **food** that you had today about what you usually have, less than usual, or more than usual?

Yes, usual ☐ No, **less** than usual ☐ No, **more** than usual ☐

Please tell us why you had less than usual

Please tell us why you had more than usual

Was the amount you had to **drink** today, including water, tea, coffee and soft drinks [and alcohol], about what you usually have, less than usual, or more than usual?

Yes, usual ☐ No, **less** than usual ☐ No, **more** than usual ☐

Please tell us why you had less than usual

Please tell us why you had more than usual

For peer review only - http://bmjopen.bmj.com/site/about/guidelines.xhtml

Did you **finish all the food and drink** that you recorded in the diary today?

Yes ☐ No ☐

If no, please **go back to the diary and make a note of any leftovers**

Did you take any **vitamins, minerals or other food supplements** today?

Yes ☐ No ☐

If yes, **please describe the supplements you took below**

**Brand**	**Name (in full) including strength**	**Number of pills, capsules, teaspoons**

Please record on the next pages details of any recipes or (if not already described) ingredients of made up dishes or take-away dishes.

**Write in recipes or ingredients of made up dishes or take-away dishes**			
NAME OF DISH:		***Serves:***	
Ingredients	***Amount***	***Ingredients***	***Amount***
Brief description of cooking method			

For peer review only - http://bmjopen.bmj.com/site/about/guidelines.xhtml

Write in recipes or ingredients of made up dishes or take-away dishes			
NAME OF DISH:		***Serves:***	
Ingredients	***Amount***	***Ingredients***	***Amount***
Brief description of cooking method			

DAY 4

Please remember to complete the general questions on pages 61-66!

Day 4:		Date:		
Time	Where?<br>With Whom?<br>TV on?<br>At table?	Food/Drink description & preparation	Brand Name	Portion size or quantity eaten
How to describe what you had and how much you had can be found on pages 16 - 21				
6am to 9am				
9am to 12 noon				

For peer review only - http://bmjopen.bmj.com/site/about/guidelines.xhtml

Time	Where?<br>With Whom?<br>TV on?<br>At table?	Food/Drink description & preparation	Brand Name	Portion size or quantity eaten
		12 noon to 2pm		
		2pm to 5pm		

Time	Where?<br>With Whom?<br>TV on?<br>At table?	Food/Drink description & preparation	Brand Name	Portion size or quantity eaten
		5pm to 8pm		
		8pm to 10pm		
		10pm to 6am		

Was the amount of **food** that you had today about what you usually have, less than usual, or more than usual?

Yes, usual ☐ No, **less** than usual ☐ No, **more** than usual ☐

Please tell us why you had less than usual

Please tell us why you had more than usual

Was the amount you had to **drink** today, including water, tea, coffee and soft drinks [and alcohol], about what you usually have, less than usual, or more than usual?

Yes, usual ☐ No, **less** than usual ☐ No, **more** than usual ☐

Please tell us why you had less than usual

Please tell us why you had more than usual

Did you **finish all the food and drink** that you recorded in the diary today?

Yes ☐ No ☐

If no, please **go back to the diary and make a note of any leftovers**

Did you take any **vitamins, minerals or other food supplements** today?

Yes ☐ No ☐

If yes, **please describe the supplements you took below**

Brand	Name (in full) including strength	Number of pills, capsules, teaspoons

Please record on the next pages details of any recipes or (if not already described) ingredients of made up dishes or take-away dishes.

Write in recipes or ingredients of made up dishes or take-away dishes			
NAME OF DISH:		***Serves:***	
Ingredients	***Amount***	***Ingredients***	***Amount***
Brief description of cooking method			

Write in recipes or ingredients of made up dishes or take-away dishes			
NAME OF DISH:		***Serves:***	
Ingredients	***Amount***	***Ingredients***	***Amount***
Brief description of cooking method			

For peer review only - http://bmjopen.bmj.com/site/about/guidelines.xhtml

General questions about your food/ drink during the recording period.

Special diet

1. Did you follow a special diet during the recording period e.g. vegetarian, cholesterol lowering, weight reducing?

Yes ☐ *Please specify* No ☐

Milk

2. Which type of milk did you use most often during the recording period?

Whole, fresh, pasteurised ☐ Semi-skimmed fresh, pasteurised ☐ Skimmed (fat free) fresh, pasteurised ☐ 1% fat milk, pasteurised ☐

Dried ☐ *Type* Soya ☐ *Type*

Other ☐ *Type* Did not use ☐

Tea and coffee

3. How much milk did you usually have in coffee/ tea?

Coffee A lot ☐ Some ☐ A little ☐ None/did not drink ☐

Tea A lot ☐ Some ☐ A little ☐ None/did not drink ☐

4. Did you usually sweeten your coffee/ tea with sugar?

Coffee Yes ☐ How many teaspoons in a mug/cup? ☐ No/did not drink ☐

Tea Yes ☐ How many teaspoons in a mug/cup? ☐ No/did not drink ☐

5. Did you usually sweeten your coffee/ tea with artificial sweetener?

Coffee Yes ☐ How many tablets or teaspoons in a mug/cup? ☐ No/did not drink ☐

Tea Yes ☐ How many tablets or teaspoons in a mug/cup? ☐ No/did not drink ☐

6. Did you drink decaffeinated coffee/ tea during the recording period?

Coffee Always ☐ Sometimes ☐ Never ☐

Tea Always ☐ Sometimes ☐ Never ☐

For peer review only - http://bmjopen.bmj.com/site/about/guidelines.xhtml

Breakfast cereals

7. How much milk did you usually have on breakfast cereal?

Drowned ☐ Average ☐ Damp ☐ None/did not eat ☐

8. How did you usually make your porridge?

With all water ☐ With all milk ☐ With milk and water ☐ Did not eat ☐

9. Did you usually sweeten or salt your porridge?

With sugar ☐ With honey ☐ With salt ☐ Neither/did not eat ☐

10. How did you usually make your instant oat cereal?

With all water ☐ With all milk ☐ With milk and water ☐ Did not eat ☐

11. Did you usually sweeten or salt your instant oat cereal?

With sugar ☐ With honey ☐ With salt ☐ Neither/did not eat ☐

Fats for spreading and cooking

12. Which type of butter, margarine or other fat spread did you use most often during the recording period? Please record the full product name and fat content

Name:

None ☐

e.g. Flora Omega 3 plus, low fat spread, 38% fat, polyunsaturated

13. How thickly did you spread butter, margarine on bread, crackers etc?

Thick ☐ Medium ☐ Thin ☐ N/A ☐

14. Which type of cooking fat/oil did your household use most often over the recording period? Please record the full product name e.g. *Sainsbury's sunflower oil*

Name:

None ☐

Bread

15. Which type of bread did you eat most often during the recording period?

White ☐ Granary ☐ Wholemeal ☐ Brown ☐

50/50 bread e.g. Hovis Best of Both ☐ Other ☐ *Type* Did not eat ☐

16. Was it a large loaf or a small loaf?

Large ☐ Small ☐

For peer review only - http://bmjopen.bmj.com/site/about/guidelines.xhtml

17. If the bread was shop bought, how was it sliced?

Thick ☐ Medium ☐ Thin ☐ Unsliced ☐ N/A ☐

Meat

18. If you ate meat during the recording period, did you eat the visible fat?

Always ☐ Sometimes ☐ Never ☐ Did not eat meat ☐

19. If you ate poultry (e.g. chicken, turkey) during the recording period, did you eat the skin?

Always ☐ Sometimes ☐ Never ☐ Did not eat poultry ☐

Fruit and vegetables

20. If you ate apples during the recording period, did you eat the skin?

Always ☐ Sometimes ☐ Never ☐ Did not eat ☐

21. If you ate pears during the recording period, did you eat the skin?

Always ☐ Sometimes ☐ Never ☐ Did not eat ☐

22. If you ate new potatoes during the recording period, did you eat the skin?

Always ☐ Sometimes ☐ Never ☐ Did not eat ☐

23. If you ate baked/jacket potatoes during the recording period, did you eat the skin?

Always ☐ Sometimes ☐ Never ☐ Did not eat ☐

Salt

24. Do you add salt to your food at the table?

Always ☐ Sometimes ☐ Never ☐

25. Do you add salt substitute to your food at the table? *e.g. LoSalt*

Always ☐ Sometimes ☐ Never ☐

Water

26. Which type of water did you drink most often during the recording period?

Tap ☐ Filtered ☐ Bottled ☐ *brand* ______ Did not drink ☐

Thank you for completing this diary.

For peer review only - http://bmjopen.bmj.com/site/about/guidelines.xhtml

Children Feeding Behaviour

ID:

Child Eating Behaviour Questionnaire (CEBQ)

Please read the following statements and tick the boxes most appropriate to your child's eating behaviour.

	Never	Rarely	Sometimes	Often	Always
My child loves food	☐	☐	☐	☐	☐
My child eats more when worried	☐	☐	☐	☐	☐
My child has a big appetite	☐	☐	☐	☐	☐
My child finishes his/her meal quickly	☐	☐	☐	☐	☐
My child is interested in food	☐	☐	☐	☐	☐
My child is always asking for a drink	☐	☐	☐	☐	☐
My child refuses new foods at first	☐	☐	☐	☐	☐
My child eats slowly	☐	☐	☐	☐	☐
My child eats less when angry	☐	☐	☐	☐	☐
My child enjoys tasting new foods	☐	☐	☐	☐	☐
My child eats less when s/he is tired	☐	☐	☐	☐	☐
My child is always asking for food	☐	☐	☐	☐	☐
My child eats more when annoyed	☐	☐	☐	☐	☐
If allowed to, my child would eat too much	☐	☐	☐	☐	☐
My child eats more when anxious	☐	☐	☐	☐	☐
My child enjoys a wide variety of foods	☐	☐	☐	☐	☐
My child leaves food on his/her plate at the end of a meal	☐	☐	☐	☐	☐
My child takes more than 30 minutes to finish a meal	☐	☐	☐	☐	☐

For peer review only - http://bmjopen.bmj.com/site/about/guidelines.xhtml

	Never	Rarely	Some-times	Often	Always
Given the choice, my child would eat most of the time	☐	☐	☐	☐	☐
My child looks forward to mealtimes	☐	☐	☐	☐	☐
My child gets full before his/her meal is finished	☐	☐	☐	☐	☐
My child enjoys eating	☐	☐	☐	☐	☐
My child eats more when she is happy	☐	☐	☐	☐	☐
My child is difficult to please with meals	☐	☐	☐	☐	☐
My child eats less when upset	☐	☐	☐	☐	☐
My child gets full up easily	☐	☐	☐	☐	☐
My child eats more when s/he has nothing else to do	☐	☐	☐	☐	☐
Even if my child is full up s/he finds room to eat his/her favourite food	☐	☐	☐	☐	☐
If given the chance, my child would drink continuously throughout the day	☐	☐	☐	☐	☐
My child cannot eat a meal if s/he has had a snack just before	☐	☐	☐	☐	☐
If given the chance, my child would always be having a drink	☐	☐	☐	☐	☐
My child is interested in tasting food s/he hasn't tasted before	☐	☐	☐	☐	☐
My child decides that s/he doesn't like a food, even without tasting it	☐	☐	☐	☐	☐
If given the chance, my child would always have food in his/her mouth	☐	☐	☐	☐	☐
My child eats more and more slowly during the course of a meal	☐	☐	☐	☐	☐

For peer review only - http://bmjopen.bmj.com/site/about/guidelines.xhtml

Parental Feeding Style

Parental Feeding Style Questionnaire

Please read the following statements and tick the appropriate boxes to show how you deal with feeding your child. It is important to remember that there are no right or wrong answers to these questions, we are interested in what parents really feel and do.

ID

	Never	Rarely	Some-times	Often	Always
I allow my child to choose which foods to have for meals	☐	☐	☐	☐	☐
I give my child something to eat to make him/her feel better when s/he is feeling upset	☐	☐	☐	☐	☐
I encourage my child to look forward to the meal	☐	☐	☐	☐	☐
I praise my child if s/he eats what I give him/her	☐	☐	☐	☐	☐
I decide how many snacks my child should have	☐	☐	☐	☐	☐
I encourage my child to eat a wide variety of foods	☐	☐	☐	☐	☐
In order to get my child to behave him/herself I promise him/her something to eat	☐	☐	☐	☐	☐
I present food in an attractive way to my child	☐	☐	☐	☐	☐
If my child misbehaves I withhold his/her favourite food	☐	☐	☐	☐	☐
I encourage my child to taste each of the foods I serve at mealtimes	☐	☐	☐	☐	☐
I allow my child to wander around during a meal	☐	☐	☐	☐	☐
I encourage my child to try foods that s/he hasn't tasted before	☐	☐	☐	☐	☐
I give my child something to eat to make him/her feel better when s/he has been hurt	☐	☐	☐	☐	☐
I let my child decide when s/he would like to have her meal	☐	☐	☐	☐	☐
I give my child something to eat if s/he is feeling bored	☐	☐	☐	☐	☐
I allow my child to decide when s/he has had enough snacks to eat	☐	☐	☐	☐	☐
I decide when it is time for my child to have a snack	☐	☐	☐	☐	☐
I use puddings as a bribe to get my child to eat his/her main course	☐	☐	☐	☐	☐
I encourage my child to enjoy his/her food	☐	☐	☐	☐	☐

PTO

For peer review only - http://bmjopen.bmj.com/site/about/guidelines.xhtml

Network Diffusion Form

(To be completed by participants at the end of each PLA session and at 6 months from baseline)

1. How many people did you share your material/information from the PLA sessions with?..

2. How did you share this information/material (verbal, social media, whatsapp etc)?...

3. Please fill the table below with details of the people you shared the information with -

Serial number	Relation to you	Age/Gender	Family size	How did you share (verbal, social media, whatsapp)

For peer review only - http://bmjopen.bmj.com/site/about/guidelines.xhtml

Equality Impact Assessment

Equality Impact Assessment- NEON

Question	Response
**1. Name of intervention being assessed**	NEON Intervention (PLA Cycle)
**2. Summary of aims and objectives of the intervention**	To improve infant feeding, care and dental hygiene practices amongst the South Asian Communities of Tower Hamlets, Newham, and Waltham Forest
**3. What involvement and consultation has been done in relation to this intervention?** *(e.g., with relevant groups and stakeholders)*	
**4. Who is affected by the intervention?**	Infants (aged<24 months)<br>Mothers<br>Pregnant women<br>Carers- Grandmother etc.
**5. What are the arrangements for monitoring and reviewing the actual impact of the intervention?**	

Protected Characteristic Group	Is there a potential for positive or negative impact?	Please explain and give examples of any evidence/data used	Action to address negative impact (e.g. adjustment to the intervention)
**Disability**			
**Gender reassignment**			
**Marriage or civil partnership**			
**Pregnancy and maternity**			
**Race**			
**Religion or belief**			
**Sexual orientation**			
**Sex (gender)**			
**Age**			

For peer review only - http://bmjopen.bmj.com/site/about/guidelines.xhtml

Evaluation:

Question	Explanation / justification	
Is it possible the proposed intervention could discriminate or unfairly disadvantage people?		
**Final Decision:**	**Tick the relevant box**	**Include any explanation / justification required**
1. No barriers identified, therefore activity will **proceed**.		
2. You can decide to **stop** the intervention at some point because the data shows bias towards one or more groups		
3. You can **adapt or change** the intervention in a way which you think will eliminate the bias		
4. Barriers and impact identified, however having considered all available options carefully, there appear to be no other proportionate ways to achieve the aim of the intervention (e.g., in extreme cases or where positive action is taken). Therefore, you are going to **proceed with caution** with this intervention knowing that it may favour some people less than others, providing justification for this decision.		

**Date completed:**	
**Completed by whom (Borough/individual):**	

Staff time questionnaire

Measurement of Time Use for staff involved in *NEON*

This questionnaire is for collecting information on how staff from the study team who are contributing to the Nurture Early for Optimal Nutrition (*NEON*) divide their time between the different trial components. We aim to collect this information from all our partners staff involved in *NEON* over the duration of the 3-year of the study.

The information you provide will only be used for research purposes only. It ***will not*** be used to monitor performance or to plan future activities. The information you provide will help us to divide the total *NEON* cost into the following components: intervention, M&E, process evaluation, and research. This will allow us to report the cost effectiveness of the *NEON* intervention. We also want to separate out the setting up cost from the implementation cost, to inform others who may wish to implement these interventions.

Your name will not be used in the analysis but you will be identifiable through your answers. If you do not want to proceed, or wish to stop at any time, you can do so. If you have any questions, please discuss with Shereen Al Laham (*s.laham@ucl.ac.uk*).

==

Name of Staff Member: ....

Job title / role: ....

Institution: ....

Date: ....

---

1. When did your involvement with *NEON* first begin? .... (Month/Year)
2. Since you started, have you been involved in any project other than *NEON*?
   - ☐ Yes
   - ☐ No. **Go to question 6.**
3. If Yes, how many days did you spend on *NEON* in the last 30 days?
   .... Days OR .... Percent of time
4. Was this a typical month for you, since you started work on *NEON*?
   - ☐ Yes. **Go to question 6.**
   - ☐ No
5. If No, please tell me how you have divided your time between *NEON* and any other project since you first started on *NEON*.
   ....

....

(E.g: On average, 2 days per week from January to May, then full-time since June. Answer can be in percentage, days, or weeks – whatever you think most accurately represents your actual time use.)

6. Thinking now about the **last 30 days**, and only those days you worked on *NEON*, how have you in practice divided your time between the following *NEON* activities?

a) I spent .... days OR .... percent time on Monitoring and Evaluation.
b) I spent .... days OR .... percent time on study delivery and implementation.
c) I spent .... days OR .... percent time on Research Work, that is, working on the study design, reading the literature, or writing up or presenting the research findings.
d) I spent .... days OR .... percent time on Joint Work, that is, it is not possible to say it was one of the above. For example, general management or support functions.

7. Is this how you usually divide your time?

☐ Yes. **Go to question 9.**

☐ No

8. If No, please tell me how you have divided your time between the different *NEON* activities since you first started on *NEON*. *(Use the categories in question 6.)*

....

....

....

(E.g: 50% Joint work for the first 6 months, then 1 day per week Joint work but otherwise only NEON. Answer can be in percentage, days, or weeks – whatever you think most accurately represents your actual time use.)

9. Finally, I want to ask you about the division of time between **setting up** and **implementation** of the *NEON* study. Examples of setting up activities are: recruiting and training; developing and testing the intervention material. These activities may still be ongoing.
Please could you tell us when you start to work on implementation, and do setting up activities still continue

	**Activity**	**Start date of implementation** *(Month/Year)*	**Time spend on SETTING UP activities since start date** *(days per week or percentage of time)*
a	NEON intervention implementation		
b	Monitoring and Evaluation implementation		
c	Process Evaluation implementation		

== Thank You ==

SPIRIT

STANDARD PROTOCOL ITEMS: RECOMMENDATIONS FOR INTERVENTIONAL TRIALS

SPIRIT 2013 Checklist: Recommended items to address in a clinical trial protocol and related documents*

Section/ item	Item No	Description	Page
**Administrative information**			
Title	1	Descriptive title identifying the study design, population, interventions, and, if applicable, trial acronym	1
Trial registration	2a	Trial identifier and registry name. If not yet registered, name of intended registry	3, 17, 18
	2b	All items from the World Health Organization Trial Registration Data Set	N/A
Protocol version	3	Date and version identifier	17, 18
Funding	4	Sources and types of financial, material, and other support	17
Roles and responsibilities	5a	Names, affiliations, and roles of protocol contributors	1, 17
	5b	Name and contact information for the trial sponsor	17
	5c	Role of study sponsor and funders, if any, in study design; collection, management, analysis, and interpretation of data; writing of the report; and the decision to submit the report for publication, including whether they will have ultimate authority over any of these activities	17, 18
	5d	Composition, roles, and responsibilities of the coordinating centre, steering committee, endpoint adjudication committee, data management team, and other individuals or groups overseeing the trial, if applicable (see Item 21a for data monitoring committee)	11, 16
**Introduction**			
Background and rationale	6a	Description of research question and justification for undertaking the trial, including summary of relevant studies (published and unpublished) examining benefits and harms for each intervention	3, 5
	6b	Explanation for choice of comparators	6, 7
Objectives	7	Specific objectives or hypotheses	6
Trial design	8	Description of trial design including type of trial (eg, parallel group, crossover, factorial, single group), allocation ratio, and framework (eg, superiority, equivalence, noninferiority, exploratory)	6, 7

For peer review only

Methods: Participants, interventions, and outcomes

Study setting	9	Description of study settings (eg, community clinic, academic hospital) and list of countries where data will be collected. Reference to where list of study sites can be obtained	7
Eligibility criteria	10	Inclusion and exclusion criteria for participants. If applicable, eligibility criteria for study centres and individuals who will perform the interventions (eg, surgeons, psychotherapists)	7, 8
Interventions	11a	Interventions for each group with sufficient detail to allow replication, including how and when they will be administered	6, 7, 10
	11b	Criteria for discontinuing or modifying allocated interventions for a given trial participant (eg, drug dose change in response to harms, participant request, or improving/worsening disease)	N/A
	11c	Strategies to improve adherence to intervention protocols, and any procedures for monitoring adherence (eg, drug tablet return, laboratory tests)	N/A
	11d	Relevant concomitant care and interventions that are permitted or prohibited during the trial	N/A
Outcomes	12	Primary, secondary, and other outcomes, including the specific measurement variable (eg, systolic blood pressure), analysis metric (eg, change from baseline, final value, time to event), method of aggregation (eg, median, proportion), and time point for each outcome. Explanation of the clinical relevance of chosen efficacy and harm outcomes is strongly recommended	11, 12
Participant timeline	13	Time schedule of enrolment, interventions (including any run-ins and washouts), assessments, and visits for participants. A schematic diagram is highly recommended (see Figure)	9
Sample size	14	Estimated number of participants needed to achieve study objectives and how it was determined, including clinical and statistical assumptions supporting any sample size calculations	8
Recruitment	15	Strategies for achieving adequate participant enrolment to reach target sample size	8, 9

Methods: Assignment of interventions (for controlled trials)

Allocation:

Sequence generation	16a	Method of generating the allocation sequence (eg, computer-generated random numbers), and list of any factors for stratification. To reduce	9, 10

For peer review only - http://bmjopen.bmj.com/site/about/guidelines.xhtml

		predictability of a random sequence, details of any planned restriction (eg, blocking) should be provided in a separate document that is unavailable to those who enrol participants or assign interventions	
Allocation concealment mechanism	16b	Mechanism of implementing the allocation sequence (eg, central telephone; sequentially numbered, opaque, sealed envelopes), describing any steps to conceal the sequence until interventions are assigned	9, 10
Implementation	16c	Who will generate the allocation sequence, who will enrol participants, and who will assign participants to interventions	9, 10
Blinding (masking)	17a	Who will be blinded after assignment to interventions (eg, trial participants, care providers, outcome assessors, data analysts), and how	9, 10
	17b	If blinded, circumstances under which unblinding is permissible, and procedure for revealing a participant's allocated intervention during the trial	9, 10
**Methods: Data collection, management, and analysis**			
Data collection methods	18a	Plans for assessment and collection of outcome, baseline, and other trial data, including any related processes to promote data quality (eg, duplicate measurements, training of assessors) and a description of study instruments (eg, questionnaires, laboratory tests) along with their reliability and validity, if known. Reference to where data collection forms can be found, if not in the protocol	11, 12, 13
	18b	Plans to promote participant retention and complete follow-up, including list of any outcome data to be collected for participants who discontinue or deviate from intervention protocols	14, 16
Data management	19	Plans for data entry, coding, security, and storage, including any related processes to promote data quality (eg, double data entry; range checks for data values). Reference to where details of data management procedures can be found, if not in the protocol	14
Statistical methods	20a	Statistical methods for analysing primary and secondary outcomes. Reference to where other details of the statistical analysis plan can be found, if not in the protocol	14, 15
	20b	Methods for any additional analyses (eg, subgroup and adjusted analyses)	N/A
	20c	Definition of analysis population relating to protocol non-adherence (eg, as randomised analysis), and any statistical methods to handle missing data (eg, multiple imputation)	N/A
**Methods: Monitoring**			
Data monitoring	21a	Composition of data monitoring committee (DMC); summary of its role and reporting structure; statement of whether it is independent from the sponsor and competing interests; and reference to where further details	16

For peer review only - http://bmjopen.bmj.com/site/about/guidelines.xhtml

		about its charter can be found, if not in the protocol. Alternatively, an explanation of why a DMC is not needed	
	21b	Description of any interim analyses and stopping guidelines, including who will have access to these interim results and make the final decision to terminate the trial	16
/Harms	22	Plans for collecting, assessing, reporting, and managing solicited and spontaneously reported adverse events and other unintended effects of trial interventions or trial conduct	N/A
Auditing	23	Frequency and procedures for auditing trial conduct, if any, and whether the process will be independent from investigators and the sponsor	N/A
**Ethics and dissemination**			
Research ethics approval	24	Plans for seeking research ethics committee/institutional review board (REC/IRB) approval	2, 16, 18
Protocol amendments	25	Plans for communicating important protocol modifications (eg, changes to eligibility criteria, outcomes, analyses) to relevant parties (eg, investigators, REC/IRBs, trial participants, trial registries, journals, regulators)	16, 17
Consent or assent	26a	Who will obtain informed consent or assent from potential trial participants or authorised surrogates, and how (see Item 32)	18
	26b	Additional consent provisions for collection and use of participant data and biological specimens in ancillary studies, if applicable	N/A
Confidentiality	27	How personal information about potential and enrolled participants will be collected, shared, and maintained in order to protect confidentiality before, during, and after the trial	17, 18
Declaration of interests	28	Financial and other competing interests for principal investigators for the overall trial and each study site	17, 18
Access to data	29	Statement of who will have access to the final trial dataset, and disclosure of contractual agreements that limit such access for investigators	18
Ancillary and post-trial care	30	Provisions, if any, for ancillary and post-trial care, and for compensation to those who suffer harm from trial participation	N/A
Dissemination policy	31a	Plans for investigators and sponsor to communicate trial results to participants, healthcare professionals, the public, and other relevant groups (eg, via publication, reporting in results databases, or other data sharing arrangements), including any publication restrictions	18
	31b	Authorship eligibility guidelines and any intended use of professional writers	N/A

For peer review only - http://bmjopen.bmj.com/site/about/guidelines.xhtml

	31c	Plans, if any, for granting public access to the full protocol, participant-level dataset, and statistical code	18
**Appendices**			
Informed consent materials	32	Model consent form and other related documentation given to participants and authorised surrogates	Suppl. material
Biological specimens	33	Plans for collection, laboratory evaluation, and storage of biological specimens for genetic or molecular analysis in the current trial and for future use in ancillary studies, if applicable	N/A

*It is strongly recommended that this checklist be read in conjunction with the SPIRIT 2013 Explanation & Elaboration for important clarification on the items. Amendments to the protocol should be tracked and dated. The SPIRIT checklist is copyrighted by the SPIRIT Group under the Creative Commons "Attribution-NonCommercial-NoDerivs 3.0 Unported" license.

For peer review only

For peer review only - http://bmjopen.bmj.com/site/about/guidelines.xhtml

BMJ Open

Nurture Early for Optimal Nutrition (NEON) participatory learning and action women's groups to improve infant feeding and practices in South Asian infants: pilot randomised trial study protocol

Journal:	*BMJ Open*
Manuscript ID	bmjopen-2022-063885.R2
Article Type:	Protocol
Date Submitted by the Author:	10-Jul-2023
Complete List of Authors:	Manikam, Logan; UCL Institute of Epidemiology and Health Care, Epidemiology and Public Health; Aceso Global Health Consultants<br>Patil, Priyanka; University College London Institute of Epidemiology and Health Care; Aceso Global Health Consultants Pte Limited<br>Naman, Maryan; Aceso Global Health Consultants Pte Limited<br>Allaham, Shereen ; UCL Institute of Epidemiology and Health Care, Department of Epidemiology and Public Health; Aceso Global Health Consultants Ltd,<br>Ong, Zhen Ling; Aceso Global Health Consultants Limited<br>Demel, Isabel-Cathérine; King's College London Faculty of Life Sciences and Medicine, Guy's, King's and St.Thomas' School of Medicine<br>Batura, Neha; University College London, Institute for Global Health<br>Llewellyn, Clare; University College London Department of Behavioural Science and Health<br>Hayward, Andrew; University College London, Epidemiology and Public Health<br>Lakshman, Rajalakshmi; Cambridgeshire County Council, Public Health Directorate; University of Cambridge, Medical Research Council Epidemiology Unit<br>Gilmour, Jenny; Mile End Hospital, Tower Hamlets GP Care Group<br>Webb-Martin, Kelley; London Borough of Newham, Children's Health 0-19 Service<br>Irish, Carol; London Borough of Newham, Children's Health 0-19 Service<br>Archibong, Mfon; London Borough of Newham, Children's Health 0-19 Service<br>Clarkson, Corinne; London Borough of Waltham Forest, Council, Public Health Strategist for Maternal & Child Health<br>Delceta, Daley; Chingford Health Centre Waltham Forest, Operational Lead<br>Islam, Lily; London Borough of Tower Hamlets, Community Facilitator<br>Bajwa, Seema; London Borough of Newham, Community Facilitator<br>Malek, Sabiha; London Borough of Newham, Community Facilitator<br>Bhachu, Jasvir; London Borough of Newham, Community Facilitator<br>Pushpakanthan, Geromini; London Borough of Newham, Community Facilitator<br>Heys, Michelle; University College London Institute of Child Health, University College London Institute of Child Health; Specialist Children and Young People's Services, East London NHS Foundation Trust

	Lakhanpaul, Monica; UCL Great Ormond Street Institute of Child Health Population Policy and Practice, Institute of Child Health; Whittington Health NHS Trust
**Primary Subject Heading**:	Public health
Secondary Subject Heading:	Epidemiology, Evidence based practice, Global health, Nutrition and metabolism, Public health
Keywords:	Community child health < PAEDIATRICS, NUTRITION & DIETETICS, PAEDIATRICS, PUBLIC HEALTH

SCHOLARONE™ Manuscripts

Nurture Early for Optimal Nutrition (NEON) participatory learning and action women's groups to improve infant feeding and practices in South Asian infants: pilot randomised trial study protocol

Short Title/ Acronym: Nurture Early for Optimal Nutrition (NEON) Pilot Feasibility Randomised Controlled Trial

Authors:
[1]Dr Logan Manikam, NIHR Advanced Fellow & Honorary Consultant in Public Health Medicine[1,2]
Priyanka Patil, Research Assistant & Consultant[1,2]
Maryan Naman, Consultant[2]
Shereen Allaham, Research Assistant & Consultant[1,2]
Zhen Ling Ong, Associate[2]
Isabel-Cathérine Demel, Medical Student[2,3]
Dr Neha Batura, Associate Professor in Health Economics[6]
Dr Clare Llewellyn, Associate Professor in Behavioural Obesity Research[7]
Prof Andrew Hayward, Professor of Infectious Disease Epidemiology and Inclusion Health Research[1]
Dr Rajalakshmi Lakshman, Consultant in Public Health Medicine[8]
Jenny Gilmour, 0-19 Service Development Lead THGPCG[9]
Kelley Webb Martin, Director of Delivery, Compliance & Transformation[10]
Carol Irish, Head of Children's Health 0-19 Service[10]
Mfon Archibong, Specialist Health Visiting Research Lead[10]
Corinne Clarkson, Public Health Strategist for Maternal & Child Health[11]
Daley Delceta, Operational Lead[12]
Lily Islam, Tower Hamlets community facilitator
Seema Bajwa, Newham community facilitator
Sabiha Malek, Newham community facilitator
Jasvir Bhachu, Newham community facilitator
Geromini Pushpakanthan, Newham community facilitator
[2]Dr Michelle Heys, Associate Professor of Community and Population Child Health, Consultant Paediatrician[4,5]
Monica Lakhanpaul Professor of Integrated Community Child Health[13,14] , On Behalf of the NEON Steering Team.

Membership of the NEON Steering Team is listed in the Acknowledgements

---

[1] Logan Manikam and Priyanka Patil are joint first authors

[2] Dr Michelle Heys and Professor Monica Lakhanpaul are joint senior authors

For peer review only - http://bmjopen.bmj.com/site/about/guidelines.xhtml

Affiliations:

1. Department of Epidemiology and Public Health, University College London Institute of Epidemiology and Health Care, London, UK
2. Aceso Global Health Consultants Pte Limited, Singapore
3. Department of Life Sciences & Medicine, King's College London GKT School of Medical Education, London, UK
4. Specialist Children and Young People's Services, East London NHS Foundation Trust, 84 West Ham Lane, London E15 4PT.
5. Population, Policy and Practice, UCL Great Ormond Street Institute of Child Health, 30 Guildford Street, London WC1N 1EH, UK
6. Institute for Global Health, University College London, 30 Guilford Street, London WC1N 1EH.
7. Department of Behavioural Sciences & Health, UCL IEHC, 1-19 Torrington Pl, Fitzrovia, London WC1E 7HB.
8. MRC Epidemiology Unit, University of Cambridge
9. Tower Hamlets GP Care Group, Mile End Hospital, London, UK
10. Children's Health 0-19 Service, London Borough of Newham, 1000 Dockside Road E16 2QU
11. Waltham Forest Council, London, UK
12. Chingford Health Centre Waltham Forest, London, UK
13. Population, Policy and Practice, UCL Great Ormond Street Institute of Child Health, University College London, London, WC1N 1EH, UK
14. Whittington Health NHS Trust, London, UK, N19 5NF

Corresponding author: Dr Logan Manikam

Email: logan.manikam.10@ucl.ac.uk
Telephone: +44 7868721152

Key Words: Community-based intervention; infant; child health; nutrition; feeding practices; clinical trial

Word Count: 3999

For peer review only

ABSTRACT

Introduction: Feeding practices developed in early life can impact a child's nutrition, growth, dental health, cognitive development and lifetime risk of chronic diseases. Substantial evidence suggests ethnic health inequalities, and non-recommended complementary infant feeding practices among UK's South Asian (SA) population. Nurture Early for Optimal Nutrition (NEON) aims to use Women's group Participatory Learning and Action (PLA) cycles to optimise infant feeding, care and dental hygiene practices in SA infants <2 years in East London.

Methods and analysis: A three-arm pilot feasibility cluster randomised controlled trial will assess feasibility, acceptability, costs and explore preliminary effectiveness for proposed primary outcome (i.e. reporting on BMI z-score). Multilingual SA community facilitators (CFs) will deliver the intervention, Group PLA Cycle, to mothers/carers in respective ethnic/language groups. 12 wards are randomised to face-to-face PLA, online PLA and usual care arms in 1:1:1 ratio. Primary outcomes are feasibility and process measures (i.e. BMI z-score, study records, feedback questionnaires, direct observation of intervention, and sustainability) for assessment against Go/Stop criteria. Secondary outcomes are cluster-level and economic outcomes (i.e. eating behaviour, parental feeding practices, network diffusion, children development performance, level of dental caries, GP utilisation, costs, staff time). Outcomes are measured at baseline, every two weeks during intervention, 14-weeks and at 6-months by blinded outcome assessors where possible. This study will use concurrent mixed-methods evaluation. Quantitative analyses include descriptive summary with 95% confidence interval (CI) and sample size calculation for the definitive trial. The intervention effect with CI will be estimated for child BMI z-score. Implementation will be evaluated qualitatively using thematic framework analysis.

Ethics and dissemination: Ethics approval was obtained from UCL, NHS (HRA and HRCW). Results will be published in peer-reviewed journals, presented at scientific conferences/workshops with commissioners, partners and participating communities. Plain language summaries will be disseminated through community groups, websites and social media.

Word Count: 299/300

Trial registration: This trial will be registered with the International Standard Randomised Controlled Trial Number Register: IRAS-ID – 296259 (ISRCTN10234623).

For peer review only

ARTICLE SUMMARY

Strengths

- The three-armed approach will allow for comparisons of online and face to face delivery with usual care
- The collection and interrogation of process and feasibility data with clearly defined stop-go criteria will allow for an informed decision around feasibility of a larger scale evaluation
- A broad set of secondary outcome measures will allow for estimation of potential impact, in addition to understanding of the feasibility of capturing reliable and complete data measuring these outcomes

Limitations

- Project capacity precludes the spacing of group sessions according to religious celebrations which might affect retention to group session.
- Risk of performance and measurement bias as neither participants nor all research staff will be fully blinded.

For peer review only

INTRODUCTION

The first 1,000 days of life is important for growth and brain development. There is mounting evidence that influences during pregnancy and infancy may alter lifetime risk of nutrition and dental related diseases [1]. Feeding practices developed during this period can impact children's nutrition, growth, dental health, cognitive development, and may lead eventually to heart disease, obesity, and diabetes [2].

Britain's ethnically diverse population is mostly disadvantaged across a range of socioeconomic outcomes, forming fundamental causes of ethnic health inequalities in the UK [3]. Some of the widest differences have been observed in the South Asian (SA) population, more so for Pakistani and Bangladeshi communities compared to Indians [4].

Systematic reviews assessing complementary feeding practices and the sociocultural beliefs underpinning them in children <2 years within SA families in the UK were explored. Despite the adoption of the WHO Infant and Young Children Feeding Guidelines, there remains substantial evidence of non-recommended complementary feeding practices such as early introduction of solids, introduction of minimum dietary diversity or minimum frequency of meals being followed [5-7]. Contributing factors that persisted post-migration included bicultural issues or low acculturation levels and conflicting information among health professionals, extended family, and religious and community leaders.

Effective early life interventions tailored to different ethnic groups have great potential to reduce the development of short- and long-term conditions and, thereby, lifetime inequalities. At present, however, few of such interventions exist [8]. Traditionally, UK health services provide unidirectional information based on guidelines and NHS recommendations. Specific ethnic groups may be marginalised by this approach as most advice is not tailored to their cultural practices. There is a need for interventions that target these communities through a top-down unidirectional approach and bring a change originating from within the communities. Therefore, it is important to work in partnership with communities thereby building their capacity to work closely with local authorities/stakeholders. The asset-based community development approach (ABCD) has been successful in community development as this helps communities identify their assets through asset mapping and mobilize them to bring along the desired change [9].

Based on Lakhanpaul et al.'s MARKS (Motivation Awareness Resources Knowledge and Skills) model [10], current practice lacks the space to understand parents' motivation and ability to support families in skills development, all of which is key to providing optimal infant feeding, care, and dental hygiene support. Considering the resource constraints of the NHS and its 10 Year Forward Plan which aims to shift the emphasis to the community, highlighting a need for interventions that enable communities to utilise their available community assets. The Participatory Learning and Action (PLA) cycle is an iterative process led by multilingual facilitators through a four-stage cycle of identifying and prioritising contextual issues, designing problem-solving strategies, implementing these strategies and a post-implementation evaluation. The PLA approach is a low-cost, community-based, culturally

For peer review only - http://bmjopen.bmj.com/site/about/guidelines.xhtml

sensitive intervention that can be adapted from Low- and Middle-income Countries (LMICs) to High-income Countries (HICs), as well as to different population groups and topic areas. This strategy is also recommended by the WHO to improve maternal and infant survival [11]. A meta-analysis of seven cluster randomised controlled trials (RCTs) utilising PLA in LMICs showed reduced maternal and neonatal mortality by 37% and 23% respectively [12].
An RCT and controlled before-and-after study have demonstrated reduced maternal depression, increased exclusive breastfeeding rates, and decreased under-5 morbidity [13]. A Mumbai-based RCT also showed improvements in maternal practices and care-seeking behaviour [14]. Recognising WHO recommendations and the success of PLA in LMICs, this approach was adopted for the Nurture Early for Optimal Nutrition (NEON) programme.

In NEON Phase 1 (Intervention Development) was co-developed with the SA community facilitators (CF) (now community researchers/independent observers in NEON Phase 2), community members, research team and independent experts (i.e. health visitors, dentists, dieticians, nutritionists, GPs, and midwives). This followed the MRC complex intervention framework , which involved using formative research and prior trials as evidence-based factors; micro-adaptations involved adjusting language, literacy levels for materials, using picture cards, face-to-face, and interactive learning delivery methods. This protocol describes NEON Phase 2: Pilot Feasibility Cluster RCT.

AIMS AND OBJECTIVES

This pilot feasibility study aims to evaluate the feasibility and inform the design and conduct of a definitive cluster RCT comparing NEON Women's Group PLA Cycle versus usual care to optimise infant feeding, care, and dental hygiene practices in SA infants aged <2 years in East London.

Primary objectives:

Assess: 1) the feasibility of proceeding to a definitive trial against predetermined Go/Stop criteria (i.e. recruitment, retention rates, intervention support, acceptability),2) intervention fidelity and participant' adherence, 3) implementation of face-to-face versus online intervention arms

Secondary objectives:

Assess: 1) feasibility, completeness, acceptability and adequacy of blinding when collecting proposed outcomes to establish optimal outcomes and data collection procedures, 2) time needed to competently deliver both versions of NEON intervention, 3)the mean, standard deviation and intervention effect with 95% confidence interval (CI) of the proposed primary outcome, child BMI z-score

For peer review only - http://bmjopen.bmj.com/site/about/guidelines.xhtml

METHODS AND ANALYSIS

Design

This three-arm pilot feasibility cluster RCT study is expected to run from July 2021-May 2023 in 3 East London boroughs. 18 clusters defined as borough wards will be randomised with 1:1:1 allocation to 2 intervention arms (face-to-face or online NEON Women's Group PLA Cycle) and 1 control arm (usual care). An online arm enables the trial to continue under Covid-19 restrictions; usual care was selected as control to avoid depriving participants of essential and available infant and maternal care services. Multilingual CFs will deliver interventions in respective ethnic/language groups. Ward-level randomisation is a trade-off between minimising contamination and ensuring maximum SA population representation given the diversity in the ethnic makeup, i.e. SA sub-groups, of the aforementioned boroughs.

Patient and Public Involvement

The research has been co-designed with SA CFs involved in all stages of developing and evaluating the intervention to ensure relevant research questions and an acceptable study design. Development phase, representing different SA communities, are now recruited as Community Researchers (CRs) to support; protocol development and ethics application by ensuring participants-facing documents are clear, appropriate and sensitively-worded; topic guide and questionnaire development; strategy development and troubleshooting (e.g. recruitment); interpretation of findings into appropriate and attainable recommendations for practice; review and revision of draft academic papers; dissemination activities and development of plain language summaries.

Setting

This study will run sequentially in the London Boroughs of Tower Hamlets (TH), Newham (NH) and Waltham Forest (WF), all of which are considered to be among 5% most deprived in England [15]. The boroughs were analysed in terms of ethnic make-up; in each borough, the 6 wards with the highest density of Asian population were selected based on census data [16]. The most common SA ethnic/language groups will be targeted. Figure 1 shows the number of wards and PLA groups by boroughs. Figure 2 shows the overview of participant flow throughout the trial.

Figure 1. Overview schematic detailing number of wards & PLA groups by East London boroughs

Figure 2. Overview of the NEON pilot feasibility RCT design and participant flow

For peer review only - http://bmjopen.bmj.com/site/about/guidelines.xhtml

Randomisation

Randomization of wards will be done before the recruitment by a separate member at UCL using the Research Randomizer software for the 18 wards stratified by borough [17]. The randomised wards will be shared with the RA at the end of recruitment. Once sufficient numbers are recruited in an ethnic/language group, the RA will assign participants to their respective arms. Recruitment will be performed by NHS personnel who will not be aware of the randomisation. CFs/CRs will be made aware of the participant allocation before the PLA sessions start to make contact and invite participants

Eligibility Criteria

Participants

Inclusion

- Mothers or female carers of an infant <24 months;
- Indian, Pakistani, Sri Lankan or Bangladeshi background;
- Resident in a randomised study ward in TH, NH and WF;
- Willing and able to provide informed consent

Exclusion

- <18 years old;
- Anticipating moving out of *a priori* defined geographical areas before or after delivery
- Current or recent participation in another study within 4 weeks of trial commencement.

Staff (Community Facilitator) Eligibility

- Female
- Have at least one child, preferably <24 months
- From the SA community in TH, NH, or WF
- Able to read and write
- Fluent in English and another local language (Hindi, Bengali, Sylheti, Urdu, or Tamil)
- Understand SA social norms, values and culture within study boroughs
- Known and respected by their local community
- Motivated to address issues related to infant growth and development
- Able to manage a group and have some leadership qualities

For peer review only - http://bmjopen.bmj.com/site/about/guidelines.xhtml

Sample size

With communication and cultural sensitivity as key drivers of the NEON intervention, the sample size will partly depend on the coverage of ethnic and language groupings. The choice of ethnic/language groups were informed by the 2011 ONS Census [16] and input from CFs stressing that, while language is important for communication, people's practices are embedded in different ethnicities.

The main ethnic/language group(s) identified are: 1 group in TH (Bangladeshi/ Bengali & Sylleti); 4 groups in NH (Indian/Gujrati, Indian/Punjabi, Pakistani/Urdu, and Sri Lankan/Tamil); 3 groups in WF (Indian/Punjabi, Pakistani/Urdu, Sri Lankan/Tamil). In each borough, 1 Women's Group PLA Cycle will run per ethnic/language group per ward.

We aim to run 20 to 32 Women's Group PLA Cycle (with equal numbers of face-to-face and online PLA groups) at 6-8 participants per PLA group. Including control groups totals to 288-384 participants. Assuming an 80% recruitment or retention rate, this enables us to be 85% confident that the true population rate will fall between 0.76%-0.84% (precision=0.04). This was estimated using a geometric mean cluster size=16 participants, cluster size coefficient of variation (COV)=0.7 and Intra-Cluster Correlation Coefficient (ICC)=0.02.

Recruitment

Participants

Due to the diversity of the target population, multiple recruitment methods are needed to maximise reach and minimise inequalities or non-representative samples [18] [19]. For instance, relying solely on online recruitment may be ineffective due to digital poverty. Thus, other mediums including leveraging on existing social networks (snowballing), identifying and fostering collaborations with
community leaders, and creating clear and succinct recruitment materials [20] were employed. All study promotional materials (e.g. participants information sheets, posters) will be available in English and local languages. To standardise the recruitment process, we will provide the same recruitment script and study materials to all recruiters. Potential participants will contact the RA, who will confirm eligibility, consent and register them into the study.

Participant recruitment will follow three strategies with regular advice by CRs to maximise reach:
1)Recruitment through CFs: CFs will share study materials with their network to invite eligible participants with subsequent snowballing. We will provide training to standardise CF recruitment. 2)Recruitment through Health Visitor (HVs), GPs, and Midwifery teams: HVs, GPs, and midwives who have been identified based on their geographical proximity to study wards will share study materials to eligible participants. Details of those interested will be given to the RA. 3)Online recruitment: Online social media campaigns on Facebook and Instagram will be utilised. See Supplementary Material-1 for advertisement materials. Recruitment bias will

For peer review only - http://bmjopen.bmj.com/site/about/guidelines.xhtml

be limited due to the above-mentioned multiple channels of recruitment and all recruiters will be blinded thereby not knowing the allocation details at the time of recruitment.

Staff

We will recruit CFs from our local stakeholders' network. Recruitment via CFs is particularly useful for studies with underserved or traditionally hard-to-reach populations [21]. CFs are leaders/champions in their local community who, with training and support, help improve the health and wellbeing of their communities. In line with NIHR's "INVOLVE" guideline, CFs will be paid £30 for each PLA meeting and additional £5 to cover travel costs for face-to-face meetings. A comprehensive manual for the CFs will be developed with PLA experts. A tool kit will be provided to support the learnings. The CFs will attend a three-day comprehensive workshop and additional sessions will be conducted in between the 8-meeting cycle as a refresher and address any challenges. Biweekly meetings with the RA will be conducted to discuss learnings from the sessions. The CFs will be paid through vouchers for their time similar to a zero-hour contract. HV, GP and midwives would be identified and recruited by our study partners at each study ward. Payments will be supported by NHS support costs and decided with the relevant clinical lead.

Allocation concealment and blinding

Firstly, treatment allocation will be concealed from participants and recruiters during participant recruitment to reduce recruitment bias. After recruitment has been completed for that ethnic/language group in the borough, the RA will reveal ward allocation to participants and CFs via text or email, listing postcodes by trial arms to allow CFs to effectively organise and deliver the intervention.

Secondly, outcome assessors (CRs) will be blinded where possible to reduce measurement bias. This will be achieved by CRs logging the data collected onto an electronic platform that automatically links participant ID to maintain blinding. Baseline measurement of BMI will be done by health visitors in each borough who will be blinded.

Intervention

NEON Women's Group PLA Cycle

The co-adapted NEON Women's Group PLA Cycle tailored to ethnic/language groups will be delivered face-to-face at community/children's centre or online over 14 weeks (1 session per 2 weeks). HVs, GP, and midwives' teams will be invited to 1-2 sessions to provide evidence-based information upon participants' request. To reduce study contamination, participants may not switch wards (i.e. switch arms) after treatment allocation is revealed. Figure 3 illustrates the aim of each PLA session.

Figure 3. NEON Women's Group PLA Cycle

A culturally-sensitive intervention package has been co-developed consisting of:

1. PLA group facilitator manual
2. Picture cards with recommended and non-recommended infant feeding, care and dental hygiene practices, and facilitators/barriers to uptake
3. Healthy baby food recipes

For peer review only - http://bmjopen.bmj.com/site/about/guidelines.xhtml
For peer review only

4. Community asset maps (e.g. low-cost fruit/vegetable shops)
5. List of relevant resources and services.

Online arm participants may access this toolkit through the eRedbook platform and receive NEON information the via the app or website. A Logic Model (Supplementary Material-2) shows the relationship between these activities and resources and their intended short-, medium- and long-term effects.

Control (Usual Care)

In all wards, HV teams have regular mandatory postnatal visits for all families of new-borns and infants. These visits are immediately after birth, 6-8 weeks, 12-16 weeks, 1 year, and 2-2.5 years. One optional prenatal visit is conducted by HVs between 28-32 weeks of pregnancy. Supplementary Material-3 lists other initiatives and resources available in each study borough as part of usual care.

Outcome

Primary outcomes: Feasibility and process outcome measures
We will collect quantitative and qualitative data to assess Go/Stop criteria (Table 1) on recruitment and retention rates, intervention support, acceptability, fidelity and participant adherence using:

1. *Study record:* An enrolment log will record all eligible participants, total enrolled, reasons for non-participation, number followed-up on child BMI z-score, and the date on how many people responded to adverts/invitations. Pre-screen failure logs will record those eligible but not enrolled, with reasons. PLA session audio/video recordings will be collected using secured Dictaphone, video cameras, CCTV, and/or Zoom recording.

2. *Process measurements:* These include Participants' Feedback Questionnaire, Facilitator's Feedback Form, PLA Cycle Meeting Register, direct observation of intervention delivery and CF performance by CRs, and Sustainability Assessment Tool (Supplementary Material-4).

Definite Go	Definite Stop
>=50% of eligible participants consenting to pilot feasibility study	<40% of eligible participants consenting to pilot feasibility trial
>=80% of mothers attend >=60% of planned sessions in the intervention arm.	<20% of mothers attend >=60% of sessions as planned in each intervention arm
Retention of >=70% of consented participants for primary outcome data collection	Retention of <50% of participants at 6 months
High intervention support with respect to content, frequency, duration, and quality	Low support of Intervention Procedures

Intervention is perceived as acceptable	Intervention perceived as unacceptable

Table 1: Go/ Stop criteria for the definitive trial[3]

Secondary outcomes: Proposed outcomes for the definitive trial
All other individual-level, cluster-level and economic outcome measures proposed for the definitive trial will be assessed on the response, completion rates, acceptability and the adequacy of blinding for outcome assessors during data collection. Table 2 summarises outcome measures and data collection timing. See Supplementary Material-5 for collection tools.

Outcome	Outcome Measures	Data Collection	Timing			
			Baseline	Every 2 weeks (end of each PLA meeting)	14 weeks (end of PLA cycle)	6 months
**Proposed primary individual-level outcome**	Individual Child BMI z-score	RA, CRs	✓		✓	✓
**Proposed secondary individual-level outcome**	Children feeding behaviour[3]	RA, CRs	✓		✓	✓
	Parental feeding style[3]	RA, CRs	✓		✓	✓
	Audio/Video recording of child eating behaviours & parental feeding practices	RA	✓		✓	✓
	4-day food diary[4]	RA, CRs	✓		✓	✓
	Network diffusion†[3]	RA, CRs	✓			✓
**Proposed secondary cluster-level outcome**	Children's development performance	RA, CRs		✓		✓
	Level of dental caries	RA	✓			✓

[3] The study will have to meet the Definite Go criteria in order for the study to be feasible and to be able to proceed. However, if any one of the Definite Stop criteria is met then the study stops. The 'Go/Stop' indicates that it is either feasible or not feasible to proceed to a definitive trial. The progression rules have been approved by the steering and data management team. Should any of the progression rules not be met, assessments and adjustments of the NEON pilot feasibility RCT will be negotiated before proceeding to a definitive trial.

[4] Community Facilitators will assist in administering or completing these data collection tools before sending them to RA/ CRs during the intervention period; †For intervention groups only; CR= Community Researchers; RA = Research Assistant

	GP healthcare utilisation	RA	✓			✓
**Economic outcome**	Cost tool	RA	✓			✓
	Partners time questionnaire	RA				✓
**Process outcome†**	Participants Feedback[3]	RA, CRs		✓		
	PLA cycle meeting register[3]	RA, CRs		✓		
	Direct observation	RA, CRs		✓		
	Sustainability assessment	RA, CRs		✓		

Table 2. Schedule of Outcome Assessments

Primary individual-level outcome

1. *Child BMI z-score:* Health visitors will measure infants' length/height, head circumference and weight. The RA will refer to the WHO 2006 growth standard [2], using age- and gender-adjusted length or height(m)/weight (kg)^2 to provide a comparison on the cluster-level.

Secondary individual-level outcomes

1. *Children's feeding behaviour* will be measured using 6 of 8 domains adapted from the validated Children's Eating Behaviour Questionnaire (CEBQ) deemed suitable for infants: food responsiveness; enjoyment of food; emotional overeating; desire to drink; satiety responsiveness; slowness in eating; emotional undereating; and food fussiness [7, 8, 22]. Obesity risk could be linked with external eating, emotional overeating, and food fussiness [7].

2. *Parental feeding style* will be assessed with a validated self-reported Parental Feeding Style Questionnaire (PFSQ) with four scales: emotional feeding; instrumental feeding; encouragement to eat; and control overeating [23].

3. *Audio/video recording of child eating behaviour and parental feeding practices* [24] involves asking participants about the meal (e.g. name, ingredients) and creating annotations that link to behaviour codes identified from NEON formative study [25]. Before-and-after audio/video recordings will be thematically analysed using Elan Software [26] to measure intervention effect.

4. *Child food intake*: The 4-day food diary [7] is a self-reported, prospective, open-ended survey that collects detailed quantitative estimates of food consumption of infants between 4-18 months [6]. It assesses nutrient intake (e.g. total fat, total carbohydrate, salt, sugar) by comparing with age/sex specific UK dietary reference values [27]. Dietary data will be presented by age group (e.g. 4-6 months, 7-9 months, 12-18 months).

5. *Network diffusion of study materials between participants and their communities*: CRs will track the number of downloads using the eRedbook platform. Participants will also complete a questionnaire on the number of people they shared the material with, their family size, age, gender, relationship to the participant and platform used.

6. *Equality impact assessment (EIA)*: This trial should reflect the diversity of the target population by enabling equal access while accommodating different needs. An EIA tool will systematically assess likely effects of the intervention on people (e.g. with respect to disability,

For peer review only

gender, ethnicity, age, sexual orientation, religion/belief etc.), mitigate adverse impacts and identify active steps to address existing disadvantage and promote equality.

Secondary cluster-level routine outcomes

Although this intervention acts on the individual-level, we expect a social diffusion of good infant feeding and dental hygiene awareness and practices in the medium- to long-term (Supplementary Material-2, Logic Model). While immediate cluster-level changes are not expected, we would like to pilot cluster-level routine data collection.

1. Development performance of children: The Ages and Stages questionnaire (ASQ-3) include communication, gross motor, fine motor, problem-solving, and personal-social domains [5]. Three versions are available: 24-months, 27-months and 30-months. ASQ-3 data collected by HVs during mandated visits are stored in EMIS/ RIO databases.
2. GP healthcare utilisation data is available in participants' medical record.
3. Level of dental caries and other signs of tooth decay is available in NHS dental services records for registered patients beginning at 6-months of age and approximately every half-year following initial visit [17,28,29].

Economic outcomes

1. Cost: The RA will use a spreadsheet will capture the cost of the NEON Intervention Development
and pilot feasibility RCT phases every 6 months.
2. Partners' time used: A questionnaire will capture time spent for the delivery of NEON-associated programmes.

Data Collection and Management

Outcome data will be collected at baseline, every 2-weeks during intervention (for process measures), 14-weeks immediately post-intervention, and 6-months follow-up (for economic data). During the intervention period, several outcome measurements will be administered by CFs to participants or completed by CFs and sent to CRs/RA. Other outcomes and post-intervention outcomes will be assessed directly by CRs/RA (Table 2). Each case will be assigned an ID. Data collected digitally or in paper format will be stored in UCL S: Drive and/or UCL cabinets. We will use RedCap electronic data management system [30]; the RedCap API allows flexible and straightforward data import and export to data analysis software.
We will form a data sharing agreement with study partners for the RA to extract and link routine cluster-level data to trial participants. To guarantee confidentiality, pseudonymised participant data will be stored on encrypted, password-protected computer or UCL S:Drive accessible only to study staff and authorised personnel. Password-protected personal data will be stored separately from trial data on secure UCL computers. Only pseudonymised quotes or data from audio/video recording may be published. Personal data and audio/video recordings will be stored for 3-years post-study and destroyed; research data will be stored for 20-years post-study.

Analysis

For peer review only - http://bmjopen.bmj.com/site/about/guidelines.xhtml

Overall, quantitative and qualitative data will be analysed concurrently at multiple time points to identify early problems that are rectified as the trial progresses. Quantitative data such as the BMI z-score will be descriptively summarised using mean and standard deviation (SD) for continuous variables, number with percentages for categorical variables, and 95% CI with a breakdown of participants by trial arm, ethnic/language group and borough where relevant. An intention to treat analysis will also be considered. Results will be collectively assessed against the Go/Stop criteria. Before progression to the definitive trial, we will systematically and rigorously assess areas of improvement using a structured discussion tool: A process for Decision-making after Pilot and feasibility Trials (ADePT) [31]. All statistical analysis will be done by an independent statistician who would be blinded.

Sample Characteristics

Participant demographic characteristics will be descriptively summarised by trial arm. We will check for recruitment bias across different arms at baseline and compare later recruits who are prone to unblinded recruitment.

Feasibility and Process Measures

Across the three arms, we will check for any differential recruitment or retention rates and any consequent systematic differences in participant characteristics over time, as control arm participants may be less keen to participate or remain in the study. For the two intervention arms, we will conduct a mixed-methods implementation evaluation to explore the possibility of a blended approach in the definitive trial as some features of each delivery mode might be particularly salient. Qualitative data will be analysed thematically using framework analysis that include participants' motivation for engagement, expectations, experiences, intervention acceptability, implementation barriers, and suggestions for improvement. Negative 'deviant' cases will inform interpretation.

Outcome measures for the definitive trial

1. *Feasibility of collecting outcome measures:* To assess completeness, degrees of missingness at the individual item level and the entire outcome measure will be reported overall, by time-point and trial arms. Other feasibility measures would be captured by analyses of process measures above.

2. *Intervention effect for proposed primary outcome (BMI z-score):* Conditioned upon low recruitment bias and sufficient adherence to trial protocol, we will estimate the difference in BMI z-score between each intervention arm versus the control arm. We will consider methods to pool data across age groups depending on the actual sample age breakdown.

3. *Sample size and power calculation for the definitive trial:* Data from the pilot feasibility trial will enable us to determine (i) the smallest difference of clinical importance (through multiple testing of several key endpoints for the proposed primary outcome), (ii) the clinically justifiable power-significance level or scientifically acceptable probability of "false positive" (type I error) results, and (iii) whether we need to adjust the calculated sample size for expected level of non-compliance/ drop-in and drop-outs. We will report the ICC.

4. *Economic Evaluation:* This will be conducted from a provider and user perspective which includes direct cost (e.g. time, resources spent on training and participation of CF, participants' overall household consumption and childcare costs). We will estimate the total cost, average annual cost, cost-effectiveness, equity impact analysis and compare across three arms.

Monitoring

The Principal Investigator (PI) will be responsible for day-to-day monitoring and study management.

For peer review only - http://bmjopen.bmj.com/site/about/guidelines.xhtml

The NEON Steering and Data Management Team will ensure adherence to all relevant regulations and principles of good clinical practice. The team consists of multidisciplinary experts in child health, practitioners, PLA expert, SA independent observers from NEON 1, and Queen Mary University of London Pragmatic Clinical Trials Unit. Committee meetings will be held every 3 months to review the Go/Stop criteria (Table 1) and interim analyses. The team will agree to the trial protocol and facilitate any necessary protocol amendments; provide independent expert advice (including on project management governance); monitor progress; ensure adequate deadlines are set and met; monitor and advise NEON Core Team (Trial Management Group) on strategic decisions in light of new evidence; and ensure successful delivery by meeting and reporting on study progress with the Core Team biannually. The PI and other Core Team members will attend all necessary meetings and report on the study progress.

Any adverse events or safety concerns will be captured in the Participant Feedback Questionnaire and Facilitator Report Form (every 2-weeks during intervention period) and reported to the steering and safety monitoring team.

The UCLH/UCL Joint Research Office, on behalf of the Sponsor (UCL), may conduct random audits in accordance with the UK Policy Framework for Health and Social Care Research and UCL's policies and procedures.

ETHICS AND DISSEMINATION

Ethical approval will be obtained from UCL Research Ethics Committee and NHS Health Research Authority. The participant information sheet will be provided in English or participants' native language in-writing and/or verbally by community facilitators for informed consent. The RA or CRs will obtain written or audio-recorded consent ≥24 hours later [32]. Participation will be voluntary and they may withdraw anytime without prejudicing treatment or usual care. No incentives will be provided except for small reimbursements (e.g. childcare at the local centre where PLA meetings take place).

UCL will provide insurance cover and we will also signpost any vulnerable participants who require protection from harm to relevant safeguarding organisations.

Regular progress updates will be shared via UCL or NIHR websites and patient group newsletters. Study findings will be submitted for 3-5 publications in high impact factor peer-reviewed journals and presented at national and international conferences. Researchers and community members from the steering team will contribute to confirming analysis and write-up, adhering to authorship guidelines. To reach wider audiences, we will (i) disseminate plain language summaries for each academic paper through community groups, social media and You Tube; (ii)present findings at lay-person meetings at community or children centres through the NIHR CLAHRC network and (iii)organise 2-3 annual workshops with commissioners, partner organisations and NEON community facilitators to share key findings and recommendations.

Acknowledgements

The authors would like to thank the South Asian community facilitators in the NEON Intervention Development phase: Lily Islam, Seema Bajwa, Aasma Baiyat, Jasvir Bhachu, Geromini Pushpakanthan, Sabiha Malek, Cllr Lakmini Shah; and community members of the London Boroughs of Tower Hamlet, Newham, and Waltham Forest for their important contribution and engagement to this research project. Furthermore, the authors would like to thank the 'Conversation Café' initiative led by Cllr

For peer review only - http://bmjopen.bmj.com/site/about/guidelines.xhtml

Lakmini Shah for her significant help in the recruitment of the community facilitators and members, and in identifying the community centres to carry out the intervention development meetings. The authors would also like to acknowledge the contribution of the NEON core team, steering team and all health experts in validating intervention NEON toolkit. We would like to thank the Women & Children First Charity and First Steps Nutrition Trust for their valuable contributions and guidance throughout the study. In addition to the authors, members of the NEON steering team consist of Prof Atul Singhal, Joanna Drazdzewska, Dr Sonia Ahmed, Amelie Gonguet, Gary Wooten, Dr Ian Warwick, Vaikuntanath Kakarla, Alex Nelson, Phoebe Kalungi, Prof Richard Watt, Prof Mitch Blair, Prof Audrey Prost, Dr Edward Fottrell, Ashlee Teakle, Prof Oyinlola Oyebode, Ciarán Forde, Keri McCrickerd, Dr Rana Conway, Subarna Chakraborty, Sumire Fujita, Tala Khatib and Delaney Douglas-Hiley.

Steering team members had an opportunity to critically review results and contribute to the process of finalising this paper.

For peer review only

Author's Contributions

ML, LM, MH, NB, CL, AH, SA conceived the original concept of the study and designed the research methodology. SA carried out the Intervention Development meetings, workshops, co-development of the intervention toolkit, wrote and devised the paper along with LI, SB, SM, JB and GP. LM, ML, ,MH, SA, PP, MN, ZO, NB, CL, AH, RL, JG, KWM, CI, MA, CC, DD validated the study and revised the manuscript critically for important intellectual content. ICD was involved in drafting materials for the NEON meetings and workshops of the Intervention Development Phase. MN and ICD contributed to the manuscript writing, and prepared it for submission. LM, SA, ML and MH had primary responsibility for the final content. All authors read and contributed to reviewing the study data, the designing of the manuscript, and the approval of the final manuscript.

Funding

Logan Manikam, Shereen Al Laham & Priyanka Patil are funded via a National Institute for Health Research (NIHR) Advanced Fellowship (Ref: NIHR300020) to undertake the Pilot Feasibility Cluster Randomised Controlled Trial of the *NEON* programme in East London. Prof Monica Lakhanpaul was funded by the NIHR Collaboration for Leadership in Applied Health Research and Care (CLAHRC) North Thames.

Disclaimer

The views expressed in the publication are those of the author(s) and not necessarily those of the sponsor (UCL), funder (NIHR), study partners (Tower Hamlets GP Care Group, London Borough of Newham Council, Waltham Forest Council).

Conflicts of Interest

The authors declared no potential conflicts of interest with respect to the research, authorship and/or publication of this paper.

Participant consent for publication

Participant information sheets and consent forms have been provided to community members and those expressing interest. Community participants have agreed to participate and given audio/video consent prior to their participation in the workshops. Verbal consent was witnessed and formally recorded. All participants have been informed of their right to freely withdraw from the study at any

For peer review only - http://bmjopen.bmj.com/site/about/guidelines.xhtml

time. Confidentiality of personal data has been ensured through the use of anonymisation techniques as stated in the Data Protection Act (1998) and in line with the General Data Protection Regulation (2018). All participant data has been anonymised and stored on an encrypted password protected computer. Data can only be accessed by the authorised research personnel.

Ethics approval

This study has obtained ethical approval from UCL Research Ethics Committee [Ethics ID 17269/001], Sponsor reference number: 142600, Funding Reference: NIHR300020 and IRAS number: 296259, Ref: 21/SW/0142. Study protocols and relevant documents will be reviewed by UCL Research Ethics Committee, NHS Health Research Authority (HRA) and study partners involved in establishing data sharing agreement for linking participants' routine data (Tower Hamlets GP Care Group, London Borough of Newham Council, Waltham Forest Council).

Provenance and Peer Review

Not commissioned; peer reviewed for ethical and funding approval prior to submission.

Data Sharing Statement

The data supporting the findings of this study is available upon reasonable request from the corresponding author.

CURRENT STUDY STATUS (AS OF JAN 2023)

The study is currently in the intervention delivery phase.
There has been an overall delay in the timeline of the study due to the COVID - 19 pandemic, lockdown and NHS staff shortage in our study wards.

Recruitment for the study commenced in March 2022 and was completed in Sep 2022. Following recruitment, we launched our first PLA meeting in Tower Hamlets in Oct 2022 and then in Newham in the week following. Waltham Forest dropped out of the study due to lack of supporting staff in the recruitment phase.

We have completed 5 out of the 8 PLA meetings and the PLA meeting cycle will conclude in Feb 2023. Following this we will initiate data analysis and reporting of study findings. We will collect follow-up data in April 2023 which will be the last data collection time point for this trial.

REFERENCES

[1] World Health Organization (WHO), Postnatal care for mothers and newborns: Highlights from the World Health Organization 2013 Guidelines. Available from: http://www.who.int/maternal_child_adolescent/publications/WHOMCA-PNC-2014-Briefer_

For peer review only

A, 2015. 4.

[2] Group, W.M.G.R.S., WHO Child Growth Standards based on length/height, weight and age. Actapaediatrica (Oslo, Norway: 1992). Supplement, 2006. 450: p. 76.

[3] Evandrou M, Falkingham J, Feng Z, et al Ethnic inequalities in limiting health and self-reported health in later life revisited J Epidemiol Community Health 2016;70:653-662.

[4] Brown MS. Religion and economic activity in the South Asian population. Ethnic and Racial Studies. 2000;23(6):1035-61

[5] ASQ-3. The Ages & Stages Questionnaires, Third Edition. [cited 2019 30 November]; Available from: https://agesandstages.com/products-pricing/asq3/.

[6] Lennox, A., et al., Diet and nutrition survey of infants and young children, 2011. A survey carried out on behalf of the Department of Health and Food Standards Agency, 2013.

[7] NATIONAL DIET AND NUTRITION SURVEY: Food and Drink Diary. 2010; Available from: https://webarchive.nationalarchives.gov.uk/20130413115649/http://media.dh.gov.uk/network/261/files/2012/07/Appendix-E_All-docs-combined-v2.pdf.

[8] 2011. Early Years Interventions To Address Health Inequalities In London – The Economic Case.[ebook] London: Greater London Authority. Available at:<https://www.london.gov.uk/sites/default/files/gla_migrate_files_destination/Early%20Years%20report%20OPT.pdf> [Accessed 5 January 2021].

[9] High S, Mckie I, Mackay M, et alO-09 Beyond traditional approaches to community engagement: using asset based community development to discover, connect and mobilise communities to support each other right to the end of lifeBMJ Supportive & Palliative Care 2022;12:A4.

[10] Lakhanpaul, M., 2019. PANCHSHEEEL - MRC HEEE STUDY. [online] Ucl.ac.uk. Available <https://www.ucl.ac.uk/child-health/sites/child-health/files/newsletter_3_0.pdf

[11] WHO. WHO recommendation on facilitated participatory learning and action cycles with women`s groups during pregnancy: The WHO Reproductive Health Library 2018 [from: https://extranet.who.int/rhl/topics/improving-health-system-performance/implementation-strategies/who-recommendation-facilitated-participatory-learning-and-action-cycles-womens-groups-during.

[12] Seward N, Neuman M, Colbourn T, Osrin D, Lewycka S, Azad K, et al. Effects of women's groups practising participatory learning and action on preventive and care-seeking

For peer review only

behaviours to reduce neonatal mortality: A meta-analysis of cluster-randomised trials. PLoS medicine. 2017;14(12):e1002467.

[13] Tripathy P, Nair N, Barnett S, Mahapatra R, Borghi J, Rath S, et al. Effect of a participatory intervention with women's groups on birth outcomes and maternal depression in Jharkhand and Orissa, India: a cluster-randomised controlled trial. The Lancet. 2010;375(9721):1182-92

[14] Prost A, Colbourn T, Seward N, Azad K, Coomarasamy A, Copas A, et al. Women's groups practising participatory learning and action to improve maternal and newborn health in low-resource settings: a systematic review and meta-analysis. The Lancet. 2013;381(9879):1736-46

[15] Mayor of London. London's socio-economic issues. 2016; Available at:https://www.london.gov.uk/sites/default/files/chapter10-economic-evidence-base-2016.pdf.Accessed 08/06/, 2020.

[16] ONS. 2011 Census Data. 2011; Available at: https://www.ons.gov.uk/census/2011census.

[17] Faculty of Dental Surgery. 2015. The state of children's oral health in England. [PDF]

[18] Newington L, Metcalfe A. Factors influencing recruitment to research: qualitative study of the experiences and perceptions of research teams. BMC Med Res Methodol. 2014;14:10. Published 2014 Jan 23. doi:10.1186/1471-2288-14-10

[19] Office for National Statistics. 2020. Domestic Abuse During The Coronavirus (COVID-19) Pandemic, England And Wales - Office For National Statistics. [online] Available at: <https://www.ons.gov.uk/peoplepopulationandcommunity/crimeandjustice/articles/domes ticabuse duringthecoronaviruscovid19pandemicenglandandwales/november2020> [Accessed 10 January 2021].

[20] Joseph RP, Keller C, Ainsworth BE. Recruiting Participants into Pilot Trials: Techniques Researchers with Shoestring Budgets. Calif J Health Promot. 2016;14(2):81-89.

[21] An innovative approach to recruiting homebound older adults. Crawford Shearer NB, Fleury JD, Belyea MRes Gerontol Nurs. 2010 Jan; 3(1):11-8.

[22] Llewellyn, C.H., et al., Development and factor structure of the Baby Eating Behaviour Questionnaire in the Gemini birth cohort. Appetite, 2011. 57(2): p. 388-396.

[23] Daniels, L.A., et al., Outcomes of an early feeding practices intervention to prevent

For peer review only

For peer review only - http://bmjopen.bmj.com/site/about/guidelines.xhtml

childhood obesity. Pediatrics, 2013. 132(1): p. e109-e118.

[24] Fogel, A., et al., Prospective associations between parental feeding practices and children's oral processing behaviours. Maternal & child nutrition, 2019. 15(1): p. e12635.

[25] Lakhanpaul M, Benton L, Lloyd-Houldey O, et al. Nurture Early for Optimal Nutrition (NEON) programme: qualitative study of drivers of infant feeding and care practices in a British-Bangladeshi population. BMJ Open 2020;10:e035347. doi:10.1136/ bmjopen-2019-035347

[26] Tla.mpi.nl. 2020. ELAN | The Language Archive. [online] Available at: http://tla.mpi.nl/tools/tla-tools/elan

[27] Health Do. Infant feeding recommendation. Department of Health London, England; 2003.

[28] 2019. Dental Record Keeping Standards: a consensus approach. [PDF] NHS England and NHS Improvement.

[29] Faculty of General Dental Practice, 2012. 7. Collecting Data. Available at: <https://www.fgdp.org.uk/research-primary-dental-care/7-collecting-data> [Accessed April 15, 2021].

[30] Lawrence, C.E. et al. A REDCap-based model for electronic consent (eConsent): Moving toward a more personalized consent. Journal of Clinical and Translational Science. 2020; 4(4):345–353.

[31] Bugge C, et al. A process for Decision-making after Pilot and feasibility Trials (ADePT): Development following a feasibility study of a complex intervention for pelvic organ prolapse. Trials.2013 Oct 25;14(1):353.

[32] Health Research Authority. 2018. HRA And MHRA Publish Joint Statement On Seeking And Documenting Consent Using Electronic Methods (Econsent). [online] Available at: <https://s3.eu-west-2.amazonaws.com/www.hra.nhs.uk/media/documents/hra-mhra-econsent-statement-sept-18.pdf> [Accessed 8 January 2021]

For peer review only - http://bmjopen.bmj.com/site/about/guidelines.xhtml

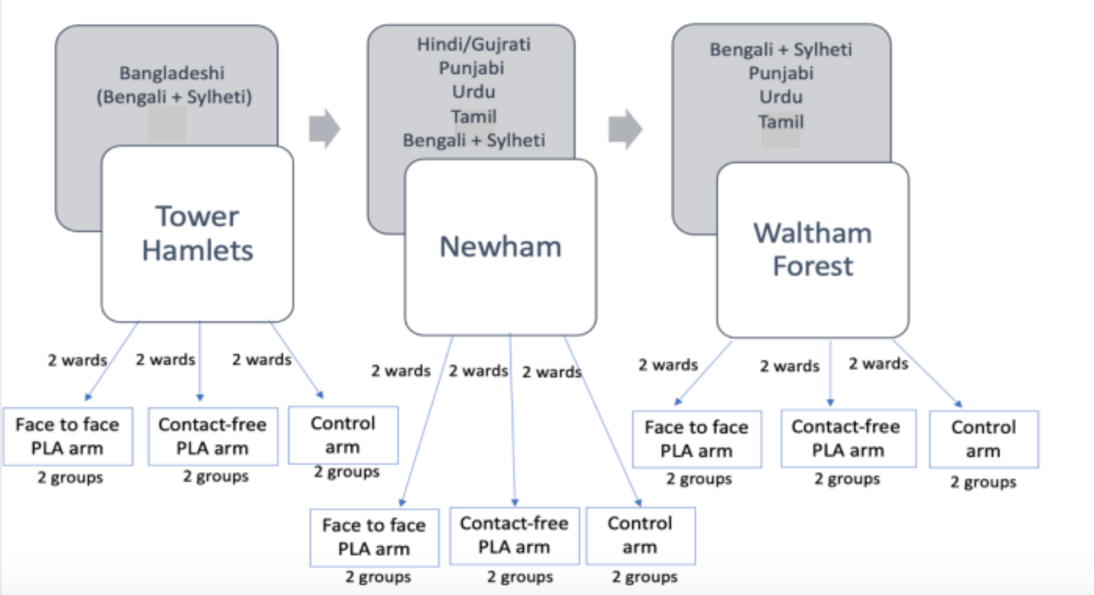

Figure 1. Overview schematic detailing number of wards & PLA groups by East London boroughs

Footnote: To illustrate, TH will have 2 face-to-face Bangladeshi/ Bengali & Sylheti PLA groups (1 per ward), 2 groups of Bangladeshi/ Bengali & Sylheti online PLA group (1 per ward), and 2 Bangladeshi/ Bengali & Sylheti control groups (1 per ward). The more the ethnic/ language group in the borough, the more the intervention and control groups.

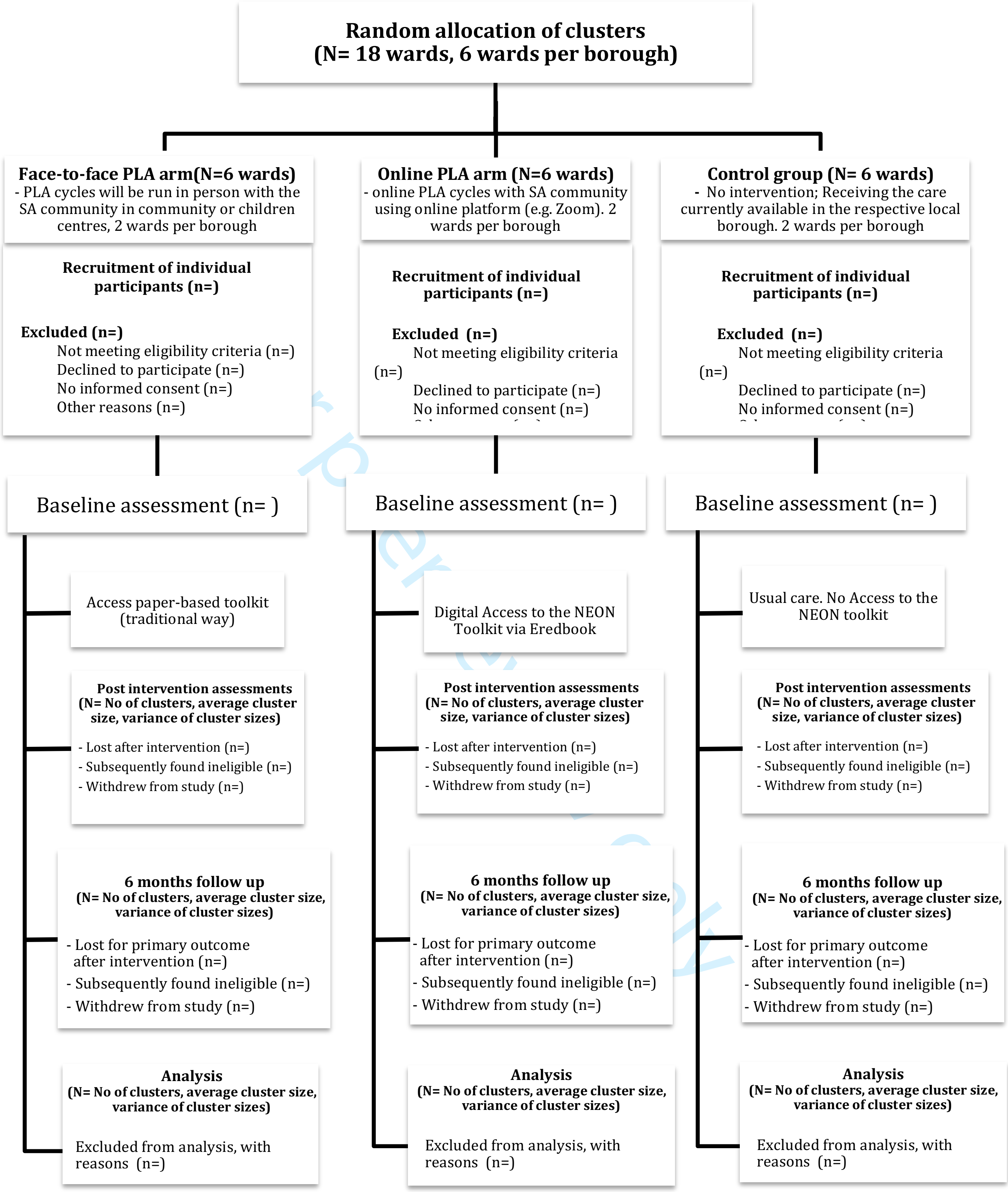

Figure 2. Overview of the NEON pilot feasibility RCT design and participant flow

For peer review only - http://bmjopen.bmj.com/site/about/guidelines.xhtml

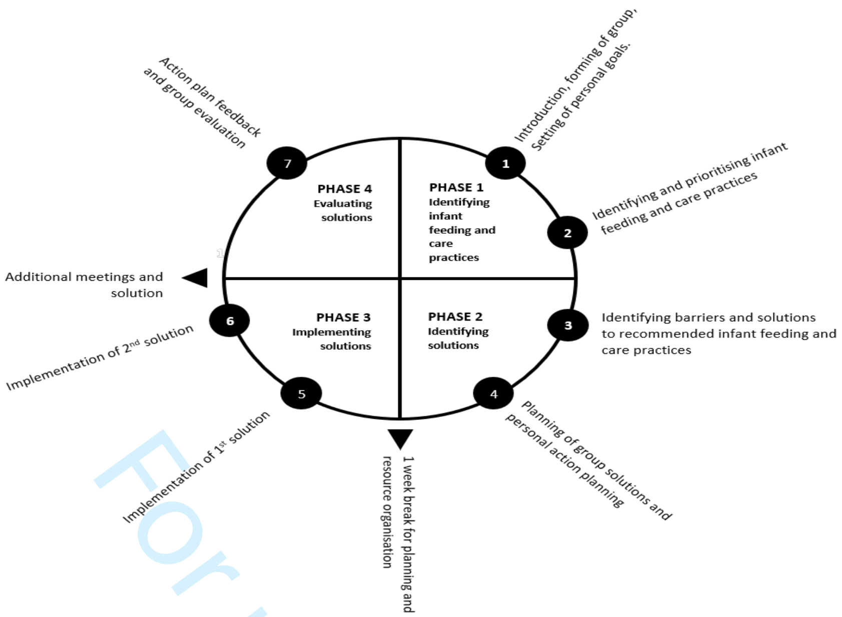

Figure 3. NEON Women's Group PLA Cycle

For peer review only

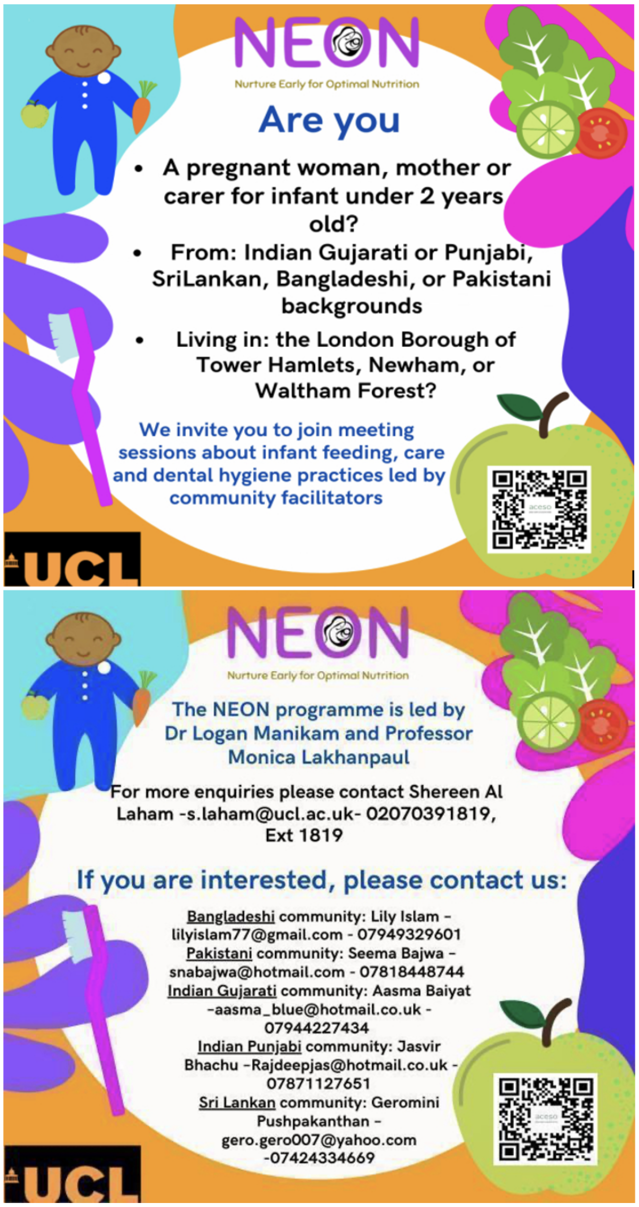

125x234mm (144 x 144 DPI)

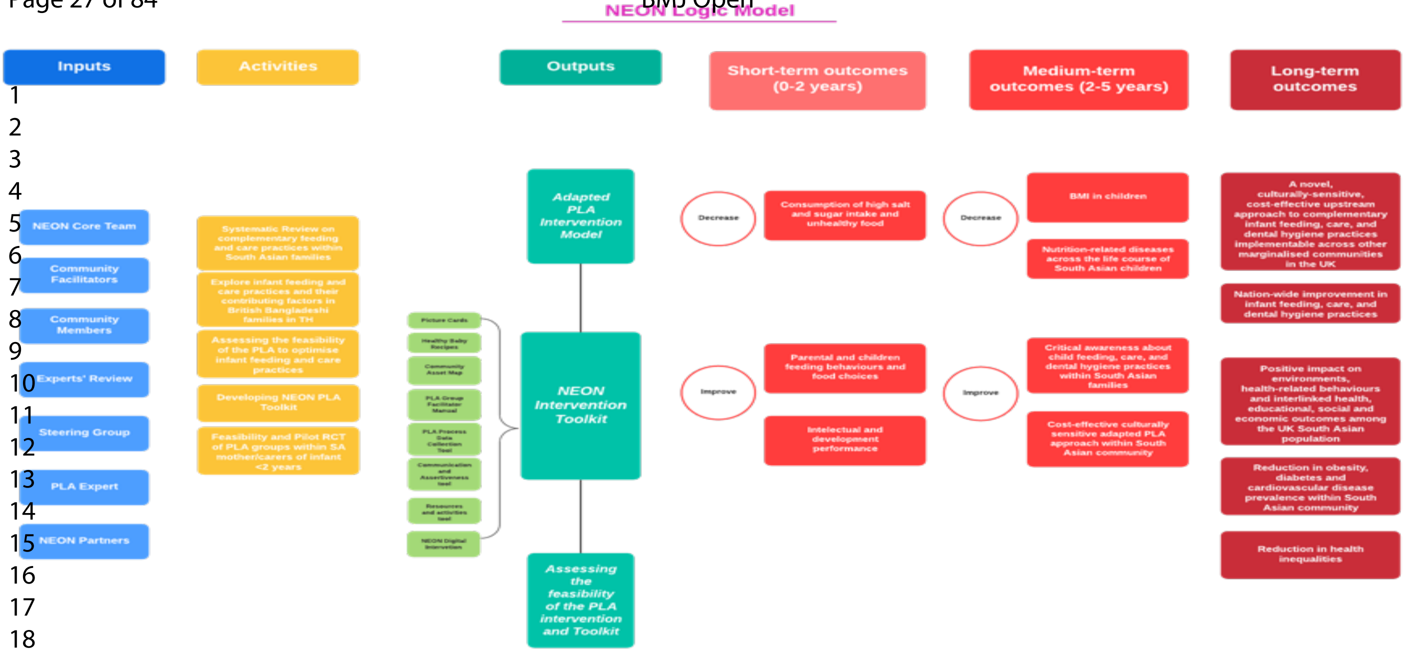

NEON Logic Model
Inputs
Activities
Outputs
Short-term outcomes (0-2 years)
Medium-term outcomes (2-5 years)
Long-term outcomes
NEON Core Team
Community Facilitators
Community Members
Experts' Review
Steering Group
PLA Expert
NEON Partners
Systematic Review on complementary feeding and care practices within South Asian families
Explore infant feeding and care practices and their contributing factors in British Bangladeshi families in TH
Assessing the feasibility of the PLA to optimise infant feeding and care practices
Developing NEON PLA Toolkit
Feasibility and Pilot RCT of PLA groups within SA mother/carers of infant <2 years
Picture Cards
Healthy Baby Recipes
Community Asset Map
PLA Group Facilitator Manual
PLA Process Data Collection Tool
Communication and Assertiveness tool
Resources and activities tool
NEON Digital Intervention
Adapted PLA Intervention Model
NEON Intervention Toolkit
Assessing the feasibility of the PLA intervention and Toolkit
Decrease
Consumption of high salt and sugar intake and unhealthy food
Improve
Parental and children feeding behaviours and food choices
Intellectual and development performance
Decrease
BMI in children
Nutrition-related diseases across the life course of South Asian children
Improve
Critical awareness about child feeding, care, and dental hygiene practices within South Asian families
Cost-effective culturally sensitive adapted PLA approach within South Asian community
A novel, culturally-sensitive, cost-effective upstream approach to complementary infant feeding, care, and dental hygiene practices implementable across other marginalised communities in the UK
Nation-wide improvement in infant feeding, care, and dental hygiene practices
Positive impact on environments, health-related behaviours and interlinked health, educational, social and economic outcomes among the UK South Asian population
Reduction in obesity, diabetes and cardiovascular disease prevalence within South Asian community
Reduction in health inequalities

RESOURCES AND SERVICES SUPPORTING INFANT FEEDING, CARE AND DENTAL HYGIENE

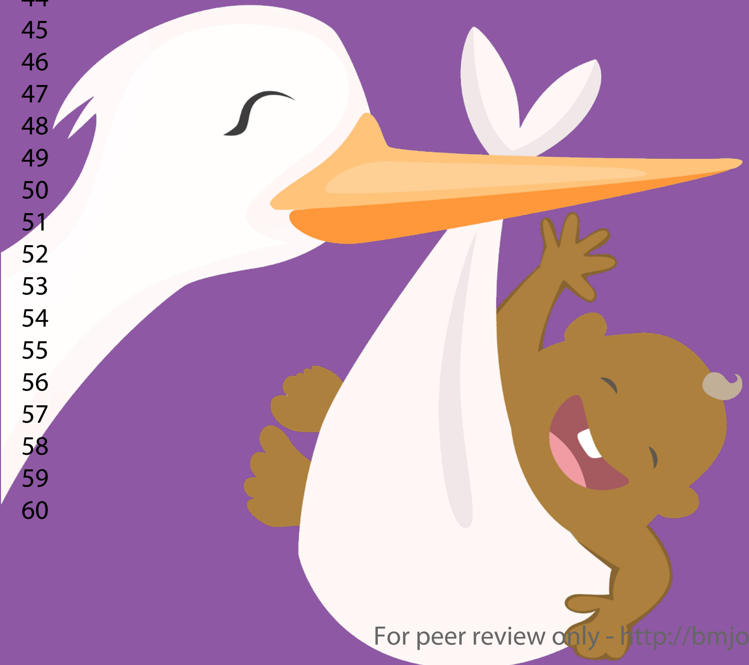

NEON
Nurture Early for Optimal Nutrition

NHS AND COUNCILS ADVICE AND SUPPORT

1. NHS STAR4LIFE

https://www.nhs.uk/start4life

Website provides trusted NHS help and advice for parents and carers during pregnency, and birth and the early years.

2. NHS CHOICES

NHS Choices (www.nhs.uk) is the official website of the National Health Service in England and provides comprehensive health information service with thousands of articles, videos and tools. Key pages which give guidance on infant feeding and care include:

- **Your newborn:** *https://www.nhs.uk/conditions/pregnancy-and-baby/childrens-meal-ideas/?tab-name=your-newborn*
- **Babies and toddlers** *https://www.nhs.uk/conditions/pregnancy-and-baby/childrens-meal-ideas/?tabname=ba-bies-and-toddlers*

3.CHILDREN CENTRES AND LIBRARIES

Tower Hamlets has 12 children centres across the borough providing a range of services to infants, children and families to give them the support they need to be safe, healthy and happy, so that they are able to reach their full potential. This web page on the **Tower Hamlets Council** website gives locations and contact details of all the children's centres in the borough: *https://www.towerhamlets.gov.uk/lgnl/education_and_learning/childcare_and_early_years_educ/Children_centres/childrens_centres.aspx*

The borough of Newham offers support to its residents with children under the age of 5 through **Best Start in Life (BSiL)** Children's centres. These include play activities and childminder services, as well as supporting parents to improve their confidence, support their children with healthy eating and weaning. Libraries offer free courses and internet services for parents, and they offer devices and digital equipment to borrow.

Locations and details can be found on the Families Newham website: *https://families.newham.gov.uk/kb5/newham/directory/family.page?familychannel=3-2*

Waltham Forest offers to its families a similar service, with support for healthy development and access to financial support. They have implemented the 2 Year Old Partnership Pathway in which professionals empower and inform parents to assure the best start in life for their children. They offer advice on a wide range of issues including health, finance and cognitive/motor development. **Waltham Forest** offers support directed at healthy weight and eating through HARRY.

More information can be accessed via the following link: *https://www.walthamforest.gov.uk/content/2yo-partnership-pathway*

Further informal support that does not require formal registration can generally be accessed in public libraries

For peer review only

GUIDENCE DOCUMENTS, ONLINE RESOURCES AND APP'S

1. BABY FRIENDLY INITIATIVE

Published by the Department of Health and the Baby Friendly Initiative, the leaflets below provide mothers and health professionals with key information about breast feeding, introducing solid foods to babies, and evidence-based guidance around formula. The **Baby Friendly Initiative** is part of a global partnership between the World Health Organization (WHO) and UNICEF. The guidance below should be prioritised over other online resources (other than NHS Choices) as they the baby friendly initiative is less influenced by commercial interests, which can sometimes influence the advice offered to mothers and parents regarding infant feeding and care.

- **Leaflet on weaning and starting solid food:** https://www.unicef.org.uk/babyfriendly/baby-friendly-resources/leaflets-and-posters/weaning-starting-solid-food/
- **Leaflet on breastfeeding:** https://www.unicef.org.uk/babyfriendly/baby-friendly-resources/leaflets-and-posters/off-to-the-beststart/ -
- **Leaflet on infant formula:** https://www.unicef.org.uk/babyfriendly/baby-friendly-resources/leaflets-and-posters/simple-formula-guide-for-parents/
- **Leaflet on bottle feeding:** https://www.unicef.org.uk/babyfriendly/baby-friendly-resources/leaflets-and-posters/guide-to-bottle-feeding/
- **Baby friendly initiative resources in Bengali:** https://www.unicef.org.uk/babyfriendly/baby-friendly-resources/leaflets-and-posters/foreign-language-resources/bengali-resources/
- **Baby friendly initiative resources in Hindi:** बच्चे के अनुकूल पहल संसाधन https://www.unicef.org.uk/babyfriendly/baby-friendly-resources/foreign-language-resources/hindi-resources/
- **Baby friendly Initiative resources in Urdu:** لئاس وےک(تشادہگن(ءامنوشن ئادتبای ک ےچب https://www.unicef.org.uk/babyfriendly/baby-friendly-resources/foreign-language-resources/urdu-resources/

Other Baby Friendly Initiative resources/leaflets:

https://www.unicef.org.uk/babyfriendly/baby-friendly-resources/leaflets-and-posters/

2. FIRST STEPS NUTRITION TRUST

https://www.firststepsnutrition.org

First steps nutrition can also be used as a trusted source of good information and guidance regarding nutrition and feeding in pregnancy, infants, and early years. **First Steps Nutrition Trust** is funded by research or charitable grants and donations, and does not take money from any commercial organisation or any organisation or individual linked to the sales of infant formula and other food or drink product or service which is associated with poor nutritional health of women and children.

For peer review only

First Steps Nutrition Trust offers some useful resources on meal choices and portion sizes, with helpful diagrams to illustrate portion size. Remember! Portion size is a maximum, not a minimum, never encourage a child to eat more when they indicate they have had enough.

- Eating well: the first year – guidance on introducing foods, food choices and meals for up to 7 months, 7-9 months, and 10-12 months: http://www.firststepsnutrition.org/newpages/Infants/first_year_of_life.html
- Eating well: Early years – guidance on food choices, meals, snacks and portion size http://www.firststepsnutrition.org/newpages/Early_Years/eating_well-early_years.html

3. BORN TO MOVE APP

Born to move is a free NHS app to help parents or carers with their newborn babies right up to pre-school, specifically relating to physical activity and play. It's packed with advice, tips and games that you can play with your child to support their development.

https://itunes.apple.com/gb/app/born-to-move/id976095022?mt=8Local%20initiatives/projects

https://play.google.com/store/apps/details?id=com.project.Born&hl=en_GB

For peer review only

4. EREDBOOK

ERedbook is an app based digital version of the paper Redbook. It provides solutions for parents that allows them to review their child's NHS records whilst providing health information and guidance from NHS and other sources. Additionally, it contains personalised information for new parents, including relevant videos and information from local sources & reminders and appointments updates. Parents can review the NHS child's health records on the app when they connect record to NHS. Also, The NEON participants will be able to review the NEON toolkit and receive correct information via the ERedbook app at any time and place.

https://www.eredbook.org.uk

https://apps.apple.com/gb/app/eredbook/id606443658

https://play.google.com/store/apps/details?id=com.sitekit.eRedBook&hl=en_GB

OTHER SOLUTION IDEAS TO SUPPORT PLAY AND ACTIVITY:

1. Develop activity and play schedules for you and your infant with guidance from our local children centre or health visitor and make time for these activities.

2. Download the 'born to move' app for access to information and guidance on infant activity and play.

3. Designate a play area (even if this needs to be set-up and put away at times of day) for your infant in the home.

4. Arrange play and physical activity sessions together as a group, or with other parents, extended family, carers and infants – this could help with support and confidence in exercising and conducting physical activity in public spaces.

OTHER LOCAL INITIATIVES, CHARITIES AND PROJECTS IN EAST LONDON

1. BREASTFEEDING NETWORK

Find your nearest breast feeding drop-in-group in Tower Hamlets
https://www.breastfeedingnetwork.org.uk

There are groups run at Shadwell Children's Centre, Chrisp Children's Centre, Overland Children's Centre, Collingwood Children's Centre, Wapping Children's Centre and many more. If you need information or support with breastfeeding in the evenings or at weekends please call:

National Breastfeeding Helpline: *0300 1000 212*

BfN Support in Bengali/Sylheti: *0300 456 2421*

Open from *9.30am - 9.30pm 365 days a year*

Newham: https://www.nct.org.uk/local-activities-meet-ups/region-london/newham/breastfeeding-support

Tower Hamlets: https://www.breastfeedingnetwork.org.uk/tower-hamlets/

Waltham Forest offers a team of infant feeding practitioners delivering 1:1 sessions, a helpline and at home visitors to support the breastfeeding family through HENRY.

For information:
Email: wfsupport@henry.org.uk
Telephone: 020 8496 5223

2. DENTAL SERVICES AND RESOURCES

Dentists across Tower Hamlets, Waltham Forest, and Newham offer free dental check-ups, which you can attend as soon as your child's teeth first appear.

To find your nearest dental practice in Tower Hamlets, please call 020 7364 5000.

HENRY has put together some tips for healthy teeth in young children and a list of Waltham Forest dentists currently accepting new patients from birth: https://www.henry.org.uk/sites/www.henry.org.uk/files/inline-files/Healthy%20Teeth%20Leaflet%20%28April%29_0.pdf

The Kent Community Health NHS Foundation Trust has compiled some online oral health promotion resources for parents here: https://www.kentcht.nhs.uk/service/dental-services/oral-health-promotion-resources/

The Kent Community Health NHS Foundation Trust has compiled some online oral health promotion resources for parents here: https://www.kentcht.nhs.uk/service/dental-services/oral-health-promotion-resources/

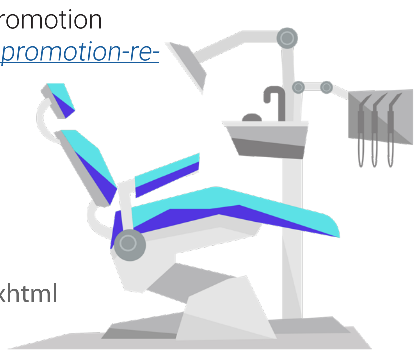

For peer review only

Here are examples of dental services by borough (to find out more, please see the community asset map):

Tower Hamlets

- Fresh Springs Dental Practice
  T: 020 3216 0077
  E: reception@freshspringsdentalpractice.co.uk

- Together Dental Whitechapel
  T: 020 7247 4600
  E: whitechapelreception@together.dental

Waltham Forest

- Abbey Dental Practice
  T: 020 8521 2816
  E: info@abbeydentalwalthamstow.co.uk

- Inspire Dental Walthamstow
  T: 020 8521 6656
  E: info@inspiredentalwalthamstow.co.uk

3. MATERNITY MATES

http://www.whfs.org.uk/index.php/what-we-do/maternity-mates

Maternity Mates™ is a project run by the charity Women's Health and Family Services which recruits, trains and match-ups volunteer Maternity Mates with pregnant women in need of extra support in Tower Hamlets. A Maternity Mate is a female volunteer trained by WHFS to provide practical and emotional support to women during pregnancy, childbirth and the early weeks of motherhood. Support can start any time from the 5th month of pregnancy, through to birth and up to either 6 or 12 weeks after the baby is born (6 weeks in Tower Hamlets and 12 weeks in Newham). **Maternity Mates** are recruited from the communities they serve, and where possible, will speak the same language as the mother-to-be.

For further information, please contact:

Jasmin Begum - Programme Manager
M: *07960 327 760*
T: *020 7377 9640*
E: *jasmin.begum@whfs.org.uk*

For information about the project in Tower Hamlets and Waltham Forest, please contact:

Rubi Rodriguez - Project Co-ordinator Tower Hamlets and Waltham Forest
M: 07496 764 881
E: rubi.rodriguez@whfs.org.uk

For information about the project in Newham, please contact:

Irantzu Perez Arribas - Project Co-ordinator Newham
M: 07436 139 235
E: irantzu.perez@whfs.org.uk

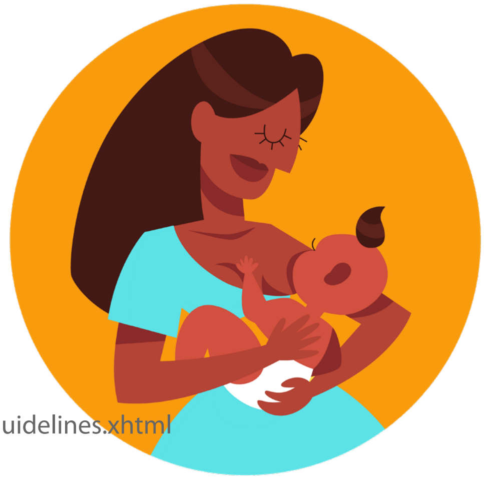

For peer review only

4. BOUNTY

https://www.bounty.com/about-bounty/bounty-packs/newborn-pack

Bounty provides mothers and pregnant women with free subscription packs including advice from Public Health England, as well as a variety of sample sized baby products (nappies, baby formula etc.) and coupons.

Every mother with a Newborn will receive a Newborn Pack at the hospital after delivery, and more freebies and advice are available through the Bounty app.

5. EMMA'S DIARY

https://www.emmasdiary.co.uk
Contact: 01628 535 483 Mon-Fri 9am to 4:30pm

Emma's Diary is a free to access resource created by a board of GPs and midwives that provide information ranging from trying to get pregnant from wellbeing information during and post-pregnancy. There's a free to download mobile app to allow you to access information more easily, tailored towards recent mothers and baby related advice and offers.

6. THE TOYHOUSE CENTRE

http://www.toyhouse.org.uk/

Phone: 0020 7987 7399
E-mail: info@toyhouse.org.uk

Toy House is a local charity in Tower Hamlets which runs projects and services aimed at support play and wellbeing of infants, children and families.

Toy Libraries
http://www.toyhouse.org.uk/toy-libraries/
The charity has its own toy library, and has information and links to over 19 toy libraries and stay and play sessions run across Tower Hamlets.

7.MEND - MIND, EXERCISE, NUTRITION...DO IT!

https://www.mytimeactive.co.uk/mend

MEND is a programme which empowers mums, children and adults to become fitter, healthier and happier and to reach or maintain a healthier weight. It's free to attend and run by local teams in local communities, after school or at weekends. MEND have a range of programmes for different ages run in different boroughs across London.

MEND Mums is designed for post-natal women with a baby up to 2 years and a BMI of 25 or above, **MEND** Mums is a fun and interactive weight management programme incorporating energy boosting workouts and great nutrition tips to help establish healthy habits for life.

For more information about any of the MEND programmes in Tower Hamlets call:
020 8323 1725

For peer review only

8. MIND

MIND is a mental health charity with a specific branch in Newham and Tower Hamlets that offers free mental health services talking therapies, COVID19 Support and Advocacy services

https://www.mithn.org.uk
Phone: 020 7510 1081
Email: info@mithn.org.uk

9. REBECCA CHEETHAM NURSERY & CHILDREN'S CENTRE

Website: https://www.rebeccacheetham.newham.sch.uk
Phone:
Email:

The Rebecca Cheetham Children's Centre offers free child-minding services as well as advice and support for families with young children. However they have temporarily stopped face to face sessions in light of the COVID pandemic. Limited online services are still running for examples: a Family Support Team that can refer you to foodbanks.

OTHER IDEAS AND SUGGESTIONS

- **Breakfast clubs** – these are available in most schools to give children a healthy start to the day. Speak to your local school. Due to COVID19 Restrictions, these breakfast clubs have moved to.
- Despite this, virtual coffee mornings are ongoing

- **Social support** – if you feel you need more support from other mothers, parents or people local to you, you can attend local coffee mornings, toddler groups and parent forums. E.g. One stop shop in Newham and Tower Hamlets. Father examples would be the "idea Store" which is based in Tower Hamlets and offers a range of free activities and educational services for children and families alike. http://www.healthwatchnewham.co.uk/sites/default/files/newham_domestic_abuse_one-stop-shop.pdf

- **Triple P – Positive Parenting Programme** – is an evidence based programme aimed at supporting parents develop relationships with their children.

  - Newham offers a Parenting Befriending service and bookable workshops accessible via the following website: https://families.newham.gov.uk/kb5/newham/directory/family.page?family-channel=1-1

  - Tower Hamlets offers a similar service: https://www.towerhamlets.gov.uk/lgnl/educa tion_and_learning/parental_support/parenting_programmes.aspx
    parenting@towerhamlets.gov.uk.

  - Waltham Forest offers a series of 6 sessions for parents with children aged 3 and over. https://www.walthamforest.gov.uk/content/parenting-support

    Call: *020 8496 2442*
    Email: *earlyhelpparenting@walthamforest.gov.uk*

- **Tunmarsh Centre**– support for parents with children with special needs
  The New Tunmarsh Centre based in Newham provides free education for children with ASD / DHD and support for their families. Parents are regularly invited for coffee mornings with speakers.

  E-mail: Faz.Mac@gmail.com

For peer review only

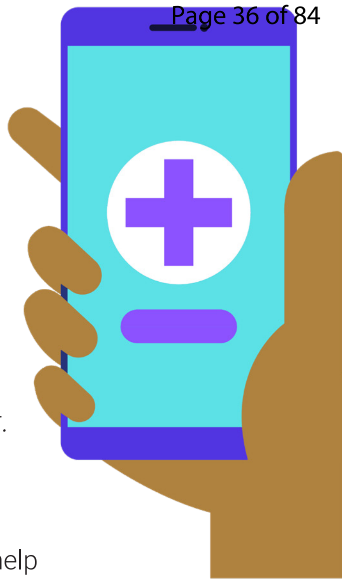

- **John Smith Children's Centre**

  Telephone: 0207 364 0537
  Contact: Deborah Wooding
  Email: deborah.wooding@towerhamlets.gov.uk
  Based in Tower Hamlets, The John Smith Children's Centre that offer childcare support.

- **The Trinity Centre**
  Website: www.thetrinitycentre.org/home/
  The Trinity Centre offers a day care nursery as well as independent groups and faith groups to help support parents form minority and marginalised communities

- **Around Poplar Children's Centre**

  Contact: Brenda Pascal
  T: 020 7364 0540
  E: Brenda.pascal@towerhamlets.gov.uk

  The Around Poplar Children's Centre offers childcare, family, health services, and early education support for families with children under 5 free of charge.

  For a list of similar children's centres in Tower Hamlets, see here:
  https://www.localoffertowerhamlets.co.uk/organisations?utf8=%E2%9C%93&search_organisation%5Bterm%5D=&search_organisation%5Blocation%5D=&search_organisation%5Blatitude%5D=&search_organisation%5Blongitude%5D=&search_organisation%5Bcategory_ids%5D%5B%5D=3308

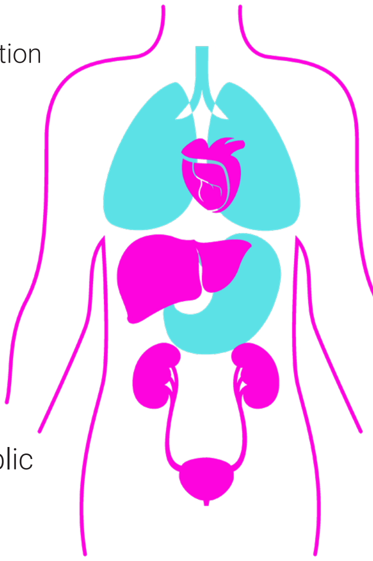

- **Idea Store**

  Website: www.ideastore.co.uk
  Branches of the Idea Store in Tower Hamlets offer library services and access to public computers for families and individuals free of charge.

- **Outreach centre** Outreach centres are open till 6 pm where you can drop your children there, and it is free for people who are entitled for public funds. They offer: Child minding, after school club, breakfast club for parents and children <3 years. Key examples would be Community Centres and Children's Centres.

EXAMPLES:

Newham Council: https://www.newham.gov.uk/homepage/115/community-centres
Tower Hamlets: Community and day centres (towerhamlets.gov.uk)

Plaistow Children's Centre
Website: https://plaistow.newham.sch.uk/Plaistow-Children-s-Centre/

The Plaistow Children's Centre is based in Newham and offers care and support for young children ranging from 0-5 years for 49 weeks within the year led by staff and volunteers. Other services range from nutrition webinars to overall family support and advice. The Children's Centre is still open during the pandemic and offers slightly modified services as a result.

For peer review only

OTHER SUPPORT

1. Request interpretation support for GP visits from Tower Hamlets GP care group.

2. Advocacy & Interpreting Service in Newham (https://www.gpcaregroup.org/section/455/Services/page/eb745e07-85ab-4cee-bc8f-c880a-d400a95/Advocacy-Interpreting)

3. Provide feedback on services or lodge a complaint via Health Watch Tower Hamlets. More information can be accessed here: https://www.healthwatchtowerhamlets.co.uk/

FOOD BANKS:

Suggested Food Bank locations: https://www.google.com/maps/d/edit?mid=1ZyqIE2-HgdQ-

NEON Community Asset Map: https://www.google.com/maps/d/edit?mid=1ZyqIE2-Hg-dQ-Hzow7KwhAiYsdtQnP_Zq&usp=sharing

- **Newham Food Alliance**

  Website: https://www.newham.gov.uk/newhamfoodalliance

  The Newham Food Alliance is a food bank based in Newham that can offer access to food for those who are struggling financially. There is also delivery support they can provide to those self-isolating due to COVID and providing food remotely during the pandemic regardless.

- **Church Food Banks:**

  Website: www.churchesfoodbank.org.uk/

  Food Banks located in the Borough of Newham. To access these you need to be referred from another agency such as a school, advice centres or your GP.

The 2 main sites are:

Woodgrange Baptist Church

345 Romford Road,Forest Gate,E7 8AA

0208 555 9880

St Paul's Church

65 Maryland Road,Stratford,E15 1JL

0208 534 1164

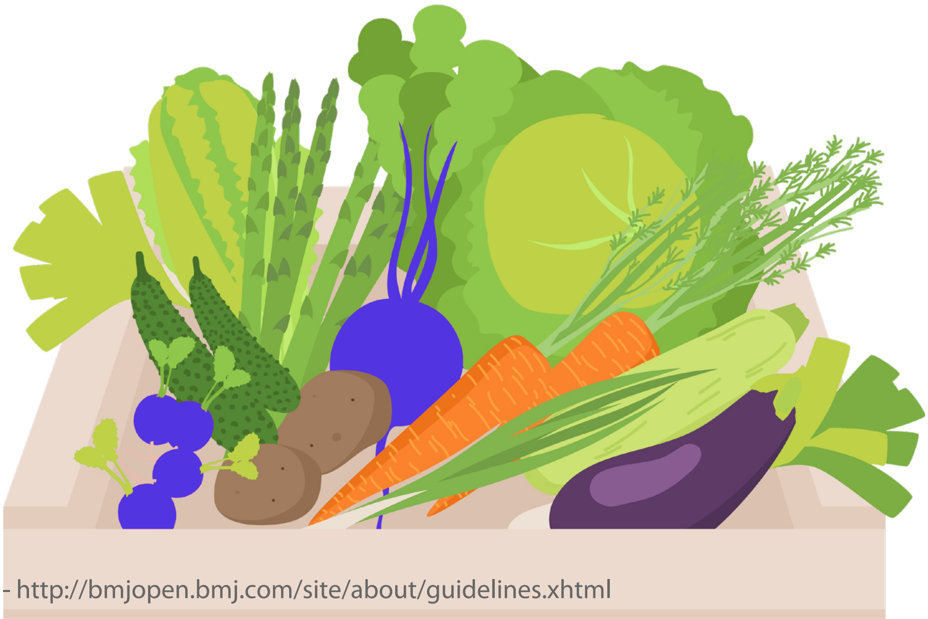

For peer review only

o The Trussell Trust

Website: https://www.trusselltrust.org

The Trussell Trust is a charity that provides nationwide network of food banks as well as emergency food for those in need. They also run regular campaigns to further increase support for people who need access to regular foods but cannot afford to do so. The website is useful as it flag foodbanks that are local to you.

o First Love Foundation Foodbank

Website: www.firstlovefoundation.org.uk/

The First Love Foundation coordinates the Tower Hamlets Foodbank. It provides daily necessities to families in need and alongside this, operates as a crisis service.

o Eat or Heat - Waltham Forest Food Bank

Website: http://eatorheat.org

Phone Number: 0800 772 0212

Email: referrals@eatorheat.org

A charity based in East London that supports local families. On the website, different professionals who can refer you to this service are listed.

o Rukhsana Khan Foundation

Website: https://www.rukhsanakhanfoundation.org

Phone Number: 07939 232 123

Email: rukhsanakhanfoundation@outlook.com

Rukshana Khan Foundation is a local charity that supports families in East London. They run food parcels and deliveries for those on benefits and those referred from Citizens Advice, NHS, Local Doctor's Surgery, Job Centre, Baby Food Bank, or the Council. They are also supporting those classed as clinically vulnerable. You can register for support on their website.

They run a foodbank on Saturdays 11am-12pm at the William Morris Community Centre.

o Waltham Forest Liberal Democrats

Website: https://www.walthamforestlibdems.org/local_support_for_families_in_need

The Waltham Forest Liberal Democrats have collated a set of resources highlighting local foodbanks with Waltham Forest that local families can access

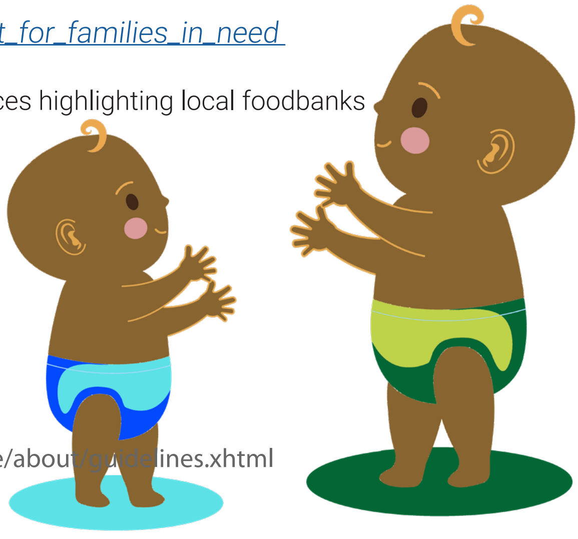

For peer review only

- **Eden Girls' School, Waltham Forest**

  A local food bank based at Eden Girls' School that provides food to local people from 1-3pm every Friday

  Contact Details:

  Eden Girls' School, Waltham Forest

  Silver Birch and Landmark Houses,

  Blackhorse Lane, Walthamstow

  London, E17 5SD

  Tel: 0208 523 1810

  Email: info@egwf.staracademies.org

- **Community Food Projects in Tower Hamlets**

  https://www.wen.org.uk/2020/03/30/https-www-wen-org-uk-2020-03-30-information-on-local-response-to-covid-19/

- **Healthwatch groups available in different boroughs:**

  Newham http://www.healthwatchnewham.co.uk/

  Tower Hamlets https://www.healthwatchtowerhamlets.co.uk/

  Redbridge http://www.healthwatchredbridge.co.uk/

  Waltham Forest https://www.healthwatchwalthamforest.co.uk/

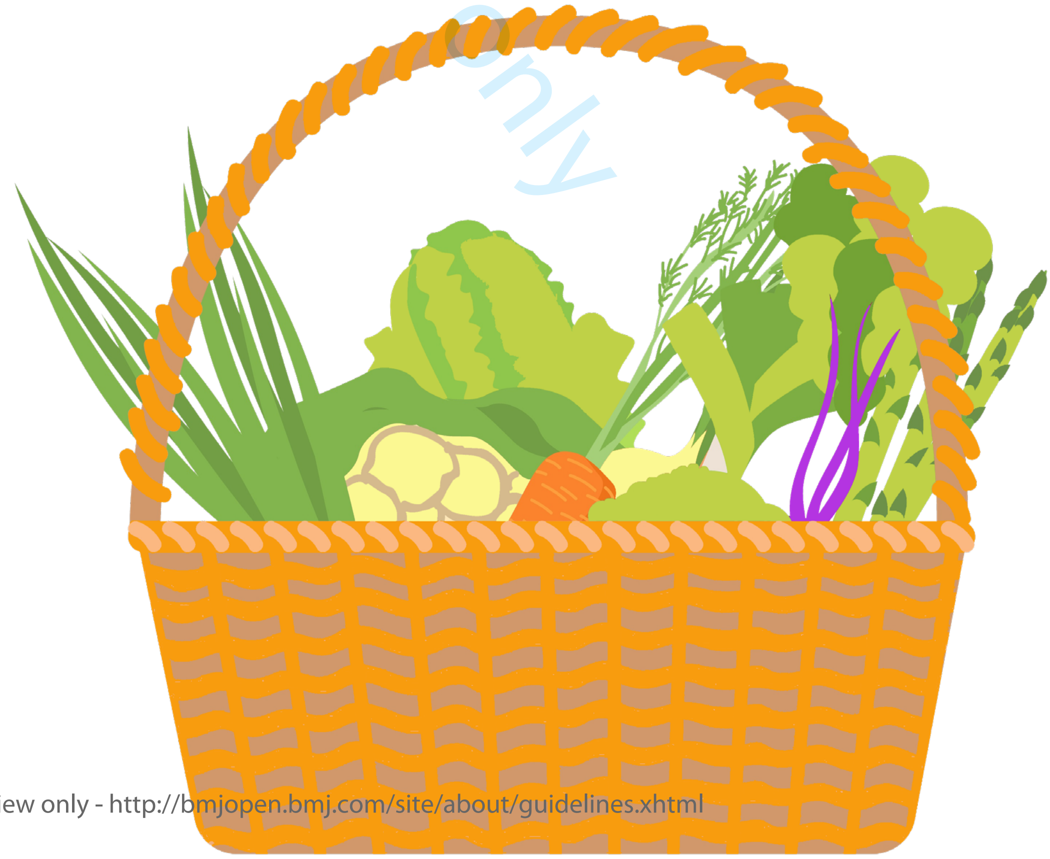

For peer review only

Participants Feedback Questionnaire

1. What were your reasons for wanting to attend the group meetings?

2. What made you come back after attending a meeting? /or, what was your reason for not coming back to later meetings?

i. *What made you keep coming back to the meetings? /Or, what could we have done differently to motivate you to come back to the next meeting?*

3. Did the group manage to meet your expectations?

i. *how did it meet your expectations?*
ii. *If it did not, why was this the case?*

4. What could be improved if this project was run again?

i. *How could we improve the content (meeting exercises)*
ii. *How could we improve the tools (picture cards / infant feeding resources)*
iii. *How could we improve the tailoring to your needs as British Bangladeshi mother/pregnant women/or grandmother*
iv. *Would it be beneficial to have picture cards and other project materials written in Bengali (with the picture on the front and Bengali text on the back)*
v. *Is there any additional information or resources you would have liked to have been offered in the meetings?*

5. How involved did you feel in the learning process of the group?

i. Did you feel you worked together as a group during the meetings? How so?
ii. Were there any barriers you experienced to participating?
iii. Were you given opportunities to share experiences? Please give examples.

6. Are you in contact with any of the other participants outside of the group meetings?

i. Have you discussed any issues raised in the group meetings?
ii. Have you supported each other on any issues raised in the group meeting?

9. Have you found new people, groups or sources of information as a result of attending the group meetings? Please give examples.

10. Did you share any information from the group meetings with any other members of your family or community outside of the group?

11. If we were to implement a full project using this approach, the next stage would involve more group meetings (possibly up to 8 additional meetings) where by the group would support each other to implement some of the solutions you identified and then after implementing

For peer review only - http://bmjopen.bmj.com/site/about/guidelines.xhtml

solutions, evaluate their success. Do you think this is something you or Bangladeshi carers would be willing to undertake or partake in?

i. *Can you think of any barriers to using this approach we need to consider at this stage?*
ii. *How could additional meetings best support you to implement the solutions you identified, and introduce the recommended infant feeding and care practices? What should they focus on?*
iii. *What additional meetings would be acceptable to you and feasible to attend?*

12. Is there anything else you would like to share about your experience on the project?

For peer review only

For peer review only - http://bmjopen.bmj.com/site/about/guidelines.xhtml

Facilitator Report Form

**PLA GROUP FACILITATOR REPORT - PLA group meeting xx**					
Group location/name (XX children's centre group)		Meeting number		Me etin g dat e	
Facilitator name					
Number of picture cards discussed					
Did you manage to go through all questions on the picture cards?<br>• *What does the picture show?*<br>• *Clarify which practice is being discussed*<br>• *Ask and explain how this affects infant growth and development*<br>• *Use info on the back of the card to ask questions about the practice and probe*<br>• *Ask if this occurs in the community? Is it common?*<br>Were there any barriers to a full discussion on the above questions?					
Were there any picture cards not discussed at all?					

For peer review only - http://bmjopen.bmj.com/site/about/guidelines.xhtml

Which exercises were completed during your meeting?	
What was the outcome of each exercise?	
How long did the meeting run for?	
Did anything go well/did anything not go well?	
GROUP FACILITATOR TO COMPLETE AFTER EACH MEETING CONDUCTED.	

**PLA GROUP FACILITATOR REPORT – PLA group meeting 3**					
Group location/name (XX children's centre group)		Meeting number		Me etin g dat e	
Facilitator name					
Number and type of picture cards discussed					
Did you manage to go through all the probing questions on the picture cards?<br>• *What does the picture show?*<br>• *Clarify what barrier is being discussed*<br>• *Ask and explain how this barrier may affect or influence behaviour relating to infant feeding and care*					

peer review only

• *Ask which infant feeding and care practice the barrier may prevent parents from practicing?*<br><br>• *Ask if the barrier exists in the community*<br>Were there any barriers to a full discussion on the above questions?	
Were there any picture cards not discussed at all?	
Which exercises were completed during your meeting?	
What was the outcome of each exercise?<br><br>Which barrier cards were identified for prioritised practice 1?<br><br>Which barrier cards were identified for prioritised practice 2?<br><br>Which barrier cards were identified for prioritised practice 3?	
How long did the meeting run for?	
Did anything go well/did anything not go well?	
GROUP FACILITATOR TO COMPLETE AFTER EACH MEETING CONDUCTED.	

For peer review only

For peer review only - http://bmjopen.bmj.com/site/about/guidelines.xhtml

PLA GROUP FACILITATOR REPORT – PLA group meeting 4					
Group location/name (XX children's centre group)		Meeting number		Me etin g dat e	
Facilitator name					
Number and type of picture cards discussed					
Did you manage to go through all the probing questions on the picture cards?<br>• *What does the picture show?*<br>• *Ask participants to identify what solution they are discussing and what non-recommended infant feeding and care practice the solution helps to reduce or manage.*<br>• *Ask participants to suggest ideas on what activities the group (or they, as individuals) could undertake to achieve this solution. Use the information on the back of the card to suggest ideas on activities which could be undertaken. Ask if the barrier exists in the community*<br>• Were there any barriers to a full discussion on the above questions?					
Were there any picture cards not discussed at all?					
Which exercises were completed during your meeting?					

For peer review only - http://bmjopen.bmj.com/site/about/guidelines.xhtml

What was the outcome of each exercise?<br><br>Which barrier solution cards were identified for prioritised practice 1<br><br>Which management solution cards were identified for prioritised practice 1<br><br>Which barrier solution cards were identified for prioritised practice 2<br><br>Which management solution cards were identified for prioritised practice 2<br><br>Which barrier solution cards were identified for prioritised practice 2<br><br>Which management solution cards were identified for prioritised practice 2	
How long did the meeting run for?	
Did anything go well/did anything not go well?	
GROUP FACILITATOR TO COMPLETE AFTER EACH MEETING CONDUCTED.	

For peer review only - http://bmjopen.bmj.com/site/about/guidelines.xhtml

Sustainability assessment

1. PARTICIPATION

Criteria for assessment	*Are many people participating? Are they active? Is anyone excluded from participation?*
Score / Average score assigned	
Identified successes	
Identified concerns / areas for improvement	
Actions agreed	
Additional notes	

2. LEADERSHIP

Criteria for assessment	*Do groups have leaders, are they helping or hindering the group? Do they have the capacity to lead groups well?*
Score / Average score assigned	
Identified successes	
Identified concerns / areas for improvement	
Actions agreed	
Additional notes	

For peer review only - http://bmjopen.bmj.com/site/about/guidelines.xhtml

WOMEN AND CHILDREN FIRST (UK)

3. ORGANISATIONAL STRUCTURE

**Criteria for assessment**	***Are the organisational structures, including the committee and taskforces, set up in the groups? If so, what is their purpose and how are they functioning?***
**Score / Average score assigned**	
**Identified successes**	
**Identified concerns / areas for improvement**	
**Actions agreed**	
**Additional notes**	

4. NEEDS / PROBLEM ASSESSMENT

**Criteria for assessment**	***Are the problems identified in the groups important and relevant to their respective community?***
**Score / Average score assigned**	
**Identified successes**	
**Identified concerns / areas for improvement**	
**Actions agreed**	
**Additional notes**	

5. RESOURCE MOBILISATION

Criteria for assessment	***Are the groups able to mobilise resources to implement their solutions? Are they able to mobilise resources from within the groups and/or outside the groups?***
Score / Average score assigned	
Identified successes	
Identified concerns / areas for improvement	
Actions agreed	
Additional notes	

6. ABILITY TO ASK "WHY"?

Criteria for assessment	***Do groups try to address root causes of the problems they face? If so, how well? By root causes we are referring to meeting re: contributing factors – the broader causes of ill health such as poverty.***
Score / Average score assigned	
Identified successes	
Identified concerns / areas for improvement	
Actions agreed	
Additional notes	

7. LINKS TO OTHER ORGANISATIONS / PEOPLE

**Criteria for assessment**	***Are they linking to other stakeholders (people, groups, organisations) who can help them solve identified issues? If so, who are they linking with and why?***
**Score / Average score assigned**	
**Identified successes**	
**Identified concerns / areas for improvement**	
**Actions agreed**	
**Additional notes**	

8. RELATIONSHIP WITH OUTSIDE AGENT

**Criteria for assessment**	***Is the external agent – Ministry of Health counterparts – involved and supporting the groups, building their capacity?***
**Score / Average score assigned**	
**Identified successes**	
**Identified concerns / areas for improvement**	
**Actions agreed**	
**Additional notes**	

9. PROGRAMME MANAGEMENT

Criteria for assessment	*Do groups feel they own the groups and their work? Are they capable of continuing the activities once the project comes to an end? Is the NGO implementing partner building group capacity to solve health problems and strengthening them to be sustainable?*
Score / Average score assigned	
Identified successes	
Identified concerns / areas for improvement	
Actions agreed	
Additional notes	

For peer review only

Direct observation of intervention delivery and CF performance Form:

Did the facilitator deliver the components of the sessions according to the manual?
(Please rate this on scale of 1-4 scale i.e. 1 = no elements of the programme delivered;
2 = some elements of the programme, others missed or inserted;
3 = majority elements of the programme delivered;
4 = all elements of the programme delivered as per handbook).

What are your thoughts about the overall session?

Did you think there are areas for improvement and what where they?

Would you have done anything differently while facilitating the sessions and how?

What advice do you have for the community facilitator?

For peer review only

For peer review only - http://bmjopen.bmj.com/site/about/guidelines.xhtml

Development performance of children – (ASQ-3)

Improving lives

NHS
Oxleas
NHS Foundation Trust

Parents of Shiv LOGAN
3 Abbey Terrace
Abbey Wood
London
SE2 9EY

380 D

Oxleas 0 – 19 Years Public Health Nursing Service
Central Administration Team
Tel: 020 8836 8621
Email: oxl-tr.cat@nhs.net

11 Nov 2019

Dear Parents of Shiv,

9 – 12 month review Appointment confirmation

This letter is to confirm your appointment to review your child's development.

At: Gallions Reach Health Centre Bentham Road Thamesmead London SE28 8BE

On: Tuesday 10 Dec 2019 10:30

Enclosed with this letter are the 'Ages and Stages' questionnaires that are used for this developmental review. Please complete these forms and try out the activities with your baby before the appointment.

It is important that you complete the questionnaires with your child before your appointment and bring the completed questionnaires and Red Book with you on the day.

If you require any help completing this questionnaire or you have any queries about your appointment please contact us on **020 8836 8621**

To find out about Healthy Start benefits search online for the Healthy Start website

Visit our website which was designed by Oxleas Health Care professionals, service users and families to provide you with information about your health and wellbeing and how to access our services.

www.Oxleas.nhs.uk/CYP

Follow us on Twitter @oxleasCYPGre

There is a drop in Health Advice Session open every day from Monday to Friday across Greenwich. For times and venues see Health Visitor Timetable in the link above.

Yours sincerely
Public Health Nursing Service 0 -19 Years

For peer review only - http://bmjopen.bmj.com/site/about/guidelines.xhtml

ASQ-3 **10 Month Questionnaire** *page 3 of 6*

GROSS MOTOR *(continued)*

YES SOMETIMES NOT YET

3. When you stand your baby next to furniture or the cot rail, does she hold on without leaning her chest against the furniture for support?

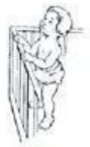

4. While holding onto furniture, does your baby bend down and pick up a toy from the floor and then return to a standing position?

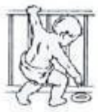

5. While holding onto furniture, does your baby lower himself with control (without falling or flopping down)?

6. Does your baby walk beside furniture while holding on with only one hand?

GROSS MOTOR TOTAL ___

FINE MOTOR

YES SOMETIMES NOT YET

1. Does your baby pick up a small toy with only one hand?

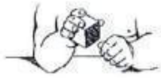

2. Does your baby *successfully* pick up a small cube of bread by using her thumb and all of her fingers in a raking motion? (*If she already picks up a small cube of bread, mark "yes" for this item.*)

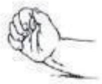

3. Does your baby pick up a small toy with the *tips* of his thumb and fingers? (*You should see a space between the toy and his palm.*)

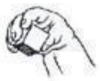

4. After one or two tries, does your baby pick up a piece of string with her first finger and thumb? (*The string may be attached to a toy.*)

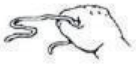

5. Does your baby pick up a small cube of bread with the tips of his thumb and a finger? (*He may rest his arm or hand on the table while doing it.*) ___ *

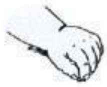

6. Does your baby put a small toy down, without dropping it, and then take her hand off the toy?

FINE MOTOR TOTAL ___

* If Fine Motor Item 5 is marked "yes" or "sometimes," mark Fine Motor Item 2 "yes."

BE Av. 1 1.15

Ages & Stages Questionnaires®, Third Edition (ASQ-3™), Squires & Bricker
© 2009-2015 Paul H. Brookes Publishing Co. All rights reserved.
Adaptation into British English prepared with the Department of Health.

OVERALL

Parents and providers may use the space below for additional comments.

1. Does your baby use both hands and both legs equally well? If no, explain: ○ YES ○ NO

2. When you help your baby stand, are his feet flat on the surface most of the time? If no, explain: ○ YES ○ NO

3. Do you have concerns that your baby is too quiet or does not make sounds like other babies? If yes, explain: ○ YES ○ NO

4. Does either parent have a family history of childhood deafness or hearing problems? If yes, explain: ○ YES ○ NO

5. Do you have concerns about your baby's eyesight? If yes, explain: ○ YES ○ NO

6. Has your baby had any medical or health-related problems in the last few months? If yes, explain: ○ YES ○ NO

29891/W1P00067353/0000069/0404/

BE Av. 1 1.15

Ages & Stages Questionnaires®, Third Edition (ASQ-3™), Squires & Bricker
© 2009-2015 Paul H. Brookes Publishing Co. All rights reserved.
Adaptation into British English prepared with the Department of Health.

4-day Food Diary

DAY 1

Day 1:		Date:		
**Time**	**Where? With Whom? TV on? At table?**	**Food/Drink description & preparation**	**Brand Name**	**Portion size or quantity eaten**
How to describe what you had and how much you had can be found on pages 16 - 21				
6am to 9am				
9am to 12 noon				

Time	Where? With Whom? TV on? At table?	Food/Drink description & preparation	Brand Name	Portion size or quantity eaten
12 noon to 2pm				
2pm to 5pm				

For peer review only - http://bmjopen.bmj.com/site/about/guidelines.xhtml

Time	Where?<br>With Whom?<br>TV on?<br>At table?	Food/Drink description & preparation	Brand Name	Portion size or quantity eaten
		5pm to 8pm		
		8pm to 10pm		
		10pm to 6am		

Was the amount of **food** that you had today about what you usually have, less than usual, or more than usual?

Yes, usual ☐ No, **less** than usual ☐ No, **more** than usual ☐

Please tell us why you had less than usual

Please tell us why you had more than usual

Was the amount you had to **drink** today, including water, tea, coffee and soft drinks [and alcohol], about what you usually have, less than usual, or more than usual?

Yes, usual ☐ No, **less** than usual ☐ No, **more** than usual ☐

Please tell us why you had less than usual

Please tell us why you had more than usual

For peer review only - http://bmjopen.bmj.com/site/about/guidelines.xhtml

Did you **finish all the food and drink** that you recorded in the diary today?

Yes ☐ No ☐

If no, please **go back to the diary and make a note of any leftovers**

Did you take any **vitamins, minerals or other food supplements** today?

Yes ☐ No ☐

If yes, **please describe the supplements you took below**

Brand	Name (in full) including strength	Number of pills, capsules, teaspoons

Please record on the next pages details of any recipes or (if not already described) ingredients of made up dishes or take-away dishes.

Write in recipes or ingredients of made up dishes or take-away dishes			
NAME OF DISH:		***Serves:***	
Ingredients	***Amount***	***Ingredients***	***Amount***
Brief description of cooking method			

Write in recipes or ingredients of made up dishes or take-away dishes			
NAME OF DISH:		***Serves:***	
Ingredients	***Amount***	***Ingredients***	***Amount***
Brief description of cooking method			

DAY 2

Day 2:		Date:		
Time	Where? With Whom? TV on? At table?	Food/Drink description & preparation	Brand Name	Portion size or quantity eaten
How to describe what you had and how much you had can be found on pages 16 - 21				
6am to 9am				
9am to 12 noon				

For peer review only - http://bmjopen.bmj.com/site/about/guidelines.xhtml

Time	Where? With Whom? TV on? At table?	Food/Drink description & preparation	Brand Name	Portion size or quantity eaten
		12 noon to 2pm		
		2pm to 5pm		

Time	Where? With Whom? TV on? At table?	Food/Drink description & preparation	Brand Name	Portion size or quantity eaten
		5pm to 8pm		
		8pm to 10pm		
		10pm to 6am		

For peer review only - http://bmjopen.bmj.com/site/about/guidelines.xhtml

Was the amount of **food** that you had today about what you usually have, less than usual, or more than usual?

Yes, usual ☐ No, **less** than usual ☐ No, **more** than usual ☐

Please tell us why you had less than usual

Please tell us why you had more than usual

Was the amount you had to **drink** today, including water, tea, coffee and soft drinks [and alcohol], about what you usually have, less than usual, or more than usual?

Yes, usual ☐ No, **less** than usual ☐ No, **more** than usual ☐

Please tell us why you had less than usual

Please tell us why you had more than usual

Did you **finish all the food and drink** that you recorded in the diary today?

Yes ☐ No ☐

If no, please **go back to the diary and make a note of any leftovers**

Did you take any **vitamins, minerals or other food supplements** today?

Yes ☐ No ☐

If yes, **please describe the supplements you took below**

Brand	Name (in full) including strength	Number of pills, capsules, teaspoons

Please record on the next pages details of any recipes or (if not already described) ingredients of made up dishes or take-away dishes.

For peer review only - http://bmjopen.bmj.com/site/about/guidelines.xhtml

Write in recipes or ingredients of made up dishes or take-away dishes			
NAME OF DISH:		***Serves:***	
Ingredients	***Amount***	***Ingredients***	***Amount***
Brief description of cooking method			

Write in recipes or ingredients of made up dishes or take-away dishes			
NAME OF DISH:		***Serves:***	
Ingredients	***Amount***	***Ingredients***	***Amount***
Brief description of cooking method			

DAY 3

Day 3:		Date:		
Time	Where?<br>With Whom?<br>TV on?<br>At table?	Food/Drink description & preparation	Brand Name	Portion size or quantity eaten
How to describe what you had and how much you had can be found on pages 16 - 21				
6am to 9am				
9am to 12 noon				

Time	Where?<br>With Whom?<br>TV on?<br>At table?	Food/Drink description & preparation	Brand Name	Portion size or quantity eaten
12 noon to 2pm				
2pm to 5pm				

For peer review only - http://bmjopen.bmj.com/site/about/guidelines.xhtml

Time	Where? With Whom? TV on? At table?	Food/Drink description & preparation	Brand Name	Portion size or quantity eaten
		5pm to 8pm		
		8pm to 10pm		
		10pm to 6am		

Was the amount of **food** that you had today about what you usually have, less than usual, or more than usual?

Yes, usual ☐ No, **less** than usual ☐ No, **more** than usual ☐

Please tell us why you had less than usual

Please tell us why you had more than usual

Was the amount you had to **drink** today, including water, tea, coffee and soft drinks [and alcohol], about what you usually have, less than usual, or more than usual?

Yes, usual ☐ No, **less** than usual ☐ No, **more** than usual ☐

Please tell us why you had less than usual

Please tell us why you had more than usual

For peer review only - http://bmjopen.bmj.com/site/about/guidelines.xhtml

Did you **finish all the food and drink** that you recorded in the diary today?

Yes ☐ No ☐

If no, please **go back to the diary and make a note of any leftovers**

Did you take any **vitamins, minerals or other food supplements** today?

Yes ☐ No ☐

If yes, **please describe the supplements you took below**

Brand	Name (in full) including strength	Number of pills, capsules, teaspoons

Please record on the next pages details of any recipes or (if not already described) ingredients of made up dishes or take-away dishes.

**Write in recipes or ingredients of made up dishes or take-away dishes**			
NAME OF DISH:		***Serves:***	
Ingredients	***Amount***	***Ingredients***	***Amount***
Brief description of cooking method			

Write in recipes or ingredients of made up dishes or take-away dishes			
NAME OF DISH:		***Serves:***	
Ingredients	***Amount***	***Ingredients***	***Amount***
Brief description of cooking method			

DAY 4

Please remember to complete the general questions on pages 61-66!

Day 4:		Date:		
Time	Where? With Whom? TV on? At table?	Food/Drink description & preparation	Brand Name	Portion size or quantity eaten
How to describe what you had and how much you had can be found on pages 16 - 21				
6am to 9am				
9am to 12 noon				

For peer review only - http://bmjopen.bmj.com/site/about/guidelines.xhtml

Time	Where?<br>With Whom?<br>TV on?<br>At table?	Food/Drink description & preparation	Brand Name	Portion size or quantity eaten
		12 noon to 2pm		
		2pm to 5pm		

Time	Where?<br>With Whom?<br>TV on?<br>At table?	Food/Drink description & preparation	Brand Name	Portion size or quantity eaten
		5pm to 8pm		
		8pm to 10pm		
		10pm to 6am		

For peer review only - http://bmjopen.bmj.com/site/about/guidelines.xhtml

Was the amount of **food** that you had today about what you usually have, less than usual, or more than usual?

Yes, usual ☐ No, **less** than usual ☐ No, **more** than usual ☐

Please tell us why you had less than usual

Please tell us why you had more than usual

Was the amount you had to **drink** today, including water, tea, coffee and soft drinks [and alcohol], about what you usually have, less than usual, or more than usual?

Yes, usual ☐ No, **less** than usual ☐ No, **more** than usual ☐

Please tell us why you had less than usual

Please tell us why you had more than usual

Did you **finish all the food and drink** that you recorded in the diary today?

Yes ☐ No ☐

If no, please **go back to the diary and make a note of any leftovers**

Did you take any **vitamins, minerals or other food supplements** today?

Yes ☐ No ☐

If yes, **please describe the supplements you took below**

Brand	Name (in full) including strength	Number of pills, capsules, teaspoons

Please record on the next pages details of any recipes or (if not already described) ingredients of made up dishes or take-away dishes.

For peer review only - http://bmjopen.bmj.com/site/about/guidelines.xhtml

Write in recipes or ingredients of made up dishes or take-away dishes			
NAME OF DISH:		***Serves:***	
Ingredients	***Amount***	***Ingredients***	***Amount***
Brief description of cooking method			

Write in recipes or ingredients of made up dishes or take-away dishes			
NAME OF DISH:		***Serves:***	
Ingredients	***Amount***	***Ingredients***	***Amount***
Brief description of cooking method			

General questions about your food/ drink during the recording period.

Special diet

1. Did you follow a special diet during the recording period e.g. vegetarian, cholesterol lowering, weight reducing?

Yes ☐ *Please specify* No ☐

Milk

2. Which type of milk did you use most often during the recording period?

Whole, fresh, pasteurised ☐ Semi-skimmed fresh, pasteurised ☐ Skimmed (fat free) fresh, pasteurised ☐ 1% fat milk, pasteurised ☐

Dried ☐ *Type* Soya ☐ *Type*

Other ☐ *Type* Did not use ☐

Tea and coffee

3. How much milk did you usually have in coffee/ tea?

Coffee A lot ☐ Some ☐ A little ☐ None/did not drink ☐

Tea A lot ☐ Some ☐ A little ☐ None/did not drink ☐

4. Did you usually sweeten your coffee/ tea with sugar?

Coffee Yes ☐ How many teaspoons in a mug/cup? ☐ No/did not drink ☐

Tea Yes ☐ How many teaspoons in a mug/cup? ☐ No/did not drink ☐

5. Did you usually sweeten your coffee/ tea with artificial sweetener?

Coffee Yes ☐ How many tablets or teaspoons in a mug/cup? ☐ No/did not drink ☐

Tea Yes ☐ How many tablets or teaspoons in a mug/cup? ☐ No/did not drink ☐

6. Did you drink decaffeinated coffee/ tea during the recording period?

Coffee Always ☐ Sometimes ☐ Never ☐

Tea Always ☐ Sometimes ☐ Never ☐

For peer review only - http://bmjopen.bmj.com/site/about/guidelines.xhtml

Breakfast cereals

7. How much milk did you usually have on breakfast cereal?

Drowned ☐ Average ☐ Damp ☐ None/did not eat ☐

8. How did you usually make your porridge?

With all water ☐ With all milk ☐ With milk and water ☐ Did not eat ☐

9. Did you usually sweeten or salt your porridge?

With sugar ☐ With honey ☐ With salt ☐ Neither/did not eat ☐

10. How did you usually make your instant oat cereal?

With all water ☐ With all milk ☐ With milk and water ☐ Did not eat ☐

11. Did you usually sweeten or salt your instant oat cereal?

With sugar ☐ With honey ☐ With salt ☐ Neither/did not eat ☐

Fats for spreading and cooking

12. Which type of butter, margarine or other fat spread did you use most often during the recording period? Please record the full product name and fat content

Name:

None ☐

e.g. Flora Omega 3 plus, low fat spread, 38% fat, polyunsaturated

13. How thickly did you spread butter, margarine on bread, crackers etc?

Thick ☐ Medium ☐ Thin ☐ N/A ☐

14. Which type of cooking fat/oil did your household use most often over the recording period? Please record the full product name e.g. *Sainsbury's sunflower oil*

Name:

None ☐

Bread

15. Which type of bread did you eat most often during the recording period?

White ☐ Granary ☐ Wholemeal ☐ Brown ☐

50/50 bread e.g. Hovis Best of Both ☐ Other ☐ *Type* Did not eat ☐

16. Was it a large loaf or a small loaf?

Large ☐ Small ☐

17. If the bread was shop bought, how was it sliced?

Thick ☐ Medium ☐ Thin ☐ Unsliced ☐ N/A ☐

Meat

18. If you ate meat during the recording period, did you eat the visible fat?

Always ☐ Sometimes ☐ Never ☐ Did not eat meat ☐

19. If you ate poultry (e.g. chicken, turkey) during the recording period, did you eat the skin?

Always ☐ Sometimes ☐ Never ☐ Did not eat poultry ☐

Fruit and vegetables

20. If you ate apples during the recording period, did you eat the skin?

Always ☐ Sometimes ☐ Never ☐ Did not eat ☐

21. If you ate pears during the recording period, did you eat the skin?

Always ☐ Sometimes ☐ Never ☐ Did not eat ☐

22. If you ate new potatoes during the recording period, did you eat the skin?

Always ☐ Sometimes ☐ Never ☐ Did not eat ☐

23. If you ate baked/jacket potatoes during the recording period, did you eat the skin?

Always ☐ Sometimes ☐ Never ☐ Did not eat ☐

Salt

24. Do you add salt to your food at the table?

Always ☐ Sometimes ☐ Never ☐

25. Do you add salt substitute to your food at the table? *e.g. LoSalt*

Always ☐ Sometimes ☐ Never ☐

Water

26. Which type of water did you drink most often during the recording period?

Tap ☐ Filtered ☐ Bottled ☐ *brand* ______ Did not drink ☐

Thank you for completing this diary.

For peer review only - http://bmjopen.bmj.com/site/about/guidelines.xhtml

Children Feeding Behaviour

ID:

Child Eating Behaviour Questionnaire (CEBQ)

Please read the following statements and tick the boxes most appropriate to your child's eating behaviour.

	Never	Rarely	Some-times	Often	Always
My child loves food	☐	☐	☐	☐	☐
My child eats more when worried	☐	☐	☐	☐	☐
My child has a big appetite	☐	☐	☐	☐	☐
My child finishes his/her meal quickly	☐	☐	☐	☐	☐
My child is interested in food	☐	☐	☐	☐	☐
My child is always asking for a drink	☐	☐	☐	☐	☐
My child refuses new foods at first	☐	☐	☐	☐	☐
My child eats slowly	☐	☐	☐	☐	☐
My child eats less when angry	☐	☐	☐	☐	☐
My child enjoys tasting new foods	☐	☐	☐	☐	☐
My child eats less when s/he is tired	☐	☐	☐	☐	☐
My child is always asking for food	☐	☐	☐	☐	☐
My child eats more when annoyed	☐	☐	☐	☐	☐
If allowed to, my child would eat too much	☐	☐	☐	☐	☐
My child eats more when anxious	☐	☐	☐	☐	☐
My child enjoys a wide variety of foods	☐	☐	☐	☐	☐
My child leaves food on his/her plate at the end of a meal	☐	☐	☐	☐	☐
My child takes more than 30 minutes to finish a meal	☐	☐	☐	☐	☐

For peer review only - http://bmjopen.bmj.com/site/about/guidelines.xhtml

	Never	Rarely	Some-times	Often	Always
Given the choice, my child would eat most of the time	☐	☐	☐	☐	☐
My child looks forward to mealtimes	☐	☐	☐	☐	☐
My child gets full before his/her meal is finished	☐	☐	☐	☐	☐
My child enjoys eating	☐	☐	☐	☐	☐
My child eats more when she is happy	☐	☐	☐	☐	☐
My child is difficult to please with meals	☐	☐	☐	☐	☐
My child eats less when upset	☐	☐	☐	☐	☐
My child gets full up easily	☐	☐	☐	☐	☐
My child eats more when s/he has nothing else to do	☐	☐	☐	☐	☐
Even if my child is full up s/he finds room to eat his/her favourite food	☐	☐	☐	☐	☐
If given the chance, my child would drink continuously throughout the day	☐	☐	☐	☐	☐
My child cannot eat a meal if s/he has had a snack just before	☐	☐	☐	☐	☐
If given the chance, my child would always be having a drink	☐	☐	☐	☐	☐
My child is interested in tasting food s/he hasn't tasted before	☐	☐	☐	☐	☐
My child decides that s/he doesn't like a food, even without tasting it	☐	☐	☐	☐	☐
If given the chance, my child would always have food in his/her mouth	☐	☐	☐	☐	☐
My child eats more and more slowly during the course of a meal	☐	☐	☐	☐	☐

For peer review only - http://bmjopen.bmj.com/site/about/guidelines.xhtml

Parental Feeding Style

Parental Feeding Style Questionnaire

Please read the following statements and tick the appropriate boxes to show how you deal with feeding your child. It is important to remember that there are no right or wrong answers to these questions, we are interested in what parents really feel and do.

ID

	Never	Rarely	Some-times	Often	Always
I allow my child to choose which foods to have for meals	☐	☐	☐	☐	☐
I give my child something to eat to make him/her feel better when s/he is feeling upset	☐	☐	☐	☐	☐
I encourage my child to look forward to the meal	☐	☐	☐	☐	☐
I praise my child if s/he eats what I give him/her	☐	☐	☐	☐	☐
I decide how many snacks my child should have	☐	☐	☐	☐	☐
I encourage my child to eat a wide variety of foods	☐	☐	☐	☐	☐
In order to get my child to behave him/herself I promise him/her something to eat	☐	☐	☐	☐	☐
I present food in an attractive way to my child	☐	☐	☐	☐	☐
If my child misbehaves I withhold his/her favourite food	☐	☐	☐	☐	☐
I encourage my child to taste each of the foods I serve at mealtimes	☐	☐	☐	☐	☐
I allow my child to wander around during a meal	☐	☐	☐	☐	☐
I encourage my child to try foods that s/he hasn't tasted before	☐	☐	☐	☐	☐
I give my child something to eat to make him/her feel better when s/he has been hurt	☐	☐	☐	☐	☐
I let my child decide when s/he would like to have her meal	☐	☐	☐	☐	☐
I give my child something to eat if s/he is feeling bored	☐	☐	☐	☐	☐
I allow my child to decide when s/he has had enough snacks to eat	☐	☐	☐	☐	☐
I decide when it is time for my child to have a snack	☐	☐	☐	☐	☐
I use puddings as a bribe to get my child to eat his/her main course	☐	☐	☐	☐	☐
I encourage my child to enjoy his/her food	☐	☐	☐	☐	☐

PTO

For peer review only - http://bmjopen.bmj.com/site/about/guidelines.xhtml

Network Diffusion Form

(To be completed by participants at the end of each PLA session and at 6 months from baseline)

1. How many people did you share your material/information from the PLA sessions with?..

2. How did you share this information/material (verbal, social media, whatsapp etc)?..

3. Please fill the table below with details of the people you shared the information with -

Serial number	Relation to you	Age/Gender	Family size	How did you share (verbal, social media, whatsapp)

For peer review only - http://bmjopen.bmj.com/site/about/guidelines.xhtml

Equality Impact Assessment

Equality Impact Assessment- NEON

Question	Response
**1. Name of intervention being assessed**	NEON Intervention (PLA Cycle)
**2. Summary of aims and objectives of the intervention**	To improve infant feeding, care and dental hygiene practices amongst the South Asian Communities of Tower Hamlets, Newham, and Waltham Forest
**3. What involvement and consultation has been done in relation to this intervention?** *(e.g., with relevant groups and stakeholders)*	
**4. Who is affected by the intervention?**	Infants (aged<24 months)<br>Mothers<br>Pregnant women<br>Carers- Grandmother etc.
**5. What are the arrangements for monitoring and reviewing the actual impact of the intervention?**	

Protected Characteristic Group	Is there a potential for positive or negative impact?	Please explain and give examples of any evidence/data used	Action to address negative impact (e.g. adjustment to the intervention)
**Disability**			
**Gender reassignment**			
**Marriage or civil partnership**			
**Pregnancy and maternity**			
**Race**			
**Religion or belief**			
**Sexual orientation**			
**Sex (gender)**			
**Age**			

For peer review only - http://bmjopen.bmj.com/site/about/guidelines.xhtml

Evaluation:

Question	Explanation / justification	
Is it possible the proposed intervention could discriminate or unfairly disadvantage people?		
**Final Decision:**	**Tick the relevant box**	**Include any explanation / justification required**
1. No barriers identified, therefore activity will **proceed**.		
2. You can decide to **stop** the intervention at some point because the data shows bias towards one or more groups		
3. You can **adapt or change** the intervention in a way which you think will eliminate the bias		
4. Barriers and impact identified, however having considered all available options carefully, there appear to be no other proportionate ways to achieve the aim of the intervention (e.g., in extreme cases or where positive action is taken). Therefore, you are going to **proceed with caution** with this intervention knowing that it may favour some people less than others, providing justification for this decision.		

**Date completed:**	
**Completed by whom (Borough/individual):**	

Staff time questionnaire

Measurement of Time Use for staff involved in *NEON*

This questionnaire is for collecting information on how staff from the study team who are contributing to the Nurture Early for Optimal Nutrition (*NEON*) divide their time between the different trial components. We aim to collect this information from all our partners staff involved in *NEON* over the duration of the 3-year of the study.

The information you provide will only be used for research purposes only. It ***will not*** be used to monitor performance or to plan future activities. The information you provide will help us to divide the total *NEON* cost into the following components: intervention, M&E, process evaluation, and research. This will allow us to report the cost effectiveness of the *NEON* intervention. We also want to separate out the setting up cost from the implementation cost, to inform others who may wish to implement these interventions.

Your name will not be used in the analysis but you will be identifiable through your answers. If you do not want to proceed, or wish to stop at any time, you can do so. If you have any questions, please discuss with Shereen Al Laham (*s.laham@ucl.ac.uk*).

==

Name of Staff Member: ....

Job title / role: ....

Institution: ....

Date: ....

---

1. When did your involvement with *NEON* first begin? .... (Month/Year)
2. Since you started, have you been involved in any project other than *NEON*?

   ☐ Yes

   ☐ No. **Go to question 6.**

3. If Yes, how many days did you spend on *NEON* in the last 30 days?

   .... Days OR .... Percent of time

4. Was this a typical month for you, since you started work on *NEON*?

   ☐ Yes. **Go to question 6.**

   ☐ No

5. If No, please tell me how you have divided your time between *NEON* and any other project since you first started on *NEON*.

   ....

....

(E.g: On average, 2 days per week from January to May, then full-time since June. Answer can be in percentage, days, or weeks – whatever you think most accurately represents your actual time use.)

6. Thinking now about the **last 30 days**, and only those days you worked on *NEON*, how have you in practice divided your time between the following *NEON* activities?

Page **1** of **2**

For peer review only - http://bmjopen.bmj.com/site/about/guidelines.xhtml

a) I spent .... days OR .... percent time on Monitoring and Evaluation.
b) I spent .... days OR .... percent time on study delivery and implementation.
c) I spent .... days OR .... percent time on Research Work, that is, working on the study design, reading the literature, or writing up or presenting the research findings.
d) I spent .... days OR .... percent time on Joint Work, that is, it is not possible to say it was one of the above. For example, general management or support functions.

7. Is this how you usually divide your time?

☐ Yes. **Go to question 9.**

☐ No

8. If No, please tell me how you have divided your time between the different *NEON* activities since you first started on *NEON*. *(Use the categories in question 6.)*

....

....

....

(E.g: 50% Joint work for the first 6 months, then 1 day per week Joint work but otherwise only NEON. Answer can be in percentage, days, or weeks – whatever you think most accurately represents your actual time use.)

9. Finally, I want to ask you about the division of time between **setting up** and **implementation** of the *NEON* study. Examples of setting up activities are: recruiting and training; developing and testing the intervention material. These activities may still be ongoing.
Please could you tell us when you start to work on implementation, and do setting up activities still continue

	**Activity**	**Start date of implementation** *(Month/Year)*	**Time spend on SETTING UP activities since start date** *(days per week or percentage of time)*
a	NEON intervention implementation		
b	Monitoring and Evaluation implementation		
c	Process Evaluation implementation		

== **Thank You** ==

For peer review only - http://bmjopen.bmj.com/site/about/guidelines.xhtml

SPIRIT

STANDARD PROTOCOL ITEMS: RECOMMENDATIONS FOR INTERVENTIONAL TRIALS

SPIRIT 2013 Checklist: Recommended items to address in a clinical trial protocol and related documents*

Section/ item	Item No	Description	Page
**Administrative information**			
Title	1	Descriptive title identifying the study design, population, interventions, and, if applicable, trial acronym	1
Trial registration	2a	Trial identifier and registry name. If not yet registered, name of intended registry	3, 17, 18
	2b	All items from the World Health Organization Trial Registration Data Set	N/A
Protocol version	3	Date and version identifier	17, 18
Funding	4	Sources and types of financial, material, and other support	17
Roles and responsibilities	5a	Names, affiliations, and roles of protocol contributors	1, 17
	5b	Name and contact information for the trial sponsor	17
	5c	Role of study sponsor and funders, if any, in study design; collection, management, analysis, and interpretation of data; writing of the report; and the decision to submit the report for publication, including whether they will have ultimate authority over any of these activities	17, 18
	5d	Composition, roles, and responsibilities of the coordinating centre, steering committee, endpoint adjudication committee, data management team, and other individuals or groups overseeing the trial, if applicable (see Item 21a for data monitoring committee)	11, 16
**Introduction**			
Background and rationale	6a	Description of research question and justification for undertaking the trial, including summary of relevant studies (published and unpublished) examining benefits and harms for each intervention	3, 5
	6b	Explanation for choice of comparators	6, 7
Objectives	7	Specific objectives or hypotheses	6
Trial design	8	Description of trial design including type of trial (eg, parallel group, crossover, factorial, single group), allocation ratio, and framework (eg, superiority, equivalence, noninferiority, exploratory)	6, 7

For peer review only

Methods: Participants, interventions, and outcomes

Study setting	9	Description of study settings (eg, community clinic, academic hospital) and list of countries where data will be collected. Reference to where list of study sites can be obtained	7
Eligibility criteria	10	Inclusion and exclusion criteria for participants. If applicable, eligibility criteria for study centres and individuals who will perform the interventions (eg, surgeons, psychotherapists)	7, 8
Interventions	11a	Interventions for each group with sufficient detail to allow replication, including how and when they will be administered	6, 7, 10
	11b	Criteria for discontinuing or modifying allocated interventions for a given trial participant (eg, drug dose change in response to harms, participant request, or improving/worsening disease)	N/A
	11c	Strategies to improve adherence to intervention protocols, and any procedures for monitoring adherence (eg, drug tablet return, laboratory tests)	N/A
	11d	Relevant concomitant care and interventions that are permitted or prohibited during the trial	N/A
Outcomes	12	Primary, secondary, and other outcomes, including the specific measurement variable (eg, systolic blood pressure), analysis metric (eg, change from baseline, final value, time to event), method of aggregation (eg, median, proportion), and time point for each outcome. Explanation of the clinical relevance of chosen efficacy and harm outcomes is strongly recommended	11, 12
Participant timeline	13	Time schedule of enrolment, interventions (including any run-ins and washouts), assessments, and visits for participants. A schematic diagram is highly recommended (see Figure)	9
Sample size	14	Estimated number of participants needed to achieve study objectives and how it was determined, including clinical and statistical assumptions supporting any sample size calculations	8
Recruitment	15	Strategies for achieving adequate participant enrolment to reach target sample size	8, 9

Methods: Assignment of interventions (for controlled trials)

Allocation:

Sequence generation	16a	Method of generating the allocation sequence (eg, computer-generated random numbers), and list of any factors for stratification. To reduce	9, 10

For peer review only - http://bmjopen.bmj.com/site/about/guidelines.xhtml

		predictability of a random sequence, details of any planned restriction (eg, blocking) should be provided in a separate document that is unavailable to those who enrol participants or assign interventions	
Allocation concealment mechanism	16b	Mechanism of implementing the allocation sequence (eg, central telephone; sequentially numbered, opaque, sealed envelopes), describing any steps to conceal the sequence until interventions are assigned	9, 10
Implementation	16c	Who will generate the allocation sequence, who will enrol participants, and who will assign participants to interventions	9, 10
Blinding (masking)	17a	Who will be blinded after assignment to interventions (eg, trial participants, care providers, outcome assessors, data analysts), and how	9, 10
	17b	If blinded, circumstances under which unblinding is permissible, and procedure for revealing a participant's allocated intervention during the trial	9, 10
**Methods: Data collection, management, and analysis**			
Data collection methods	18a	Plans for assessment and collection of outcome, baseline, and other trial data, including any related processes to promote data quality (eg, duplicate measurements, training of assessors) and a description of study instruments (eg, questionnaires, laboratory tests) along with their reliability and validity, if known. Reference to where data collection forms can be found, if not in the protocol	11, 12, 13
	18b	Plans to promote participant retention and complete follow-up, including list of any outcome data to be collected for participants who discontinue or deviate from intervention protocols	14, 16
Data management	19	Plans for data entry, coding, security, and storage, including any related processes to promote data quality (eg, double data entry; range checks for data values). Reference to where details of data management procedures can be found, if not in the protocol	14
Statistical methods	20a	Statistical methods for analysing primary and secondary outcomes. Reference to where other details of the statistical analysis plan can be found, if not in the protocol	14, 15
	20b	Methods for any additional analyses (eg, subgroup and adjusted analyses)	N/A
	20c	Definition of analysis population relating to protocol non-adherence (eg, as randomised analysis), and any statistical methods to handle missing data (eg, multiple imputation)	N/A
**Methods: Monitoring**			
Data monitoring	21a	Composition of data monitoring committee (DMC); summary of its role and reporting structure; statement of whether it is independent from the sponsor and competing interests; and reference to where further details	16

		about its charter can be found, if not in the protocol. Alternatively, an explanation of why a DMC is not needed	
	21b	Description of any interim analyses and stopping guidelines, including who will have access to these interim results and make the final decision to terminate the trial	16
/Harms	22	Plans for collecting, assessing, reporting, and managing solicited and spontaneously reported adverse events and other unintended effects of trial interventions or trial conduct	N/A
Auditing	23	Frequency and procedures for auditing trial conduct, if any, and whether the process will be independent from investigators and the sponsor	N/A
**Ethics and dissemination**			
Research ethics approval	24	Plans for seeking research ethics committee/institutional review board (REC/IRB) approval	2, 16, 18
Protocol amendments	25	Plans for communicating important protocol modifications (eg, changes to eligibility criteria, outcomes, analyses) to relevant parties (eg, investigators, REC/IRBs, trial participants, trial registries, journals, regulators)	16, 17
Consent or assent	26a	Who will obtain informed consent or assent from potential trial participants or authorised surrogates, and how (see Item 32)	18
	26b	Additional consent provisions for collection and use of participant data and biological specimens in ancillary studies, if applicable	N/A
Confidentiality	27	How personal information about potential and enrolled participants will be collected, shared, and maintained in order to protect confidentiality before, during, and after the trial	17, 18
Declaration of interests	28	Financial and other competing interests for principal investigators for the overall trial and each study site	17, 18
Access to data	29	Statement of who will have access to the final trial dataset, and disclosure of contractual agreements that limit such access for investigators	18
Ancillary and post-trial care	30	Provisions, if any, for ancillary and post-trial care, and for compensation to those who suffer harm from trial participation	N/A
Dissemination policy	31a	Plans for investigators and sponsor to communicate trial results to participants, healthcare professionals, the public, and other relevant groups (eg, via publication, reporting in results databases, or other data sharing arrangements), including any publication restrictions	18
	31b	Authorship eligibility guidelines and any intended use of professional writers	N/A

For peer review only - http://bmjopen.bmj.com/site/about/guidelines.xhtml

	31c	Plans, if any, for granting public access to the full protocol, participant-level dataset, and statistical code	18
**Appendices**			
Informed consent materials	32	Model consent form and other related documentation given to participants and authorised surrogates	Suppl. material
Biological specimens	33	Plans for collection, laboratory evaluation, and storage of biological specimens for genetic or molecular analysis in the current trial and for future use in ancillary studies, if applicable	N/A

*It is strongly recommended that this checklist be read in conjunction with the SPIRIT 2013 Explanation & Elaboration for important clarification on the items. Amendments to the protocol should be tracked and dated.

The SPIRIT checklist is copyrighted by the SPIRIT Group under the Creative Commons "Attribution-NonCommercial-NoDerivs 3.0 Unported" license.

For peer review only

BMJ Open

Nurture Early for Optimal Nutrition (NEON) participatory learning and action women's groups to improve infant feeding and practices in South Asian infants: pilot randomised trial study protocol

Journal:	*BMJ Open*
Manuscript ID	bmjopen-2022-063885.R3
Article Type:	Protocol
Date Submitted by the Author:	19-Oct-2023

Complete List of Authors:	Manikam, Logan; UCL Institute of Epidemiology and Health Care, Epidemiology and Public Health; Aceso Global Health Consultants<br>Allaham, Shereen ; UCL Institute of Epidemiology and Health Care, Department of Epidemiology and Public Health<br>Patil, Priyanka; University College London Institute of Epidemiology and Health Care; Aceso Global Health Consultants Pte Limited<br>Naman, Maryan; Aceso Global Health Consultants Pte Limited<br>Ong, Zhen Ling; Aceso Global Health Consultants Limited<br>Demel, Isabel-Cathérine; King's College London Faculty of Life Sciences and Medicine, Guy's, King's and St.Thomas' School of Medicine<br>Batura, Neha; University College London, Institute for Global Health<br>Llewellyn, Clare; University College London Department of Behavioural Science and Health<br>Hayward, Andrew; University College London, Epidemiology and Public Health<br>Lakshman, Rajalakshmi; Cambridgeshire County Council, Public Health Directorate; University of Cambridge, Medical Research Council Epidemiology Unit<br>Gilmour, Jenny; Mile End Hospital, Tower Hamlets GP Care Group<br>Webb-Martin, Kelley; London Borough of Newham, Children's Health 0-19 Service<br>Irish, Carol; London Borough of Newham, Children's Health 0-19 Service<br>Archibong, Mfon; London Borough of Newham, Children's Health 0-19 Service<br>Clarkson, Corinne; London Borough of Waltham Forest, Council, Public Health Strategist for Maternal & Child Health<br>Delceta, Daley; Chingford Health Centre Waltham Forest, Operational Lead<br>Islam, Lily; London Borough of Tower Hamlets, Community Facilitator<br>Bajwa, Seema; London Borough of Newham, Community Facilitator<br>Malek, Sabiha; London Borough of Newham, Community Facilitator<br>Bhachu, Jasvir; London Borough of Newham, Community Facilitator<br>Pushpakanthan, Geromini; London Borough of Newham, Community Facilitator<br>Heys, Michelle; University College London Institute of Child Health, University College London Institute of Child Health; Specialist Children and Young People's Services, East London NHS Foundation Trust<br>Lakhanpaul, Monica; UCL Great Ormond Street Institute of Child Health

	Population Policy and Practice, Institute of Child Health; Whittington Health NHS Trust
**Primary Subject Heading**:	Public health
Secondary Subject Heading:	Epidemiology, Evidence based practice, Global health, Nutrition and metabolism, Public health
Keywords:	Community child health < PAEDIATRICS, NUTRITION & DIETETICS, PAEDIATRICS, PUBLIC HEALTH

Nurture Early for Optimal Nutrition (NEON) participatory learning and action women's groups to improve infant feeding and practices in South Asian infants: pilot randomised trial study protocol

Short Title/ Acronym: Nurture Early for Optimal Nutrition (NEON) Pilot Feasibility Randomised Controlled Trial

Authors:
[1]Dr Logan Manikam, NIHR Advanced Fellow & Honorary Consultant in Public Health Medicine[1,2]
Shereen Allaham, Research Assistant & Consultant[1]
Priyanka Patil, Research Assistant & Consultant[1,2]
Maryan Naman, Consultant[2]
Zhen Ling Ong, Associate[2]
Isabel-Cathérine Demel, Medical Student[2,3]
Dr Neha Batura, Associate Professor in Health Economics[6]
Dr Clare Llewellyn, Associate Professor in Behavioural Obesity Research[7]
Prof Andrew Hayward, Professor of Infectious Disease Epidemiology and Inclusion Health Research[1]
Dr Rajalakshmi Lakshman, Consultant in Public Health Medicine[8]
Jenny Gilmour, 0-19 Service Development Lead THGPCG[9]
Kelley Webb Martin, Director of Delivery, Compliance & Transformation[10]
Carol Irish, Head of Children's Health 0-19 Service[10]
Mfon Archibong, Specialist Health Visiting Research Lead[10]
Corinne Clarkson, Public Health Strategist for Maternal & Child Health[11]
Daley Delceta, Operational Lead[12]
Lily Islam, Tower Hamlets community facilitator
Seema Bajwa, Newham community facilitator
Sabiha Malek, Newham community facilitator
Jasvir Bhachu, Newham community facilitator
Geromini Pushpakanthan, Newham community facilitator
[2]Dr Michelle Heys, Associate Professor of Community and Population Child Health, Consultant Paediatrician[4,5]
Monica Lakhanpaul Professor of Integrated Community Child Health[13,14] , On Behalf of the NEON Steering Team.

Membership of the NEON Steering Team is listed in the Acknowledgements

---

[1] Logan Manikam and Shereen Allaham are joint first authors

[2] Dr Michelle Heys and Professor Monica Lakhanpaul are joint senior authors

For peer review only - http://bmjopen.bmj.com/site/about/guidelines.xhtml
For peer review only

Affiliations:

1. Department of Epidemiology and Public Health, University College London Institute of Epidemiology and Health Care, London, UK
2. Aceso Global Health Consultants Pte Limited, Singapore
3. Department of Life Sciences & Medicine, King's College London GKT School of Medical Education, London, UK
4. Specialist Children and Young People's Services, East London NHS Foundation Trust, 84 West Ham Lane, London E15 4PT.
5. Population, Policy and Practice, UCL Great Ormond Street Institute of Child Health, 30 Guildford Street, London WC1N 1EH, UK
6. Institute for Global Health, University College London, 30 Guilford Street, London WC1N 1EH.
7. Department of Behavioural Sciences & Health, UCL IEHC, 1-19 Torrington Pl, Fitzrovia, London WC1E 7HB.
8. MRC Epidemiology Unit, University of Cambridge
9. Tower Hamlets GP Care Group, Mile End Hospital, London, UK
10. Children's Health 0-19 Service, London Borough of Newham, 1000 Dockside Road E16 2QU
11. Waltham Forest Council, London, UK
12. Chingford Health Centre Waltham Forest, London, UK
13. Population, Policy and Practice, UCL Great Ormond Street Institute of Child Health, University College London, London, WC1N 1EH, UK
14. Whittington Health NHS Trust, London, UK, N19 5NF

Corresponding author: Dr Logan Manikam

Email: logan.manikam.10@ucl.ac.uk
Telephone: +44 7868721152

Key Words: Community-based intervention; infant; child health; nutrition; feeding practices; clinical trial

Word Count: 3999

For peer review only

ABSTRACT

Introduction: Feeding practices developed in early life can impact a child's nutrition, growth, dental health, cognitive development and lifetime risk of chronic diseases. Substantial evidence suggests ethnic health inequalities, and non-recommended complementary infant feeding practices among UK's South Asian (SA) population. Nurture Early for Optimal Nutrition (NEON) aims to use Women's group Participatory Learning and Action (PLA) cycles to optimise infant feeding, care and dental hygiene practices in SA infants <2 years in East London.

Methods and analysis: A three-arm pilot feasibility cluster randomised controlled trial will assess feasibility, acceptability, costs and explore preliminary effectiveness for proposed primary outcome (i.e. reporting on BMI z-score). Multilingual SA community facilitators (CFs) will deliver the intervention, Group PLA Cycle, to mothers/carers in respective ethnic/language groups. 12 wards are randomised to face-to-face PLA, online PLA and usual care arms in 1:1:1 ratio. Primary outcomes are feasibility and process measures (i.e. BMI z-score, study records, feedback questionnaires, direct observation of intervention, and sustainability) for assessment against Go/Stop criteria. Secondary outcomes are cluster-level and economic outcomes (i.e. eating behaviour, parental feeding practices, network diffusion, children development performance, level of dental caries, GP utilisation, costs, staff time). Outcomes are measured at baseline, every two weeks during intervention, 14-weeks and at 6-months by blinded outcome assessors where possible. This study will use concurrent mixed-methods evaluation. Quantitative analyses include descriptive summary with 95% confidence interval (CI) and sample size calculation for the definitive trial. The intervention effect with CI will be estimated for child BMI z-score. Implementation will be evaluated qualitatively using thematic framework analysis.

Ethics and dissemination: Ethics approval was obtained from UCL, NHS (HRA and HRCW). Results will be published in peer-reviewed journals, presented at scientific conferences/workshops with commissioners, partners and participating communities. Plain language summaries will be disseminated through community groups, websites and social media.

Word Count: 299/300

Trial registration: This trial will be registered with the International Standard Randomised Controlled Trial Number Register: IRAS-ID – 296259 (ISRCTN10234623).

For peer review only

ARTICLE SUMMARY

Strengths

- The three-armed approach will allow for comparisons of online and face to face delivery with usual care
- The collection and interrogation of process and feasibility data with clearly defined stop-go criteria will allow for an informed decision around feasibility of a larger scale evaluation
- A broad set of secondary outcome measures will allow for estimation of potential impact, in addition to understanding of the feasibility of capturing reliable and complete data measuring these outcomes

Limitations

- Project capacity precludes the spacing of group sessions according to religious celebrations which might affect retention to group session.
- Risk of performance and measurement bias as neither participants nor all research staff will be fully blinded.

For peer review only

INTRODUCTION

The first 1,000 days of life is important for growth and brain development. There is mounting evidence that influences during pregnancy and infancy may alter lifetime risk of nutrition and dental related diseases [1]. Feeding practices developed during this period can impact children's nutrition, growth, dental health, cognitive development, and may lead eventually to heart disease, obesity, and diabetes [2].

Britain's ethnically diverse population is mostly disadvantaged across a range of socioeconomic outcomes, forming fundamental causes of ethnic health inequalities in the UK [3]. Some of the widest differences have been observed in the South Asian (SA) population, more so for Pakistani and Bangladeshi communities compared to Indians [4].

Systematic reviews assessing complementary feeding practices and the sociocultural beliefs underpinning them in children <2 years within SA families in the UK were explored. Despite the adoption of the WHO Infant and Young Children Feeding Guidelines, there remains substantial evidence of non-recommended complementary feeding practices such as early introduction of solids, introduction of minimum dietary diversity or minimum frequency of meals being followed [5-7]. Contributing factors that persisted post-migration included bicultural issues or low acculturation levels and conflicting information among health professionals, extended family, and religious and community leaders.

Effective early life interventions tailored to different ethnic groups have great potential to reduce the development of short- and long-term conditions and, thereby, lifetime inequalities. At present, however, few of such interventions exist [8]. Traditionally, UK health services provide unidirectional information based on guidelines and NHS recommendations. Specific ethnic groups may be marginalised by this approach as most advice is not tailored to their cultural practices. There is a need for interventions that target these communities through a top-down unidirectional approach and bring a change originating from within the communities. Therefore, it is important to work in partnership with communities thereby building their capacity to work closely with local authorities/stakeholders. The asset-based community development approach (ABCD) has been successful in community development as this helps communities identify their assets through asset mapping and mobilize them to bring along the desired change [9].

Based on Lakhanpaul et al.'s MARKS (Motivation Awareness Resources Knowledge and Skills) model [10], current practice lacks the space to understand parents' motivation and ability to support families in skills development, all of which is key to providing optimal infant feeding, care, and dental hygiene support. Considering the resource constraints of the NHS and its 10 Year Forward Plan which aims to shift the emphasis to the community, highlighting a need for interventions that enable communities to utilise their available community assets. The Participatory Learning and Action (PLA) cycle is an iterative process led by multilingual facilitators through a four-stage cycle of identifying and prioritising contextual issues, designing problem-solving strategies, implementing these strategies and a post-implementation evaluation. The PLA approach is a low-cost, community-based, culturally

For peer review only - http://bmjopen.bmj.com/site/about/guidelines.xhtml

sensitive intervention that can be adapted from Low- and Middle-income Countries (LMICs) to High-income Countries (HICs), as well as to different population groups and topic areas. This strategy is also recommended by the WHO to improve maternal and infant survival [11]. A meta-analysis of seven cluster randomised controlled trials (RCTs) utilising PLA in LMICs showed reduced maternal and neonatal mortality by 37% and 23% respectively [12].
An RCT and controlled before-and-after study have demonstrated reduced maternal depression, increased exclusive breastfeeding rates, and decreased under-5 morbidity [13]. A Mumbai-based RCT also showed improvements in maternal practices and care-seeking behaviour [14]. Recognising WHO recommendations and the success of PLA in LMICs, this approach was adopted for the Nurture Early for Optimal Nutrition (NEON) programme.

In NEON Phase 1 (Intervention Development) was co-developed with the SA community facilitators (CF) (now community researchers/independent observers in NEON Phase 2), community members, research team and independent experts (i.e. health visitors, dentists, dieticians, nutritionists, GPs, and midwives). This followed the MRC complex intervention framework , which involved using formative research and prior trials as evidence-based factors; micro-adaptations involved adjusting language, literacy levels for materials, using picture cards, face-to-face, and interactive learning delivery methods. This protocol describes NEON Phase 2: Pilot Feasibility Cluster RCT.

AIMS AND OBJECTIVES

This pilot feasibility study aims to evaluate the feasibility and inform the design and conduct of a definitive cluster RCT comparing NEON Women's Group PLA Cycle versus usual care to optimise infant feeding, care, and dental hygiene practices in SA infants aged <2 years in East London.

Primary objectives:

Assess: 1) the feasibility of proceeding to a definitive trial against predetermined Go/Stop criteria (i.e. recruitment, retention rates, intervention support, acceptability),2) intervention fidelity and participant' adherence, 3) implementation of face-to-face versus online intervention arms

Secondary objectives:

Assess: 1) feasibility, completeness, acceptability and adequacy of blinding when collecting proposed outcomes to establish optimal outcomes and data collection procedures, 2) time needed to competently deliver both versions of NEON intervention, 3)the mean, standard deviation and intervention effect with 95% confidence interval (CI) of the proposed primary outcome, child BMI z-score

For peer review only - http://bmjopen.bmj.com/site/about/guidelines.xhtml

METHODS AND ANALYSIS

Design

This three-arm pilot feasibility cluster RCT study is expected to run from July 2021-May 2023 in 3 East London boroughs. 18 clusters defined as borough wards will be randomised with 1:1:1 allocation to 2 intervention arms (face-to-face or online NEON Women's Group PLA Cycle) and 1 control arm (usual care). An online arm enables the trial to continue under Covid-19 restrictions; usual care was selected as control to avoid depriving participants of essential and available infant and maternal care services. Multilingual CFs will deliver interventions in respective ethnic/language groups. Ward-level randomisation is a trade-off between minimising contamination and ensuring maximum SA population representation given the diversity in the ethnic makeup, i.e. SA sub-groups, of the aforementioned boroughs.

Patient and Public Involvement

The research has been co-designed with SA CFs involved in all stages of developing and evaluating the intervention to ensure relevant research questions and an acceptable study design. Development phase, representing different SA communities, are now recruited as Community Researchers (CRs) to support; protocol development and ethics application by ensuring participants-facing documents are clear, appropriate and sensitively-worded; topic guide and questionnaire development; strategy development and troubleshooting (e.g. recruitment); interpretation of findings into appropriate and attainable recommendations for practice; review and revision of draft academic papers; dissemination activities and development of plain language summaries.

Setting

This study will run sequentially in the London Boroughs of Tower Hamlets (TH), Newham (NH) and Waltham Forest (WF), all of which are considered to be among 5% most deprived in England [15]. The boroughs were analysed in terms of ethnic make-up; in each borough, the 6 wards with the highest density of Asian population were selected based on census data [16]. The most common SA ethnic/language groups will be targeted. Figure 1 shows the number of wards and PLA groups by boroughs. Figure 2 shows the overview of participant flow throughout the trial.

Figure 1. Overview schematic detailing number of wards & PLA groups by East London boroughs

Figure 2. Overview of the NEON pilot feasibility RCT design and participant flow

For peer review only - http://bmjopen.bmj.com/site/about/guidelines.xhtml

Randomisation

Randomization of wards will be done before the recruitment by a separate member at UCL using the Research Randomizer software for the 18 wards stratified by borough [17]. The randomised wards will be shared with the RA at the end of recruitment. Once sufficient numbers are recruited in an ethnic/language group, the RA will assign participants to their respective arms. Recruitment will be performed by NHS personnel who will not be aware of the randomisation. CFs/CRs will be made aware of the participant allocation before the PLA sessions start to make contact and invite participants

For peer review only

Eligibility Criteria

Participants

Inclusion

- Mothers or female carers of an infant <24 months;
- Indian, Pakistani, Sri Lankan or Bangladeshi background;
- Resident in a randomised study ward in TH, NH and WF;
- Willing and able to provide informed consent

Exclusion

- <18 years old;
- Anticipating moving out of *a priori* defined geographical areas before or after delivery
- Current or recent participation in another study within 4 weeks of trial commencement.

Staff (Community Facilitator) Eligibility

- Female
- Have at least one child, preferably <24 months
- From the SA community in TH, NH, or WF
- Able to read and write
- Fluent in English and another local language (Hindi, Bengali, Sylheti, Urdu, or Tamil)
- Understand SA social norms, values and culture within study boroughs
- Known and respected by their local community
- Motivated to address issues related to infant growth and development
- Able to manage a group and have some leadership qualities

Sample size

With communication and cultural sensitivity as key drivers of the NEON intervention, the sample size will partly depend on the coverage of ethnic and language groupings. The choice of ethnic/language groups were informed by the 2011 ONS Census [16] and input from CFs stressing that, while language is important for communication, people's practices are embedded in different ethnicities.

The main ethnic/language group(s) identified are: 1 group in TH (Bangladeshi/ Bengali & Sylleti); 4 groups in NH (Indian/Gujrati, Indian/Punjabi, Pakistani/Urdu, and Sri Lankan/Tamil); 3 groups in WF (Indian/Punjabi, Pakistani/Urdu, Sri Lankan/Tamil). In each borough, 1 Women's Group PLA Cycle will run per ethnic/language group per ward.

We aim to run 20 to 32 Women's Group PLA Cycle (with equal numbers of face-to-face and online PLA groups) at 6-8 participants per PLA group. Including control groups totals to 288-384 participants. Assuming an 80% recruitment or retention rate, this enables us to be 85% confident that the true population rate will fall between 0.76%-0.84% (precision=0.04). This was estimated using a geometric mean cluster size=16 participants, cluster size coefficient of variation (COV)=0.7 and Intra-Cluster Correlation Coefficient (ICC)=0.02.

Recruitment

Participants

Due to the diversity of the target population, multiple recruitment methods are needed to maximise reach and minimise inequalities or non-representative samples [18] [19]. For instance, relying solely on online recruitment may be ineffective due to digital poverty. Thus, other mediums including leveraging on existing social networks (snowballing), identifying and fostering collaborations with

community leaders, and creating clear and succinct recruitment materials [20] were employed. All study promotional materials (e.g. participants information sheets, posters) will be available in English and local languages. To standardise the recruitment process, we will provide the same recruitment script and study materials to all recruiters. Potential participants will contact the RA, who will confirm eligibility, consent and register them into the study.

Participant recruitment will follow three strategies with regular advice by CRs to maximise reach:
1)Recruitment through CFs: CFs will share study materials with their network to invite eligible participants with subsequent snowballing. We will provide training to standardise CF recruitment. 2)Recruitment through Health Visitor (HVs), GPs, and Midwifery teams: HVs, GPs, and midwives who have been identified based on their geographical proximity to study wards will share study materials to eligible participants. Details of those interested will be given to the RA. 3)Online recruitment: Online social media campaigns on Facebook and Instagram will be utilised. See Supplementary Material-1 for advertisement materials. Recruitment bias will

For peer review only - http://bmjopen.bmj.com/site/about/guidelines.xhtml

be limited due to the above-mentioned multiple channels of recruitment and all recruiters will be blinded thereby not knowing the allocation details at the time of recruitment.

Staff

We will recruit CFs from our local stakeholders' network. Recruitment via CFs is particularly useful for studies with underserved or traditionally hard-to-reach populations [21]. CFs are leaders/champions in their local community who, with training and support, help improve the health and wellbeing of their communities. In line with NIHR's "INVOLVE" guideline, CFs will be paid £30 for each PLA meeting and additional £5 to cover travel costs for face-to-face meetings. A comprehensive manual for the CFs will be developed with PLA experts. A tool kit will be provided to support the learnings. The CFs will attend a three-day comprehensive workshop and additional sessions will be conducted in between the 8-meeting cycle as a refresher and address any challenges. Biweekly meetings with the RA will be conducted to discuss learnings from the sessions. The CFs will be paid through vouchers for their time similar to a zero-hour contract. HV, GP and midwives would be identified and recruited by our study partners at each study ward. Payments will be supported by NHS support costs and decided with the relevant clinical lead.

Allocation concealment and blinding

Firstly, treatment allocation will be concealed from participants and recruiters during participant recruitment to reduce recruitment bias. After recruitment has been completed for that ethnic/language group in the borough, the RA will reveal ward allocation to participants and CFs via text or email, listing postcodes by trial arms to allow CFs to effectively organise and deliver the intervention.

Secondly, outcome assessors (CRs) will be blinded where possible to reduce measurement bias. This will be achieved by CRs logging the data collected onto an electronic platform that automatically links participant ID to maintain blinding. Baseline measurement of BMI will be done by health visitors in each borough who will be blinded.

Intervention

NEON Women's Group PLA Cycle

The co-adapted NEON Women's Group PLA Cycle tailored to ethnic/language groups will be delivered face-to-face at community/children's centre or online over 14 weeks (1 session per 2 weeks). HVs, GP, and midwives' teams will be invited to 1-2 sessions to provide evidence-based information upon participants' request. To reduce study contamination, participants may not switch wards (i.e. switch arms) after treatment allocation is revealed. Figure 3 illustrates the aim of each PLA session.

Figure 3. NEON Women's Group PLA Cycle

A culturally-sensitive intervention package has been co-developed consisting of:

1. PLA group facilitator manual
2. Picture cards with recommended and non-recommended infant feeding, care and dental hygiene practices, and facilitators/barriers to uptake
3. Healthy baby food recipes

For peer review only - http://bmjopen.bmj.com/site/about/guidelines.xhtml

4. Community asset maps (e.g. low-cost fruit/vegetable shops)
5. List of relevant resources and services.

Online arm participants may access this toolkit through the eRedbook platform and receive NEON information the via the app or website. A Logic Model (Supplementary Material-2) shows the relationship between these activities and resources and their intended short-, medium- and long-term effects.

Control (Usual Care)

In all wards, HV teams have regular mandatory postnatal visits for all families of new-borns and infants. These visits are immediately after birth, 6-8 weeks, 12-16 weeks, 1 year, and 2-2.5 years. One optional prenatal visit is conducted by HVs between 28-32 weeks of pregnancy. Supplementary Material-3 lists other initiatives and resources available in each study borough as part of usual care.

Outcome

Primary outcomes: Feasibility and process outcome measures
We will collect quantitative and qualitative data to assess Go/Stop criteria (Table 1) on recruitment and retention rates, intervention support, acceptability, fidelity and participant adherence using:

1. *Study record:* An enrolment log will record all eligible participants, total enrolled, reasons for non-participation, number followed-up on child BMI z-score, and the date on how many people responded to adverts/invitations. Pre-screen failure logs will record those eligible but not enrolled, with reasons. PLA session audio/video recordings will be collected using secured Dictaphone, video cameras, CCTV, and/or Zoom recording.

2. *Process measurements:* These include Participants' Feedback Questionnaire, Facilitator's Feedback Form, PLA Cycle Meeting Register, direct observation of intervention delivery and CF performance by CRs, and Sustainability Assessment Tool (Supplementary Material-4).

Definite Go	Definite Stop
>=50% of eligible participants consenting to pilot feasibility study	<40% of eligible participants consenting to pilot feasibility trial
>=80% of mothers attend >=60% of planned sessions in the intervention arm.	<20% of mothers attend >=60% of sessions as planned in each intervention arm
Retention of >=70% of consented participants for primary outcome data collection	Retention of <50% of participants at 6 months
High intervention support with respect to content, frequency, duration, and quality	Low support of Intervention Procedures

For peer review only - http://bmjopen.bmj.com/site/about/guidelines.xhtml

Intervention is perceived as acceptable	Intervention perceived as unacceptable

Table 1: Go/ Stop criteria for the definitive trial[3]

Secondary outcomes: Proposed outcomes for the definitive trial

All other individual-level, cluster-level and economic outcome measures proposed for the definitive trial will be assessed on the response, completion rates, acceptability and the adequacy of blinding for outcome assessors during data collection. Table 2 summarises outcome measures and data collection timing. See Supplementary Material-5 for collection tools.

Outcome	Outcome Measures	Data Collection	Timing			
			Baseline	Every 2 weeks (end of each PLA meeting)	14 weeks (end of PLA cycle)	6 months
**Proposed primary individual-level outcome**	Individual Child BMI z-score	RA, CRs	✓		✓	✓
**Proposed secondary individual-level outcome**	Children feeding behaviour[3]	RA, CRs	✓		✓	✓
	Parental feeding style[3]	RA, CRs	✓		✓	✓
	Audio/Video recording of child eating behaviours & parental feeding practices	RA	✓		✓	✓
	4-day food diary[4]	RA, CRs	✓		✓	✓
	Network diffusion†[3]	RA, CRs	✓			✓
**Proposed secondary cluster-level outcome**	Children's development performance	RA, CRs		✓		✓
	Level of dental caries	RA	✓			✓

[3] The study will have to meet the Definite Go criteria in order for the study to be feasible and to be able to proceed. However, if any one of the Definite Stop criteria is met then the study stops. The 'Go/Stop' indicates that it is either feasible or not feasible to proceed to a definitive trial. The progression rules have been approved by the steering and data management team. Should any of the progression rules not be met, assessments and adjustments of the NEON pilot feasibility RCT will be negotiated before proceeding to a definitive trial.

[4] Community Facilitators will assist in administering or completing these data collection tools before sending them to RA/ CRs during the intervention period; †For intervention groups only; CR= Community Researchers; RA = Research Assistant

For peer review only - http://bmjopen.bmj.com/site/about/guidelines.xhtml

	GP healthcare utilisation	RA	✓			✓
**Economic outcome**	Cost tool	RA	✓			✓
	Partners time questionnaire	RA				✓
**Process outcome†**	Participants Feedback[3]	RA, CRs		✓		
	PLA cycle meeting register[3]	RA, CRs		✓		
	Direct observation	RA, CRs		✓		
	Sustainability assessment	RA, CRs		✓		

Table 2. Schedule of Outcome Assessments

Primary individual-level outcome

1. *Child BMI z-score:* Health visitors will measure infants' length/height, head circumference and weight. The RA will refer to the WHO 2006 growth standard [2], using age- and gender-adjusted length or height(m)/weight $(kg)^2$ to provide a comparison on the cluster-level.

Secondary individual-level outcomes

1. *Children's feeding behaviour* will be measured using 6 of 8 domains adapted from the validated Children's Eating Behaviour Questionnaire (CEBQ) deemed suitable for infants: food responsiveness; enjoyment of food; emotional overeating; desire to drink; satiety responsiveness; slowness in eating; emotional undereating; and food fussiness [7, 8, 22]. Obesity risk could be linked with external eating, emotional overeating, and food fussiness [7].

2. *Parental feeding style* will be assessed with a validated self-reported Parental Feeding Style Questionnaire (PFSQ) with four scales: emotional feeding; instrumental feeding; encouragement to eat; and control overeating [23].

3. *Audio/video recording of child eating behaviour and parental feeding practices* [24] involves asking participants about the meal (e.g. name, ingredients) and creating annotations that link to behaviour codes identified from NEON formative study [25]. Before-and-after audio/video recordings will be thematically analysed using Elan Software [26] to measure intervention effect.

4. *Child food intake*: The 4-day food diary [7] is a self-reported, prospective, open-ended survey that collects detailed quantitative estimates of food consumption of infants between 4-18 months [6]. It assesses nutrient intake (e.g. total fat, total carbohydrate, salt, sugar) by comparing with age/sex specific UK dietary reference values [27]. Dietary data will be presented by age group (e.g. 4-6 months, 7-9 months, 12-18 months).

5. *Network diffusion of study materials between participants and their communities*: CRs will track the number of downloads using the eRedbook platform. Participants will also complete a questionnaire on the number of people they shared the material with, their family size, age, gender, relationship to the participant and platform used.

6. *Equality impact assessment (EIA)*: This trial should reflect the diversity of the target population by enabling equal access while accommodating different needs. An EIA tool will systematically assess likely effects of the intervention on people (e.g. with respect to disability,

For peer review only - http://bmjopen.bmj.com/site/about/guidelines.xhtml

gender, ethnicity, age, sexual orientation, religion/belief etc.), mitigate adverse impacts and identify active steps to address existing disadvantage and promote equality.

Secondary cluster-level routine outcomes

Although this intervention acts on the individual-level, we expect a social diffusion of good infant feeding and dental hygiene awareness and practices in the medium- to long-term (Supplementary Material-2, Logic Model). While immediate cluster-level changes are not expected, we would like to pilot cluster-level routine data collection.

1. Development performance of children: The Ages and Stages questionnaire (ASQ-3) include communication, gross motor, fine motor, problem-solving, and personal-social domains [5]. Three versions are available: 24-months, 27-months and 30-months. ASQ-3 data collected by HVs during mandated visits are stored in EMIS/ RIO databases.
2. GP healthcare utilisation data is available in participants' medical record.
3. Level of dental caries and other signs of tooth decay is available in NHS dental services records for registered patients beginning at 6-months of age and approximately every half-year following initial visit [17,28,29].

Economic outcomes

1. Cost: The RA will use a spreadsheet will capture the cost of the NEON Intervention Development
and pilot feasibility RCT phases every 6 months.
2. Partners' time used: A questionnaire will capture time spent for the delivery of NEON-associated programmes.

Data Collection and Management

Outcome data will be collected at baseline, every 2-weeks during intervention (for process measures), 14-weeks immediately post-intervention, and 6-months follow-up (for economic data). During the intervention period, several outcome measurements will be administered by CFs to participants or completed by CFs and sent to CRs/RA. Other outcomes and post-intervention outcomes will be assessed directly by CRs/RA (Table 2). Each case will be assigned an ID. Data collected digitally or in paper format will be stored in UCL S: Drive and/or UCL cabinets. We will use RedCap electronic data management system [30]; the RedCap API allows flexible and straightforward data import and export to data analysis software.
We will form a data sharing agreement with study partners for the RA to extract and link routine cluster-level data to trial participants. To guarantee confidentiality, pseudonymised participant data will be stored on encrypted, password-protected computer or UCL S:Drive accessible only to study staff and authorised personnel. Password-protected personal data will be stored separately from trial data on secure UCL computers. Only pseudonymised quotes or data from audio/video recording may be published. Personal data and audio/video recordings will be stored for 3-years post-study and destroyed; research data will be stored for 20-years post-study.

Analysis

For peer review only - http://bmjopen.bmj.com/site/about/guidelines.xhtml

Overall, quantitative and qualitative data will be analysed concurrently at multiple time points to identify early problems that are rectified as the trial progresses. Quantitative data such as the BMI z-score will be descriptively summarised using mean and standard deviation (SD) for continuous variables, number with percentages for categorical variables, and 95% CI with a breakdown of participants by trial arm, ethnic/language group and borough where relevant. An intention to treat analysis will also be considered. Results will be collectively assessed against the Go/Stop criteria. Before progression to the definitive trial, we will systematically and rigorously assess areas of improvement using a structured discussion tool: A process for Decision-making after Pilot and feasibility Trials (ADePT) [31]. All statistical analysis will be done by an independent statistician who would be blinded.

Sample Characteristics

Participant demographic characteristics will be descriptively summarised by trial arm. We will check for recruitment bias across different arms at baseline and compare later recruits who are prone to unblinded recruitment.

Feasibility and Process Measures

Across the three arms, we will check for any differential recruitment or retention rates and any consequent systematic differences in participant characteristics over time, as control arm participants may be less keen to participate or remain in the study. For the two intervention arms, we will conduct a mixed-methods implementation evaluation to explore the possibility of a blended approach in the definitive trial as some features of each delivery mode might be particularly salient. Qualitative data will be analysed thematically using framework analysis that include participants' motivation for engagement, expectations, experiences, intervention acceptability, implementation barriers, and suggestions for improvement. Negative 'deviant' cases will inform interpretation.

Outcome measures for the definitive trial

1. *Feasibility of collecting outcome measures:* To assess completeness, degrees of missingness at the individual item level and the entire outcome measure will be reported overall, by time-point and trial arms. Other feasibility measures would be captured by analyses of process measures above.

2. *Intervention effect for proposed primary outcome (BMI z-score):* Conditioned upon low recruitment bias and sufficient adherence to trial protocol, we will estimate the difference in BMI z-score between each intervention arm versus the control arm. We will consider methods to pool data across age groups depending on the actual sample age breakdown.

3. *Sample size and power calculation for the definitive trial:* Data from the pilot feasibility trial will enable us to determine (i) the smallest difference of clinical importance (through multiple testing of several key endpoints for the proposed primary outcome), (ii) the clinically justifiable power-significance level or scientifically acceptable probability of "false positive" (type I error) results, and (iii) whether we need to adjust the calculated sample size for expected level of non-compliance/ drop-in and drop-outs. We will report the ICC.

4. *Economic Evaluation:* This will be conducted from a provider and user perspective which includes direct cost (e.g. time, resources spent on training and participation of CF, participants' overall household consumption and childcare costs). We will estimate the total cost, average annual cost, cost-effectiveness, equity impact analysis and compare across three arms.

Monitoring

The Principal Investigator (PI) will be responsible for day-to-day monitoring and study management.

For peer review only - http://bmjopen.bmj.com/site/about/guidelines.xhtml

The NEON Steering and Data Management Team will ensure adherence to all relevant regulations and principles of good clinical practice. The team consists of multidisciplinary experts in child health, practitioners, PLA expert, SA independent observers from NEON 1, and Queen Mary University of London Pragmatic Clinical Trials Unit. Committee meetings will be held every 3 months to review the Go/Stop criteria (Table 1) and interim analyses. The team will agree to the trial protocol and facilitate any necessary protocol amendments; provide independent expert advice (including on project management governance); monitor progress; ensure adequate deadlines are set and met; monitor and advise NEON Core Team (Trial Management Group) on strategic decisions in light of new evidence; and ensure successful delivery by meeting and reporting on study progress with the Core Team biannually. The PI and other Core Team members will attend all necessary meetings and report on the study progress.

Any adverse events or safety concerns will be captured in the Participant Feedback Questionnaire and Facilitator Report Form (every 2-weeks during intervention period) and reported to the steering and safety monitoring team.

The UCLH/UCL Joint Research Office, on behalf of the Sponsor (UCL), may conduct random audits in accordance with the UK Policy Framework for Health and Social Care Research and UCL's policies and procedures.

ETHICS AND DISSEMINATION

Ethical approval will be obtained from UCL Research Ethics Committee and NHS Health Research Authority. The participant information sheet will be provided in English or participants' native language in-writing and/or verbally by community facilitators for informed consent. The RA or CRs will obtain written or audio-recorded consent ≥24 hours later [32]. Participation will be voluntary and they may withdraw anytime without prejudicing treatment or usual care. No incentives will be provided except for small reimbursements (e.g. childcare at the local centre where PLA meetings take place).

UCL will provide insurance cover and we will also signpost any vulnerable participants who require protection from harm to relevant safeguarding organisations.

Regular progress updates will be shared via UCL or NIHR websites and patient group newsletters. Study findings will be submitted for 3-5 publications in high impact factor peer-reviewed journals and presented at national and international conferences. Researchers and community members from the steering team will contribute to confirming analysis and write-up, adhering to authorship guidelines. To reach wider audiences, we will (i) disseminate plain language summaries for each academic paper through community groups, social media and You Tube; (ii)present findings at lay-person meetings at community or children centres through the NIHR CLAHRC network and (iii)organise 2-3 annual workshops with commissioners, partner organisations and NEON community facilitators to share key findings and recommendations.

Acknowledgements

The authors would like to thank the South Asian community facilitators in the NEON Intervention Development phase: Lily Islam, Seema Bajwa, Aasma Baiyat, Jasvir Bhachu, Geromini Pushpakanthan, Sabiha Malek, Cllr Lakmini Shah; and community members of the London Boroughs of Tower Hamlet, Newham, and Waltham Forest for their important contribution and engagement to this research project. Furthermore, the authors would like to thank the 'Conversation Café' initiative led by Cllr

For peer review only - http://bmjopen.bmj.com/site/about/guidelines.xhtml

Lakmini Shah for her significant help in the recruitment of the community facilitators and members, and in identifying the community centres to carry out the intervention development meetings. The authors would also like to acknowledge the contribution of the NEON core team, steering team and all health experts in validating intervention NEON toolkit. We would like to thank the Women & Children First Charity and First Steps Nutrition Trust for their valuable contributions and guidance throughout the study. In addition to the authors, members of the NEON steering team consist of Prof Atul Singhal, Joanna Drazdzewska, Dr Sonia Ahmed, Amelie Gonguet, Gary Wooten, Dr Ian Warwick, Vaikuntanath Kakarla, Alex Nelson, Phoebe Kalungi, Prof Richard Watt, Prof Mitch Blair, Prof Audrey Prost, Dr Edward Fottrell, Ashlee Teakle, Prof Oyinlola Oyebode, Ciarán Forde, Keri McCrickerd, Dr Rana Conway, Subarna Chakraborty, Sumire Fujita, Tala Khatib and Delaney Douglas-Hiley.

Steering team members had an opportunity to critically review results and contribute to the process of finalising this paper.

Author's Contributions

ML, LM, MH, NB, CL, AH, SA conceived the original concept of the study and designed the research methodology. SA carried out the Intervention Development meetings, workshops, co-development of the intervention toolkit, wrote and devised the paper along with LI, SB, SM, JB and GP. LM, ML, ,MH, SA, PP, MN, ZO, NB, CL, AH, RL, JG, KWM, CI, MA, CC, DD validated the study and revised the manuscript critically for important intellectual content. ICD was involved in drafting materials for the NEON meetings and workshops of the Intervention Development Phase. MN and ICD contributed to the manuscript writing, and prepared it for submission. LM, SA, ML and MH had primary responsibility for the final content. All authors read and contributed to reviewing the study data, the designing of the manuscript, and the approval of the final manuscript.

Funding

Logan Manikam, Shereen Al Laham & Priyanka Patil are funded via a National Institute for Health Research (NIHR) Advanced Fellowship (Ref: NIHR300020) to undertake the Pilot Feasibility Cluster Randomised Controlled Trial of the *NEON* programme in East London. Prof Monica Lakhanpaul was funded by the NIHR Collaboration for Leadership in Applied Health Research and Care (CLAHRC) North Thames.

Disclaimer

The views expressed in the publication are those of the author(s) and not necessarily those of the sponsor (UCL), funder (NIHR), study partners (Tower Hamlets GP Care Group, London Borough of Newham Council, Waltham Forest Council).

Conflicts of Interest

The authors declared no potential conflicts of interest with respect to the research, authorship and/or publication of this paper.

Participant consent for publication

Participant information sheets and consent forms have been provided to community members and those expressing interest. Community participants have agreed to participate and given audio/video consent prior to their participation in the workshops. Verbal consent was witnessed and formally recorded. All participants have been informed of their right to freely withdraw from the study at any

For peer review only - http://bmjopen.bmj.com/site/about/guidelines.xhtml

For peer review only

time. Confidentiality of personal data has been ensured through the use of anonymisation techniques as stated in the Data Protection Act (1998) and in line with the General Data Protection Regulation (2018). All participant data has been anonymised and stored on an encrypted password protected computer. Data can only be accessed by the authorised research personnel.

Ethics approval

This study has obtained ethical approval from UCL Research Ethics Committee [Ethics ID 17269/001], Sponsor reference number: 142600, Funding Reference: NIHR300020 and IRAS number: 296259, Ref: 21/SW/0142. Study protocols and relevant documents will be reviewed by UCL Research Ethics Committee, NHS Health Research Authority (HRA) and study partners involved in establishing data sharing agreement for linking participants' routine data (Tower Hamlets GP Care Group, London Borough of Newham Council, Waltham Forest Council).

Provenance and Peer Review

Not commissioned; peer reviewed for ethical and funding approval prior to submission.

Data Sharing Statement

The data supporting the findings of this study is available upon reasonable request from the corresponding author.

CURRENT STUDY STATUS (AS OF JAN 2023)

The study is currently in the intervention delivery phase.
There has been an overall delay in the timeline of the study due to the COVID - 19 pandemic, lockdown and NHS staff shortage in our study wards.

Recruitment for the study commenced in March 2022 and was completed in Sep 2022. Following recruitment, we launched our first PLA meeting in Tower Hamlets in Oct 2022 and then in Newham in the week following. Waltham Forest dropped out of the study due to lack of supporting staff in the recruitment phase.

We have completed 5 out of the 8 PLA meetings and the PLA meeting cycle will conclude in Feb 2023. Following this we will initiate data analysis and reporting of study findings. We will collect follow-up data in April 2023 which will be the last data collection time point for this trial.

REFERENCES

[1] World Health Organization (WHO), Postnatal care for mothers and newborns: Highlights from the World Health Organization 2013 Guidelines. Available from: http://www.who.int/maternal_child_adolescent/publications/WHOMCA-PNC-2014-Briefer_

For peer review only - http://bmjopen.bmj.com/site/about/guidelines.xhtml

A, 2015. 4.

[2] Group, W.M.G.R.S., WHO Child Growth Standards based on length/height, weight and age. Actapaediatrica (Oslo, Norway: 1992). Supplement, 2006. 450: p. 76.

[3] Evandrou M, Falkingham J, Feng Z, et al Ethnic inequalities in limiting health and self-reported health in later life revisited J Epidemiol Community Health 2016;70:653-662.

[4] Brown MS. Religion and economic activity in the South Asian population. Ethnic and Racial Studies. 2000;23(6):1035-61

[5] ASQ-3. The Ages & Stages Questionnaires, Third Edition. [cited 2019 30 November]; Available from: https://agesandstages.com/products-pricing/asq3/.

[6] Lennox, A., et al., Diet and nutrition survey of infants and young children, 2011. A survey carried out on behalf of the Department of Health and Food Standards Agency, 2013.

[7] NATIONAL DIET AND NUTRITION SURVEY: Food and Drink Diary. 2010; Available from: https://webarchive.nationalarchives.gov.uk/20130413115649/http://media.dh.gov.uk/network/261/files/2012/07/Appendix-E_All-docs-combined-v2.pdf.

[8] 2011. Early Years Interventions To Address Health Inequalities In London – The Economic Case.[ebook] London: Greater London Authority. Available at:<https://www.london.gov.uk/sites/default/files/gla_migrate_files_destination/Early%20Years%20report%20OPT.pdf> [Accessed 5 January 2021].

[9] High S, Mckie I, Mackay M, et alO-09 Beyond traditional approaches to community engagement: using asset based community development to discover, connect and mobilise communities to support each other right to the end of lifeBMJ Supportive & Palliative Care 2022;12:A4.

[10] Lakhanpaul, M., 2019. PANCHSHEEEL - MRC HEEE STUDY. [online] Ucl.ac.uk. Available <https://www.ucl.ac.uk/child-health/sites/child-health/files/newsletter_3_0.pdf

[11] WHO. WHO recommendation on facilitated participatory learning and action cycles with women`s groups during pregnancy: The WHO Reproductive Health Library 2018 [from: https://extranet.who.int/rhl/topics/improving-health-system-performance/implementation-strategies/who-recommendation-facilitated-participatory-learning-and-action-cycles-womens-groups-during.

[12] Seward N, Neuman M, Colbourn T, Osrin D, Lewycka S, Azad K, et al. Effects of women's groups practising participatory learning and action on preventive and care-seeking

For peer review only

behaviours to reduce neonatal mortality: A meta-analysis of cluster-randomised trials. PLoS medicine. 2017;14(12):e1002467.

[13] Tripathy P, Nair N, Barnett S, Mahapatra R, Borghi J, Rath S, et al. Effect of a participatory intervention with women's groups on birth outcomes and maternal depression in Jharkhand and Orissa, India: a cluster-randomised controlled trial. The Lancet. 2010;375(9721):1182-92

[14] Prost A, Colbourn T, Seward N, Azad K, Coomarasamy A, Copas A, et al. Women's groups practising participatory learning and action to improve maternal and newborn health in low-resource settings: a systematic review and meta-analysis. The Lancet. 2013;381(9879):1736-46

[15] Mayor of London. London's socio-economic issues. 2016; Available at:https://www.london.gov.uk/sites/default/files/chapter10-economic-evidence-base-2016.pdf.Accessed 08/06/, 2020.

[16] ONS. 2011 Census Data. 2011; Available at: https://www.ons.gov.uk/census/2011census.

[17] Faculty of Dental Surgery. 2015. The state of children's oral health in England. [PDF]

[18] Newington L, Metcalfe A. Factors influencing recruitment to research: qualitative study of the experiences and perceptions of research teams. BMC Med Res Methodol. 2014;14:10. Published 2014 Jan 23. doi:10.1186/1471-2288-14-10

[19] Office for National Statistics. 2020. Domestic Abuse During The Coronavirus (COVID-19) Pandemic, England And Wales - Office For National Statistics. [online] Available at: <https://www.ons.gov.uk/peoplepopulationandcommunity/crimeandjustice/articles/domesticabuse duringthecoronaviruscovid19pandemicenglandandwales/november2020> [Accessed 10 January 2021].

[20] Joseph RP, Keller C, Ainsworth BE. Recruiting Participants into Pilot Trials: Techniques Researchers with Shoestring Budgets. Calif J Health Promot. 2016;14(2):81-89.

[21] An innovative approach to recruiting homebound older adults. Crawford Shearer NB, Fleury JD, Belyea MRes Gerontol Nurs. 2010 Jan; 3(1):11-8.

[22] Llewellyn, C.H., et al., Development and factor structure of the Baby Eating Behaviour Questionnaire in the Gemini birth cohort. Appetite, 2011. 57(2): p. 388-396.

[23] Daniels, L.A., et al., Outcomes of an early feeding practices intervention to prevent

For peer review only

childhood obesity. Pediatrics, 2013. 132(1): p. e109-e118.

[24] Fogel, A., et al., Prospective associations between parental feeding practices and children's oral processing behaviours. Maternal & child nutrition, 2019. 15(1): p. e12635.

[25] Lakhanpaul M, Benton L, Lloyd-Houldey O, et al. Nurture Early for Optimal Nutrition (NEON) programme: qualitative study of drivers of infant feeding and care practices in a British-Bangladeshi population. BMJ Open 2020;10:e035347. doi:10.1136/ bmjopen-2019-035347

[26] Tla.mpi.nl. 2020. ELAN | The Language Archive. [online] Available at: http://tla.mpi.nl/tools/tla-tools/elan

[27] Health Do. Infant feeding recommendation. Department of Health London, England; 2003.

[28] 2019. Dental Record Keeping Standards: a consensus approach. [PDF] NHS England and NHS Improvement.

[29] Faculty of General Dental Practice, 2012. 7. Collecting Data. Available at: <https://www.fgdp.org.uk/research-primary-dental-care/7-collecting-data> [Accessed April 15, 2021].

[30] Lawrence, C.E. et al. A REDCap-based model for electronic consent (eConsent): Moving toward a more personalized consent. Journal of Clinical and Translational Science. 2020; 4(4):345–353.

[31] Bugge C, et al. A process for Decision-making after Pilot and feasibility Trials (ADePT): Development following a feasibility study of a complex intervention for pelvic organ prolapse. Trials.2013 Oct 25;14(1):353.

[32] Health Research Authority. 2018. HRA And MHRA Publish Joint Statement On Seeking And Documenting Consent Using Electronic Methods (Econsent). [online] Available at: <https://s3.eu-west-2.amazonaws.com/www.hra.nhs.uk/media/documents/hra-mhra-econsent-statement-sept-18.pdf> [Accessed 8 January 2021]

For peer review only - http://bmjopen.bmj.com/site/about/guidelines.xhtml

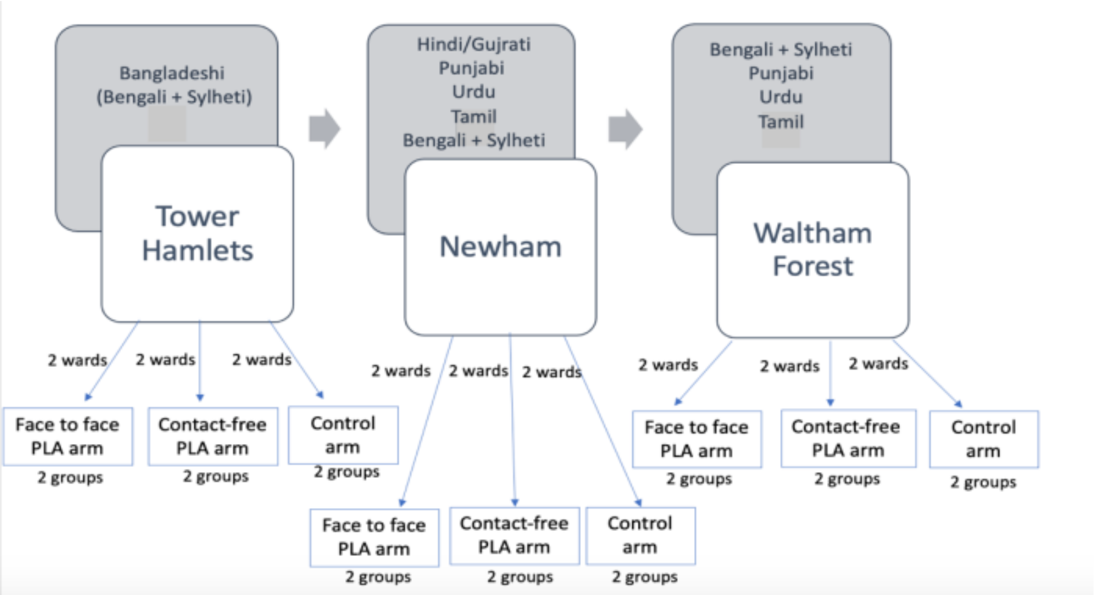

Figure 1. Overview schematic detailing number of wards & PLA groups by East London boroughs

Footnote: To illustrate, TH will have 2 face-to-face Bangladeshi/ Bengali & Sylheti PLA groups (1 per ward), 2 groups of Bangladeshi/ Bengali & Sylheti online PLA group (1 per ward), and 2 Bangladeshi/ Bengali & Sylheti control groups (1 per ward). The more the ethnic/ language group in the borough, the more the intervention and control groups.

For peer review only

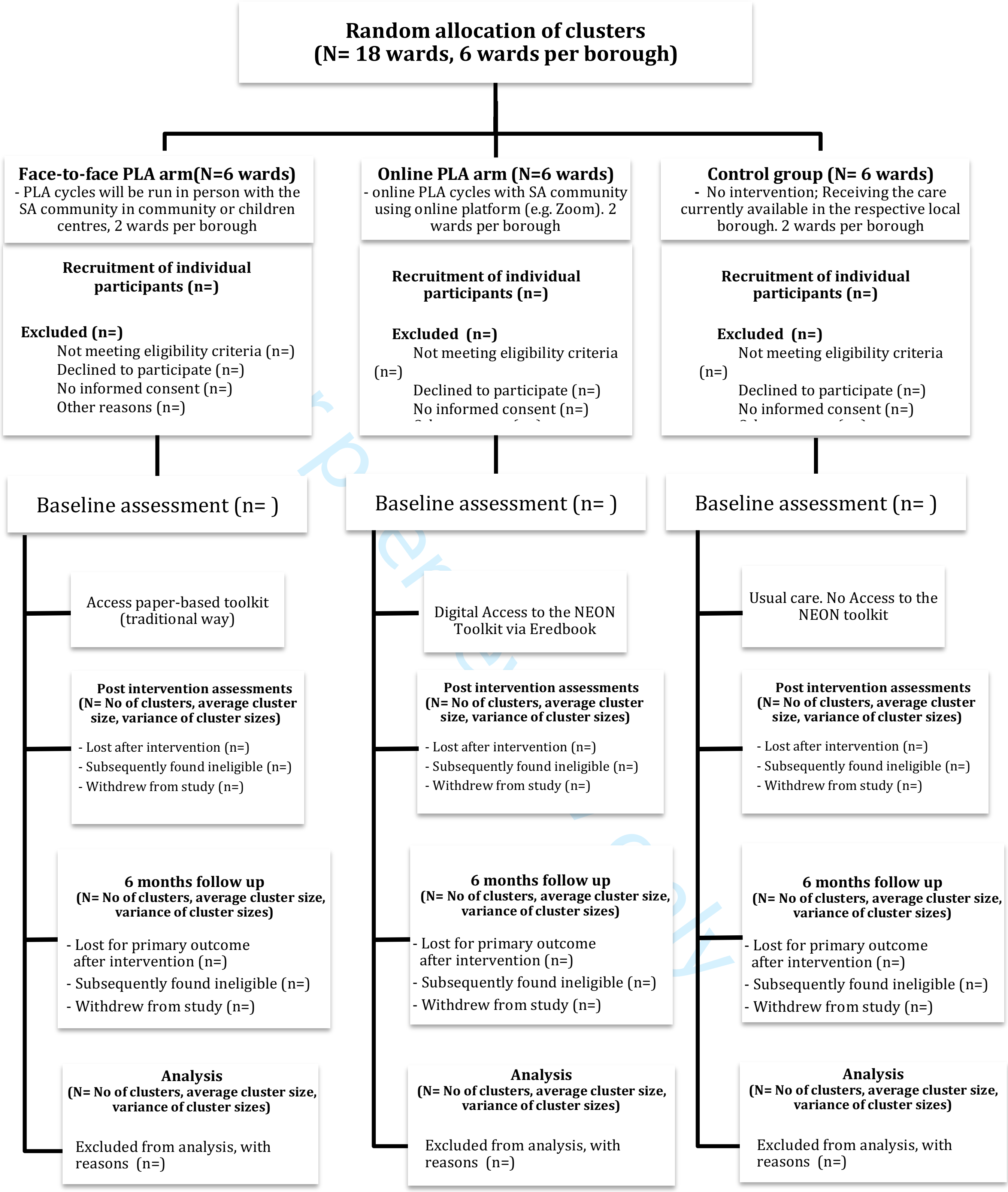

Figure 2. Overview of the NEON pilot feasibility RCT design and participant flow

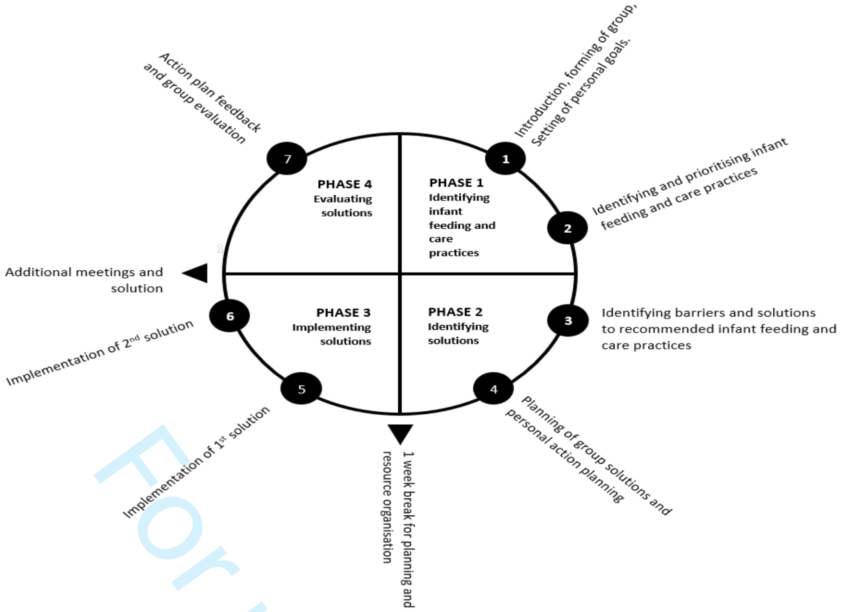

Figure 3. NEON Women's Group PLA Cycle

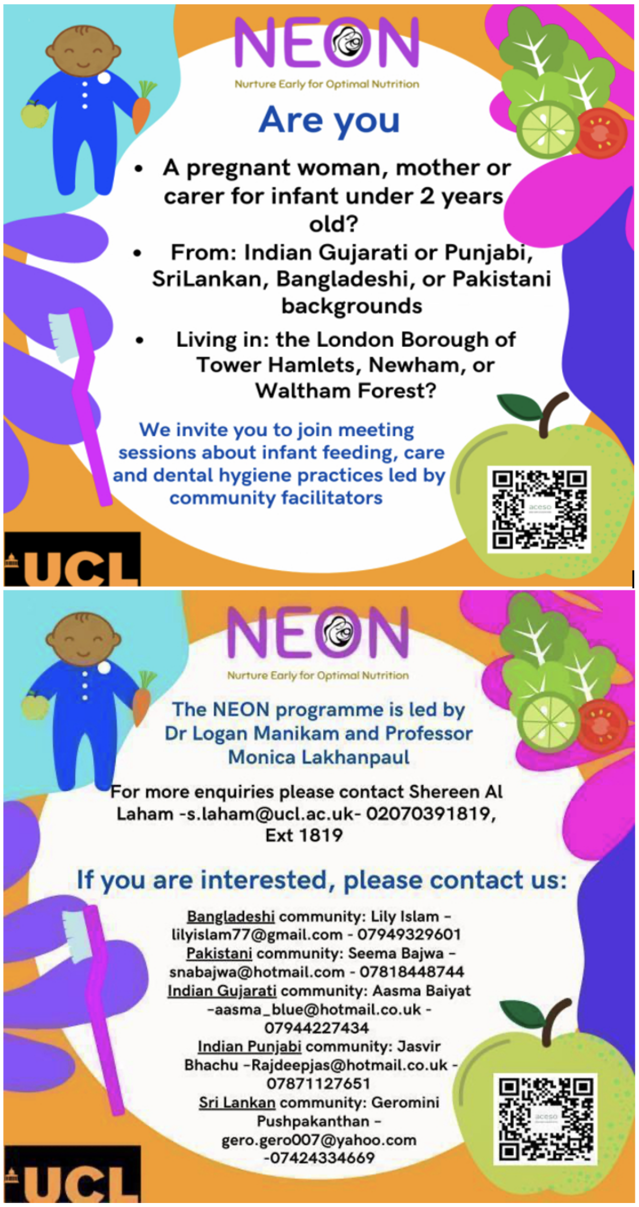

125x234mm (144 x 144 DPI)

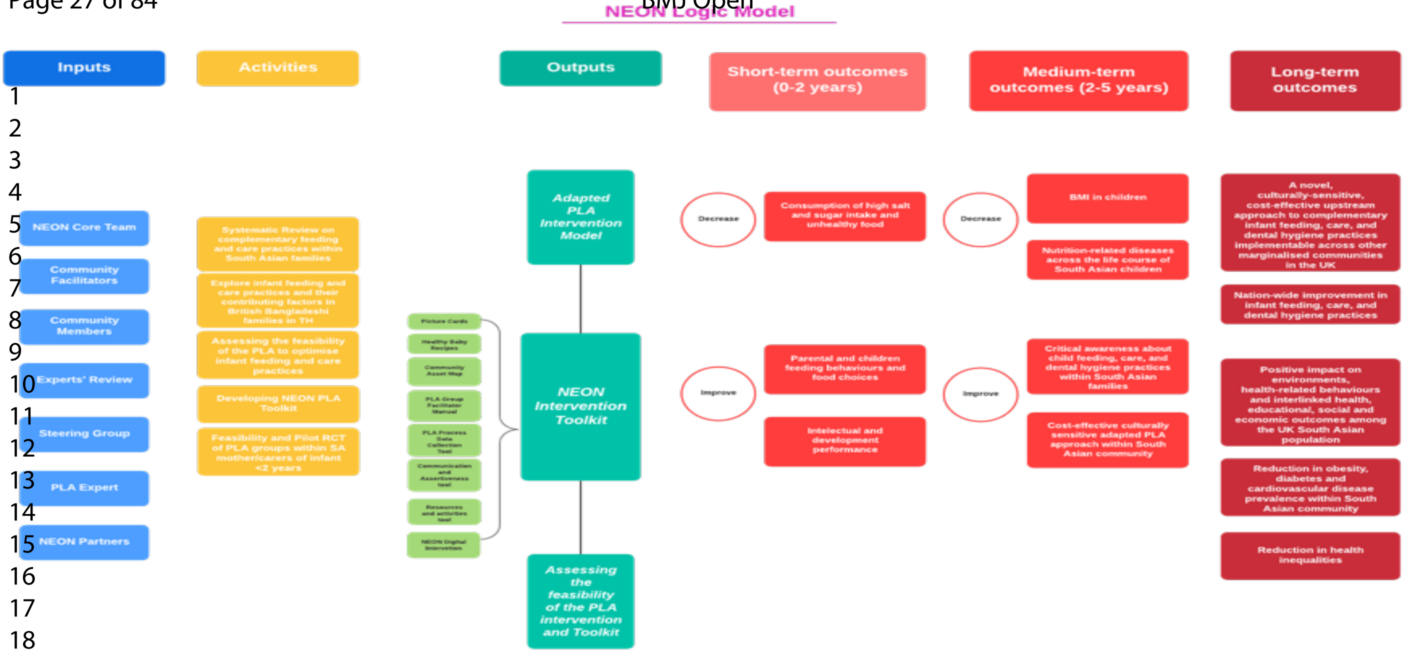
NEON Logic Model
Inputs
Activities
Outputs
Short-term outcomes (0-2 years)
Medium-term outcomes (2-5 years)
Long-term outcomes
NEON Core Team
Community Facilitators
Community Members
Experts' Review
Steering Group
PLA Expert
NEON Partners
Systematic Review on complementary feeding and care practices within South Asian families
Explore infant feeding and care practices and their contributing factors in British Bangladeshi families in TH
Assessing the feasibility of the PLA to optimise infant feeding and care practices
Developing NEON PLA Toolkit
Feasibility and Pilot RCT of PLA groups within SA mother/carers of infant <2 years
Picture Cards
Healthy Baby Recipes
Community Asset Map
PLA Group Facilitator Manual
PLA Process Data Collection Tool
Communication and Assertiveness tool
Resources and activities tool
NEON Digital Intervention
Adapted PLA Intervention Model
NEON Intervention Toolkit
Assessing the feasibility of the PLA intervention and Toolkit
Decrease
Consumption of high salt and sugar intake and unhealthy food
Improve
Parental and children feeding behaviours and food choices
Intellectual and development performance
Decrease
BMI in children
Nutrition-related diseases across the life course of South Asian children
Improve
Critical awareness about child feeding, care, and dental hygiene practices within South Asian families
Cost-effective culturally sensitive adapted PLA approach within South Asian community
A novel, culturally-sensitive, cost-effective upstream approach to complementary infant feeding, care, and dental hygiene practices implementable across other marginalised communities in the UK
Nation-wide improvement in infant feeding, care, and dental hygiene practices
Positive impact on environments, health-related behaviours and interlinked health, educational, social and economic outcomes among the UK South Asian population
Reduction in obesity, diabetes and cardiovascular disease prevalence within South Asian community
Reduction in health inequalities

RESOURCES AND SERVICES SUPPORTING INFANT FEEDING, CARE AND DENTAL HYGIENE

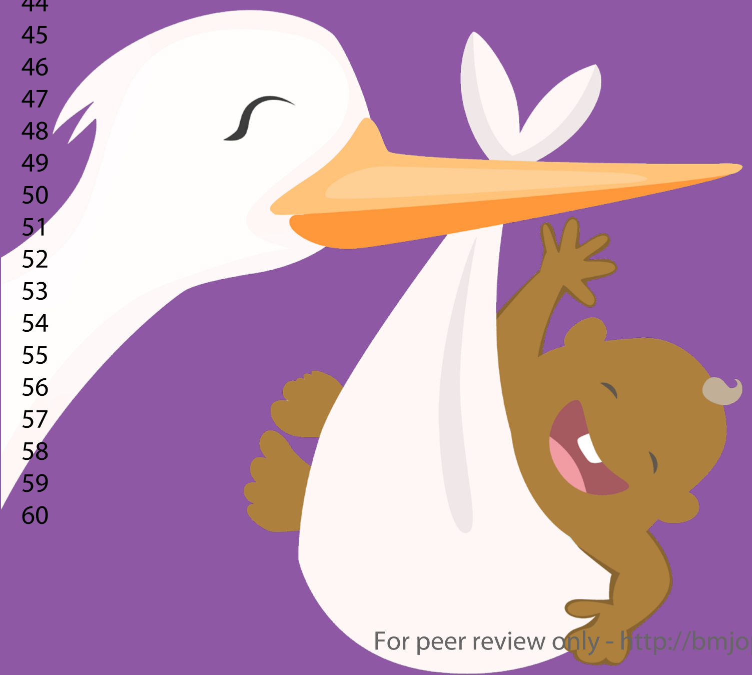

NEON
Nurture Early for Optimal Nutrition

For peer review only - http://bmjopen.bmj.com/site/about/guidelines.xhtml

NHS AND COUNCILS ADVICE AND SUPPORT

1. NHS STAR4LIFE

https://www.nhs.uk/start4life

Website provides trusted NHS help and advice for parents and carers during pregnency, and birth and the early years.

2. NHS CHOICES

NHS Choices (www.nhs.uk) is the official website of the National Health Service in England and provides comprehensive health information service with thousands of articles, videos and tools. Key pages which give guidance on infant feeding and care include:

- **Your newborn:** *https://www.nhs.uk/conditions/pregnancy-and-baby/childrens-meal-ideas/?tab-name=your-newborn*
- **Babies and toddlers** *https://www.nhs.uk/conditions/pregnancy-and-baby/childrens-meal-ideas/?tabname=ba-bies-and-toddlers*

3.CHILDREN CENTRES AND LIBRARIES

Tower Hamlets has 12 children centres across the borough providing a range of services to infants, children and families to give them the support they need to be safe, healthy and happy, so that they are able to reach their full potential. This web page on the **Tower Hamlets Council** website gives locations and contact details of all the children's centres in the borough: *https://www.towerhamlets.gov.uk/lgnl/education_and_learning/childcare_and_early_years_educ/Children_centres/childrens_centres.aspx*

The borough of Newham offers support to its residents with children under the age of 5 through **Best Start in Life (BSiL)** Children's centres. These include play activities and childminder services, as well as supporting parents to improve their confidence, support their children with healthy eating and weaning. Libraries offer free courses and internet services for parents, and they offer devices and digital equipment to borrow.

Locations and details can be found on the Families Newham website: *https://families.newham.gov.uk/kb5/newham/directory/family.page?familychannel=3-2*

Waltham Forest offers to its families a similar service, with support for healthy development and access to financial support. They have implemented the 2 Year Old Partnership Pathway in which professionals empower and inform parents to assure the best start in life for their children. They offer advice on a wide range of issues including health, finance and cognitive/motor development. **Waltham Forest** offers support directed at healthy weight and eating through HARRY.

More information can be accessed via the following link: *https://www.walthamforest.gov.uk/content/2yo-partnership-pathway*

Further informal support that does not require formal registration can generally be accessed in public libraries

For peer review only

GUIDENCE DOCUMENTS, ONLINE RESOURCES AND APP'S

1. BABY FRIENDLY INITIATIVE

Published by the Department of Health and the Baby Friendly Initiative, the leaflets below provide mothers and health professionals with key information about breast feeding, introducing solid foods to babies, and evidence-based guidance around formula. The **Baby Friendly Initiative** is part of a global partnership between the World Health Organization (WHO) and UNICEF. The guidance below should be prioritised over other online resources (other than NHS Choices) as they the baby friendly initiative is less influenced by commercial interests, which can sometimes influence the advice offered to mothers and parents regarding infant feeding and care.

- **Leaflet on weaning and starting solid food:**
  https://www.unicef.org.uk/babyfriendly/baby-friendly-resources/leaflets-and-posters/weaning-starting-solid-food/

- **Leaflet on breastfeeding:**
  https://www.unicef.org.uk/babyfriendly/baby-friendly-resources/leaflets-and-posters/off-to-the-beststart/ -

- **Leaflet on infant formula:**
  https://www.unicef.org.uk/babyfriendly/baby-friendly-resources/leaflets-and-posters/simple-formula-guide-for-parents/

- **Leaflet on bottle feeding:**
  https://www.unicef.org.uk/babyfriendly/baby-friendly-resources/leaflets-and-posters/guide-to-bottle-feeding/

- **Baby friendly initiative resources in Bengali:**
  https://www.unicef.org.uk/babyfriendly/baby-friendly-resources/leaflets-and-posters/foreign-language-resources/bengali-resources/

- **Baby friendly initiative resources in Hindi:** बच्चे के अनुकूल पहल संसाधन
  https://www.unicef.org.uk/babyfriendly/baby-friendly-resources/foreign-language-resources/hindi-resources/

- **Baby friendly Initiative resources in Urdu:** بچےکی ابتدائی نشونماء(نگہداشت)کے وسائل
  https://www.unicef.org.uk/babyfriendly/baby-friendly-resources/foreign-language-resources/urdu-resources/

Other Baby Friendly Initiative resources/leaflets:

https://www.unicef.org.uk/babyfriendly/baby-friendly-resources/leaflets-and-posters/

2. FIRST STEPS NUTRITION TRUST

https://www.firststepsnutrition.org

First steps nutrition can also be used as a trusted source of good information and guidance regarding nutrition and feeding in pregnancy, infants, and early years. **First Steps Nutrition Trust** is funded by research or charitable grants and donations, and does not take money from any commercial organisation or any organisation or individual linked to the sales of infant formula and other food or drink product or service which is associated with poor nutritional health of women and children.

For peer review only

First Steps Nutrition Trust offers some useful resources on meal choices and portion sizes, with helpful diagrams to illustrate portion size. Remember! Portion size is a maximum, not a minimum, never encourage a child to eat more when they indicate they have had enough.

- Eating well: the first year – guidance on introducing foods, food choices and meals for up to 7 months, 7-9 months, and 10-12 months: http://www.firststepsnutrition.org/newpages/Infants/first_year_of_life.html
- Eating well: Early years – guidance on food choices, meals, snacks and portion size http://www.firststepsnutrition.org/newpages/Early_Years/eating_well-early_years.html

3. BORN TO MOVE APP

Born to move is a free NHS app to help parents or carers with their newborn babies right up to pre-school, specifically relating to physical activity and play. It's packed with advice, tips and games that you can play with your child to support their development.

https://itunes.apple.com/gb/app/born-to-move/id976095022?mt=8Local%20initiatives/projects

https://play.google.com/store/apps/details?id=com.project.Born&hl=en_GB

4. EREDBOOK

ERedbook is an app based digital version of the paper Redbook. It provides solutions for parents that allows them to review their child's NHS records whilst providing health information and guidance from NHS and other sources. Additionally, it contains personalised information for new parents, including relevant videos and information from local sources & reminders and appointments updates. Parents can review the NHS child's health records on the app when they connect record to NHS. Also, The NEON participants will be able to review the NEON toolkit and receive correct information via the ERedbook app at any time and place.

https://www.eredbook.org.uk

https://apps.apple.com/gb/app/eredbook/id606443658

https://play.google.com/store/apps/details?id=com.sitekit.eRedBook&hl=en_GB

OTHER SOLUTION IDEAS TO SUPPORT PLAY AND ACTIVITY:

1. Develop activity and play schedules for you and your infant with guidance from our local children centre or health visitor and make time for these activities.

2. Download the 'born to move' app for access to information and guidance on infant activity and play.

3. Designate a play area (even if this needs to be set-up and put away at times of day) for your infant in the home.

4. Arrange play and physical activity sessions together as a group, or with other parents, extended family, carers and infants – this could help with support and confidence in exercising and conducting physical activity in public spaces.

For peer review only

OTHER LOCAL INITIATIVES, CHARITIES AND PROJECTS IN EAST LONDON

1. BREASTFEEDING NETWORK

Find your nearest breast feeding drop-in-group in Tower Hamlets
https://www.breastfeedingnetwork.org.uk

There are groups run at Shadwell Children's Centre, Chrisp Children's Centre, Overland Children's Centre, Collingwood Children's Centre, Wapping Children's Centre and many more. If you need information or support with breastfeeding in the evenings or at weekends please call:

National Breastfeeding Helpline: *0300 1000 212*

BfN Support in Bengali/Sylheti: *0300 456 2421*

Open from *9.30am - 9.30pm 365 days a year*

Newham: https://www.nct.org.uk/local-activities-meet-ups/region-london/newham/breastfeeding-support

Tower Hamlets: https://www.breastfeedingnetwork.org.uk/tower-hamlets/

For peer review only

Waltham Forest offers a team of infant feeding practitioners delivering 1:1 sessions, a helpline and at home visitors to support the breastfeeding family through HENRY.

For information:
Email: wfsupport@henry.org.uk
Telephone: 020 8496 5223

2. DENTAL SERVICES AND RESOURCES

Dentists across Tower Hamlets, Waltham Forest, and Newham offer free dental check-ups, which you can attend as soon as your child's teeth first appear.

To find your nearest dental practice in Tower Hamlets, please call 020 7364 5000.

HENRY has put together some tips for healthy teeth in young children and a list of Waltham Forest dentists currently accepting new patients from birth: https://www.henry.org.uk/sites/www.henry.org.uk/files/inline-files/Healthy%20Teeth%20Leaflet%20%28April%29_0.pdf

The Kent Community Health NHS Foundation Trust has compiled some online oral health promotion resources for parents here: https://www.kentcht.nhs.uk/service/dental-services/oral-health-promotion-resources/

The Kent Community Health NHS Foundation Trust has compiled some online oral health promotion resources for parents here: https://www.kentcht.nhs.uk/service/dental-services/oral-health-promotion-resources/

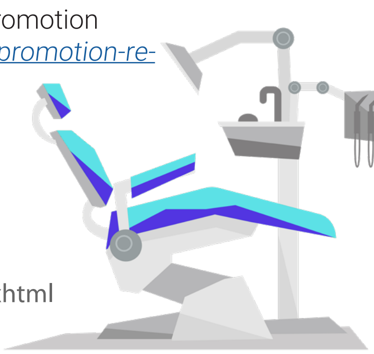

Here are examples of dental services by borough (to find out more, please see the community asset map):

Tower Hamlets

- Fresh Springs Dental Practice
  T: 020 3216 0077
  E: reception@freshspringsdentalpractice.co.uk

- Together Dental Whitechapel
  T: 020 7247 4600
  E: whitechapelreception@together.dental

Waltham Forest

- Abbey Dental Practice
  T: 020 8521 2816
  E: info@abbeydentalwalthamstow.co.uk

- Inspire Dental Walthamstow
  T: 020 8521 6656
  E: info@inspiredentalwalthamstow.co.uk

3. MATERNITY MATES

http://www.whfs.org.uk/index.php/what-we-do/maternity-mates

Maternity Mates™ is a project run by the charity Women's Health and Family Services which recruits, trains and match-ups volunteer Maternity Mates with pregnant women in need of extra support in Tower Hamlets. A Maternity Mate is a female volunteer trained by WHFS to provide practical and emotional support to women during pregnancy, childbirth and the early weeks of motherhood. Support can start any time from the 5th month of pregnancy, through to birth and up to either 6 or 12 weeks after the baby is born (6 weeks in Tower Hamlets and 12 weeks in Newham). **Maternity Mates** are recruited from the communities they serve, and where possible, will speak the same language as the mother-to-be.

For further information, please contact:

Jasmin Begum - Programme Manager
M: *07960 327 760*
T: *020 7377 9640*
E: *jasmin.begum@whfs.org.uk*

For information about the project in Tower Hamlets and Waltham Forest, please contact:

Rubi Rodriguez - Project Co-ordinator Tower Hamlets and Waltham Forest
M: 07496 764 881
E: rubi.rodriguez@whfs.org.uk

For information about the project in Newham, please contact:

Irantzu Perez Arribas - Project Co-ordinator Newham
M: 07436 139 235
E: irantzu.perez@whfs.org.uk

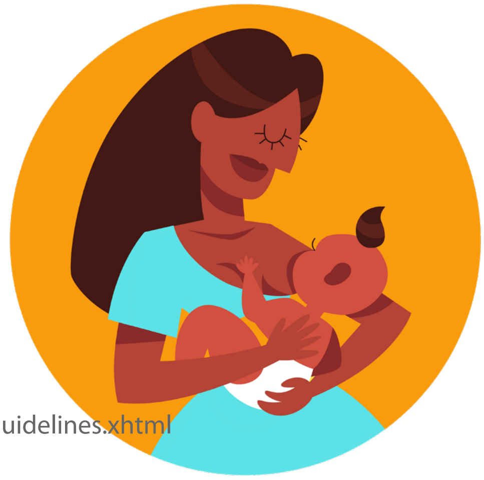

For peer review only

4. BOUNTY

https://www.bounty.com/about-bounty/bounty-packs/newborn-pack

Bounty provides mothers and pregnant women with free subscription packs including advice from Public Health England, as well as a variety of sample sized baby products (nappies, baby formula etc.) and coupons.

Every mother with a Newborn will receive a Newborn Pack at the hospital after delivery, and more freebies and advice are available through the Bounty app.

5. EMMA'S DIARY

https://www.emmasdiary.co.uk
Contact: 01628 535 483 Mon-Fri 9am to 4:30pm

Emma's Diary is a free to access resource created by a board of GPs and midwives that provide information ranging from trying to get pregnant from wellbeing information during and post-pregnancy. There's a free to download mobile app to allow you to access information more easily, tailored towards recent mothers and baby related advice and offers.

6. THE TOYHOUSE CENTRE

http://www.toyhouse.org.uk/

Phone: 0020 7987 7399
E-mail: info@toyhouse.org.uk

Toy House is a local charity in Tower Hamlets which runs projects and services aimed at support play and wellbeing of infants, children and families.

Toy Libraries
http://www.toyhouse.org.uk/toy-libraries/
The charity has its own toy library, and has information and links to over 19 toy libraries and stay and play sessions run across Tower Hamlets.

7.MEND - MIND, EXERCISE, NUTRITION...DO IT!

https://www.mytimeactive.co.uk/mend

MEND is a programme which empowers mums, children and adults to become fitter, healthier and happier and to reach or maintain a healthier weight. It's free to attend and run by local teams in local communities, after school or at weekends. MEND have a range of programmes for different ages run in different boroughs across London.

MEND Mums is designed for post-natal women with a baby up to 2 years and a BMI of 25 or above, **MEND** Mums is a fun and interactive weight management programme incorporating energy boosting workouts and great nutrition tips to help establish healthy habits for life.

For more information about any of the MEND programmes in Tower Hamlets call:
020 8323 1725

For peer review only

8. MIND

MIND is a mental health charity with a specific branch in Newham and Tower Hamlets that offers free mental health services talking therapies, COVID19 Support and Advocacy services

https://www.mithn.org.uk
Phone: 020 7510 1081
Email: info@mithn.org.uk

9. REBECCA CHEETHAM NURSERY & CHILDREN'S CENTRE

Website: https://www.rebeccacheetham.newham.sch.uk
Phone:
Email:

The Rebecca Cheetham Children's Centre offers free child-minding services as well as advice and support for families with young children. However they have temporarily stopped face to face sessions in light of the COVID pandemic. Limited online services are still running for examples: a Family Support Team that can refer you to foodbanks.

OTHER IDEAS AND SUGGESTIONS

- **Breakfast clubs** – these are available in most schools to give children a healthy start to the day. Speak to your local school. Due to COVID19 Restrictions, these breakfast clubs have moved to.
- Despite this, virtual coffee mornings are ongoing

- **Social support** – if you feel you need more support from other mothers, parents or people local to you, you can attend local coffee mornings, toddler groups and parent forums. E.g. One stop shop in Newham and Tower Hamlets. Father examples would be the "idea Store" which is based in Tower Hamlets and offers a range of free activities and educational services for children and families alike. http://www.healthwatchnewham.co.uk/sites/default/files/newham_domestic_abuse_one-stop-shop.pdf

- **Triple P – Positive Parenting Programme** – is an evidence based programme aimed at supporting parents develop relationships with their children.

  - Newham offers a Parenting Befriending service and bookable workshops accessible via the following website: https://families.newham.gov.uk/kb5/newham/directory/family.page?family-channel=1-1

  - Tower Hamlets offers a similar service: https://www.towerhamlets.gov.uk/lgnl/educa tion_and_learning/parental_support/parenting_programmes.aspx
    parenting@towerhamlets.gov.uk.

  - Waltham Forest offers a series of 6 sessions for parents with children aged 3 and over. https://www.walthamforest.gov.uk/content/parenting-support

    Call: *020 8496 2442*
    Email: *earlyhelpparenting@walthamforest.gov.uk*

- **Tunmarsh Centre**– support for parents with children with special needs
  The New Tunmarsh Centre based in Newham provides free education for children with ASD / DHD and support for their families. Parents are regularly invited for coffee mornings with speakers.

  E-mail: Faz.Mac@gmail.com

For peer review only

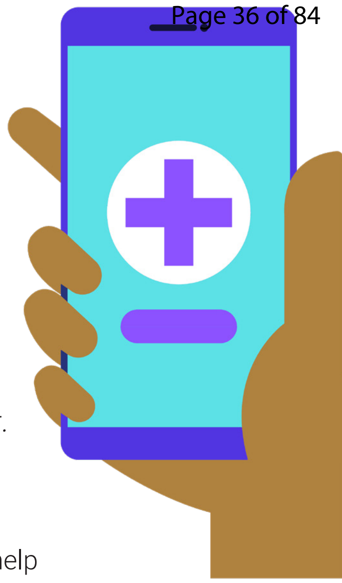

- **John Smith Children's Centre**

  Telephone: 0207 364 0537
  Contact: Deborah Wooding
  Email: deborah.wooding@towerhamlets.gov.uk
  Based in Tower Hamlets, The John Smith Children's Centre that offer childcare support.

- **The Trinity Centre**
  Website: *www.thetrinitycentre.org/home/*
  The Trinity Centre offers a day care nursery as well as independent groups and faith groups to help support parents form minority and marginalised communities

- **Around Poplar Children's Centre**

  Contact: Brenda Pascal
  T: 020 7364 0540
  E: Brenda.pascal@towerhamlets.gov.uk

  The Around Poplar Children's Centre offers childcare, family, health services, and early education support for families with children under 5 free of charge.

  For a list of similar children's centres in Tower Hamlets, see here:
  https://www.localoffertowerhamlets.co.uk/organisations?utf8=%E2%9C%93&search_organisation%5Bterm%5D=&search_organisation%5Blocation%5D=&search_organisation%5Blatitude%5D=&search_organisation%5Blongitude%5D=&search_organisation%5Bcategory_ids%5D%5B%5D=3308

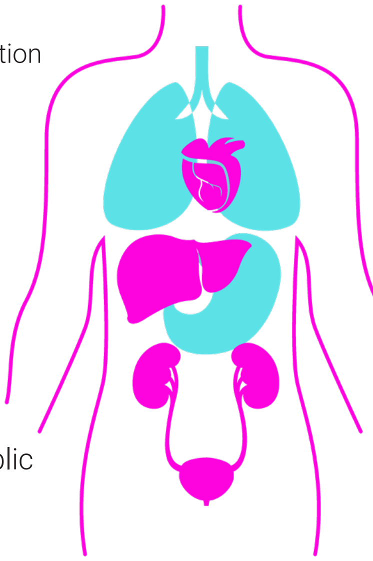

- **Idea Store**

  Website: www.ideastore.co.uk
  Branches of the Idea Store in Tower Hamlets offer library services and access to public computers for families and individuals free of charge.

- **Outreach centre** Outreach centres are open till 6 pm where you can drop your children there, and it is free for people who are entitled for public funds. They offer: Child minding, after school club, breakfast club for parents and children <3 years. Key examples would be Community Centres and Children's Centres.

EXAMPLES:

Newham Council: *https://www.newham.gov.uk/homepage/115/community-centres*
Tower Hamlets: Community and day centres (towerhamlets.gov.uk)

Plaistow Children's Centre
Website: *https://plaistow.newham.sch.uk/Plaistow-Children-s-Centre/*

The Plaistow Children's Centre is based in Newham and offers care and support for young children ranging from 0-5 years for 49 weeks within the year led by staff and volunteers. Other services range from nutrition webinars to overall family support and advice. The Children's Centre is still open during the pandemic and offers slightly modified services as a result.

For peer review only

OTHER SUPPORT

1. Request interpretation support for GP visits from Tower Hamlets GP care group.
2. Advocacy & Interpreting Service in Newham (https://www.gpcaregroup.org/section/455/Services/page/eb745e07-85ab-4cee-bc8f-c880a-d400a95/Advocacy-Interpreting)
3. Provide feedback on services or lodge a complaint via Health Watch Tower Hamlets. More information can be accessed here: https://www.healthwatchtowerhamlets.co.uk/

FOOD BANKS:

Suggested Food Bank locations: https://www.google.com/maps/d/edit?mid=1ZyqIE2-HgdQ-

NEON Community Asset Map: https://www.google.com/maps/d/edit?mid=1ZyqIE2-Hg-dQ-Hzow7KwhAiYsdtQnP_Zq&usp=sharing

- **Newham Food Alliance**

  Website: https://www.newham.gov.uk/newhamfoodalliance

  The Newham Food Alliance is a food bank based in Newham that can offer access to food for those who are struggling financially. There is also delivery support they can provide to those self-isolating due to COVID and providing food remotely during the pandemic regardless.

- **Church Food Banks:**

  Website: www.churchesfoodbank.org.uk/

  Food Banks located in the Borough of Newham. To access these you need to be referred from another agency such as a school, advice centres or your GP.

The 2 main sites are:

Woodgrange Baptist Church

345 Romford Road,Forest Gate,E7 8AA

0208 555 9880

St Paul's Church

65 Maryland Road,Stratford,E15 1JL

0208 534 1164

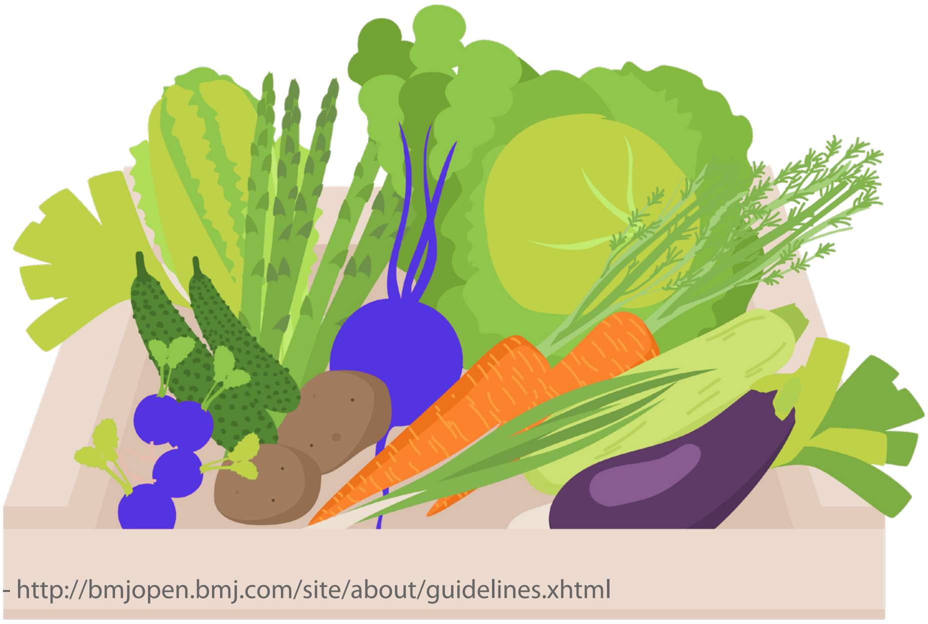

For peer review only

- **The Trussell Trust**

Website: https://www.trusselltrust.org

The Trussell Trust is a charity that provides nationwide network of food banks as well as emergency food for those in need. They also run regular campaigns to further increase support for people who need access to regular foods but cannot afford to do so. The website is useful as it flag foodbanks that are local to you.

- **First Love Foundation Foodbank**

Website: www.firstlovefoundation.org.uk/

The First Love Foundation coordinates the Tower Hamlets Foodbank. It provides daily necessities to families in need and alongside this, operates as a crisis service.

- **Eat or Heat - Waltham Forest Food Bank**

Website: http://eatorheat.org

Phone Number: 0800 772 0212

Email: referrals@eatorheat.org

A charity based in East London that supports local families. On the website, different professionals who can refer you to this service are listed.

- **Rukhsana Khan Foundation**

Website: https://www.rukhsanakhanfoundation.org

Phone Number: 07939 232 123

Email: rukhsanakhanfoundation@outlook.com

Rukshana Khan Foundation is a local charity that supports families in East London. They run food parcels and deliveries for those on benefits and those referred from Citizens Advice, NHS, Local Doctor's Surgery, Job Centre, Baby Food Bank, or the Council. They are also supporting those classed as clinically vulnerable. You can register for support on their website.

They run a foodbank on Saturdays 11am-12pm at the William Morris Community Centre.

- **Waltham Forest Liberal Democrats**

Website: https://www.walthamforestlibdems.org/local_support_for_families_in_need

The Waltham Forest Liberal Democrats have collated a set of resources highlighting local foodbanks with Waltham Forest that local families can access

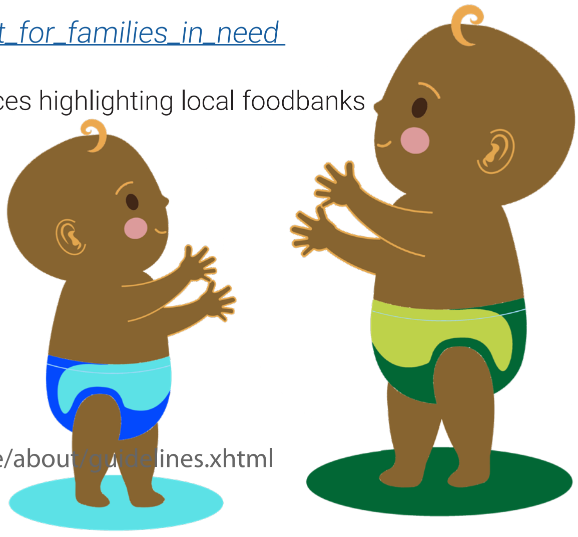

For peer review only

- **Eden Girls' School, Waltham Forest**

  A local food bank based at Eden Girls' School that provides food to local people from 1-3pm every Friday

  Contact Details:

  Eden Girls' School, Waltham Forest

  Silver Birch and Landmark Houses,

  Blackhorse Lane, Walthamstow

  London, E17 5SD

  Tel: 0208 523 1810

  Email: info@egwf.staracademies.org

- **Community Food Projects in Tower Hamlets**

  https://www.wen.org.uk/2020/03/30/https-www-wen-org-uk-2020-03-30-information-on-local-response-to-covid-19/

- **Healthwatch groups available in different boroughs:**

  Newham http://www.healthwatchnewham.co.uk/

  Tower Hamlets https://www.healthwatchtowerhamlets.co.uk/

  Redbridge http://www.healthwatchredbridge.co.uk/

  Waltham Forest https://www.healthwatchwalthamforest.co.uk/

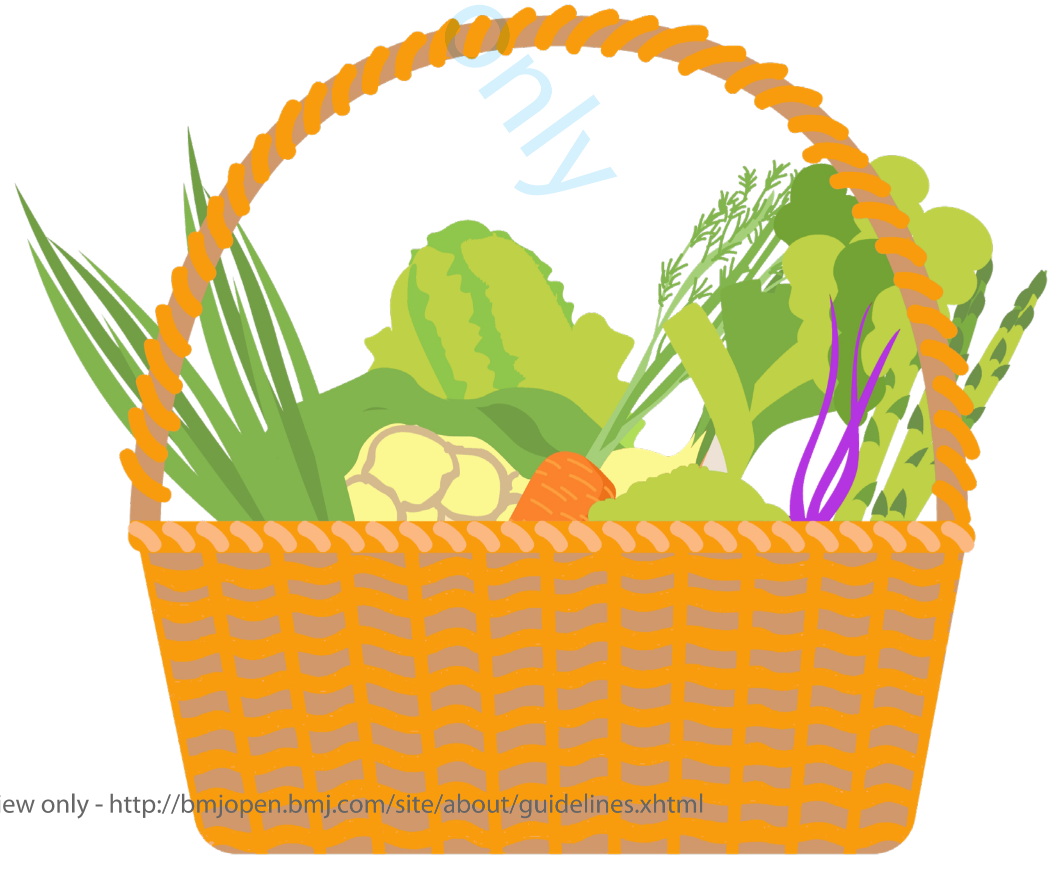

For peer review only

Participants Feedback Questionnaire

1. What were your reasons for wanting to attend the group meetings?

2. What made you come back after attending a meeting? /or, what was your reason for not coming back to later meetings?

i. *What made you keep coming back to the meetings? /Or, what could we have done differently to motivate you to come back to the next meeting?*

3. Did the group manage to meet your expectations?

i. *how did it meet your expectations?*
ii. *If it did not, why was this the case?*

4. What could be improved if this project was run again?

i. *How could we improve the content (meeting exercises)*
ii. *How could we improve the tools (picture cards / infant feeding resources)*
iii. *How could we improve the tailoring to your needs as British Bangladeshi mother/pregnant women/or grandmother*
iv. *Would it be beneficial to have picture cards and other project materials written in Bengali (with the picture on the front and Bengali text on the back)*
v. *Is there any additional information or resources you would have liked to have been offered in the meetings?*

5. How involved did you feel in the learning process of the group?

i. Did you feel you worked together as a group during the meetings? How so?
ii. Were there any barriers you experienced to participating?
iii. Were you given opportunities to share experiences? Please give examples.

6. Are you in contact with any of the other participants outside of the group meetings?

i. Have you discussed any issues raised in the group meetings?
ii. Have you supported each other on any issues raised in the group meeting?

9. Have you found new people, groups or sources of information as a result of attending the group meetings? Please give examples.

10. Did you share any information from the group meetings with any other members of your family or community outside of the group?

11. If we were to implement a full project using this approach, the next stage would involve more group meetings (possibly up to 8 additional meetings) where by the group would support each other to implement some of the solutions you identified and then after implementing

For peer review only - http://bmjopen.bmj.com/site/about/guidelines.xhtml

For peer review only

solutions, evaluate their success. Do you think this is something you or Bangladeshi carers would be willing to undertake or partake in?

i. *Can you think of any barriers to using this approach we need to consider at this stage?*
ii. *How could additional meetings best support you to implement the solutions you identified, and introduce the recommended infant feeding and care practices? What should they focus on?*
iii. *What additional meetings would be acceptable to you and feasible to attend?*

12. Is there anything else you would like to share about your experience on the project?

For peer review only

Facilitator Report Form

<table>
<tr><td colspan="6">PLA GROUP FACILITATOR REPORT - PLA group meeting xx</td></tr>
<tr><td>Group location/name (XX children's centre group)</td><td></td><td rowspan="2">Meeting number</td><td rowspan="2"></td><td rowspan="2">Meeting date</td><td rowspan="2"></td></tr>
<tr><td>Facilitator name</td><td></td></tr>
<tr><td>Number of picture cards discussed</td><td colspan="5"></td></tr>
<tr><td>Did you manage to go through all questions on the picture cards?
• What does the picture show?
• Clarify which practice is being discussed
• Ask and explain how this affects infant growth and development
• Use info on the back of the card to ask questions about the practice and probe
• Ask if this occurs in the community? Is it common?
Were there any barriers to a full discussion on the above questions?</td><td colspan="5"></td></tr>
<tr><td>Were there any picture cards not discussed at all?</td><td colspan="5"></td></tr>
</table>

For peer review only - http://bmjopen.bmj.com/site/about/guidelines.xhtml

Which exercises were completed during your meeting?	
What was the outcome of each exercise?	
How long did the meeting run for?	
Did anything go well/did anything not go well?	
GROUP FACILITATOR TO COMPLETE AFTER EACH MEETING CONDUCTED.	

**PLA GROUP FACILITATOR REPORT – PLA group meeting 3**					
Group location/name (XX children's centre group)		Meeting number		Me etin g dat e	
Facilitator name					
Number and type of picture cards discussed					
Did you manage to go through all the probing questions on the picture cards?<br>• *What does the picture show?*<br>• *Clarify what barrier is being discussed*<br>• *Ask and explain how this barrier may affect or influence behaviour relating to infant feeding and care*					

peer review only

• *Ask which infant feeding and care practice the barrier may prevent parents from practicing?*<br><br>• *Ask if the barrier exists in the community*<br>Were there any barriers to a full discussion on the above questions?	
Were there any picture cards not discussed at all?	
Which exercises were completed during your meeting?	
What was the outcome of each exercise?<br><br>Which barrier cards were identified for prioritised practice 1?<br><br>Which barrier cards were identified for prioritised practice 2?<br><br>Which barrier cards were identified for prioritised practice 3?	
How long did the meeting run for?	
Did anything go well/did anything not go well?	
GROUP FACILITATOR TO COMPLETE AFTER EACH MEETING CONDUCTED.	

For peer review only - http://bmjopen.bmj.com/site/about/guidelines.xhtml

<table>
<tr><td colspan="7">PLA GROUP FACILITATOR REPORT – PLA group meeting 4</td></tr>
<tr><td colspan="2">Group location/name (XX children's centre group)</td><td></td><td rowspan="2">Meeting number</td><td rowspan="2"></td><td rowspan="2">Me etin g dat e</td><td rowspan="2"></td></tr>
<tr><td colspan="2">Facilitator name</td><td></td></tr>
<tr><td colspan="2">Number and type of picture cards discussed</td><td colspan="5"></td></tr>
<tr><td colspan="2">Did you manage to go through all the probing questions on the picture cards?
● What does the picture show?
● Ask participants to identify what solution they are discussing and what non-recommended infant feeding and care practice the solution helps to reduce or manage.
● Ask participants to suggest ideas on what activities the group (or they, as individuals) could undertake to achieve this solution. Use the information on the back of the card to suggest ideas on activities which could be undertaken. Ask if the barrier exists in the community
● Were there any barriers to a full discussion on the above questions?</td><td colspan="5"></td></tr>
<tr><td colspan="2">Were there any picture cards not discussed at all?</td><td colspan="5"></td></tr>
<tr><td colspan="2">Which exercises were completed during your meeting?</td><td colspan="5"></td></tr>
</table>

For peer review only

What was the outcome of each exercise?<br><br>Which barrier solution cards were identified for prioritised practice 1<br><br>Which management solution cards were identified for prioritised practice 1<br><br>Which barrier solution cards were identified for prioritised practice 2<br><br>Which management solution cards were identified for prioritised practice 2<br><br>Which barrier solution cards were identified for prioritised practice 2<br><br>Which management solution cards were identified for prioritised practice 2	
How long did the meeting run for?	
Did anything go well/did anything not go well?	
GROUP FACILITATOR TO COMPLETE AFTER EACH MEETING CONDUCTED.	

For peer review only - http://bmjopen.bmj.com/site/about/guidelines.xhtml

Sustainability assessment

1. PARTICIPATION

Criteria for assessment	*Are many people participating? Are they active? Is anyone excluded from participation?*
Score / Average score assigned	
Identified successes	
Identified concerns / areas for improvement	
Actions agreed	
Additional notes	

2. LEADERSHIP

Criteria for assessment	*Do groups have leaders, are they helping or hindering the group? Do they have the capacity to lead groups well?*
Score / Average score assigned	
Identified successes	
Identified concerns / areas for improvement	
Actions agreed	
Additional notes	

For peer review only - http://bmjopen.bmj.com/site/about/guidelines.xhtml

WOMEN AND CHILDREN FIRST (UK)

3. ORGANISATIONAL STRUCTURE

Criteria for assessment	*Are the organisational structures, including the committee and taskforces, set up in the groups? If so, what is their purpose and how are they functioning?*
Score / Average score assigned	
Identified successes	
Identified concerns / areas for improvement	
Actions agreed	
Additional notes	

4. NEEDS / PROBLEM ASSESSMENT

Criteria for assessment	*Are the problems identified in the groups important and relevant to their respective community?*
Score / Average score assigned	
Identified successes	
Identified concerns / areas for improvement	
Actions agreed	
Additional notes	

WOMEN AND CHILDREN FIRST (UK)

5. RESOURCE MOBILISATION

Criteria for assessment	***Are the groups able to mobilise resources to implement their solutions? Are they able to mobilise resources from within the groups and/or outside the groups?***
Score / Average score assigned	
Identified successes	
Identified concerns / areas for improvement	
Actions agreed	
Additional notes	

6. ABILITY TO ASK "WHY"?

Criteria for assessment	***Do groups try to address root causes of the problems they face? If so, how well? By root causes we are referring to meeting re: contributing factors – the broader causes of ill health such as poverty.***
Score / Average score assigned	
Identified successes	
Identified concerns / areas for improvement	
Actions agreed	
Additional notes	

7. LINKS TO OTHER ORGANISATIONS / PEOPLE

Criteria for assessment	*Are they linking to other stakeholders (people, groups, organisations) who can help them solve identified issues? If so, who are they linking with and why?*
Score / Average score assigned	
Identified successes	
Identified concerns / areas for improvement	
Actions agreed	
Additional notes	

8. RELATIONSHIP WITH OUTSIDE AGENT

Criteria for assessment	*Is the external agent – Ministry of Health counterparts – involved and supporting the groups, building their capacity?*
Score / Average score assigned	
Identified successes	
Identified concerns / areas for improvement	
Actions agreed	
Additional notes	

9. PROGRAMME MANAGEMENT

**Criteria for assessment**	***Do groups feel they own the groups and their work? Are they capable of continuing the activities once the project comes to an end? Is the NGO implementing partner building group capacity to solve health problems and strengthening them to be sustainable?***
**Score / Average score assigned**	
**Identified successes**	
**Identified concerns / areas for improvement**	
**Actions agreed**	
**Additional notes**	

For peer review only - http://bmjopen.bmj.com/site/about/guidelines.xhtml

Direct observation of intervention delivery and CF performance Form:

Did the facilitator deliver the components of the sessions according to the manual?
(Please rate this on scale of 1-4 scale i.e. 1 = no elements of the programme delivered;
2 = some elements of the programme, others missed or inserted;
3 = majority elements of the programme delivered;
4 = all elements of the programme delivered as per handbook).

What are your thoughts about the overall session?

Did you think there are areas for improvement and what where they?

Would you have done anything differently while facilitating the sessions and how?

What advice do you have for the community facilitator?

For peer review only

For peer review only - http://bmjopen.bmj.com/site/about/guidelines.xhtml

Development performance of children – (ASQ-3)

Improving lives

NHS
Oxleas
NHS Foundation Trust

Parents of Shiv LOGAN
3 Abbey Terrace
Abbey Wood
London
SE2 9EY

380 D

Oxleas 0 – 19 Years Public Health Nursing Service
Central Administration Team
Tel: 020 8836 8621
Email: oxl-tr.cat@nhs.net

11 Nov 2019

Dear Parents of Shiv,

9 – 12 month review Appointment confirmation

This letter is to confirm your appointment to review your child's development.

At: Gallions Reach Health Centre Bentham Road Thamesmead London SE28 8BE

On: Tuesday 10 Dec 2019 10:30

Enclosed with this letter are the 'Ages and Stages' questionnaires that are used for this developmental review. Please complete these forms and try out the activities with your baby before the appointment.

It is important that you complete the questionnaires with your child before your appointment and bring the completed questionnaires and Red Book with you on the day.

If you require any help completing this questionnaire or you have any queries about your appointment please contact us on **020 8836 8621**

To find out about Healthy Start benefits search online for the Healthy Start website

Visit our website which was designed by Oxleas Health Care professionals, service users and families to provide you with information about your health and wellbeing and how to access our services.

www.Oxleas.nhs.uk/CYP

Follow us on Twitter @oxleasCYPGre

There is a drop in Health Advice Session open every day from Monday to Friday across Greenwich. For times and venues see Health Visitor Timetable in the link above.

Yours sincerely
Public Health Nursing Service 0 -19 Years

For peer review only - http://bmjopen.bmj.com/site/about/guidelines.xhtml

ASQ-3 **10 Month Questionnaire** *page 3 of 6*

GROSS MOTOR *(continued)*

	YES	SOMETIMES	NOT YET	
3. When you stand your baby next to furniture or the cot rail, does she hold on without leaning her chest against the furniture for support? 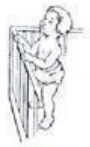	○	○	○	___
4. While holding onto furniture, does your baby bend down and pick up a toy from the floor and then return to a standing position? 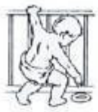	○	○	○	___
5. While holding onto furniture, does your baby lower himself with control (without falling or flopping down)?	○	○	○	___
6. Does your baby walk beside furniture while holding on with only one hand?	○	○	○	___
		GROSS MOTOR TOTAL		___

FINE MOTOR

	YES	SOMETIMES	NOT YET	
1. Does your baby pick up a small toy with only one hand? 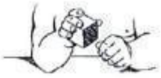	○	○	○	___
2. Does your baby *successfully* pick up a small cube of bread by using her thumb and all of her fingers in a raking motion? (*If she already picks up a small cube of bread, mark "yes" for this item.*) 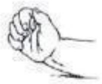	○	○	○	___
3. Does your baby pick up a small toy with the *tips* of his thumb and fingers? (*You should see a space between the toy and his palm.*) 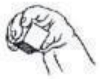	○	○	○	___
4. After one or two tries, does your baby pick up a piece of string with her first finger and thumb? (*The string may be attached to a toy.*) 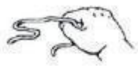	○	○	○	___
5. Does your baby pick up a small cube of bread with the tips of his thumb and a finger? (*He may rest his arm or hand on the table while doing it.*) 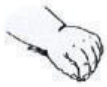	○	○	○	___ *
6. Does your baby put a small toy down, without dropping it, and then take her hand off the toy?	○	○	○	___
		FINE MOTOR TOTAL		___

* If Fine Motor Item 5 is marked "yes" or "sometimes," mark Fine Motor Item 2 "yes."

BE Av. 1 1.15

Ages & Stages Questionnaires®, Third Edition (ASQ-3™), Squires & Bricker
© 2009-2015 Paul H. Brookes Publishing Co. All rights reserved.
Adaptation into British English prepared with the Department of Health.

For peer review only - http://bmjopen.bmj.com/site/about/guidelines.xhtml

ASQ-3 **10 Month Questionnaire** *page 5 of 6*

OVERALL

Parents and providers may use the space below for additional comments.

1. Does your baby use both hands and both legs equally well? If no, explain: ○ YES ○ NO

2. When you help your baby stand, are his feet flat on the surface most of the time? If no, explain: ○ YES ○ NO

3. Do you have concerns that your baby is too quiet or does not make sounds like other babies? If yes, explain: ○ YES ○ NO

4. Does either parent have a family history of childhood deafness or hearing problems? If yes, explain: ○ YES ○ NO

5. Do you have concerns about your baby's eyesight? If yes, explain: ○ YES ○ NO

6. Has your baby had any medical or health-related problems in the last few months? If yes, explain: ○ YES ○ NO

BE Av. 1 1.15

Ages & Stages Questionnaires®, Third Edition (ASQ-3™), Squires & Bricker
© 2009-2015 Paul H. Brookes Publishing Co. All rights reserved.
Adaptation into British English prepared with the Department of Health.

For peer review only - http://bmjopen.bmj.com/site/about/guidelines.xhtml

4-day Food Diary

DAY 1

Day 1:		Date:		
Time	Where?<br>With Whom?<br>TV on?<br>At table?	Food/Drink description & preparation	Brand Name	Portion size or quantity eaten
How to describe what you had and how much you had can be found on pages 16 - 21				
6am to 9am				
9am to 12 noon				

Time	Where?<br>With Whom?<br>TV on?<br>At table?	Food/Drink description & preparation	Brand Name	Portion size or quantity eaten
12 noon to 2pm				
2pm to 5pm				

Time	Where? With Whom? TV on? At table?	Food/Drink description & preparation	Brand Name	Portion size or quantity eaten
		5pm to 8pm		
		8pm to 10pm		
		10pm to 6am		

Was the amount of **food** that you had today about what you usually have, less than usual, or more than usual?

Yes, usual ☐ No, **less** than usual ☐ No, **more** than usual ☐

Please tell us why you had less than usual

Please tell us why you had more than usual

Was the amount you had to **drink** today, including water, tea, coffee and soft drinks [and alcohol], about what you usually have, less than usual, or more than usual?

Yes, usual ☐ No, **less** than usual ☐ No, **more** than usual ☐

Please tell us why you had less than usual

Please tell us why you had more than usual

Did you **finish all the food and drink** that you recorded in the diary today?

Yes ☐ No ☐

If no, please **go back to the diary and make a note of any leftovers**

Did you take any **vitamins, minerals or other food supplements** today?

Yes ☐ No ☐

If yes, **please describe the supplements you took below**

Brand	Name (in full) including strength	Number of pills, capsules, teaspoons

Please record on the next pages details of any recipes or (if not already described) ingredients of made up dishes or take-away dishes.

**Write in recipes or ingredients of made up dishes or take-away dishes**			
NAME OF DISH:		***Serves:***	
Ingredients	***Amount***	***Ingredients***	***Amount***
Brief description of cooking method			

Write in recipes or ingredients of made up dishes or take-away dishes			
NAME OF DISH:		***Serves:***	
Ingredients	***Amount***	***Ingredients***	***Amount***
Brief description of cooking method			

DAY 2

Day 2:		Date:		
Time	Where? With Whom? TV on? At table?	Food/Drink description & preparation	Brand Name	Portion size or quantity eaten
How to describe what you had and how much you had can be found on pages 16 - 21				
6am to 9am				
9am to 12 noon				

For peer review only - http://bmjopen.bmj.com/site/about/guidelines.xhtml

Time	Where?<br>With Whom?<br>TV on?<br>At table?	Food/Drink description & preparation	Brand Name	Portion size or quantity eaten
		12 noon to 2pm		
		2pm to 5pm		

Time	Where?<br>With Whom?<br>TV on?<br>At table?	Food/Drink description & preparation	Brand Name	Portion size or quantity eaten
		5pm to 8pm		
		8pm to 10pm		
		10pm to 6am		

Was the amount of **food** that you had today about what you usually have, less than usual, or more than usual?

Yes, usual ☐ No, **less** than usual ☐ No, **more** than usual ☐

Please tell us why you had less than usual

Please tell us why you had more than usual

Was the amount you had to **drink** today, including water, tea, coffee and soft drinks [and alcohol], about what you usually have, less than usual, or more than usual?

Yes, usual ☐ No, **less** than usual ☐ No, **more** than usual ☐

Please tell us why you had less than usual

Please tell us why you had more than usual

Did you **finish all the food and drink** that you recorded in the diary today?

Yes ☐ No ☐

If no, please **go back to the diary and make a note of any leftovers**

Did you take any **vitamins, minerals or other food supplements** today?

Yes ☐ No ☐

If yes, **please describe the supplements you took below**

Brand	Name (in full) including strength	Number of pills, capsules, teaspoons

Please record on the next pages details of any recipes or (if not already described) ingredients of made up dishes or take-away dishes.

Write in recipes or ingredients of made up dishes or take-away dishes			
NAME OF DISH:		***Serves:***	
Ingredients	***Amount***	***Ingredients***	***Amount***
Brief description of cooking method			

Write in recipes or ingredients of made up dishes or take-away dishes			
NAME OF DISH:		***Serves:***	
Ingredients	***Amount***	***Ingredients***	***Amount***
Brief description of cooking method			

DAY 3

Day 3:		Date:		
Time	Where?<br>With Whom?<br>TV on?<br>At table?	Food/Drink description & preparation	Brand Name	Portion size or quantity eaten
How to describe what you had and how much you had can be found on pages 16 - 21				
6am to 9am				
9am to 12 noon				

Time	Where?<br>With Whom?<br>TV on?<br>At table?	Food/Drink description & preparation	Brand Name	Portion size or quantity eaten
12 noon to 2pm				
2pm to 5pm				

Time	Where? With Whom? TV on? At table?	Food/Drink description & preparation	Brand Name	Portion size or quantity eaten
		5pm to 8pm		
		8pm to 10pm		
		10pm to 6am		

Was the amount of **food** that you had today about what you usually have, less than usual, or more than usual?

Yes, usual ☐ No, **less** than usual ☐ No, **more** than usual ☐

Please tell us why you had less than usual

Please tell us why you had more than usual

Was the amount you had to **drink** today, including water, tea, coffee and soft drinks [and alcohol], about what you usually have, less than usual, or more than usual?

Yes, usual ☐ No, **less** than usual ☐ No, **more** than usual ☐

Please tell us why you had less than usual

Please tell us why you had more than usual

Did you **finish all the food and drink** that you recorded in the diary today?

Yes ☐ No ☐

If no, please **go back to the diary and make a note of any leftovers**

Did you take any **vitamins, minerals or other food supplements** today?

Yes ☐ No ☐

If yes, **please describe the supplements you took below**

Brand	Name (in full) including strength	Number of pills, capsules, teaspoons

Please record on the next pages details of any recipes or (if not already described) ingredients of made up dishes or take-away dishes.

Write in recipes or ingredients of made up dishes or take-away dishes			
NAME OF DISH:		***Serves:***	
Ingredients	***Amount***	***Ingredients***	***Amount***
Brief description of cooking method			

For peer review only - http://bmjopen.bmj.com/site/about/guidelines.xhtml

Write in recipes or ingredients of made up dishes or take-away dishes			
NAME OF DISH:		***Serves:***	
Ingredients	***Amount***	***Ingredients***	***Amount***
Brief description of cooking method			

DAY 4

Please remember to complete the general questions on pages 61-66!

Day 4:		Date:		
Time	Where? With Whom? TV on? At table?	Food/Drink description & preparation	Brand Name	Portion size or quantity eaten
How to describe what you had and how much you had can be found on pages 16 - 21				
6am to 9am				
9am to 12 noon				

Time	Where?<br>With Whom?<br>TV on?<br>At table?	Food/Drink description & preparation	Brand Name	Portion size or quantity eaten
		12 noon to 2pm		
		2pm to 5pm		

Time	Where?<br>With Whom?<br>TV on?<br>At table?	Food/Drink description & preparation	Brand Name	Portion size or quantity eaten
		5pm to 8pm		
		8pm to 10pm		
		10pm to 6am		

Was the amount of **food** that you had today about what you usually have, less than usual, or more than usual?

Yes, usual ☐ No, **less** than usual ☐ No, **more** than usual ☐

Please tell us why you had less than usual

Please tell us why you had more than usual

Was the amount you had to **drink** today, including water, tea, coffee and soft drinks [and alcohol], about what you usually have, less than usual, or more than usual?

Yes, usual ☐ No, **less** than usual ☐ No, **more** than usual ☐

Please tell us why you had less than usual

Please tell us why you had more than usual

Did you **finish all the food and drink** that you recorded in the diary today?

Yes ☐ No ☐

If no, please **go back to the diary and make a note of any leftovers**

Did you take any **vitamins, minerals or other food supplements** today?

Yes ☐ No ☐

If yes, **please describe the supplements you took below**

Brand	Name (in full) including strength	Number of pills, capsules, teaspoons

Please record on the next pages details of any recipes or (if not already described) ingredients of made up dishes or take-away dishes.

Write in recipes or ingredients of made up dishes or take-away dishes			
NAME OF DISH:		***Serves:***	
Ingredients	***Amount***	***Ingredients***	***Amount***
Brief description of cooking method			

Write in recipes or ingredients of made up dishes or take-away dishes			
NAME OF DISH:		***Serves:***	
Ingredients	***Amount***	***Ingredients***	***Amount***
Brief description of cooking method			

For peer review only - http://bmjopen.bmj.com/site/about/guidelines.xhtml

General questions about your food/ drink during the recording period.

Special diet

1. Did you follow a special diet during the recording period e.g. vegetarian, cholesterol lowering, weight reducing?

Yes ☐ *Please specify* ☐ No ☐

Milk

2. Which type of milk did you use most often during the recording period?

Whole, fresh, pasteurised ☐ Semi-skimmed fresh, pasteurised ☐ Skimmed (fat free) fresh, pasteurised ☐ 1% fat milk, pasteurised ☐

Dried ☐ *Type* ☐ Soya ☐ *Type* ☐

Other ☐ *Type* ☐ Did not use ☐

Tea and coffee

3. How much milk did you usually have in coffee/ tea?

Coffee	A lot ☐	Some ☐	A little ☐	None/did not drink ☐
Tea	A lot ☐	Some ☐	A little ☐	None/did not drink ☐

4. Did you usually sweeten your coffee/ tea with sugar?

Coffee	Yes ☐	How many teaspoons in a mug/cup? ☐	No/did not drink ☐
Tea	Yes ☐	How many teaspoons in a mug/cup? ☐	No/did not drink ☐

5. Did you usually sweeten your coffee/ tea with artificial sweetener?

Coffee	Yes ☐	How many tablets or teaspoons in a mug/cup? ☐	No/did not drink ☐
Tea	Yes ☐	How many tablets or teaspoons in a mug/cup? ☐	No/did not drink ☐

6. Did you drink decaffeinated coffee/ tea during the recording period?

Coffee	Always ☐	Sometimes ☐	Never ☐
Tea	Always ☐	Sometimes ☐	Never ☐

For peer review only - http://bmjopen.bmj.com/site/about/guidelines.xhtml

Breakfast cereals

7. How much milk did you usually have on breakfast cereal?

Drowned ☐ Average ☐ Damp ☐ None/did not eat ☐

8. How did you usually make your porridge?

With all water ☐ With all milk ☐ With milk and water ☐ Did not eat ☐

9. Did you usually sweeten or salt your porridge?

With sugar ☐ With honey ☐ With salt ☐ Neither/did not eat ☐

10. How did you usually make your instant oat cereal?

With all water ☐ With all milk ☐ With milk and water ☐ Did not eat ☐

11. Did you usually sweeten or salt your instant oat cereal?

With sugar ☐ With honey ☐ With salt ☐ Neither/did not eat ☐

Fats for spreading and cooking

12. Which type of butter, margarine or other fat spread did you use most often during the recording period? Please record the full product name and fat content

Name: ☐ None ☐

e.g. Flora Omega 3 plus, low fat spread, 38% fat, polyunsaturated

13. How thickly did you spread butter, margarine on bread, crackers etc?

Thick ☐ Medium ☐ Thin ☐ N/A ☐

14. Which type of cooking fat/oil did your household use most often over the recording period? Please record the full product name e.g. *Sainsbury's sunflower oil*

Name: ☐ None ☐

Bread

15. Which type of bread did you eat most often during the recording period?

White ☐ Granary ☐ Wholemeal ☐ Brown ☐

50/50 bread e.g. Hovis Best of Both ☐ Other ☐ *Type* ☐ Did not eat ☐

16. Was it a large loaf or a small loaf?

Large ☐ Small ☐

For peer review only - http://bmjopen.bmj.com/site/about/guidelines.xhtml

17. If the bread was shop bought, how was it sliced?

Thick ☐ Medium ☐ Thin ☐ Unsliced ☐ N/A ☐

Meat

18. If you ate meat during the recording period, did you eat the visible fat?

Always ☐ Sometimes ☐ Never ☐ Did not eat meat ☐

19. If you ate poultry (e.g. chicken, turkey) during the recording period, did you eat the skin?

Always ☐ Sometimes ☐ Never ☐ Did not eat poultry ☐

Fruit and vegetables

20. If you ate apples during the recording period, did you eat the skin?

Always ☐ Sometimes ☐ Never ☐ Did not eat ☐

21. If you ate pears during the recording period, did you eat the skin?

Always ☐ Sometimes ☐ Never ☐ Did not eat ☐

22. If you ate new potatoes during the recording period, did you eat the skin?

Always ☐ Sometimes ☐ Never ☐ Did not eat ☐

23. If you ate baked/jacket potatoes during the recording period, did you eat the skin?

Always ☐ Sometimes ☐ Never ☐ Did not eat ☐

Salt

24. Do you add salt to your food at the table?

Always ☐ Sometimes ☐ Never ☐

25. Do you add salt substitute to your food at the table? *e.g. LoSalt*

Always ☐ Sometimes ☐ Never ☐

Water

26. Which type of water did you drink most often during the recording period?

Tap ☐ Filtered ☐ Bottled ☐ *brand* ______ Did not drink ☐

Thank you for completing this diary.

For peer review only - http://bmjopen.bmj.com/site/about/guidelines.xhtml

Children Feeding Behaviour

ID:

Child Eating Behaviour Questionnaire (CEBQ)

Please read the following statements and tick the boxes most appropriate to your child's eating behaviour.

	Never	Rarely	Some-times	Often	Always
My child loves food	☐	☐	☐	☐	☐
My child eats more when worried	☐	☐	☐	☐	☐
My child has a big appetite	☐	☐	☐	☐	☐
My child finishes his/her meal quickly	☐	☐	☐	☐	☐
My child is interested in food	☐	☐	☐	☐	☐
My child is always asking for a drink	☐	☐	☐	☐	☐
My child refuses new foods at first	☐	☐	☐	☐	☐
My child eats slowly	☐	☐	☐	☐	☐
My child eats less when angry	☐	☐	☐	☐	☐
My child enjoys tasting new foods	☐	☐	☐	☐	☐
My child eats less when s/he is tired	☐	☐	☐	☐	☐
My child is always asking for food	☐	☐	☐	☐	☐
My child eats more when annoyed	☐	☐	☐	☐	☐
If allowed to, my child would eat too much	☐	☐	☐	☐	☐
My child eats more when anxious	☐	☐	☐	☐	☐
My child enjoys a wide variety of foods	☐	☐	☐	☐	☐
My child leaves food on his/her plate at the end of a meal	☐	☐	☐	☐	☐
My child takes more than 30 minutes to finish a meal	☐	☐	☐	☐	☐

	Never	Rarely	Some-times	Often	Always
Given the choice, my child would eat most of the time	☐	☐	☐	☐	☐
My child looks forward to mealtimes	☐	☐	☐	☐	☐
My child gets full before his/her meal is finished	☐	☐	☐	☐	☐
My child enjoys eating	☐	☐	☐	☐	☐
My child eats more when she is happy	☐	☐	☐	☐	☐
My child is difficult to please with meals	☐	☐	☐	☐	☐
My child eats less when upset	☐	☐	☐	☐	☐
My child gets full up easily	☐	☐	☐	☐	☐
My child eats more when s/he has nothing else to do	☐	☐	☐	☐	☐
Even if my child is full up s/he finds room to eat his/her favourite food	☐	☐	☐	☐	☐
If given the chance, my child would drink continuously throughout the day	☐	☐	☐	☐	☐
My child cannot eat a meal if s/he has had a snack just before	☐	☐	☐	☐	☐
If given the chance, my child would always be having a drink	☐	☐	☐	☐	☐
My child is interested in tasting food s/he hasn't tasted before	☐	☐	☐	☐	☐
My child decides that s/he doesn't like a food, even without tasting it	☐	☐	☐	☐	☐
If given the chance, my child would always have food in his/her mouth	☐	☐	☐	☐	☐
My child eats more and more slowly during the course of a meal	☐	☐	☐	☐	☐

For peer review only - http://bmjopen.bmj.com/site/about/guidelines.xhtml

Parental Feeding Style

Parental Feeding Style Questionnaire

Please read the following statements and tick the appropriate boxes to show how you deal with feeding your child. It is important to remember that there are no right or wrong answers to these questions, we are interested in what parents really feel and do.

ID

	Never	Rarely	Some-times	Often	Always
I allow my child to choose which foods to have for meals	☐	☐	☐	☐	☐
I give my child something to eat to make him/her feel better when s/he is feeling upset	☐	☐	☐	☐	☐
I encourage my child to look forward to the meal	☐	☐	☐	☐	☐
I praise my child if s/he eats what I give him/her	☐	☐	☐	☐	☐
I decide how many snacks my child should have	☐	☐	☐	☐	☐
I encourage my child to eat a wide variety of foods	☐	☐	☐	☐	☐
In order to get my child to behave him/herself I promise him/her something to eat	☐	☐	☐	☐	☐
I present food in an attractive way to my child	☐	☐	☐	☐	☐
If my child misbehaves I withhold his/her favourite food	☐	☐	☐	☐	☐
I encourage my child to taste each of the foods I serve at mealtimes	☐	☐	☐	☐	☐
I allow my child to wander around during a meal	☐	☐	☐	☐	☐
I encourage my child to try foods that s/he hasn't tasted before	☐	☐	☐	☐	☐
I give my child something to eat to make him/her feel better when s/he has been hurt	☐	☐	☐	☐	☐
I let my child decide when s/he would like to have her meal	☐	☐	☐	☐	☐
I give my child something to eat if s/he is feeling bored	☐	☐	☐	☐	☐
I allow my child to decide when s/he has had enough snacks to eat	☐	☐	☐	☐	☐
I decide when it is time for my child to have a snack	☐	☐	☐	☐	☐
I use puddings as a bribe to get my child to eat his/her main course	☐	☐	☐	☐	☐
I encourage my child to enjoy his/her food	☐	☐	☐	☐	☐

PTO

For peer review only - http://bmjopen.bmj.com/site/about/guidelines.xhtml

Network Diffusion Form

(To be completed by participants at the end of each PLA session and at 6 months from baseline)

1. How many people did you share your material/information from the PLA sessions with?..

2. How did you share this information/material (verbal, social media, whatsapp etc)?..

3. Please fill the table below with details of the people you shared the information with -

Serial number	Relation to you	Age/Gender	Family size	How did you share (verbal, social media, whatsapp)

For peer review only - http://bmjopen.bmj.com/site/about/guidelines.xhtml

Equality Impact Assessment

Equality Impact Assessment- NEON

**Question**	**Response**
**1. Name of intervention being assessed**	NEON Intervention (PLA Cycle)
**2. Summary of aims and objectives of the intervention**	To improve infant feeding, care and dental hygiene practices amongst the South Asian Communities of Tower Hamlets, Newham, and Waltham Forest
**3. What involvement and consultation has been done in relation to this intervention?** *(e.g., with relevant groups and stakeholders)*	
**4. Who is affected by the intervention?**	Infants (aged<24 months)<br>Mothers<br>Pregnant women<br>Carers- Grandmother etc.
**5. What are the arrangements for monitoring and reviewing the actual impact of the intervention?**	

**Protected Characteristic Group**	**Is there a potential for positive or negative impact?**	**Please explain and give examples of any evidence/data used**	**Action to address negative impact (e.g. adjustment to the intervention)**
**Disability**			
**Gender reassignment**			
**Marriage or civil partnership**			
**Pregnancy and maternity**			
**Race**			
**Religion or belief**			
**Sexual orientation**			
**Sex (gender)**			
**Age**			

For peer review only - http://bmjopen.bmj.com/site/about/guidelines.xhtml

Evaluation:

Question	Explanation / justification
Is it possible the proposed intervention could discriminate or unfairly disadvantage people?	

Final Decision:	Tick the relevant box	Include any explanation / justification required
1. No barriers identified, therefore activity will **proceed**.		
2. You can decide to **stop** the intervention at some point because the data shows bias towards one or more groups		
3. You can **adapt or change** the intervention in a way which you think will eliminate the bias		
4. Barriers and impact identified, however having considered all available options carefully, there appear to be no other proportionate ways to achieve the aim of the intervention (e.g., in extreme cases or where positive action is taken). Therefore, you are going to **proceed with caution** with this intervention knowing that it may favour some people less than others, providing justification for this decision.		

**Date completed:**	
**Completed by whom (Borough/individual):**	

Staff time questionnaire

Measurement of Time Use for staff involved in *NEON*

This questionnaire is for collecting information on how staff from the study team who are contributing to the Nurture Early for Optimal Nutrition (*NEON*) divide their time between the different trial components. We aim to collect this information from all our partners staff involved in *NEON* over the duration of the 3-year of the study.

The information you provide will only be used for research purposes only. It ***will not*** be used to monitor performance or to plan future activities. The information you provide will help us to divide the total *NEON* cost into the following components: intervention, M&E, process evaluation, and research. This will allow us to report the cost effectiveness of the *NEON* intervention. We also want to separate out the setting up cost from the implementation cost, to inform others who may wish to implement these interventions.

Your name will not be used in the analysis but you will be identifiable through your answers. If you do not want to proceed, or wish to stop at any time, you can do so. If you have any questions, please discuss with Shereen Al Laham (*s.laham@ucl.ac.uk*).

==

Name of Staff Member: ....

Job title / role: ....

Institution: ....

Date: ....

---

1. When did your involvement with *NEON* first begin? .... (Month/Year)
2. Since you started, have you been involved in any project other than *NEON*?

   ☐ Yes

   ☐ No. **Go to question 6.**

3. If Yes, how many days did you spend on *NEON* in the last 30 days?

   .... Days OR .... Percent of time

4. Was this a typical month for you, since you started work on *NEON*?

   ☐ Yes. **Go to question 6.**

   ☐ No

5. If No, please tell me how you have divided your time between *NEON* and any other project since you first started on *NEON*.

   ....

   ....

   (E.g: On average, 2 days per week from January to May, then full-time since June. Answer can be in percentage, days, or weeks – whatever you think most accurately represents your actual time use.)

6. Thinking now about the **last 30 days**, and only those days you worked on *NEON*, how have you in practice divided your time between the following *NEON* activities?

a) I spent .... days OR .... percent time on Monitoring and Evaluation.
b) I spent .... days OR .... percent time on study delivery and implementation.
c) I spent .... days OR .... percent time on Research Work, that is, working on the study design, reading the literature, or writing up or presenting the research findings.
d) I spent .... days OR .... percent time on Joint Work, that is, it is not possible to say it was one of the above. For example, general management or support functions.

7. Is this how you usually divide your time?

☐ Yes. **Go to question 9.**

☐ No

8. If No, please tell me how you have divided your time between the different *NEON* activities since you first started on *NEON*. *(Use the categories in question 6.)*

....

....

....

(E.g: 50% Joint work for the first 6 months, then 1 day per week Joint work but otherwise only NEON. Answer can be in percentage, days, or weeks – whatever you think most accurately represents your actual time use.)

9. Finally, I want to ask you about the division of time between **setting up** and **implementation** of the *NEON* study. Examples of setting up activities are: recruiting and training; developing and testing the intervention material. These activities may still be ongoing.
Please could you tell us when you start to work on implementation, and do setting up activities still continue

	**Activity**	**Start date of implementation** *(Month/Year)*	**Time spend on SETTING UP activities since start date** *(days per week or percentage of time)*
a	NEON intervention implementation		
b	Monitoring and Evaluation implementation		
c	Process Evaluation implementation		

== **Thank You** ==

For peer review only - http://bmjopen.bmj.com/site/about/guidelines.xhtml

SPIRIT

STANDARD PROTOCOL ITEMS: RECOMMENDATIONS FOR INTERVENTIONAL TRIALS

SPIRIT 2013 Checklist: Recommended items to address in a clinical trial protocol and related documents*

Section/ item	Item No	Description	Page
**Administrative information**			
Title	1	Descriptive title identifying the study design, population, interventions, and, if applicable, trial acronym	1
Trial registration	2a	Trial identifier and registry name. If not yet registered, name of intended registry	3, 17, 18
	2b	All items from the World Health Organization Trial Registration Data Set	N/A
Protocol version	3	Date and version identifier	17, 18
Funding	4	Sources and types of financial, material, and other support	17
Roles and responsibilities	5a	Names, affiliations, and roles of protocol contributors	1, 17
	5b	Name and contact information for the trial sponsor	17
	5c	Role of study sponsor and funders, if any, in study design; collection, management, analysis, and interpretation of data; writing of the report; and the decision to submit the report for publication, including whether they will have ultimate authority over any of these activities	17, 18
	5d	Composition, roles, and responsibilities of the coordinating centre, steering committee, endpoint adjudication committee, data management team, and other individuals or groups overseeing the trial, if applicable (see Item 21a for data monitoring committee)	11, 16
**Introduction**			
Background and rationale	6a	Description of research question and justification for undertaking the trial, including summary of relevant studies (published and unpublished) examining benefits and harms for each intervention	3, 5
	6b	Explanation for choice of comparators	6, 7
Objectives	7	Specific objectives or hypotheses	6
Trial design	8	Description of trial design including type of trial (eg, parallel group, crossover, factorial, single group), allocation ratio, and framework (eg, superiority, equivalence, noninferiority, exploratory)	6, 7

For peer review only

Methods: Participants, interventions, and outcomes

Study setting	9	Description of study settings (eg, community clinic, academic hospital) and list of countries where data will be collected. Reference to where list of study sites can be obtained	7
Eligibility criteria	10	Inclusion and exclusion criteria for participants. If applicable, eligibility criteria for study centres and individuals who will perform the interventions (eg, surgeons, psychotherapists)	7, 8
Interventions	11a	Interventions for each group with sufficient detail to allow replication, including how and when they will be administered	6, 7, 10
	11b	Criteria for discontinuing or modifying allocated interventions for a given trial participant (eg, drug dose change in response to harms, participant request, or improving/worsening disease)	N/A
	11c	Strategies to improve adherence to intervention protocols, and any procedures for monitoring adherence (eg, drug tablet return, laboratory tests)	N/A
	11d	Relevant concomitant care and interventions that are permitted or prohibited during the trial	N/A
Outcomes	12	Primary, secondary, and other outcomes, including the specific measurement variable (eg, systolic blood pressure), analysis metric (eg, change from baseline, final value, time to event), method of aggregation (eg, median, proportion), and time point for each outcome. Explanation of the clinical relevance of chosen efficacy and harm outcomes is strongly recommended	11, 12
Participant timeline	13	Time schedule of enrolment, interventions (including any run-ins and washouts), assessments, and visits for participants. A schematic diagram is highly recommended (see Figure)	9
Sample size	14	Estimated number of participants needed to achieve study objectives and how it was determined, including clinical and statistical assumptions supporting any sample size calculations	8
Recruitment	15	Strategies for achieving adequate participant enrolment to reach target sample size	8, 9

Methods: Assignment of interventions (for controlled trials)

Allocation:

Sequence generation	16a	Method of generating the allocation sequence (eg, computer-generated random numbers), and list of any factors for stratification. To reduce	9, 10

For peer review only - http://bmjopen.bmj.com/site/about/guidelines.xhtml

		predictability of a random sequence, details of any planned restriction (eg, blocking) should be provided in a separate document that is unavailable to those who enrol participants or assign interventions	
Allocation concealment mechanism	16b	Mechanism of implementing the allocation sequence (eg, central telephone; sequentially numbered, opaque, sealed envelopes), describing any steps to conceal the sequence until interventions are assigned	9, 10
Implementation	16c	Who will generate the allocation sequence, who will enrol participants, and who will assign participants to interventions	9, 10
Blinding (masking)	17a	Who will be blinded after assignment to interventions (eg, trial participants, care providers, outcome assessors, data analysts), and how	9, 10
	17b	If blinded, circumstances under which unblinding is permissible, and procedure for revealing a participant's allocated intervention during the trial	9, 10
**Methods: Data collection, management, and analysis**			
Data collection methods	18a	Plans for assessment and collection of outcome, baseline, and other trial data, including any related processes to promote data quality (eg, duplicate measurements, training of assessors) and a description of study instruments (eg, questionnaires, laboratory tests) along with their reliability and validity, if known. Reference to where data collection forms can be found, if not in the protocol	11, 12, 13
	18b	Plans to promote participant retention and complete follow-up, including list of any outcome data to be collected for participants who discontinue or deviate from intervention protocols	14, 16
Data management	19	Plans for data entry, coding, security, and storage, including any related processes to promote data quality (eg, double data entry; range checks for data values). Reference to where details of data management procedures can be found, if not in the protocol	14
Statistical methods	20a	Statistical methods for analysing primary and secondary outcomes. Reference to where other details of the statistical analysis plan can be found, if not in the protocol	14, 15
	20b	Methods for any additional analyses (eg, subgroup and adjusted analyses)	N/A
	20c	Definition of analysis population relating to protocol non-adherence (eg, as randomised analysis), and any statistical methods to handle missing data (eg, multiple imputation)	N/A
**Methods: Monitoring**			
Data monitoring	21a	Composition of data monitoring committee (DMC); summary of its role and reporting structure; statement of whether it is independent from the sponsor and competing interests; and reference to where further details	16

For peer review only - http://bmjopen.bmj.com/site/about/guidelines.xhtml

		about its charter can be found, if not in the protocol. Alternatively, an explanation of why a DMC is not needed	
	21b	Description of any interim analyses and stopping guidelines, including who will have access to these interim results and make the final decision to terminate the trial	16
/Harms	22	Plans for collecting, assessing, reporting, and managing solicited and spontaneously reported adverse events and other unintended effects of trial interventions or trial conduct	N/A
Auditing	23	Frequency and procedures for auditing trial conduct, if any, and whether the process will be independent from investigators and the sponsor	N/A
**Ethics and dissemination**			
Research ethics approval	24	Plans for seeking research ethics committee/institutional review board (REC/IRB) approval	2, 16, 18
Protocol amendments	25	Plans for communicating important protocol modifications (eg, changes to eligibility criteria, outcomes, analyses) to relevant parties (eg, investigators, REC/IRBs, trial participants, trial registries, journals, regulators)	16, 17
Consent or assent	26a	Who will obtain informed consent or assent from potential trial participants or authorised surrogates, and how (see Item 32)	18
	26b	Additional consent provisions for collection and use of participant data and biological specimens in ancillary studies, if applicable	N/A
Confidentiality	27	How personal information about potential and enrolled participants will be collected, shared, and maintained in order to protect confidentiality before, during, and after the trial	17, 18
Declaration of interests	28	Financial and other competing interests for principal investigators for the overall trial and each study site	17, 18
Access to data	29	Statement of who will have access to the final trial dataset, and disclosure of contractual agreements that limit such access for investigators	18
Ancillary and post-trial care	30	Provisions, if any, for ancillary and post-trial care, and for compensation to those who suffer harm from trial participation	N/A
Dissemination policy	31a	Plans for investigators and sponsor to communicate trial results to participants, healthcare professionals, the public, and other relevant groups (eg, via publication, reporting in results databases, or other data sharing arrangements), including any publication restrictions	18
	31b	Authorship eligibility guidelines and any intended use of professional writers	N/A

For peer review only - http://bmjopen.bmj.com/site/about/guidelines.xhtml

	31c	Plans, if any, for granting public access to the full protocol, participant-level dataset, and statistical code	18
**Appendices**			
Informed consent materials	32	Model consent form and other related documentation given to participants and authorised surrogates	Suppl. material
Biological specimens	33	Plans for collection, laboratory evaluation, and storage of biological specimens for genetic or molecular analysis in the current trial and for future use in ancillary studies, if applicable	N/A

*It is strongly recommended that this checklist be read in conjunction with the SPIRIT 2013 Explanation & Elaboration for important clarification on the items. Amendments to the protocol should be tracked and dated. The SPIRIT checklist is copyrighted by the SPIRIT Group under the Creative Commons "Attribution-NonCommercial-NoDerivs 3.0 Unported" license.

For peer review only

BMJ Open

Nurture Early for Optimal Nutrition (NEON) participatory learning and action women's groups to improve infant feeding and practices in South Asian infants: pilot randomised trial study protocol

Journal:	*BMJ Open*
Manuscript ID	bmjopen-2022-063885.R4
Article Type:	Protocol
Date Submitted by the Author:	25-Oct-2023

Complete List of Authors:	Manikam, Logan; UCL Institute of Epidemiology and Health Care, Epidemiology and Public Health; Aceso Global Health Consultants<br>Allaham, Shereen ; UCL Institute of Epidemiology and Health Care, Department of Epidemiology and Public Health<br>Patil, Priyanka; University College London Institute of Epidemiology and Health Care; Aceso Global Health Consultants Pte Limited<br>Naman, Maryan; Aceso Global Health Consultants Pte Limited<br>Ong, Zhen Ling; Aceso Global Health Consultants Limited<br>Demel, Isabel-Cathérine; King's College London Faculty of Life Sciences and Medicine, Guy's, King's and St.Thomas' School of Medicine<br>Batura, Neha; University College London, Institute for Global Health<br>Llewellyn, Clare; University College London Department of Behavioural Science and Health<br>Hayward, Andrew; University College London, Epidemiology and Public Health<br>Lakshman, Rajalakshmi; Cambridgeshire County Council, Public Health Directorate; University of Cambridge, Medical Research Council Epidemiology Unit<br>Gilmour, Jenny; Mile End Hospital, Tower Hamlets GP Care Group<br>Webb-Martin, Kelley; London Borough of Newham, Children's Health 0-19 Service<br>Irish, Carol; London Borough of Newham, Children's Health 0-19 Service<br>Archibong, Mfon; London Borough of Newham, Children's Health 0-19 Service<br>Clarkson, Corinne; London Borough of Waltham Forest, Council, Public Health Strategist for Maternal & Child Health<br>Delceta, Daley; Chingford Health Centre Waltham Forest, Operational Lead<br>Islam, Lily; London Borough of Tower Hamlets, Community Facilitator<br>Bajwa, Seema; London Borough of Newham, Community Facilitator<br>Malek, Sabiha; London Borough of Newham, Community Facilitator<br>Bhachu, Jasvir; London Borough of Newham, Community Facilitator<br>Pushpakanthan, Geronimi; London Borough of Newham, Community Facilitator<br>Heys, Michelle; University College London Institute of Child Health, University College London Institute of Child Health; Specialist Children and Young People's Services, East London NHS Foundation Trust<br>Lakhanpaul, Monica; UCL Great Ormond Street Institute of Child Health

	Population Policy and Practice, Institute of Child Health; Whittington Health NHS Trust
**Primary Subject Heading**:	Public health
Secondary Subject Heading:	Epidemiology, Evidence based practice, Global health, Nutrition and metabolism, Public health
Keywords:	Community child health < PAEDIATRICS, NUTRITION & DIETETICS, PAEDIATRICS, PUBLIC HEALTH

SCHOLARONE™
Manuscripts

Nurture Early for Optimal Nutrition (NEON) participatory learning and action women's groups to improve infant feeding and practices in South Asian infants: pilot randomised trial study protocol

Short Title/ Acronym: Nurture Early for Optimal Nutrition (NEON) Pilot Feasibility Randomised Controlled Trial

Authors:

[1]Dr Logan Manikam, NIHR Advanced Fellow & Honorary Consultant in Public Health Medicine[1,2]

Shereen Allaham, Research Assistant & Consultant[1]

Priyanka Patil, Research Assistant & Consultant[1,2]

Maryan Naman, Consultant[2]

Zhen Ling Ong, Associate[2]

Isabel-Cathérine Demel, Medical Student[2,3]

Dr Neha Batura, Associate Professor in Health Economics[6]

Dr Clare Llewellyn, Associate Professor in Behavioural Obesity Research[7]

Prof Andrew Hayward, Professor of Infectious Disease Epidemiology and Inclusion Health Research[1]

Dr Rajalakshmi Lakshman, Consultant in Public Health Medicine[8]

Jenny Gilmour, 0-19 Service Development Lead THGPCG[9]

Kelley Webb Martin, Director of Delivery, Compliance & Transformation[10]

Carol Irish, Head of Children's Health 0-19 Service[10]

Mfon Archibong, Specialist Health Visiting Research Lead[10]

Corinne Clarkson, Public Health Strategist for Maternal & Child Health[11]

Daley Delceta, Operational Lead[12]

Lily Islam, Tower Hamlets community facilitator

Seema Bajwa, Newham community facilitator

Sabiha Malek, Newham community facilitator

Jasvir Bhachu, Newham community facilitator

Geromini Pushpakanthan, Newham community facilitator

[2]Dr Michelle Heys, Associate Professor of Community and Population Child Health, Consultant Paediatrician[4,5]

Monica Lakhanpaul Professor of Integrated Community Child Health[13,14] , On Behalf of the NEON Steering Team.

Membership of the NEON Steering Team is listed in the Acknowledgements

---

[1] Logan Manikam and Shereen Allaham are joint first authors

[2] Dr Michelle Heys and Professor Monica Lakhanpaul are joint senior authors

For peer review only - http://bmjopen.bmj.com/site/about/guidelines.xhtml

For peer review only

Affiliations:

1. Department of Epidemiology and Public Health, University College London Institute of Epidemiology and Health Care, London, UK
2. Aceso Global Health Consultants Pte Limited, Singapore
3. Department of Life Sciences & Medicine, King's College London GKT School of Medical Education, London, UK
4. Specialist Children and Young People's Services, East London NHS Foundation Trust, 84 West Ham Lane, London E15 4PT.
5. Population, Policy and Practice, UCL Great Ormond Street Institute of Child Health, 30 Guildford Street, London WC1N 1EH, UK
6. Institute for Global Health, University College London, 30 Guilford Street, London WC1N 1EH.
7. Department of Behavioural Sciences & Health, UCL IEHC, 1-19 Torrington Pl, Fitzrovia, London WC1E 7HB.
8. MRC Epidemiology Unit, University of Cambridge
9. Tower Hamlets GP Care Group, Mile End Hospital, London, UK
10. Children's Health 0-19 Service, London Borough of Newham, 1000 Dockside Road E16 2QU
11. Waltham Forest Council, London, UK
12. Chingford Health Centre Waltham Forest, London, UK
13. Population, Policy and Practice, UCL Great Ormond Street Institute of Child Health, University College London, London, WC1N 1EH, UK
14. Whittington Health NHS Trust, London, UK, N19 5NF

Corresponding author: Dr Logan Manikam

Email: logan.manikam.10@ucl.ac.uk
Telephone: +44 7868721152

Key Words: Community-based intervention; infant; child health; nutrition; feeding practices; clinical trial

Word Count: 3999

For peer review only

ABSTRACT

Introduction: Feeding practices developed in early life can impact a child's nutrition, growth, dental health, cognitive development and lifetime risk of chronic diseases. Substantial evidence suggests ethnic health inequalities, and non-recommended complementary infant feeding practices among UK's South Asian (SA) population. Nurture Early for Optimal Nutrition (NEON) aims to use Women's group Participatory Learning and Action (PLA) cycles to optimise infant feeding, care and dental hygiene practices in SA infants <2 years in East London.

Methods and analysis: A three-arm pilot feasibility cluster randomised controlled trial will assess feasibility, acceptability, costs and explore preliminary effectiveness for proposed primary outcome (i.e. reporting on BMI z-score). Multilingual SA community facilitators (CFs) will deliver the intervention, Group PLA Cycle, to mothers/carers in respective ethnic/language groups. 12 wards are randomised to face-to-face PLA, online PLA and usual care arms in 1:1:1 ratio. Primary outcomes are feasibility and process measures (i.e. BMI z-score, study records, feedback questionnaires, direct observation of intervention, and sustainability) for assessment against Go/Stop criteria. Secondary outcomes are cluster-level and economic outcomes (i.e. eating behaviour, parental feeding practices, network diffusion, children development performance, level of dental caries, GP utilisation, costs, staff time). Outcomes are measured at baseline, every two weeks during intervention, 14-weeks and at 6-months by blinded outcome assessors where possible. This study will use concurrent mixed-methods evaluation. Quantitative analyses include descriptive summary with 95% confidence interval (CI) and sample size calculation for the definitive trial. The intervention effect with CI will be estimated for child BMI z-score. Implementation will be evaluated qualitatively using thematic framework analysis.

Ethics and dissemination: Ethics approval was obtained from UCL, NHS (HRA and HRCW). Results will be published in peer-reviewed journals, presented at scientific conferences/workshops with commissioners, partners and participating communities. Plain language summaries will be disseminated through community groups, websites and social media.

Word Count: 299/300

Trial registration: This trial has been registered with the International Standard Randomised Controlled Trial Number Register: IRAS-ID – 296259 (ISRCTN10234623).

For peer review only - http://bmjopen.bmj.com/site/about/guidelines.xhtml

ARTICLE SUMMARY

Strengths

- The three-armed approach will allow for comparisons of online and face to face delivery with usual care
- The collection and interrogation of process and feasibility data with clearly defined stop-go criteria will allow for an informed decision around feasibility of a larger scale evaluation
- A broad set of secondary outcome measures will allow for estimation of potential impact, in addition to understanding of the feasibility of capturing reliable and complete data measuring these outcomes

Limitations

- Project capacity precludes the spacing of group sessions according to religious celebrations which might affect retention to group session.
- Risk of performance and measurement bias as neither participants nor all research staff will be fully blinded.

For peer review only

INTRODUCTION

The first 1,000 days of life is important for growth and brain development. There is mounting evidence that influences during pregnancy and infancy may alter lifetime risk of nutrition and dental related diseases [1]. Feeding practices developed during this period can impact children's nutrition, growth, dental health, cognitive development, and may lead eventually to heart disease, obesity, and diabetes [2].

Britain's ethnically diverse population is mostly disadvantaged across a range of socioeconomic outcomes, forming fundamental causes of ethnic health inequalities in the UK [3]. Some of the widest differences have been observed in the South Asian (SA) population, more so for Pakistani and Bangladeshi communities compared to Indians [4].

Systematic reviews assessing complementary feeding practices and the sociocultural beliefs underpinning them in children <2 years within SA families in the UK were explored. Despite the adoption of the WHO Infant and Young Children Feeding Guidelines, there remains substantial evidence of non-recommended complementary feeding practices such as early introduction of solids, introduction of minimum dietary diversity or minimum frequency of meals being followed [5-7]. Contributing factors that persisted post-migration included bicultural issues or low acculturation levels and conflicting information among health professionals, extended family, and religious and community leaders.

Effective early life interventions tailored to different ethnic groups have great potential to reduce the development of short- and long-term conditions and, thereby, lifetime inequalities. At present, however, few of such interventions exist [8]. Traditionally, UK health services provide unidirectional information based on guidelines and NHS recommendations. Specific ethnic groups may be marginalised by this approach as most advice is not tailored to their cultural practices. There is a need for interventions that target these communities through a top-down unidirectional approach and bring a change originating from within the communities. Therefore, it is important to work in partnership with communities thereby building their capacity to work closely with local authorities/stakeholders. The asset-based community development approach (ABCD) has been successful in community development as this helps communities identify their assets through asset mapping and mobilize them to bring along the desired change [9].

Based on Lakhanpaul et al.'s MARKS (Motivation Awareness Resources Knowledge and Skills) model [10], current practice lacks the space to understand parents' motivation and ability to support families in skills development, all of which is key to providing optimal infant feeding, care, and dental hygiene support. Considering the resource constraints of the NHS and its 10 Year Forward Plan which aims to shift the emphasis to the community, highlighting a need for interventions that enable communities to utilise their available community assets. The Participatory Learning and Action (PLA) cycle is an iterative process led by multilingual facilitators through a four-stage cycle of identifying and prioritising contextual issues, designing problem-solving strategies, implementing these strategies and a post-implementation evaluation. The PLA approach is a low-cost, community-based, culturally

For peer review only - http://bmjopen.bmj.com/site/about/guidelines.xhtml

sensitive intervention that can be adapted from Low- and Middle-income Countries (LMICs) to High-income Countries (HICs), as well as to different population groups and topic areas. This strategy is also recommended by the WHO to improve maternal and infant survival [11]. A meta-analysis of seven cluster randomised controlled trials (RCTs) utilising PLA in LMICs showed reduced maternal and neonatal mortality by 37% and 23% respectively [12].
An RCT and controlled before-and-after study have demonstrated reduced maternal depression, increased exclusive breastfeeding rates, and decreased under-5 morbidity [13]. A Mumbai-based RCT also showed improvements in maternal practices and care-seeking behaviour [14]. Recognising WHO recommendations and the success of PLA in LMICs, this approach was adopted for the Nurture Early for Optimal Nutrition (NEON) programme.

In NEON Phase 1 (Intervention Development) was co-developed with the SA community facilitators (CF) (now community researchers/independent observers in NEON Phase 2), community members, research team and independent experts (i.e. health visitors, dentists, dieticians, nutritionists, GPs, and midwives). This followed the MRC complex intervention framework , which involved using formative research and prior trials as evidence-based factors; micro-adaptations involved adjusting language, literacy levels for materials, using picture cards, face-to-face, and interactive learning delivery methods. This protocol describes NEON Phase 2: Pilot Feasibility Cluster RCT.

AIMS AND OBJECTIVES

This pilot feasibility study aims to evaluate the feasibility and inform the design and conduct of a definitive cluster RCT comparing NEON Women's Group PLA Cycle versus usual care to optimise infant feeding, care, and dental hygiene practices in SA infants aged <2 years in East London.

Primary objectives:

Assess: 1) the feasibility of proceeding to a definitive trial against predetermined Go/Stop criteria (i.e. recruitment, retention rates, intervention support, acceptability),2) intervention fidelity and participant' adherence, 3) implementation of face-to-face versus online intervention arms

Secondary objectives:

Assess: 1) feasibility, completeness, acceptability and adequacy of blinding when collecting proposed outcomes to establish optimal outcomes and data collection procedures, 2) time needed to competently deliver both versions of NEON intervention, 3)the mean, standard deviation and intervention effect with 95% confidence interval (CI) of the proposed primary outcome, child BMI z-score

For peer review only - http://bmjopen.bmj.com/site/about/guidelines.xhtml

METHODS AND ANALYSIS

Design

This three-arm pilot feasibility cluster RCT study is expected to run from July 2021-May 2023 in 3 East London boroughs. 18 clusters defined as borough wards will be randomised with 1:1:1 allocation to 2 intervention arms (face-to-face or online NEON Women's Group PLA Cycle) and 1 control arm (usual care). An online arm enables the trial to continue under Covid-19 restrictions; usual care was selected as control to avoid depriving participants of essential and available infant and maternal care services. Multilingual CFs will deliver interventions in respective ethnic/language groups. Ward-level randomisation is a trade-off between minimising contamination and ensuring maximum SA population representation given the diversity in the ethnic makeup, i.e. SA sub-groups, of the aforementioned boroughs.

Patient and Public Involvement

The research has been co-designed with SA CFs involved in all stages of developing and evaluating the intervention to ensure relevant research questions and an acceptable study design. Development phase, representing different SA communities, are now recruited as Community Researchers (CRs) to support; protocol development and ethics application by ensuring participants-facing documents are clear, appropriate and sensitively-worded; topic guide and questionnaire development; strategy development and troubleshooting (e.g. recruitment); interpretation of findings into appropriate and attainable recommendations for practice; review and revision of draft academic papers; dissemination activities and development of plain language summaries.

Setting

This study will run sequentially in the London Boroughs of Tower Hamlets (TH), Newham (NH) and Waltham Forest (WF), all of which are considered to be among 5% most deprived in England [15]. The boroughs were analysed in terms of ethnic make-up; in each borough, the 6 wards with the highest density of Asian population were selected based on census data [16]. The most common SA ethnic/language groups will be targeted. Figure 1 shows the number of wards and PLA groups by boroughs. Figure 2 shows the overview of participant flow throughout the trial.

Figure 1. Overview schematic detailing number of wards & PLA groups by East London boroughs

Figure 2. Overview of the NEON pilot feasibility RCT design and participant flow

For peer review only - http://bmjopen.bmj.com/site/about/guidelines.xhtml

Randomisation

Randomization of wards will be done before the recruitment by a separate member at UCL using the Research Randomizer software for the 18 wards stratified by borough [17]. The randomised wards will be shared with the RA at the end of recruitment. Once sufficient numbers are recruited in an ethnic/language group, the RA will assign participants to their respective arms. Recruitment will be performed by NHS personnel who will not be aware of the randomisation. CFs/CRs will be made aware of the participant allocation before the PLA sessions start to make contact and invite participants

Eligibility Criteria

Participants

Inclusion

- Mothers or female carers of an infant <24 months;
- Indian, Pakistani, Sri Lankan or Bangladeshi background;
- Resident in a randomised study ward in TH, NH and WF;
- Willing and able to provide informed consent

Exclusion

- <18 years old;
- Anticipating moving out of *a priori* defined geographical areas before or after delivery
- Current or recent participation in another study within 4 weeks of trial commencement.

Staff (Community Facilitator) Eligibility

- Female
- Have at least one child, preferably <24 months
- From the SA community in TH, NH, or WF
- Able to read and write
- Fluent in English and another local language (Hindi, Bengali, Sylheti, Urdu, or Tamil)
- Understand SA social norms, values and culture within study boroughs
- Known and respected by their local community
- Motivated to address issues related to infant growth and development
- Able to manage a group and have some leadership qualities

For peer review only

Sample size

With communication and cultural sensitivity as key drivers of the NEON intervention, the sample size will partly depend on the coverage of ethnic and language groupings. The choice of ethnic/language groups were informed by the 2011 ONS Census [16] and input from CFs stressing that, while language is important for communication, people's practices are embedded in different ethnicities.

The main ethnic/language group(s) identified are: 1 group in TH (Bangladeshi/ Bengali & Sylleti); 4 groups in NH (Indian/Gujrati, Indian/Punjabi, Pakistani/Urdu, and Sri Lankan/Tamil); 3 groups in WF (Indian/Punjabi, Pakistani/Urdu, Sri Lankan/Tamil). In each borough, 1 Women's Group PLA Cycle will run per ethnic/language group per ward.

We aim to run 20 to 32 Women's Group PLA Cycle (with equal numbers of face-to-face and online PLA groups) at 6-8 participants per PLA group. Including control groups totals to 288-384 participants. Assuming an 80% recruitment or retention rate, this enables us to be 85% confident that the true population rate will fall between 0.76%-0.84% (precision=0.04). This was estimated using a geometric mean cluster size=16 participants, cluster size coefficient of variation (COV)=0.7 and Intra-Cluster Correlation Coefficient (ICC)=0.02.

Recruitment

Participants

Due to the diversity of the target population, multiple recruitment methods are needed to maximise reach and minimise inequalities or non-representative samples [18] [19]. For instance, relying solely on online recruitment may be ineffective due to digital poverty. Thus, other mediums including leveraging on existing social networks (snowballing), identifying and fostering collaborations with community leaders, and creating clear and succinct recruitment materials [20] were employed. All study promotional materials (e.g. participants information sheets, posters) will be available in English and local languages. To standardise the recruitment process, we will provide the same recruitment script and study materials to all recruiters. Potential participants will contact the RA, who will confirm eligibility, consent and register them into the study.

Participant recruitment will follow three strategies with regular advice by CRs to maximise reach:

1)Recruitment through CFs: CFs will share study materials with their network to invite eligible participants with subsequent snowballing. We will provide training to standardise CF recruitment. 2)Recruitment through Health Visitor (HVs), GPs, and Midwifery teams: HVs, GPs, and midwives who have been identified based on their geographical proximity to study wards will share study materials to eligible participants. Details of those interested will be given to the RA. 3)Online recruitment: Online social media campaigns on Facebook and Instagram will be utilised. See Supplementary Material-1 for advertisement materials. Recruitment bias will

For peer review only - http://bmjopen.bmj.com/site/about/guidelines.xhtml

be limited due to the above-mentioned multiple channels of recruitment and all recruiters will be blinded thereby not knowing the allocation details at the time of recruitment.

Staff

We will recruit CFs from our local stakeholders' network. Recruitment via CFs is particularly useful for studies with underserved or traditionally hard-to-reach populations [21]. CFs are leaders/champions in their local community who, with training and support, help improve the health and wellbeing of their communities. In line with NIHR's "INVOLVE" guideline, CFs will be paid £30 for each PLA meeting and additional £5 to cover travel costs for face-to-face meetings. A comprehensive manual for the CFs will be developed with PLA experts. A tool kit will be provided to support the learnings. The CFs will attend a three-day comprehensive workshop and additional sessions will be conducted in between the 8-meeting cycle as a refresher and address any challenges. Biweekly meetings with the RA will be conducted to discuss learnings from the sessions. The CFs will be paid through vouchers for their time similar to a zero-hour contract. HV, GP and midwives would be identified and recruited by our study partners at each study ward. Payments will be supported by NHS support costs and decided with the relevant clinical lead.

Allocation concealment and blinding

Firstly, treatment allocation will be concealed from participants and recruiters during participant recruitment to reduce recruitment bias. After recruitment has been completed for that ethnic/language group in the borough, the RA will reveal ward allocation to participants and CFs via text or email, listing postcodes by trial arms to allow CFs to effectively organise and deliver the intervention.

Secondly, outcome assessors (CRs) will be blinded where possible to reduce measurement bias. This will be achieved by CRs logging the data collected onto an electronic platform that automatically links participant ID to maintain blinding. Baseline measurement of BMI will be done by health visitors in each borough who will be blinded.

Intervention

NEON Women's Group PLA Cycle

The co-adapted NEON Women's Group PLA Cycle tailored to ethnic/language groups will be delivered face-to-face at community/children's centre or online over 14 weeks (1 session per 2 weeks). HVs, GP, and midwives' teams will be invited to 1-2 sessions to provide evidence-based information upon participants' request. To reduce study contamination, participants may not switch wards (i.e. switch arms) after treatment allocation is revealed. Figure 3 illustrates the aim of each PLA session.

Figure 3. NEON Women's Group PLA Cycle

A culturally-sensitive intervention package has been co-developed consisting of:

1. PLA group facilitator manual
2. Picture cards with recommended and non-recommended infant feeding, care and dental hygiene practices, and facilitators/barriers to uptake
3. Healthy baby food recipes

For peer review only - http://bmjopen.bmj.com/site/about/guidelines.xhtml

4. Community asset maps (e.g. low-cost fruit/vegetable shops)
5. List of relevant resources and services.

Online arm participants may access this toolkit through the eRedbook platform and receive NEON information the via the app or website. A Logic Model (Supplementary Material-2) shows the relationship between these activities and resources and their intended short-, medium- and long-term effects.

Control (Usual Care)

In all wards, HV teams have regular mandatory postnatal visits for all families of new-borns and infants. These visits are immediately after birth, 6-8 weeks, 12-16 weeks, 1 year, and 2-2.5 years. One optional prenatal visit is conducted by HVs between 28-32 weeks of pregnancy. Supplementary Material-3 lists other initiatives and resources available in each study borough as part of usual care.

Outcome

Primary outcomes: Feasibility and process outcome measures
We will collect quantitative and qualitative data to assess Go/Stop criteria (Table 1) on recruitment and retention rates, intervention support, acceptability, fidelity and participant adherence using:

1. *Study record:* An enrolment log will record all eligible participants, total enrolled, reasons for non-participation, number followed-up on child BMI z-score, and the date on how many people responded to adverts/invitations. Pre-screen failure logs will record those eligible but not enrolled, with reasons. PLA session audio/video recordings will be collected using secured Dictaphone, video cameras, CCTV, and/or Zoom recording.

2. *Process measurements:* These include Participants' Feedback Questionnaire, Facilitator's Feedback Form, PLA Cycle Meeting Register, direct observation of intervention delivery and CF performance by CRs, and Sustainability Assessment Tool (Supplementary Material-4).

Definite Go	Definite Stop
>=50% of eligible participants consenting to pilot feasibility study	<40% of eligible participants consenting to pilot feasibility trial
>=80% of mothers attend >=60% of planned sessions in the intervention arm.	<20% of mothers attend >=60% of sessions as planned in each intervention arm
Retention of >=70% of consented participants for primary outcome data collection	Retention of <50% of participants at 6 months
High intervention support with respect to content, frequency, duration, and quality	Low support of Intervention Procedures

For peer review only - http://bmjopen.bmj.com/site/about/guidelines.xhtml

Intervention is perceived as acceptable	Intervention perceived as unacceptable

Table 1: Go/ Stop criteria for the definitive trial[3]

Secondary outcomes: Proposed outcomes for the definitive trial

All other individual-level, cluster-level and economic outcome measures proposed for the definitive trial will be assessed on the response, completion rates, acceptability and the adequacy of blinding for outcome assessors during data collection. Table 2 summarises outcome measures and data collection timing. See Supplementary Material-5 for collection tools.

Outcome	Outcome Measures	Data Collection	Timing			
			Baseline	Every 2 weeks (end of each PLA meeting)	14 weeks (end of PLA cycle)	6 months
Proposed primary individual-level outcome	Individual Child BMI z-score	RA, CRs	✓		✓	✓
Proposed secondary individual-level outcome	Children feeding behaviour[3]	RA, CRs	✓		✓	✓
	Parental feeding style[3]	RA, CRs	✓		✓	✓
	Audio/Video recording of child eating behaviours & parental feeding practices	RA	✓		✓	✓
	4-day food diary[4]	RA, CRs	✓		✓	✓
	Network diffusion†[3]	RA, CRs	✓			✓
Proposed secondary cluster-level outcome	Children's development performance	RA, CRs		✓		✓
	Level of dental caries	RA	✓			✓

[3] The study will have to meet the Definite Go criteria in order for the study to be feasible and to be able to proceed. However, if any one of the Definite Stop criteria is met then the study stops. The 'Go/Stop' indicates that it is either feasible or not feasible to proceed to a definitive trial. The progression rules have been approved by the steering and data management team. Should any of the progression rules not be met, assessments and adjustments of the NEON pilot feasibility RCT will be negotiated before proceeding to a definitive trial.

[4] Community Facilitators will assist in administering or completing these data collection tools before sending them to RA/ CRs during the intervention period; †For intervention groups only; CR= Community Researchers; RA = Research Assistant

For peer review only - http://bmjopen.bmj.com/site/about/guidelines.xhtml

	GP healthcare utilisation	RA	✓			✓
**Economic outcome**	Cost tool	RA	✓			✓
	Partners time questionnaire	RA				✓
**Process outcome†**	Participants Feedback[3]	RA, CRs		✓		
	PLA cycle meeting register[3]	RA, CRs		✓		
	Direct observation	RA, CRs		✓		
	Sustainability assessment	RA, CRs		✓		

Table 2. Schedule of Outcome Assessments

Primary individual-level outcome

1. *Child BMI z-score:* Health visitors will measure infants' length/height, head circumference and weight. The RA will refer to the WHO 2006 growth standard [2], using age- and gender-adjusted length or height(m)/weight $(kg)^2$ to provide a comparison on the cluster-level.

Secondary individual-level outcomes

1. *Children's feeding behaviour* will be measured using 6 of 8 domains adapted from the validated Children's Eating Behaviour Questionnaire (CEBQ) deemed suitable for infants: food responsiveness; enjoyment of food; emotional overeating; desire to drink; satiety responsiveness; slowness in eating; emotional undereating; and food fussiness [7, 8, 22]. Obesity risk could be linked with external eating, emotional overeating, and food fussiness [7].

2. *Parental feeding style* will be assessed with a validated self-reported Parental Feeding Style Questionnaire (PFSQ) with four scales: emotional feeding; instrumental feeding; encouragement to eat; and control overeating [23].

3. *Audio/video recording of child eating behaviour and parental feeding practices* [24] involves asking participants about the meal (e.g. name, ingredients) and creating annotations that link to behaviour codes identified from NEON formative study [25]. Before-and-after audio/video recordings will be thematically analysed using Elan Software [26] to measure intervention effect.

4. *Child food intake*: The 4-day food diary [7] is a self-reported, prospective, open-ended survey that collects detailed quantitative estimates of food consumption of infants between 4-18 months [6]. It assesses nutrient intake (e.g. total fat, total carbohydrate, salt, sugar) by comparing with age/sex specific UK dietary reference values [27]. Dietary data will be presented by age group (e.g. 4-6 months, 7-9 months, 12-18 months).

5. *Network diffusion of study materials between participants and their communities*: CRs will track the number of downloads using the eRedbook platform. Participants will also complete a questionnaire on the number of people they shared the material with, their family size, age, gender, relationship to the participant and platform used.

6. *Equality impact assessment (EIA)*: This trial should reflect the diversity of the target population by enabling equal access while accommodating different needs. An EIA tool will systematically assess likely effects of the intervention on people (e.g. with respect to disability,

For peer review only

gender, ethnicity, age, sexual orientation, religion/belief etc.), mitigate adverse impacts and identify active steps to address existing disadvantage and promote equality.

Secondary cluster-level routine outcomes

Although this intervention acts on the individual-level, we expect a social diffusion of good infant feeding and dental hygiene awareness and practices in the medium- to long-term (Supplementary Material-2, Logic Model). While immediate cluster-level changes are not expected, we would like to pilot cluster-level routine data collection.

1. Development performance of children: The Ages and Stages questionnaire (ASQ-3) include communication, gross motor, fine motor, problem-solving, and personal-social domains [5]. Three versions are available: 24-months, 27-months and 30-months. ASQ-3 data collected by HVs during mandated visits are stored in EMIS/ RIO databases.
2. GP healthcare utilisation data is available in participants' medical record.
3. Level of dental caries and other signs of tooth decay is available in NHS dental services records for registered patients beginning at 6-months of age and approximately every half-year following initial visit [17,28,29].

Economic outcomes

1. Cost: The RA will use a spreadsheet will capture the cost of the NEON Intervention Development
and pilot feasibility RCT phases every 6 months.
2. Partners' time used: A questionnaire will capture time spent for the delivery of NEON-associated programmes.

Data Collection and Management

Outcome data will be collected at baseline, every 2-weeks during intervention (for process measures), 14-weeks immediately post-intervention, and 6-months follow-up (for economic data). During the intervention period, several outcome measurements will be administered by CFs to participants or completed by CFs and sent to CRs/RA. Other outcomes and post-intervention outcomes will be assessed directly by CRs/RA (Table 2). Each case will be assigned an ID. Data collected digitally or in paper format will be stored in UCL S: Drive and/or UCL cabinets. We will use RedCap electronic data management system [30]; the RedCap API allows flexible and straightforward data import and export to data analysis software.
We will form a data sharing agreement with study partners for the RA to extract and link routine cluster-level data to trial participants. To guarantee confidentiality, pseudonymised participant data will be stored on encrypted, password-protected computer or UCL S:Drive accessible only to study staff and authorised personnel. Password-protected personal data will be stored separately from trial data on secure UCL computers. Only pseudonymised quotes or data from audio/video recording may be published. Personal data and audio/video recordings will be stored for 3-years post-study and destroyed; research data will be stored for 20-years post-study.

Analysis

For peer review only - http://bmjopen.bmj.com/site/about/guidelines.xhtml

Overall, quantitative and qualitative data will be analysed concurrently at multiple time points to identify early problems that are rectified as the trial progresses. Quantitative data such as the BMI z-score will be descriptively summarised using mean and standard deviation (SD) for continuous variables, number with percentages for categorical variables, and 95% CI with a breakdown of participants by trial arm, ethnic/language group and borough where relevant. An intention to treat analysis will also be considered. Results will be collectively assessed against the Go/Stop criteria. Before progression to the definitive trial, we will systematically and rigorously assess areas of improvement using a structured discussion tool: A process for Decision-making after Pilot and feasibility Trials (ADePT) [31]. All statistical analysis will be done by an independent statistician who would be blinded.

Sample Characteristics

Participant demographic characteristics will be descriptively summarised by trial arm. We will check for recruitment bias across different arms at baseline and compare later recruits who are prone to unblinded recruitment.

Feasibility and Process Measures

Across the three arms, we will check for any differential recruitment or retention rates and any consequent systematic differences in participant characteristics over time, as control arm participants may be less keen to participate or remain in the study. For the two intervention arms, we will conduct a mixed-methods implementation evaluation to explore the possibility of a blended approach in the definitive trial as some features of each delivery mode might be particularly salient. Qualitative data will be analysed thematically using framework analysis that include participants' motivation for engagement, expectations, experiences, intervention acceptability, implementation barriers, and suggestions for improvement. Negative 'deviant' cases will inform interpretation.

Outcome measures for the definitive trial

1. *Feasibility of collecting outcome measures:* To assess completeness, degrees of missingness at the individual item level and the entire outcome measure will be reported overall, by time-point and trial arms. Other feasibility measures would be captured by analyses of process measures above.

2. *Intervention effect for proposed primary outcome (BMI z-score):* Conditioned upon low recruitment bias and sufficient adherence to trial protocol, we will estimate the difference in BMI z-score between each intervention arm versus the control arm. We will consider methods to pool data across age groups depending on the actual sample age breakdown.

3. *Sample size and power calculation for the definitive trial:* Data from the pilot feasibility trial will enable us to determine (i) the smallest difference of clinical importance (through multiple testing of several key endpoints for the proposed primary outcome), (ii) the clinically justifiable power-significance level or scientifically acceptable probability of "false positive" (type I error) results, and (iii) whether we need to adjust the calculated sample size for expected level of non-compliance/ drop-in and drop-outs. We will report the ICC.

4. *Economic Evaluation:* This will be conducted from a provider and user perspective which includes direct cost (e.g. time, resources spent on training and participation of CF, participants' overall household consumption and childcare costs). We will estimate the total cost, average annual cost, cost-effectiveness, equity impact analysis and compare across three arms.

Monitoring

The Principal Investigator (PI) will be responsible for day-to-day monitoring and study management.

For peer review only - http://bmjopen.bmj.com/site/about/guidelines.xhtml

The NEON Steering and Data Management Team will ensure adherence to all relevant regulations and principles of good clinical practice. The team consists of multidisciplinary experts in child health, practitioners, PLA expert, SA independent observers from NEON 1, and Queen Mary University of London Pragmatic Clinical Trials Unit. Committee meetings will be held every 3 months to review the Go/Stop criteria (Table 1) and interim analyses. The team will agree to the trial protocol and facilitate any necessary protocol amendments; provide independent expert advice (including on project management governance); monitor progress; ensure adequate deadlines are set and met; monitor and advise NEON Core Team (Trial Management Group) on strategic decisions in light of new evidence; and ensure successful delivery by meeting and reporting on study progress with the Core Team biannually. The PI and other Core Team members will attend all necessary meetings and report on the study progress.

Any adverse events or safety concerns will be captured in the Participant Feedback Questionnaire and Facilitator Report Form (every 2-weeks during intervention period) and reported to the steering and safety monitoring team.

The UCLH/UCL Joint Research Office, on behalf of the Sponsor (UCL), may conduct random audits in accordance with the UK Policy Framework for Health and Social Care Research and UCL's policies and procedures.

ETHICS AND DISSEMINATION

Ethical approval has been obtained from UCL Research Ethics Committee and NHS Health Research Authority. The participant information sheet will be provided in English or participants' native language in-writing and/or verbally by community facilitators for informed consent. The RA or CRs will obtain written or audio-recorded consent ≥24 hours later [32]. Participation will be voluntary and they may withdraw anytime without prejudicing treatment or usual care. No incentives will be provided except for small reimbursements (e.g. childcare at the local centre where PLA meetings take place).

UCL will provide insurance cover and we will also signpost any vulnerable participants who require protection from harm to relevant safeguarding organisations.

Regular progress updates will be shared via UCL or NIHR websites and patient group newsletters. Study findings will be submitted for 3-5 publications in high impact factor peer-reviewed journals and presented at national and international conferences. Researchers and community members from the steering team will contribute to confirming analysis and write-up, adhering to authorship guidelines. To reach wider audiences, we will (i) disseminate plain language summaries for each academic paper through community groups, social media and You Tube; (ii)present findings at lay-person meetings at community or children centres through the NIHR CLAHRC network and (iii)organise 2-3 annual workshops with commissioners, partner organisations and NEON community facilitators to share key findings and recommendations.

Acknowledgements

The authors would like to thank the South Asian community facilitators in the NEON Intervention Development phase: Lily Islam, Seema Bajwa, Aasma Baiyat, Jasvir Bhachu, Geromini Pushpakanthan, Sabiha Malek, Cllr Lakmini Shah; and community members of the London Boroughs of Tower Hamlet, Newham, and Waltham Forest for their important contribution and engagement to this research project. Furthermore, the authors would like to thank the 'Conversation Café' initiative led by Cllr

For peer review only

Lakmini Shah for her significant help in the recruitment of the community facilitators and members, and in identifying the community centres to carry out the intervention development meetings. The authors would also like to acknowledge the contribution of the NEON core team, steering team and all health experts in validating intervention NEON toolkit. We would like to thank the Women & Children First Charity and First Steps Nutrition Trust for their valuable contributions and guidance throughout the study. In addition to the authors, members of the NEON steering team consist of Prof Atul Singhal, Joanna Drazdzewska, Dr Sonia Ahmed, Amelie Gonguet, Gary Wooten, Dr Ian Warwick, Vaikuntanath Kakarla, Alex Nelson, Phoebe Kalungi, Prof Richard Watt, Prof Mitch Blair, Prof Audrey Prost, Dr Edward Fottrell, Ashlee Teakle, Prof Oyinlola Oyebode, Ciarán Forde, Keri McCrickerd, Dr Rana Conway, Subarna Chakraborty, Sumire Fujita, Tala Khatib and Delaney Douglas-Hiley.

Steering team members had an opportunity to critically review results and contribute to the process of finalising this paper.

Author's Contributions

ML, LM, MH, NB, CL, AH, SA conceived the original concept of the study and designed the research methodology. SA carried out the Intervention Development meetings, workshops, co-development of the intervention toolkit, wrote and devised the paper along with LI, SB, SM, JB and GP. LM, ML, ,MH, SA, PP, MN, ZO, NB, CL, AH, RL, JG, KWM, CI, MA, CC, DD validated the study and revised the manuscript critically for important intellectual content. ICD was involved in drafting materials for the NEON meetings and workshops of the Intervention Development Phase. MN and ICD contributed to the manuscript writing, and prepared it for submission. LM, SA, ML and MH had primary responsibility for the final content. All authors read and contributed to reviewing the study data, the designing of the manuscript, and the approval of the final manuscript.

Funding

Logan Manikam, Shereen Al Laham & Priyanka Patil are funded via a National Institute for Health Research (NIHR) Advanced Fellowship (Ref: NIHR300020) to undertake the Pilot Feasibility Cluster Randomised Controlled Trial of the *NEON* programme in East London. Prof Monica Lakhanpaul was funded by the NIHR Collaboration for Leadership in Applied Health Research and Care (CLAHRC) North Thames.

Disclaimer

The views expressed in the publication are those of the author(s) and not necessarily those of the sponsor (UCL), funder (NIHR), study partners (Tower Hamlets GP Care Group, London Borough of Newham Council, Waltham Forest Council).

Conflicts of Interest

The authors declared no potential conflicts of interest with respect to the research, authorship and/or publication of this paper.

Participant consent for publication

Participant information sheets and consent forms have been provided to community members and those expressing interest. Community participants have agreed to participate and given audio/video consent prior to their participation in the workshops. Verbal consent was witnessed and formally recorded. All participants have been informed of their right to freely withdraw from the study at any

For peer review only - http://bmjopen.bmj.com/site/about/guidelines.xhtml

time. Confidentiality of personal data has been ensured through the use of anonymisation techniques as stated in the Data Protection Act (1998) and in line with the General Data Protection Regulation (2018). All participant data has been anonymised and stored on an encrypted password protected computer. Data can only be accessed by the authorised research personnel.

Ethics approval

This study has obtained ethical approval from UCL Research Ethics Committee [Ethics ID 17269/001], Sponsor reference number: 142600, Funding Reference: NIHR300020 and IRAS number: 296259, Ref: 21/SW/0142. Study protocols and relevant documents have been reviewed by UCL Research Ethics Committee, NHS Health Research Authority (HRA) and study partners involved in establishing data sharing agreement for linking participants' routine data (Tower Hamlets GP Care Group, London Borough of Newham Council, Waltham Forest Council).

Provenance and Peer Review

Not commissioned; peer reviewed for ethical and funding approval prior to submission.

Data Sharing Statement

The data supporting the findings of this study is available upon reasonable request from the corresponding author.

CURRENT STUDY STATUS (AS OF JAN 2023)

The study is currently in the intervention delivery phase.
There has been an overall delay in the timeline of the study due to the COVID - 19 pandemic, lockdown and NHS staff shortage in our study wards.

Recruitment for the study commenced in March 2022 and was completed in Sep 2022. Following recruitment, we launched our first PLA meeting in Tower Hamlets in Oct 2022 and then in Newham in the week following. Waltham Forest dropped out of the study due to lack of supporting staff in the recruitment phase.

We have completed 5 out of the 8 PLA meetings and the PLA meeting cycle will conclude in Feb 2023. Following this we will initiate data analysis and reporting of study findings. We will collect follow-up data in April 2023 which will be the last data collection time point for this trial.

REFERENCES

[1] World Health Organization (WHO), Postnatal care for mothers and newborns: Highlights from the World Health Organization 2013 Guidelines. Available from: http://www.who.int/maternal_child_adolescent/publications/WHOMCA-PNC-2014-Briefer_

For peer review only - http://bmjopen.bmj.com/site/about/guidelines.xhtml

A, 2015. 4.

[2] Group, W.M.G.R.S., WHO Child Growth Standards based on length/height, weight and age. Actapaediatrica (Oslo, Norway: 1992). Supplement, 2006. 450: p. 76.

[3] Evandrou M, Falkingham J, Feng Z, et al Ethnic inequalities in limiting health and self-reported health in later life revisited J Epidemiol Community Health 2016;70:653-662.

[4] Brown MS. Religion and economic activity in the South Asian population. Ethnic and Racial Studies. 2000;23(6):1035-61

[5] ASQ-3. The Ages & Stages Questionnaires, Third Edition. [cited 2019 30 November]; Available from: https://agesandstages.com/products-pricing/asq3/.

[6] Lennox, A., et al., Diet and nutrition survey of infants and young children, 2011. A survey carried out on behalf of the Department of Health and Food Standards Agency, 2013.

[7] NATIONAL DIET AND NUTRITION SURVEY: Food and Drink Diary. 2010; Available from: https://webarchive.nationalarchives.gov.uk/20130413115649/http://media.dh.gov.uk/network/261/files/2012/07/Appendix-E_All-docs-combined-v2.pdf.

[8] 2011. Early Years Interventions To Address Health Inequalities In London – The Economic Case.[ebook] London: Greater London Authority. Available at:<https://www.london.gov.uk/sites/default/files/gla_migrate_files_destination/Early%20Years%20report%20OPT.pdf> [Accessed 5 January 2021].

[9] High S, Mckie I, Mackay M, et alO-09 Beyond traditional approaches to community engagement: using asset based community development to discover, connect and mobilise communities to support each other right to the end of lifeBMJ Supportive & Palliative Care 2022;12:A4.

[10] Lakhanpaul, M., 2019. PANCHSHEEEL - MRC HEEE STUDY. [online] Ucl.ac.uk. Available <https://www.ucl.ac.uk/child-health/sites/child-health/files/newsletter_3_0.pdf

[11] WHO. WHO recommendation on facilitated participatory learning and action cycles with women`s groups during pregnancy: The WHO Reproductive Health Library 2018 [from: https://extranet.who.int/rhl/topics/improving-health-system-performance/implementation-strategies/who-recommendation-facilitated-participatory-learning-and-action-cycles-womens-groups-during.

[12] Seward N, Neuman M, Colbourn T, Osrin D, Lewycka S, Azad K, et al. Effects of women's groups practising participatory learning and action on preventive and care-seeking

For peer review only

behaviours to reduce neonatal mortality: A meta-analysis of cluster-randomised trials. PLoS medicine. 2017;14(12):e1002467.

[13] Tripathy P, Nair N, Barnett S, Mahapatra R, Borghi J, Rath S, et al. Effect of a participatory intervention with women's groups on birth outcomes and maternal depression in Jharkhand and Orissa, India: a cluster-randomised controlled trial. The Lancet. 2010;375(9721):1182-92

[14] Prost A, Colbourn T, Seward N, Azad K, Coomarasamy A, Copas A, et al. Women's groups practising participatory learning and action to improve maternal and newborn health in low-resource settings: a systematic review and meta-analysis. The Lancet. 2013;381(9879):1736-46

[15] Mayor of London. London's socio-economic issues. 2016; Available at:https://www.london.gov.uk/sites/default/files/chapter10-economic-evidence-base-2016.pdf.Accessed 08/06/, 2020.

[16] ONS. 2011 Census Data. 2011; Available at: https://www.ons.gov.uk/census/2011census.

[17] Faculty of Dental Surgery. 2015. The state of children's oral health in England. [PDF]

[18] Newington L, Metcalfe A. Factors influencing recruitment to research: qualitative study of the experiences and perceptions of research teams. BMC Med Res Methodol. 2014;14:10. Published 2014 Jan 23. doi:10.1186/1471-2288-14-10

[19] Office for National Statistics. 2020. Domestic Abuse During The Coronavirus (COVID-19) Pandemic, England And Wales - Office For National Statistics. [online] Available at: <https://www.ons.gov.uk/peoplepopulationandcommunity/crimeandjustice/articles/domesticabuse duringthecoronaviruscovid19pandemicenglandandwales/november2020> [Accessed 10 January 2021].

[20] Joseph RP, Keller C, Ainsworth BE. Recruiting Participants into Pilot Trials: Techniques Researchers with Shoestring Budgets. Calif J Health Promot. 2016;14(2):81-89.

[21] An innovative approach to recruiting homebound older adults. Crawford Shearer NB, Fleury JD, Belyea MRes Gerontol Nurs. 2010 Jan; 3(1):11-8.

[22] Llewellyn, C.H., et al., Development and factor structure of the Baby Eating Behaviour Questionnaire in the Gemini birth cohort. Appetite, 2011. 57(2): p. 388-396.

[23] Daniels, L.A., et al., Outcomes of an early feeding practices intervention to prevent

For peer review only

childhood obesity. Pediatrics, 2013. 132(1): p. e109-e118.

[24] Fogel, A., et al., Prospective associations between parental feeding practices and children's oral processing behaviours. Maternal & child nutrition, 2019. 15(1): p. e12635.

[25] Lakhanpaul M, Benton L, Lloyd-Houldey O, et al. Nurture Early for Optimal Nutrition (NEON) programme: qualitative study of drivers of infant feeding and care practices in a British-Bangladeshi population. BMJ Open 2020;10:e035347. doi:10.1136/ bmjopen-2019-035347

[26] Tla.mpi.nl. 2020. ELAN | The Language Archive. [online] Available at: http://tla.mpi.nl/tools/tla-tools/elan

[27] Health Do. Infant feeding recommendation. Department of Health London, England; 2003.

[28] 2019. Dental Record Keeping Standards: a consensus approach. [PDF] NHS England and NHS Improvement.

[29] Faculty of General Dental Practice, 2012. 7. Collecting Data. Available at: <https://www.fgdp.org.uk/research-primary-dental-care/7-collecting-data> [Accessed April 15, 2021].

[30] Lawrence, C.E. et al. A REDCap-based model for electronic consent (eConsent): Moving toward a more personalized consent. Journal of Clinical and Translational Science. 2020; 4(4):345–353.

[31] Bugge C, et al. A process for Decision-making after Pilot and feasibility Trials (ADePT): Development following a feasibility study of a complex intervention for pelvic organ prolapse. Trials.2013 Oct 25;14(1):353.

[32] Health Research Authority. 2018. HRA And MHRA Publish Joint Statement On Seeking And Documenting Consent Using Electronic Methods (Econsent). [online] Available at: <https://s3.eu-west-2.amazonaws.com/www.hra.nhs.uk/media/documents/hra-mhra-econsent-statement-sept-18.pdf> [Accessed 8 January 2021]

For peer review only - http://bmjopen.bmj.com/site/about/guidelines.xhtml

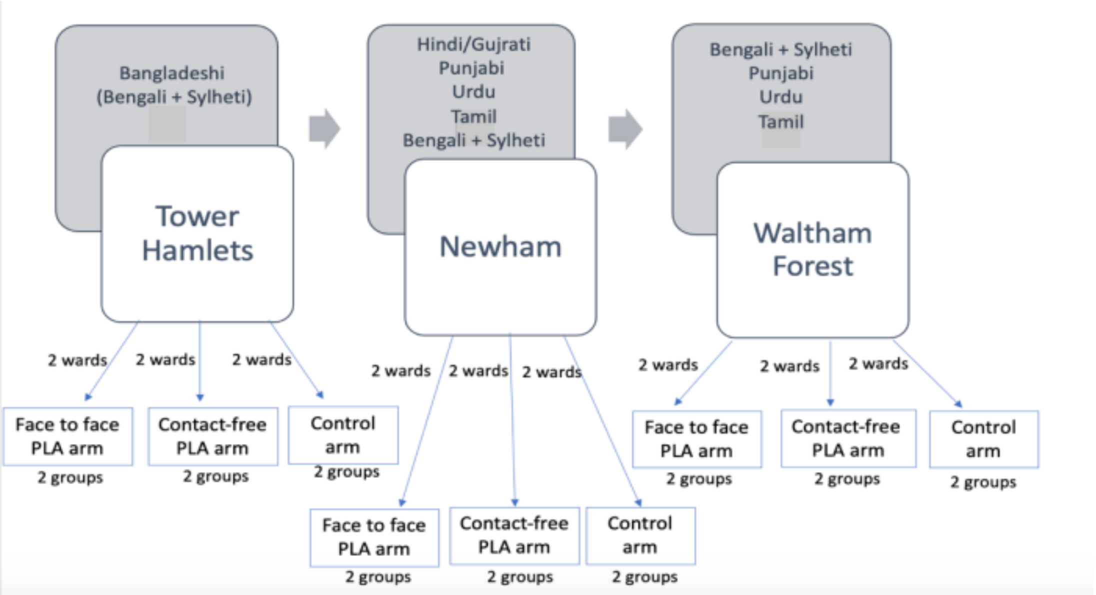

Figure 1. Overview schematic detailing number of wards & PLA groups by East London boroughs

Footnote: To illustrate, TH will have 2 face-to-face Bangladeshi/ Bengali & Sylheti PLA groups (1 per ward), 2 groups of Bangladeshi/ Bengali & Sylheti online PLA group (1 per ward), and 2 Bangladeshi/ Bengali & Sylheti control groups (1 per ward). The more the ethnic/ language group in the borough, the more the intervention and control groups.

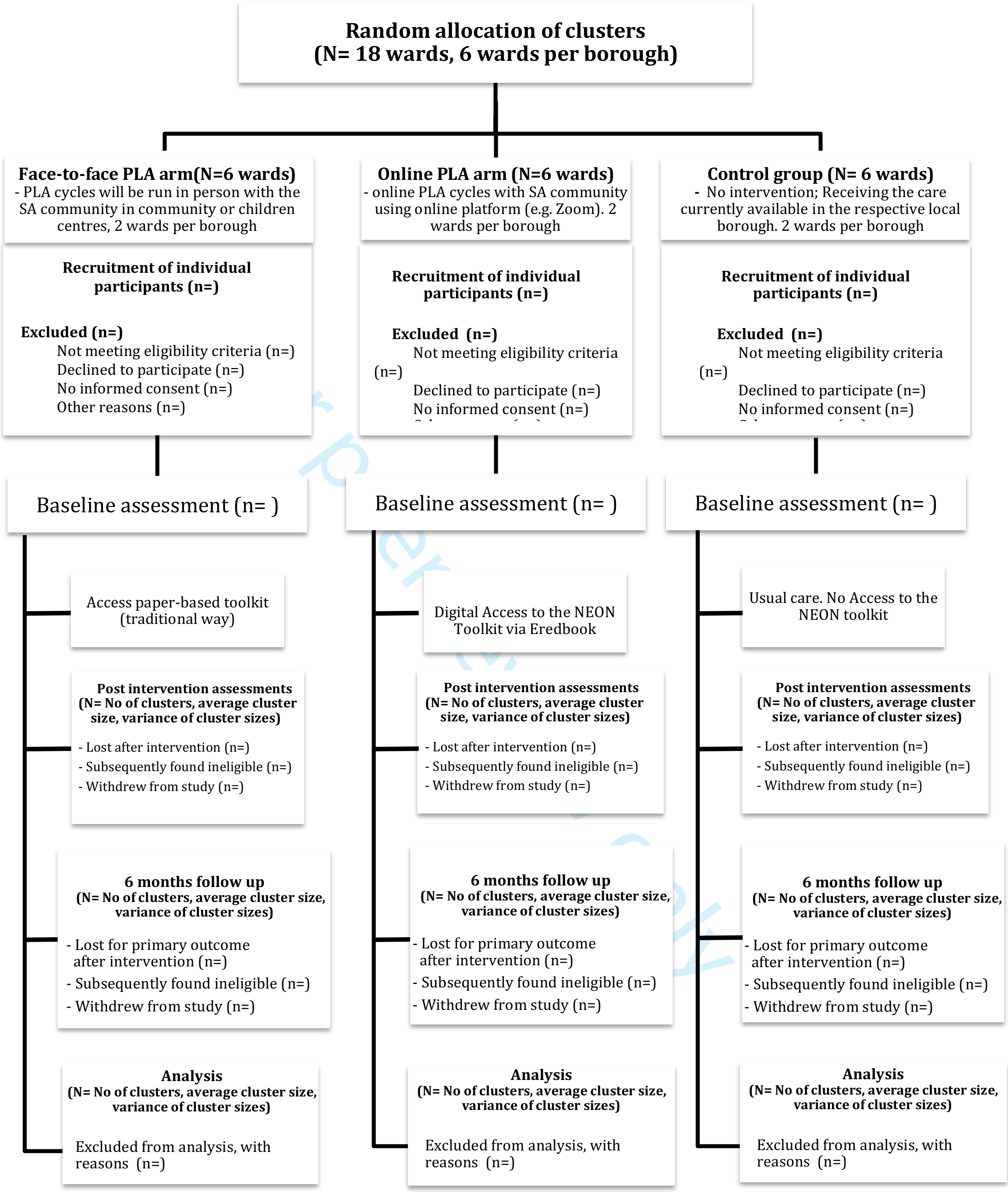

Figure 2. Overview of the NEON pilot feasibility RCT design and participant flow

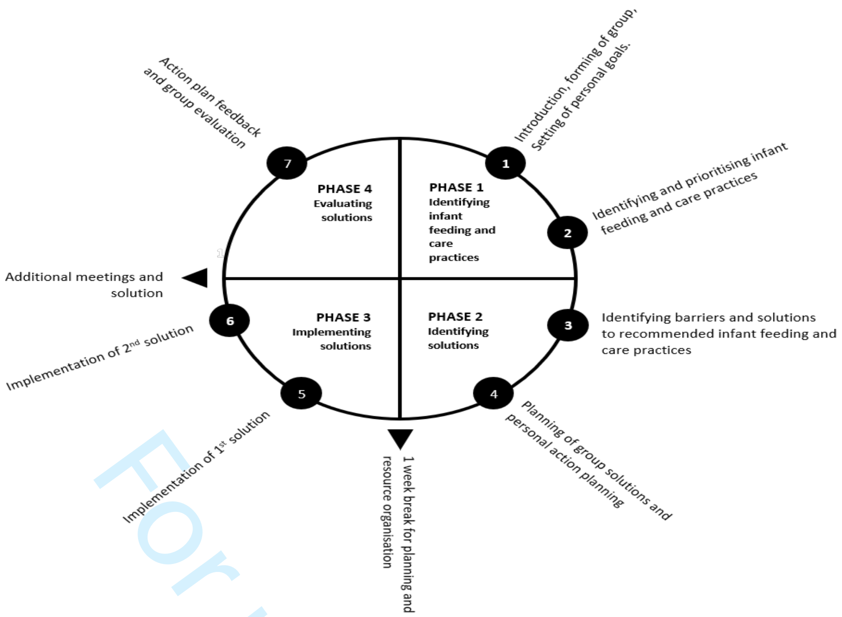

Figure 3. NEON Women's Group PLA Cycle

For peer review only

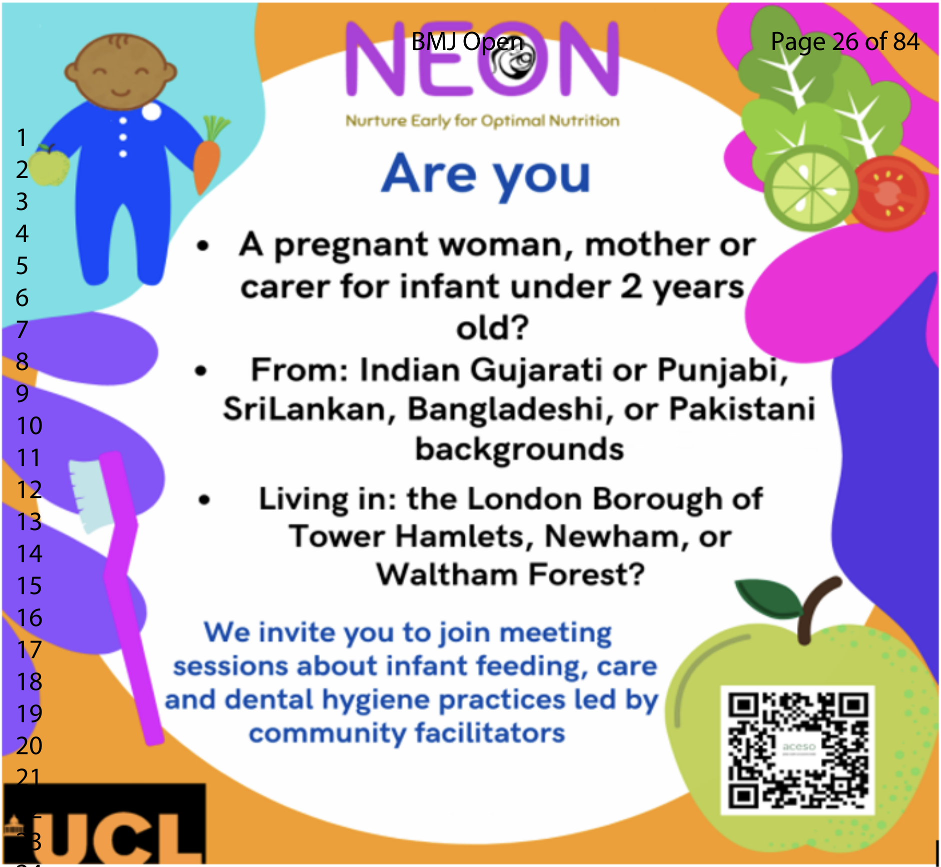
NEON
Nurture Early for Optimal Nutrition
Are you
• A pregnant woman, mother or carer for infant under 2 years old?
• From: Indian Gujarati or Punjabi, SriLankan, Bangladeshi, or Pakistani backgrounds
• Living in: the London Borough of Tower Hamlets, Newham, or Waltham Forest?
We invite you to join meeting sessions about infant feeding, care and dental hygiene practices led by community facilitators
UCL

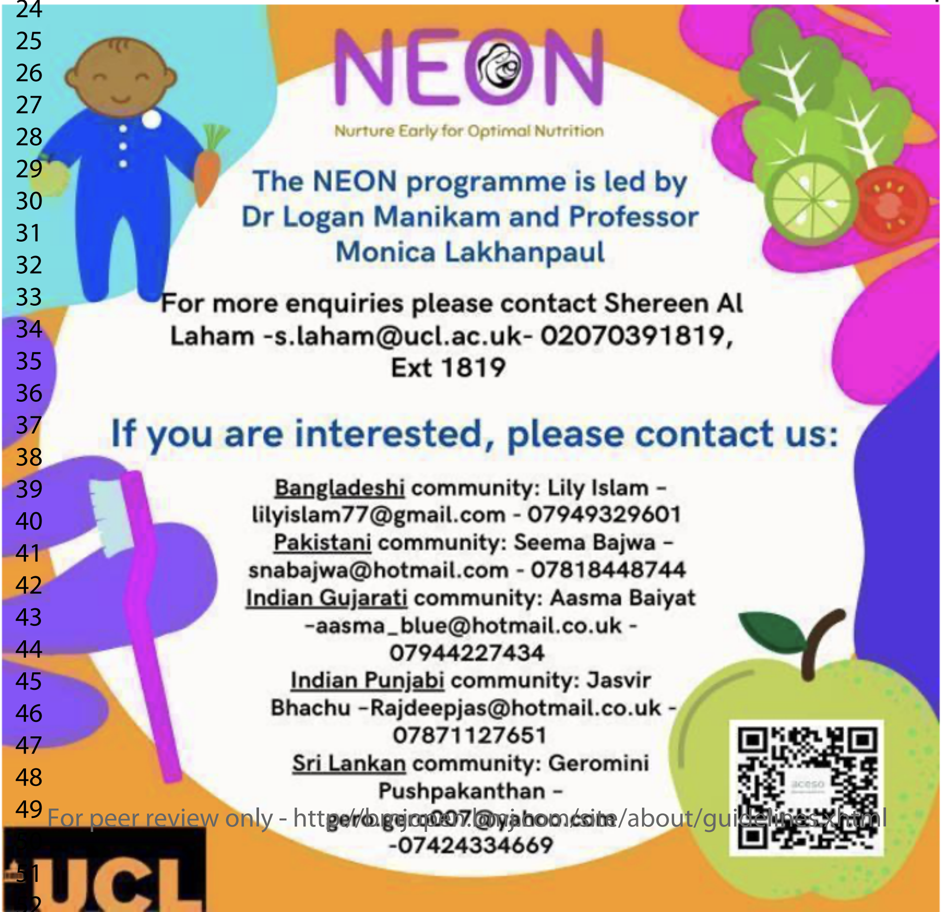
NEON
Nurture Early for Optimal Nutrition
The NEON programme is led by Dr Logan Manikam and Professor Monica Lakhanpaul
For more enquiries please contact Shereen Al Laham -s.laham@ucl.ac.uk- 02070391819, Ext 1819
If you are interested, please contact us:
Bangladeshi community: Lily Islam – lilyislam77@gmail.com - 07949329601
Pakistani community: Seema Bajwa – snabajwa@hotmail.com - 07818448744
Indian Gujarati community: Aasma Baiyat -aasma_blue@hotmail.co.uk - 07944227434
Indian Punjabi community: Jasvir Bhachu –Rajdeepjas@hotmail.co.uk - 07871127651
Sri Lankan community: Geromini Pushpakanthan –
-07424334669
UCL

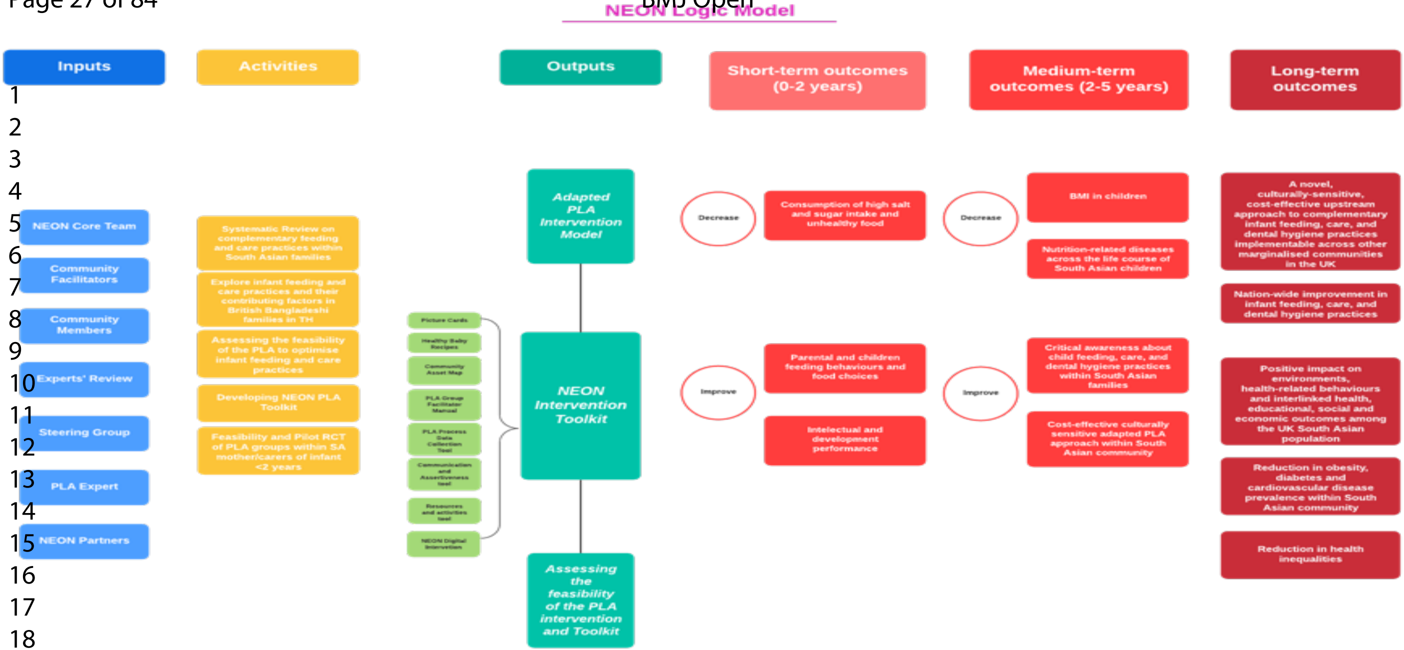
NEON Logic Model
Inputs
Activities
Outputs
Short-term outcomes (0-2 years)
Medium-term outcomes (2-5 years)
Long-term outcomes
NEON Core Team
Community Facilitators
Community Members
Experts' Review
Steering Group
PLA Expert
NEON Partners
Systematic Review on complementary feeding and care practices within South Asian families
Explore infant feeding and care practices and their contributing factors in British Bangladeshi families in TH
Assessing the feasibility of the PLA to optimise infant feeding and care practices
Developing NEON PLA Toolkit
Feasibility and Pilot RCT of PLA groups within SA mother/carers of infant <2 years
Picture Cards
Healthy Baby Recipes
Community Asset Map
PLA Group Facilitator Manual
PLA Process Data Collection Tool
Communication and Assertiveness tool
Resources and activities tool
NEON Digital Intervention
Adapted PLA Intervention Model
NEON Intervention Toolkit
Assessing the feasibility of the PLA intervention and Toolkit
Decrease
Consumption of high salt and sugar intake and unhealthy food
Improve
Parental and children feeding behaviours and food choices
Intellectual and development performance
Decrease
BMI in children
Nutrition-related diseases across the life course of South Asian children
Improve
Critical awareness about child feeding, care, and dental hygiene practices within South Asian families
Cost-effective culturally sensitive adapted PLA approach within South Asian community
A novel, culturally-sensitive, cost-effective upstream approach to complementary infant feeding, care, and dental hygiene practices implementable across other marginalised communities in the UK
Nation-wide improvement in infant feeding, care, and dental hygiene practices
Positive impact on environments, health-related behaviours and interlinked health, educational, social and economic outcomes among the UK South Asian population
Reduction in obesity, diabetes and cardiovascular disease prevalence within South Asian community
Reduction in health inequalities

RESOURCES AND SERVICES SUPPORTING INFANT FEEDING, CARE AND DENTAL HYGIENE

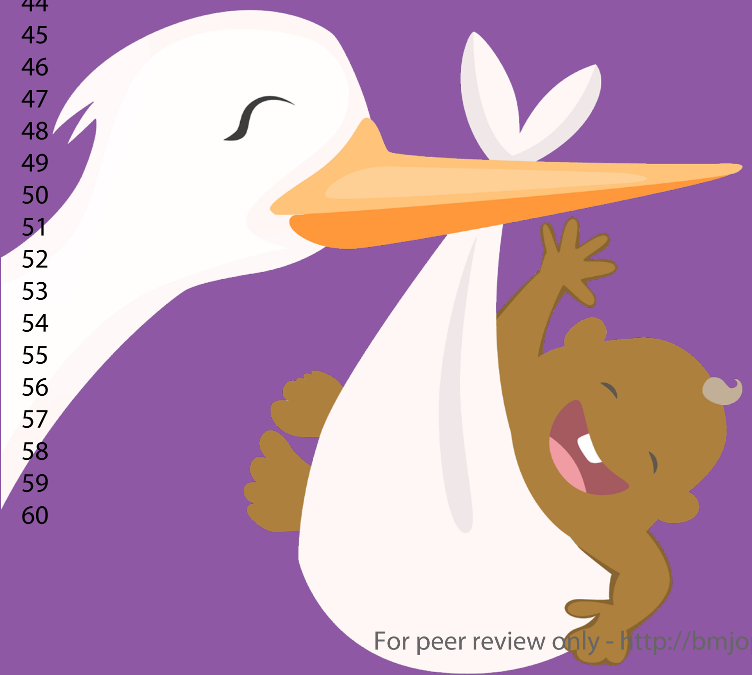

NEON

Nurture Early for Optimal Nutrition

NHS AND COUNCILS ADVICE AND SUPPORT

1. NHS STAR4LIFE

https://www.nhs.uk/start4life

Website provides trusted NHS help and advice for parents and carers during pregnency, and birth and the early years.

2. NHS CHOICES

NHS Choices (www.nhs.uk) is the official website of the National Health Service in England and provides comprehensive health information service with thousands of articles, videos and tools. Key pages which give guidance on infant feeding and care include:

- **Your newborn:** *https://www.nhs.uk/conditions/pregnancy-and-baby/childrens-meal-ideas/?tab-name=your-newborn*
- **Babies and toddlers** *https://www.nhs.uk/conditions/pregnancy-and-baby/childrens-meal-ideas/?tabname=ba-bies-and-toddlers*

3.CHILDREN CENTRES AND LIBRARIES

Tower Hamlets has 12 children centres across the borough providing a range of services to infants, children and families to give them the support they need to be safe, healthy and happy, so that they are able to reach their full potential. This web page on the **Tower Hamlets Council** website gives locations and contact details of all the children's centres in the borough: *https://www.towerhamlets.gov.uk/lgnl/education_and_learning/childcare_and_early_years_educ/Children_centres/childrens_centres.aspx*

The borough of Newham offers support to its residents with children under the age of 5 through **Best Start in Life (BSiL)** Children's centres. These include play activities and childminder services, as well as supporting parents to improve their confidence, support their children with healthy eating and weaning. Libraries offer free courses and internet services for parents, and they offer devices and digital equipment to borrow.

Locations and details can be found on the Families Newham website: *https://families.newham.gov.uk/kb5/newham/directory/family.page?familychannel=3-2*

Waltham Forest offers to its families a similar service, with support for healthy development and access to financial support. They have implemented the 2 Year Old Partnership Pathway in which professionals empower and inform parents to assure the best start in life for their children. They offer advice on a wide range of issues including health, finance and cognitive/motor development. **Waltham Forest** offers support directed at healthy weight and eating through HARRY.

More information can be accessed via the following link: *https://www.walthamforest.gov.uk/content/2yo-partnership-pathway*

Further informal support that does not require formal registration can generally be accessed in public libraries

For peer review only

GUIDENCE DOCUMENTS, ONLINE RESOURCES AND APP'S

1. BABY FRIENDLY INITIATIVE

Published by the Department of Health and the Baby Friendly Initiative, the leaflets below provide mothers and health professionals with key information about breast feeding, introducing solid foods to babies, and evidence-based guidance around formula. The **Baby Friendly Initiative** is part of a global partnership between the World Health Organization (WHO) and UNICEF. The guidance below should be prioritised over other online resources (other than NHS Choices) as they the baby friendly initiative is less influenced by commercial interests, which can sometimes influence the advice offered to mothers and parents regarding infant feeding and care.

- **Leaflet on weaning and starting solid food:**
  https://www.unicef.org.uk/babyfriendly/baby-friendly-resources/leaflets-and-posters/weaning-starting-solid-food/

- **Leaflet on breastfeeding:**
  https://www.unicef.org.uk/babyfriendly/baby-friendly-resources/leaflets-and-posters/off-to-the-beststart/ -

- **Leaflet on infant formula:**
  https://www.unicef.org.uk/babyfriendly/baby-friendly-resources/leaflets-and-posters/simple-formula-guide-for-parents/

- **Leaflet on bottle feeding:**
  https://www.unicef.org.uk/babyfriendly/baby-friendly-resources/leaflets-and-posters/guide-to-bottle-feeding/

- **Baby friendly initiative resources in Bengali:**
  https://www.unicef.org.uk/babyfriendly/baby-friendly-resources/leaflets-and-posters/foreign-language-resources/bengali-resources/

- **Baby friendly initiative resources in Hindi:** बच्चे के अनुकूल पहल संसाधन
  https://www.unicef.org.uk/babyfriendly/baby-friendly-resources/foreign-language-resources/hindi-resources/

- **Baby friendly Initiative resources in Urdu:** ل ئ اس و ے ک (ت ش اده گ ن) ء ام ن وش ن ئ ادت ب ا ی ک ے چ ب
  https://www.unicef.org.uk/babyfriendly/baby-friendly-resources/foreign-language-resources/urdu-resources/

Other Baby Friendly Initiative resources/leaflets:

https://www.unicef.org.uk/babyfriendly/baby-friendly-resources/leaflets-and-posters/

2. FIRST STEPS NUTRITION TRUST

https://www.firststepsnutrition.org

First steps nutrition can also be used as a trusted source of good information and guidance regarding nutrition and feeding in pregnancy, infants, and early years. **First Steps Nutrition Trust** is funded by research or charitable grants and donations, and does not take money from any commercial organisation or any organisation or individual linked to the sales of infant formula and other food or drink product or service which is associated with poor nutritional health of women and children.

For peer review only

First Steps Nutrition Trust offers some useful resources on meal choices and portion sizes, with helpful diagrams to illustrate portion size. Remember! Portion size is a maximum, not a minimum, never encourage a child to eat more when they indicate they have had enough.

- Eating well: the first year – guidance on introducing foods, food choices and meals for up to 7 months, 7-9 months, and 10-12 months: http://www.firststepsnutrition.org/newpages/Infants/first_year_of_life.html
- Eating well: Early years – guidance on food choices, meals, snacks and portion size http://www.firststepsnutrition.org/newpages/Early_Years/eating_well-early_years.html

3. BORN TO MOVE APP

Born to move is a free NHS app to help parents or carers with their newborn babies right up to pre-school, specifically relating to physical activity and play. It's packed with advice, tips and games that you can play with your child to support their development.

https://itunes.apple.com/gb/app/born-to-move/id976095022?mt=8Local%20initiatives/projects

https://play.google.com/store/apps/details?id=com.project.Born&hl=en_GB

4. EREDBOOK

ERedbook is an app based digital version of the paper Redbook. It provides solutions for parents that allows them to review their child's NHS records whilst providing health information and guidance from NHS and other sources. Additionally, it contains personalised information for new parents, including relevant videos and information from local sources & reminders and appointments updates. Parents can review the NHS child's health records on the app when they connect record to NHS. Also, The NEON participants will be able to review the NEON toolkit and receive correct information via the ERedbook app at any time and place.

https://www.eredbook.org.uk

https://apps.apple.com/gb/app/eredbook/id606443658

https://play.google.com/store/apps/details?id=com.sitekit.eRedBook&hl=en_GB

OTHER SOLUTION IDEAS TO SUPPORT PLAY AND ACTIVITY:

1. Develop activity and play schedules for you and your infant with guidance from our local children centre or health visitor and make time for these activities.

2. Download the 'born to move' app for access to information and guidance on infant activity and play.

3. Designate a play area (even if this needs to be set-up and put away at times of day) for your infant in the home.

4. Arrange play and physical activity sessions together as a group, or with other parents, extended family, carers and infants – this could help with support and confidence in exercising and conducting physical activity in public spaces.

For peer review only

OTHER LOCAL INITIATIVES, CHARITIES AND PROJECTS IN EAST LONDON

1. BREASTFEEDING NETWORK

Find your nearest breast feeding drop-in-group in Tower Hamlets
https://www.breastfeedingnetwork.org.uk

There are groups run at Shadwell Children's Centre, Chrisp Children's Centre, Overland Children's Centre, Collingwood Children's Centre, Wapping Children's Centre and many more. If you need information or support with breastfeeding in the evenings or at weekends please call:

National Breastfeeding Helpline: *0300 1000 212*

BfN Support in Bengali/Sylheti: *0300 456 2421*

Open from *9.30am - 9.30pm 365 days a year*

Newham: https://www.nct.org.uk/local-activities-meet-ups/region-london/newham/breastfeeding-support

Tower Hamlets: https://www.breastfeedingnetwork.org.uk/tower-hamlets/

Waltham Forest offers a team of infant feeding practitioners delivering 1:1 sessions, a helpline and at home visitors to support the breastfeeding family through HENRY.

For information:
Email: wfsupport@henry.org.uk
Telephone: 020 8496 5223

2. DENTAL SERVICES AND RESOURCES

Dentists across Tower Hamlets, Waltham Forest, and Newham offer free dental check-ups, which you can attend as soon as your child's teeth first appear.

To find your nearest dental practice in Tower Hamlets, please call 020 7364 5000.

HENRY has put together some tips for healthy teeth in young children and a list of Waltham Forest dentists currently accepting new patients from birth: https://www.henry.org.uk/sites/www.henry.org.uk/files/inline-files/Healthy%20Teeth%20Leaflet%20%28April%29_0.pdf

The Kent Community Health NHS Foundation Trust has compiled some online oral health promotion resources for parents here: https://www.kentcht.nhs.uk/service/dental-services/oral-health-promotion-resources/

The Kent Community Health NHS Foundation Trust has compiled some online oral health promotion resources for parents here: https://www.kentcht.nhs.uk/service/dental-services/oral-health-promotion-resources/

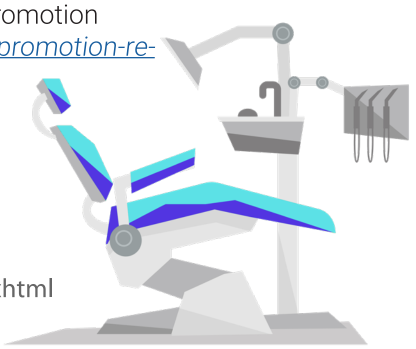

For peer review only

Here are examples of dental services by borough (to find out more, please see the community asset map):

Tower Hamlets

- Fresh Springs Dental Practice
  T: 020 3216 0077
  E: reception@freshspringsdentalpractice.co.uk

- Together Dental Whitechapel
  T: 020 7247 4600
  E: whitechapelreception@together.dental

Waltham Forest

- Abbey Dental Practice
  T: 020 8521 2816
  E: info@abbeydentalwalthamstow.co.uk

- Inspire Dental Walthamstow
  T: 020 8521 6656
  E: info@inspiredentalwalthamstow.co.uk

3. MATERNITY MATES

http://www.whfs.org.uk/index.php/what-we-do/maternity-mates

Maternity Mates™ is a project run by the charity Women's Health and Family Services which recruits, trains and match-ups volunteer Maternity Mates with pregnant women in need of extra support in Tower Hamlets. A Maternity Mate is a female volunteer trained by WHFS to provide practical and emotional support to women during pregnancy, childbirth and the early weeks of motherhood. Support can start any time from the 5th month of pregnancy, through to birth and up to either 6 or 12 weeks after the baby is born (6 weeks in Tower Hamlets and 12 weeks in Newham). **Maternity Mates** are recruited from the communities they serve, and where possible, will speak the same language as the mother-to-be.

For further information, please contact:

Jasmin Begum - Programme Manager
M: *07960 327 760*
T: *020 7377 9640*
E: *jasmin.begum@whfs.org.uk*

For information about the project in Tower Hamlets and Waltham Forest, please contact:

Rubi Rodriguez - Project Co-ordinator Tower Hamlets and Waltham Forest
M: 07496 764 881
E: rubi.rodriguez@whfs.org.uk

For information about the project in Newham, please contact:

Irantzu Perez Arribas - Project Co-ordinator Newham
M: 07436 139 235
E: irantzu.perez@whfs.org.uk

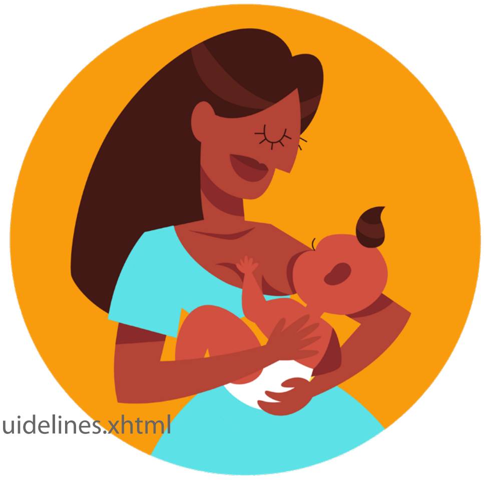

For peer review only

4. BOUNTY

https://www.bounty.com/about-bounty/bounty-packs/newborn-pack

Bounty provides mothers and pregnant women with free subscription packs including advice from Public Health England, as well as a variety of sample sized baby products (nappies, baby formula etc.) and coupons.

Every mother with a Newborn will receive a Newborn Pack at the hospital after delivery, and more freebies and advice are available through the Bounty app.

5. EMMA'S DIARY

https://www.emmasdiary.co.uk
Contact: 01628 535 483 Mon-Fri 9am to 4:30pm

Emma's Diary is a free to access resource created by a board of GPs and midwives that provide information ranging from trying to get pregnant from wellbeing information during and post-pregnancy. There's a free to download mobile app to allow you to access information more easily, tailored towards recent mothers and baby related advice and offers.

6. THE TOYHOUSE CENTRE

http://www.toyhouse.org.uk/

Phone: 0020 7987 7399
E-mail: info@toyhouse.org.uk

Toy House is a local charity in Tower Hamlets which runs projects and services aimed at support play and wellbeing of infants, children and families.

Toy Libraries
http://www.toyhouse.org.uk/toy-libraries/
The charity has its own toy library, and has information and links to over 19 toy libraries and stay and play sessions run across Tower Hamlets.

7.MEND - MIND, EXERCISE, NUTRITION...DO IT!

https://www.mytimeactive.co.uk/mend

MEND is a programme which empowers mums, children and adults to become fitter, healthier and happier and to reach or maintain a healthier weight. It's free to attend and run by local teams in local communities, after school or at weekends. MEND have a range of programmes for different ages run in different boroughs across London.

MEND Mums is designed for post-natal women with a baby up to 2 years and a BMI of 25 or above, **MEND** Mums is a fun and interactive weight management programme incorporating energy boosting workouts and great nutrition tips to help establish healthy habits for life.

For more information about any of the MEND programmes in Tower Hamlets call:
020 8323 1725

For peer review only

8. MIND •

MIND is a mental health charity with a specific branch in Newham and Tower Hamlets that offers free mental health services talking therapies, COVID19 Support and Advocacy services

https://www.mithn.org.uk
Phone: 020 7510 1081
Email: info@mithn.org.uk

9. REBECCA CHEETHAM NURSERY & CHILDREN'S CENTRE • • • •

Website: https://www.rebeccacheetham.newham.sch.uk
Phone:
Email:

The Rebecca Cheetham Children's Centre offers free child-minding services as well as advice and support for families with young children. However they have temporarily stopped face to face sessions in light of the COVID pandemic. Limited online services are still running for examples: a Family Support Team that can refer you to foodbanks.

OTHER IDEAS AND SUGGESTIONS • • • • • • • • • • • • • • •

- o **Breakfast clubs** – these are available in most schools to give children a healthy start to the day. Speak to your local school. Due to COVID19 Restrictions, these breakfast clubs have moved to.
- o Despite this, virtual coffee mornings are ongoing

- o **Social support** – if you feel you need more support from other mothers, parents or people local to you, you can attend local coffee mornings, toddler groups and parent forums. E.g. One stop shop in Newham and Tower Hamlets. Father examples would be the "idea Store" which is based in Tower Hamlets and offers a range of free activities and educational services for children and families alike. http://www.healthwatchnewham.co.uk/sites/default/files/newham_domestic_abuse_one-stop-shop.pdf

- o **Triple P – Positive Parenting Programme** – is an evidence based programme aimed at supporting parents develop relationships with their children.

  - Newham offers a Parenting Befriending service and bookable workshops accessible via the following website: https://families.newham.gov.uk/kb5/newham/directory/family.page?family-channel=1-1

  - Tower Hamlets offers a similar service: https://www.towerhamlets.gov.uk/lgnl/educa tion_and_learning/parental_support/parenting_programmes.aspx
    parenting@towerhamlets.gov.uk.

  - Waltham Forest offers a series of 6 sessions for parents with children aged 3 and over. https://www.walthamforest.gov.uk/content/parenting-support

    Call: *020 8496 2442*
    Email: *earlyhelpparenting@walthamforest.gov.uk*

- o **Tunmarsh Centre**– support for parents with children with special needs
  The New Tunmarsh Centre based in Newham provides free education for children with ASD / DHD and support for their families. Parents are regularly invited for coffee mornings with speakers.

  E-mail: Faz.Mac@gmail.com

For peer review only

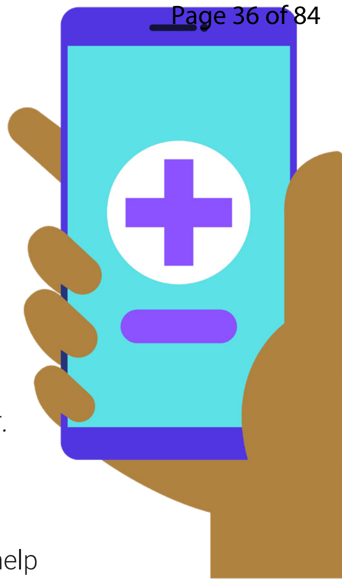

- **John Smith Children's Centre**

  Telephone: 0207 364 0537
  Contact: Deborah Wooding
  Email: deborah.wooding@towerhamlets.gov.uk
  Based in Tower Hamlets, The John Smith Children's Centre that offer childcare support.

- **The Trinity Centre**
  Website: www.thetrinitycentre.org/home/
  The Trinity Centre offers a day care nursery as well as independent groups and faith groups to help support parents form minority and marginalised communities

- **Around Poplar Children's Centre**

  Contact: Brenda Pascal
  T: 020 7364 0540
  E: Brenda.pascal@towerhamlets.gov.uk

  The Around Poplar Children's Centre offers childcare, family, health services, and early education support for families with children under 5 free of charge.

  For a list of similar children's centres in Tower Hamlets, see here:
  https://www.localoffertowerhamlets.co.uk/organisations?utf8=%E2%9C%93&search_organisation%5Bterm%5D=&search_organisation%5Blocation%5D=&search_organisation%5Blatitude%5D=&search_organisation%5Blongitude%5D=&search_organisation%5Bcategory_ids%5D%5B%5D=3308

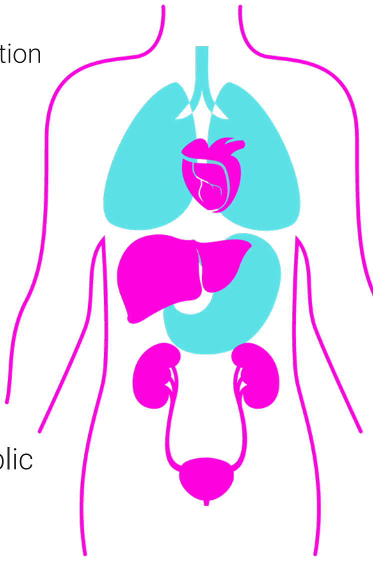

- **Idea Store**

  Website: www.ideastore.co.uk
  Branches of the Idea Store in Tower Hamlets offer library services and access to public computers for families and individuals free of charge.

- **Outreach centre** Outreach centres are open till 6 pm where you can drop your children there, and it is free for people who are entitled for public funds. They offer: Child minding, after school club, breakfast club for parents and children <3 years. Key examples would be Community Centres and Children's Centres.

EXAMPLES:

Newham Council: https://www.newham.gov.uk/homepage/115/community-centres
Tower Hamlets: Community and day centres (towerhamlets.gov.uk)

Plaistow Children's Centre
Website: https://plaistow.newham.sch.uk/Plaistow-Children-s-Centre/

The Plaistow Children's Centre is based in Newham and offers care and support for young children ranging from 0-5 years for 49 weeks within the year led by staff and volunteers. Other services range from nutrition webinars to overall family support and advice. The Children's Centre is still open during the pandemic and offers slightly modified services as a result.

For peer review only

OTHER SUPPORT

1. Request interpretation support for GP visits from Tower Hamlets GP care group.
2. Advocacy & Interpreting Service in Newham (https://www.gpcaregroup.org/section/455/Services/page/eb745e07-85ab-4cee-bc8f-c880a-d400a95/Advocacy-Interpreting)
3. Provide feedback on services or lodge a complaint via Health Watch Tower Hamlets. More information can be accessed here: https://www.healthwatchtowerhamlets.co.uk/

FOOD BANKS:

Suggested Food Bank locations: https://www.google.com/maps/d/edit?mid=1ZyqIE2-HgdQ-

NEON Community Asset Map: https://www.google.com/maps/d/edit?mid=1ZyqIE2-Hg-dQ-Hzow7KwhAiYsdtQnP_Zq&usp=sharing

- **Newham Food Alliance**

  Website: https://www.newham.gov.uk/newhamfoodalliance

  The Newham Food Alliance is a food bank based in Newham that can offer access to food for those who are struggling financially. There is also delivery support they can provide to those self-isolating due to COVID and providing food remotely during the pandemic regardless.

- **Church Food Banks:**

  Website: www.churchesfoodbank.org.uk/

  Food Banks located in the Borough of Newham. To access these you need to be referred from another agency such as a school, advice centres or your GP.

The 2 main sites are:

Woodgrange Baptist Church

345 Romford Road,Forest Gate,E7 8AA

0208 555 9880

St Paul's Church

65 Maryland Road,Stratford,E15 1JL

0208 534 1164

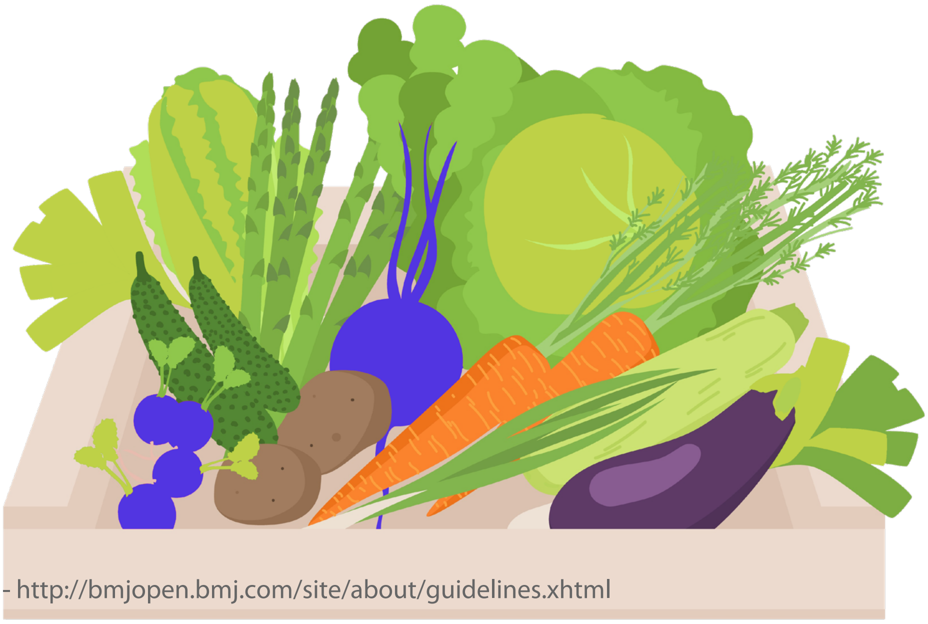

For peer review only

- **The Trussell Trust**

  Website: https://www.trusselltrust.org

  The Trussell Trust is a charity that provides nationwide network of food banks as well as emergency food for those in need. They also run regular campaigns to further increase support for people who need access to regular foods but cannot afford to do so. The website is useful as it flag foodbanks that are local to you.

- **First Love Foundation Foodbank**

  Website: www.firstlovefoundation.org.uk/

  The First Love Foundation coordinates the Tower Hamlets Foodbank. It provides daily necessities to families in need and alongside this, operates as a crisis service.

- **Eat or Heat - Waltham Forest Food Bank**

  Website: http://eatorheat.org

  Phone Number: 0800 772 0212

  Email: referrals@eatorheat.org

  A charity based in East London that supports local families. On the website, different professionals who can refer you to this service are listed.

- **Rukhsana Khan Foundation**

  Website: https://www.rukhsanakhanfoundation.org

  Phone Number: 07939 232 123

  Email: rukhsanakhanfoundation@outlook.com

  Rukshana Khan Foundation is a local charity that supports families in East London. They run food parcels and deliveries for those on benefits and those referred from Citizens Advice, NHS, Local Doctor's Surgery, Job Centre, Baby Food Bank, or the Council. They are also supporting those classed as clinically vulnerable. You can register for support on their website.

  They run a foodbank on Saturdays 11am-12pm at the William Morris Community Centre.

- **Waltham Forest Liberal Democrats**

  Website: https://www.walthamforestlibdems.org/local_support_for_families_in_need

  The Waltham Forest Liberal Democrats have collated a set of resources highlighting local foodbanks with Waltham Forest that local families can access

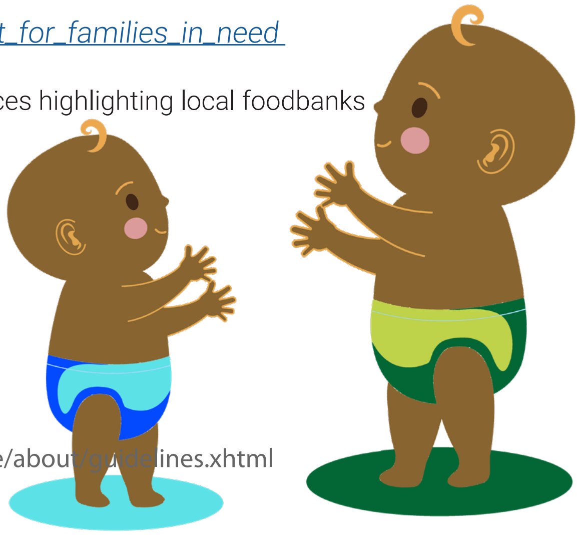

For peer review only

- **Eden Girls' School, Waltham Forest**

  A local food bank based at Eden Girls' School that provides food to local people from 1-3pm every Friday

  Contact Details:

  Eden Girls' School, Waltham Forest

  Silver Birch and Landmark Houses,

  Blackhorse Lane, Walthamstow

  London, E17 5SD

  Tel: 0208 523 1810

  Email: info@egwf.staracademies.org

- **Community Food Projects in Tower Hamlets**

  https://www.wen.org.uk/2020/03/30/https-www-wen-org-uk-2020-03-30-information-on-local-response-to-covid-19/

- **Healthwatch groups available in different boroughs:**

  Newham http://www.healthwatchnewham.co.uk/

  Tower Hamlets https://www.healthwatchtowerhamlets.co.uk/

  Redbridge http://www.healthwatchredbridge.co.uk/

  Waltham Forest https://www.healthwatchwalthamforest.co.uk/

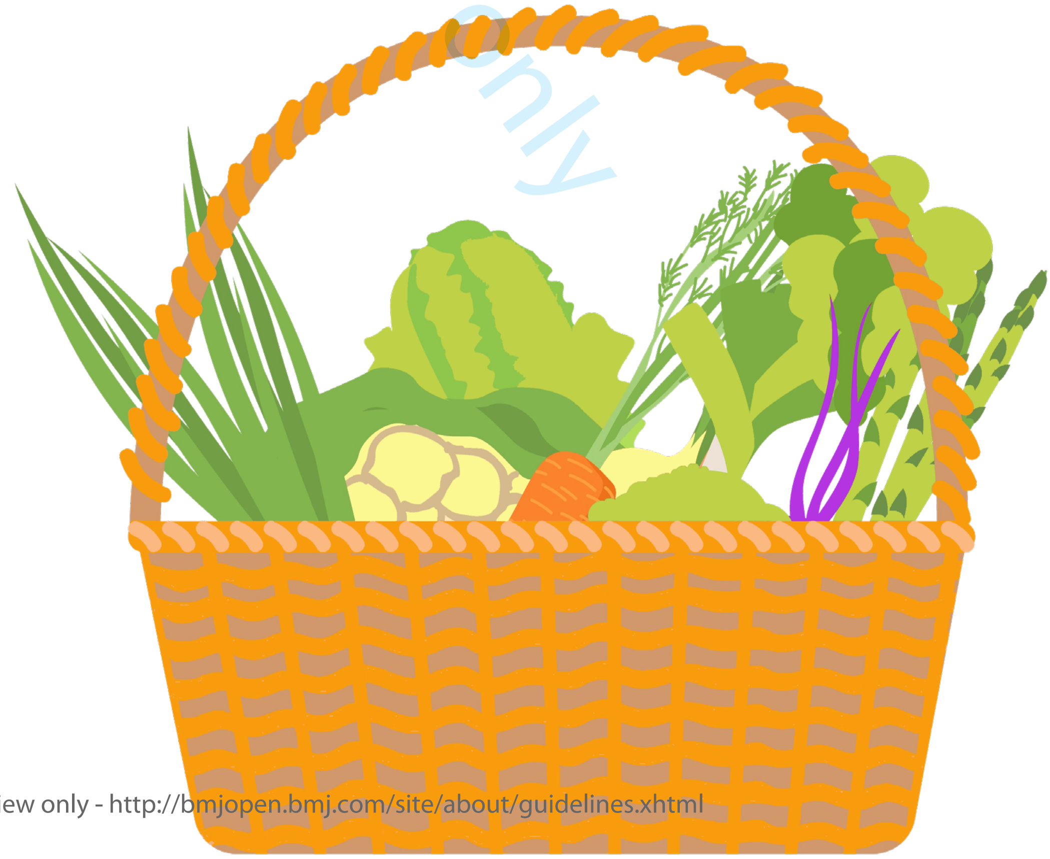

For peer review only

Participants Feedback Questionnaire

1. What were your reasons for wanting to attend the group meetings?

2. What made you come back after attending a meeting? /or, what was your reason for not coming back to later meetings?

i. *What made you keep coming back to the meetings? /Or, what could we have done differently to motivate you to come back to the next meeting?*

3. Did the group manage to meet your expectations?

i. *how did it meet your expectations?*
ii. *If it did not, why was this the case?*

4. What could be improved if this project was run again?

i. *How could we improve the content (meeting exercises)*
ii. *How could we improve the tools (picture cards / infant feeding resources)*
iii. *How could we improve the tailoring to your needs as British Bangladeshi mother/pregnant women/or grandmother*
iv. *Would it be beneficial to have picture cards and other project materials written in Bengali (with the picture on the front and Bengali text on the back)*
v. *Is there any additional information or resources you would have liked to have been offered in the meetings?*

5. How involved did you feel in the learning process of the group?

i. Did you feel you worked together as a group during the meetings? How so?
ii. Were there any barriers you experienced to participating?
iii. Were you given opportunities to share experiences? Please give examples.

6. Are you in contact with any of the other participants outside of the group meetings?

i. Have you discussed any issues raised in the group meetings?
ii. Have you supported each other on any issues raised in the group meeting?

9. Have you found new people, groups or sources of information as a result of attending the group meetings? Please give examples.

10. Did you share any information from the group meetings with any other members of your family or community outside of the group?

11. If we were to implement a full project using this approach, the next stage would involve more group meetings (possibly up to 8 additional meetings) where by the group would support each other to implement some of the solutions you identified and then after implementing

For peer review only - http://bmjopen.bmj.com/site/about/guidelines.xhtml

solutions, evaluate their success. Do you think this is something you or Bangladeshi carers would be willing to undertake or partake in?

i. *Can you think of any barriers to using this approach we need to consider at this stage?*
ii. *How could additional meetings best support you to implement the solutions you identified, and introduce the recommended infant feeding and care practices? What should they focus on?*
iii. *What additional meetings would be acceptable to you and feasible to attend?*

12. Is there anything else you would like to share about your experience on the project?

For peer review only

For peer review only - http://bmjopen.bmj.com/site/about/guidelines.xhtml

Facilitator Report Form

<table>
<tr><th colspan="6">PLA GROUP FACILITATOR REPORT - PLA group meeting xx</th></tr>
<tr><td>Group location/name (XX children's centre group)</td><td></td><td rowspan="2">Meeting number</td><td rowspan="2"></td><td rowspan="2">Me
etin
g
dat
e</td><td rowspan="2"></td></tr>
<tr><td>Facilitator name</td><td></td></tr>
<tr><td>Number of picture cards discussed</td><td colspan="5"></td></tr>
<tr><td>Did you manage to go through all questions on the picture cards?

• What does the picture show?

• Clarify which practice is being discussed

• Ask and explain how this affects infant growth and development

• Use info on the back of the card to ask questions about the practice and probe

• Ask if this occurs in the community? Is it common?

Were there any barriers to a full discussion on the above questions?</td><td colspan="5"></td></tr>
<tr><td>Were there any picture cards not discussed at all?</td><td colspan="5"></td></tr>
</table>

For peer review only

Which exercises were completed during your meeting?	
What was the outcome of each exercise?	
How long did the meeting run for?	
Did anything go well/did anything not go well?	
GROUP FACILITATOR TO COMPLETE AFTER EACH MEETING CONDUCTED.	

**PLA GROUP FACILITATOR REPORT – PLA group meeting 3**					
Group location/name (XX children's centre group)		Meeting number		Meeting date	
Facilitator name					
Number and type of picture cards discussed					
Did you manage to go through all the probing questions on the picture cards?<br>● *What does the picture show?*<br>● *Clarify what barrier is being discussed*<br>● *Ask and explain how this barrier may affect or influence behaviour relating to infant feeding and care*					

peer review only

• *Ask which infant feeding and care practice the barrier may prevent parents from practicing?*<br><br>• *Ask if the barrier exists in the community*<br>Were there any barriers to a full discussion on the above questions?	
Were there any picture cards not discussed at all?	
Which exercises were completed during your meeting?	
What was the outcome of each exercise?<br><br>Which barrier cards were identified for prioritised practice 1?<br><br>Which barrier cards were identified for prioritised practice 2?<br><br>Which barrier cards were identified for prioritised practice 3?	
How long did the meeting run for?	
Did anything go well/did anything not go well?	
GROUP FACILITATOR TO COMPLETE AFTER EACH MEETING CONDUCTED.	

For peer review only - http://bmjopen.bmj.com/site/about/guidelines.xhtml

PLA GROUP FACILITATOR REPORT – PLA group meeting 4					
Group location/name (XX children's centre group)		Meeting number		Me etin g dat e	
Facilitator name					
Number and type of picture cards discussed					
Did you manage to go through all the probing questions on the picture cards?<br>● *What does the picture show?*<br>● *Ask participants to identify what solution they are discussing and what non-recommended infant feeding and care practice the solution helps to reduce or manage.*<br>● *Ask participants to suggest ideas on what activities the group (or they, as individuals) could undertake to achieve this solution. Use the information on the back of the card to suggest ideas on activities which could be undertaken. Ask if the barrier exists in the community*<br>● Were there any barriers to a full discussion on the above questions?					
Were there any picture cards not discussed at all?					
Which exercises were completed during your meeting?					

For peer review only

What was the outcome of each exercise?<br><br>Which barrier solution cards were identified for prioritised practice 1<br><br>Which management solution cards were identified for prioritised practice 1<br><br>Which barrier solution cards were identified for prioritised practice 2<br><br>Which management solution cards were identified for prioritised practice 2<br><br>Which barrier solution cards were identified for prioritised practice 2<br><br>Which management solution cards were identified for prioritised practice 2	
How long did the meeting run for?	
Did anything go well/did anything not go well?	
GROUP FACILITATOR TO COMPLETE AFTER EACH MEETING CONDUCTED.	

For peer review only

Sustainability assessment

1. PARTICIPATION

Criteria for assessment	*Are many people participating? Are they active? Is anyone excluded from participation?*
Score / Average score assigned	
Identified successes	
Identified concerns / areas for improvement	
Actions agreed	
Additional notes	

2. LEADERSHIP

Criteria for assessment	*Do groups have leaders, are they helping or hindering the group? Do they have the capacity to lead groups well?*
Score / Average score assigned	
Identified successes	
Identified concerns / areas for improvement	
Actions agreed	
Additional notes	

WOMEN AND CHILDREN FIRST (UK)

3. ORGANISATIONAL STRUCTURE

Criteria for assessment	*Are the organisational structures, including the committee and taskforces, set up in the groups? If so, what is their purpose and how are they functioning?*
Score / Average score assigned	
Identified successes	
Identified concerns / areas for improvement	
Actions agreed	
Additional notes	

4. NEEDS / PROBLEM ASSESSMENT

Criteria for assessment	*Are the problems identified in the groups important and relevant to their respective community?*
Score / Average score assigned	
Identified successes	
Identified concerns / areas for improvement	
Actions agreed	
Additional notes	

For peer review only - http://bmjopen.bmj.com/site/about/guidelines.xhtml

WOMEN CHILDREN FIRST (UK)

5. RESOURCE MOBILISATION

Criteria for assessment	*Are the groups able to mobilise resources to implement their solutions? Are they able to mobilise resources from within the groups and/or outside the groups?*
Score / Average score assigned	
Identified successes	
Identified concerns / areas for improvement	
Actions agreed	
Additional notes	

6. ABILITY TO ASK "WHY"?

Criteria for assessment	*Do groups try to address root causes of the problems they face? If so, how well? By root causes we are referring to meeting re: contributing factors – the broader causes of ill health such as poverty.*
Score / Average score assigned	
Identified successes	
Identified concerns / areas for improvement	
Actions agreed	
Additional notes	

For peer review only - http://bmjopen.bmj.com/site/about/guidelines.xhtml

7. LINKS TO OTHER ORGANISATIONS / PEOPLE

Criteria for assessment	*Are they linking to other stakeholders (people, groups, organisations) who can help them solve identified issues? If so, who are they linking with and why?*
Score / Average score assigned	
Identified successes	
Identified concerns / areas for improvement	
Actions agreed	
Additional notes	

8. RELATIONSHIP WITH OUTSIDE AGENT

Criteria for assessment	*Is the external agent – Ministry of Health counterparts – involved and supporting the groups, building their capacity?*
Score / Average score assigned	
Identified successes	
Identified concerns / areas for improvement	
Actions agreed	
Additional notes	

WOMEN AND CHILDREN FIRST (UK)

9. PROGRAMME MANAGEMENT

**Criteria for assessment**	***Do groups feel they own the groups and their work? Are they capable of continuing the activities once the project comes to an end? Is the NGO implementing partner building group capacity to solve health problems and strengthening them to be sustainable?***
**Score / Average score assigned**	
**Identified successes**	
**Identified concerns / areas for improvement**	
**Actions agreed**	
**Additional notes**	

view only

For peer review only - http://bmjopen.bmj.com/site/about/guidelines.xhtml

Direct observation of intervention delivery and CF performance Form:

Did the facilitator deliver the components of the sessions according to the manual?
(Please rate this on scale of 1-4 scale i.e. 1 = no elements of the programme delivered;
2 = some elements of the programme, others missed or inserted;
3 = majority elements of the programme delivered;
4 = all elements of the programme delivered as per handbook).

What are your thoughts about the overall session?

Did you think there are areas for improvement and what where they?

Would you have done anything differently while facilitating the sessions and how?

What advice do you have for the community facilitator?

For peer review only

For peer review only - http://bmjopen.bmj.com/site/about/guidelines.xhtml

Development performance of children – (ASQ-3)

Improving lives

NHS
Oxleas
NHS Foundation Trust

Parents of Shiv LOGAN
3 Abbey Terrace
Abbey Wood
London
SE2 9EY

380 D

Oxleas 0 – 19 Years Public Health Nursing Service
Central Administration Team
Tel: 020 8836 8621
Email: oxl-tr.cat@nhs.net

11 Nov 2019

Dear Parents of Shiv,

9 – 12 month review Appointment confirmation

This letter is to confirm your appointment to review your child's development.

At: Gallions Reach Health Centre Bentham Road Thamesmead London SE28 8BE

On: Tuesday 10 Dec 2019 10:30

Enclosed with this letter are the 'Ages and Stages' questionnaires that are used for this developmental review. Please complete these forms and try out the activities with your baby before the appointment.

It is important that you complete the questionnaires with your child before your appointment and bring the completed questionnaires and Red Book with you on the day.

If you require any help completing this questionnaire or you have any queries about your appointment please contact us on **020 8836 8621**

To find out about Healthy Start benefits search online for the Healthy Start website

Visit our website which was designed by Oxleas Health Care professionals, service users and families to provide you with information about your health and wellbeing and how to access our services.

www.Oxleas.nhs.uk/CYP

Follow us on Twitter @oxleasCYPGre

There is a drop in Health Advice Session open every day from Monday to Friday across Greenwich. For times and venues see Health Visitor Timetable in the link above.

Yours sincerely
Public Health Nursing Service 0 -19 Years

For peer review only - http://bmjopen.bmj.com/site/about/guidelines.xhtml

ASQ-3 **10 Month Questionnaire** *page 3 of 6*

GROSS MOTOR *(continued)*

		YES	SOMETIMES	NOT YET	
3. When you stand your baby next to furniture or the cot rail, does she hold on without leaning her chest against the furniture for support?	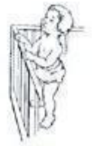	○	○	○	___
4. While holding onto furniture, does your baby bend down and pick up a toy from the floor and then return to a standing position?	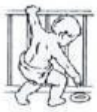	○	○	○	___
5. While holding onto furniture, does your baby lower himself with control (without falling or flopping down)?		○	○	○	___
6. Does your baby walk beside furniture while holding on with only one hand?		○	○	○	___
			GROSS MOTOR TOTAL		___

FINE MOTOR

		YES	SOMETIMES	NOT YET	
1. Does your baby pick up a small toy with only one hand?	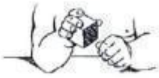	○	○	○	___
2. Does your baby *successfully* pick up a small cube of bread by using her thumb and all of her fingers in a raking motion? (*If she already picks up a small cube of bread, mark "yes" for this item.*)	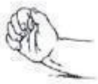	○	○	○	___
3. Does your baby pick up a small toy with the *tips* of his thumb and fingers? (*You should see a space between the toy and his palm.*)	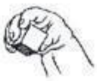	○	○	○	___
4. After one or two tries, does your baby pick up a piece of string with her first finger and thumb? (*The string may be attached to a toy.*)	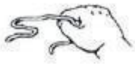	○	○	○	___
5. Does your baby pick up a small cube of bread with the tips of his thumb and a finger? (*He may rest his arm or hand on the table while doing it.*)	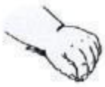	○	○	○	___ *
6. Does your baby put a small toy down, without dropping it, and then take her hand off the toy?		○	○	○	___
			FINE MOTOR TOTAL		___

* If Fine Motor Item 5 is marked "yes" or "sometimes," mark Fine Motor Item 2 "yes."

BE Av. 1 1.15

Ages & Stages Questionnaires®, Third Edition (ASQ-3™), Squires & Bricker
© 2009-2015 Paul H. Brookes Publishing Co. All rights reserved.
Adaptation into British English prepared with the Department of Health.

ASQ-3 **10 Month Questionnaire** *page 5 of 6*

OVERALL

Parents and providers may use the space below for additional comments.

1. Does your baby use both hands and both legs equally well? If no, explain: ○ YES ○ NO

2. When you help your baby stand, are his feet flat on the surface most of the time? If no, explain: ○ YES ○ NO

3. Do you have concerns that your baby is too quiet or does not make sounds like other babies? If yes, explain: ○ YES ○ NO

4. Does either parent have a family history of childhood deafness or hearing problems? If yes, explain: ○ YES ○ NO

5. Do you have concerns about your baby's eyesight? If yes, explain: ○ YES ○ NO

6. Has your baby had any medical or health-related problems in the last few months? If yes, explain: ○ YES ○ NO

BE Av. 1 1.15

Ages & Stages Questionnaires®, Third Edition (ASQ-3™), Squires & Bricker
© 2009-2015 Paul H. Brookes Publishing Co. All rights reserved.
Adaptation into British English prepared with the Department of Health.

For peer review only - http://bmjopen.bmj.com/site/about/guidelines.xhtml

4-day Food Diary

DAY 1

Day 1:		Date:		
Time	Where? With Whom? TV on? At table?	Food/Drink description & preparation	Brand Name	Portion size or quantity eaten
How to describe what you had and how much you had can be found on pages 16 - 21				
6am to 9am				
9am to 12 noon				

Time	Where? With Whom? TV on? At table?	Food/Drink description & preparation	Brand Name	Portion size or quantity eaten
12 noon to 2pm				
2pm to 5pm				

For peer review only - http://bmjopen.bmj.com/site/about/guidelines.xhtml

Time	Where? With Whom? TV on? At table?	Food/Drink description & preparation	Brand Name	Portion size or quantity eaten
		5pm to 8pm		
		8pm to 10pm		
		10pm to 6am		

Was the amount of **food** that you had today about what you usually have, less than usual, or more than usual?

Yes, usual ☐ No, **less** than usual ☐ No, **more** than usual ☐

Please tell us why you had less than usual

Please tell us why you had more than usual

Was the amount you had to **drink** today, including water, tea, coffee and soft drinks [and alcohol], about what you usually have, less than usual, or more than usual?

Yes, usual ☐ No, **less** than usual ☐ No, **more** than usual ☐

Please tell us why you had less than usual

Please tell us why you had more than usual

For peer review only - http://bmjopen.bmj.com/site/about/guidelines.xhtml

Did you **finish all the food and drink** that you recorded in the diary today?

Yes ☐ No ☐

If no, please **go back to the diary and make a note of any leftovers**

Did you take any **vitamins, minerals or other food supplements** today?

Yes ☐ No ☐

If yes, **please describe the supplements you took below**

Brand	Name (in full) including strength	Number of pills, capsules, teaspoons

Please record on the next pages details of any recipes or (if not already described) ingredients of made up dishes or take-away dishes.

**Write in recipes or ingredients of made up dishes or take-away dishes**			
NAME OF DISH:		***Serves:***	
Ingredients	***Amount***	***Ingredients***	***Amount***
Brief description of cooking method			

Write in recipes or ingredients of made up dishes or take-away dishes			
NAME OF DISH:		***Serves:***	
Ingredients	***Amount***	***Ingredients***	***Amount***
Brief description of cooking method			

DAY 2

Day 2:		Date:		
Time	Where? With Whom? TV on? At table?	Food/Drink description & preparation	Brand Name	Portion size or quantity eaten
How to describe what you had and how much you had can be found on pages 16 - 21				
6am to 9am				
9am to 12 noon				

For peer review only - http://bmjopen.bmj.com/site/about/guidelines.xhtml

Time	Where? With Whom? TV on? At table?	Food/Drink description & preparation	Brand Name	Portion size or quantity eaten
		12 noon to 2pm		
		2pm to 5pm		

Time	Where? With Whom? TV on? At table?	Food/Drink description & preparation	Brand Name	Portion size or quantity eaten
		5pm to 8pm		
		8pm to 10pm		
		10pm to 6am		

For peer review only - http://bmjopen.bmj.com/site/about/guidelines.xhtml

Was the amount of **food** that you had today about what you usually have, less than usual, or more than usual?

Yes, usual ☐ No, **less** than usual ☐ No, **more** than usual ☐

Please tell us why you had less than usual

Please tell us why you had more than usual

Was the amount you had to **drink** today, including water, tea, coffee and soft drinks [and alcohol], about what you usually have, less than usual, or more than usual?

Yes, usual ☐ No, **less** than usual ☐ No, **more** than usual ☐

Please tell us why you had less than usual

Please tell us why you had more than usual

Did you **finish all the food and drink** that you recorded in the diary today?

Yes ☐ No ☐

If no, please **go back to the diary and make a note of any leftovers**

Did you take any **vitamins, minerals or other food supplements** today?

Yes ☐ No ☐

If yes, **please describe the supplements you took below**

Brand	Name (in full) including strength	Number of pills, capsules, teaspoons

Please record on the next pages details of any recipes or (if not already described) ingredients of made up dishes or take-away dishes.

Write in recipes or ingredients of made up dishes or take-away dishes			
NAME OF DISH:		***Serves:***	
Ingredients	***Amount***	***Ingredients***	***Amount***
Brief description of cooking method			

Write in recipes or ingredients of made up dishes or take-away dishes			
NAME OF DISH:		***Serves:***	
Ingredients	***Amount***	***Ingredients***	***Amount***
Brief description of cooking method			

DAY 3

Day 3:		Date:		
Time	Where?<br>With Whom?<br>TV on?<br>At table?	Food/Drink description & preparation	Brand Name	Portion size or quantity <u>eaten</u>
How to describe what you had and how much you had can be found on pages 16 - 21				
6am to 9am				
9am to 12 noon				

Time	Where?<br>With Whom?<br>TV on?<br>At table?	Food/Drink description & preparation	Brand Name	Portion size or quantity <u>eaten</u>
12 noon to 2pm				
2pm to 5pm				

For peer review only - http://bmjopen.bmj.com/site/about/guidelines.xhtml

Time	Where? With Whom? TV on? At table?	Food/Drink description & preparation	Brand Name	Portion size or quantity eaten
		5pm to 8pm		
		8pm to 10pm		
		10pm to 6am		

Was the amount of **food** that you had today about what you usually have, less than usual, or more than usual?

Yes, usual ☐ No, **less** than usual ☐ No, **more** than usual ☐

Please tell us why you had less than usual

Please tell us why you had more than usual

Was the amount you had to **drink** today, including water, tea, coffee and soft drinks [and alcohol], about what you usually have, less than usual, or more than usual?

Yes, usual ☐ No, **less** than usual ☐ No, **more** than usual ☐

Please tell us why you had less than usual

Please tell us why you had more than usual

Did you **finish all the food and drink** that you recorded in the diary today?

Yes ☐ No ☐

If no, please **go back to the diary and make a note of any leftovers**

Did you take any **vitamins, minerals or other food supplements** today?

Yes ☐ No ☐

If yes, **please describe the supplements you took below**

Brand	Name (in full) including strength	Number of pills, capsules, teaspoons

Please record on the next pages details of any recipes or (if not already described) ingredients of made up dishes or take-away dishes.

Write in recipes or ingredients of made up dishes or take-away dishes			
NAME OF DISH:		***Serves:***	
Ingredients	***Amount***	***Ingredients***	***Amount***
Brief description of cooking method			

For peer review only - http://bmjopen.bmj.com/site/about/guidelines.xhtml

Write in recipes or ingredients of made up dishes or take-away dishes			
NAME OF DISH:		***Serves:***	
Ingredients	***Amount***	***Ingredients***	***Amount***
Brief description of cooking method			

DAY 4

Please remember to complete the general questions on pages 61-66!

Day 4:		Date:		
Time	Where?<br>With Whom?<br>TV on?<br>At table?	Food/Drink description & preparation	Brand Name	Portion size or quantity eaten
How to describe what you had and how much you had can be found on pages 16 - 21				
6am to 9am				
9am to 12 noon				

Time	Where?<br>With Whom?<br>TV on?<br>At table?	Food/Drink description & preparation	Brand Name	Portion size or quantity eaten
		12 noon to 2pm		
		2pm to 5pm		

Time	Where?<br>With Whom?<br>TV on?<br>At table?	Food/Drink description & preparation	Brand Name	Portion size or quantity eaten
		5pm to 8pm		
		8pm to 10pm		
		10pm to 6am		

For peer review only - http://bmjopen.bmj.com/site/about/guidelines.xhtml

Was the amount of **food** that you had today about what you usually have, less than usual, or more than usual?

Yes, usual ☐ No, **less** than usual ☐ No, **more** than usual ☐

Please tell us why you had less than usual

Please tell us why you had more than usual

Was the amount you had to **drink** today, including water, tea, coffee and soft drinks [and alcohol], about what you usually have, less than usual, or more than usual?

Yes, usual ☐ No, **less** than usual ☐ No, **more** than usual ☐

Please tell us why you had less than usual

Please tell us why you had more than usual

Did you **finish all the food and drink** that you recorded in the diary today?

Yes ☐ No ☐

If no, please **go back to the diary and make a note of any leftovers**

Did you take any **vitamins, minerals or other food supplements** today?

Yes ☐ No ☐

If yes, **please describe the supplements you took below**

Brand	Name (in full) including strength	Number of pills, capsules, teaspoons

Please record on the next pages details of any recipes or (if not already described) ingredients of made up dishes or take-away dishes.

For peer review only - http://bmjopen.bmj.com/site/about/guidelines.xhtml

Write in recipes or ingredients of made up dishes or take-away dishes			
NAME OF DISH:		***Serves:***	
Ingredients	***Amount***	***Ingredients***	***Amount***
Brief description of cooking method			

Write in recipes or ingredients of made up dishes or take-away dishes			
NAME OF DISH:		***Serves:***	
Ingredients	***Amount***	***Ingredients***	***Amount***
Brief description of cooking method			

For peer review only - http://bmjopen.bmj.com/site/about/guidelines.xhtml

General questions about your food/ drink during the recording period.

Special diet

1. Did you follow a special diet during the recording period e.g. vegetarian, cholesterol lowering, weight reducing?

Yes ☐ *Please specify* No ☐

Milk

2. Which type of milk did you use most often during the recording period?

Whole, fresh, pasteurised ☐ Semi-skimmed fresh, pasteurised ☐ Skimmed (fat free) fresh, pasteurised ☐ 1% fat milk, pasteurised ☐

Dried ☐ *Type* Soya ☐ *Type*

Other ☐ *Type* Did not use ☐

Tea and coffee

3. How much milk did you usually have in coffee/ tea?

Coffee	A lot ☐	Some ☐	A little ☐	None/did not drink ☐
Tea	A lot ☐	Some ☐	A little ☐	None/did not drink ☐

4. Did you usually sweeten your coffee/ tea with sugar?

Coffee	Yes ☐	How many teaspoons in a mug/cup? ☐	No/did not drink ☐
Tea	Yes ☐	How many teaspoons in a mug/cup? ☐	No/did not drink ☐

5. Did you usually sweeten your coffee/ tea with artificial sweetener?

Coffee	Yes ☐	How many tablets or teaspoons in a mug/cup? ☐	No/did not drink ☐
Tea	Yes ☐	How many tablets or teaspoons in a mug/cup? ☐	No/did not drink ☐

6. Did you drink decaffeinated coffee/ tea during the recording period?

Coffee	Always ☐	Sometimes ☐	Never ☐
Tea	Always ☐	Sometimes ☐	Never ☐

For peer review only - http://bmjopen.bmj.com/site/about/guidelines.xhtml

Breakfast cereals

7. How much milk did you usually have on breakfast cereal?

Drowned ☐ Average ☐ Damp ☐ None/did not eat ☐

8. How did you usually make your porridge?

With all water ☐ With all milk ☐ With milk and water ☐ Did not eat ☐

9. Did you usually sweeten or salt your porridge?

With sugar ☐ With honey ☐ With salt ☐ Neither/did not eat ☐

10. How did you usually make your instant oat cereal?

With all water ☐ With all milk ☐ With milk and water ☐ Did not eat ☐

11. Did you usually sweeten or salt your instant oat cereal?

With sugar ☐ With honey ☐ With salt ☐ Neither/did not eat ☐

Fats for spreading and cooking

12. Which type of butter, margarine or other fat spread did you use most often during the recording period? Please record the full product name and fat content

Name:

None ☐

e.g. Flora Omega 3 plus, low fat spread, 38% fat, polyunsaturated

13. How thickly did you spread butter, margarine on bread, crackers etc?

Thick ☐ Medium ☐ Thin ☐ N/A ☐

14. Which type of cooking fat/oil did your household use most often over the recording period? Please record the full product name e.g. *Sainsbury's sunflower oil*

Name:

None ☐

Bread

15. Which type of bread did you eat most often during the recording period?

White ☐ Granary ☐ Wholemeal ☐ Brown ☐

50/50 bread e.g. Hovis Best of Both ☐ Other ☐ *Type* Did not eat ☐

16. Was it a large loaf or a small loaf?

Large ☐ Small ☐

17. If the bread was shop bought, how was it sliced?

Thick ☐ Medium ☐ Thin ☐ Unsliced ☐ N/A ☐

Meat

18. If you ate meat during the recording period, did you eat the visible fat?

Always ☐ Sometimes ☐ Never ☐ Did not eat meat ☐

19. If you ate poultry (e.g. chicken, turkey) during the recording period, did you eat the skin?

Always ☐ Sometimes ☐ Never ☐ Did not eat poultry ☐

Fruit and vegetables

20. If you ate apples during the recording period, did you eat the skin?

Always ☐ Sometimes ☐ Never ☐ Did not eat ☐

21. If you ate pears during the recording period, did you eat the skin?

Always ☐ Sometimes ☐ Never ☐ Did not eat ☐

22. If you ate new potatoes during the recording period, did you eat the skin?

Always ☐ Sometimes ☐ Never ☐ Did not eat ☐

23. If you ate baked/jacket potatoes during the recording period, did you eat the skin?

Always ☐ Sometimes ☐ Never ☐ Did not eat ☐

Salt

24. Do you add salt to your food at the table?

Always ☐ Sometimes ☐ Never ☐

25. Do you add salt substitute to your food at the table? *e.g. LoSalt*

Always ☐ Sometimes ☐ Never ☐

Water

26. Which type of water did you drink most often during the recording period?

Tap ☐ Filtered ☐ Bottled ☐ *brand* ________ Did not drink ☐

Thank you for completing this diary.

For peer review only - http://bmjopen.bmj.com/site/about/guidelines.xhtml

Children Feeding Behaviour

ID:

Child Eating Behaviour Questionnaire (CEBQ)

Please read the following statements and tick the boxes most appropriate to your child's eating behaviour.

	Never	Rarely	Some-times	Often	Always
My child loves food	☐	☐	☐	☐	☐
My child eats more when worried	☐	☐	☐	☐	☐
My child has a big appetite	☐	☐	☐	☐	☐
My child finishes his/her meal quickly	☐	☐	☐	☐	☐
My child is interested in food	☐	☐	☐	☐	☐
My child is always asking for a drink	☐	☐	☐	☐	☐
My child refuses new foods at first	☐	☐	☐	☐	☐
My child eats slowly	☐	☐	☐	☐	☐
My child eats less when angry	☐	☐	☐	☐	☐
My child enjoys tasting new foods	☐	☐	☐	☐	☐
My child eats less when s/he is tired	☐	☐	☐	☐	☐
My child is always asking for food	☐	☐	☐	☐	☐
My child eats more when annoyed	☐	☐	☐	☐	☐
If allowed to, my child would eat too much	☐	☐	☐	☐	☐
My child eats more when anxious	☐	☐	☐	☐	☐
My child enjoys a wide variety of foods	☐	☐	☐	☐	☐
My child leaves food on his/her plate at the end of a meal	☐	☐	☐	☐	☐
My child takes more than 30 minutes to finish a meal	☐	☐	☐	☐	☐

	Never	Rarely	Some-times	Often	Always
Given the choice, my child would eat most of the time	☐	☐	☐	☐	☐
My child looks forward to mealtimes	☐	☐	☐	☐	☐
My child gets full before his/her meal is finished	☐	☐	☐	☐	☐
My child enjoys eating	☐	☐	☐	☐	☐
My child eats more when she is happy	☐	☐	☐	☐	☐
My child is difficult to please with meals	☐	☐	☐	☐	☐
My child eats less when upset	☐	☐	☐	☐	☐
My child gets full up easily	☐	☐	☐	☐	☐
My child eats more when s/he has nothing else to do	☐	☐	☐	☐	☐
Even if my child is full up s/he finds room to eat his/her favourite food	☐	☐	☐	☐	☐
If given the chance, my child would drink continuously throughout the day	☐	☐	☐	☐	☐
My child cannot eat a meal if s/he has had a snack just before	☐	☐	☐	☐	☐
If given the chance, my child would always be having a drink	☐	☐	☐	☐	☐
My child is interested in tasting food s/he hasn't tasted before	☐	☐	☐	☐	☐
My child decides that s/he doesn't like a food, even without tasting it	☐	☐	☐	☐	☐
If given the chance, my child would always have food in his/her mouth	☐	☐	☐	☐	☐
My child eats more and more slowly during the course of a meal	☐	☐	☐	☐	☐

For peer review only - http://bmjopen.bmj.com/site/about/guidelines.xhtml

Parental Feeding Style

Parental Feeding Style Questionnaire

Please read the following statements and tick the appropriate boxes to show how you deal with feeding your child. It is important to remember that there are no right or wrong answers to these questions, we are interested in what parents really feel and do.

ID ☐

	Never	Rarely	Some-times	Often	Always
I allow my child to choose which foods to have for meals	☐	☐	☐	☐	☐
I give my child something to eat to make him/her feel better when s/he is feeling upset	☐	☐	☐	☐	☐
I encourage my child to look forward to the meal	☐	☐	☐	☐	☐
I praise my child if s/he eats what I give him/her	☐	☐	☐	☐	☐
I decide how many snacks my child should have	☐	☐	☐	☐	☐
I encourage my child to eat a wide variety of foods	☐	☐	☐	☐	☐
In order to get my child to behave him/herself I promise him/her something to eat	☐	☐	☐	☐	☐
I present food in an attractive way to my child	☐	☐	☐	☐	☐
If my child misbehaves I withhold his/her favourite food	☐	☐	☐	☐	☐
I encourage my child to taste each of the foods I serve at mealtimes	☐	☐	☐	☐	☐
I allow my child to wander around during a meal	☐	☐	☐	☐	☐
I encourage my child to try foods that s/he hasn't tasted before	☐	☐	☐	☐	☐
I give my child something to eat to make him/her feel better when s/he has been hurt	☐	☐	☐	☐	☐
I let my child decide when s/he would like to have her meal	☐	☐	☐	☐	☐
I give my child something to eat if s/he is feeling bored	☐	☐	☐	☐	☐
I allow my child to decide when s/he has had enough snacks to eat	☐	☐	☐	☐	☐
I decide when it is time for my child to have a snack	☐	☐	☐	☐	☐
I use puddings as a bribe to get my child to eat his/her main course	☐	☐	☐	☐	☐
I encourage my child to enjoy his/her food	☐	☐	☐	☐	☐

PTO

For peer review only - http://bmjopen.bmj.com/site/about/guidelines.xhtml

Network Diffusion Form

(To be completed by participants at the end of each PLA session and at 6 months from baseline)

1. How many people did you share your material/information from the PLA sessions with?...

2. How did you share this information/material (verbal, social media, whatsapp etc)?...

3. Please fill the table below with details of the people you shared the information with -

Serial number	Relation to you	Age/Gender	Family size	How did you share (verbal, social media, whatsapp)

For peer review only

For peer review only - http://bmjopen.bmj.com/site/about/guidelines.xhtml

Equality Impact Assessment

Equality Impact Assessment- NEON

Question	Response
**1. Name of intervention being assessed**	NEON Intervention (PLA Cycle)
**2. Summary of aims and objectives of the intervention**	To improve infant feeding, care and dental hygiene practices amongst the South Asian Communities of Tower Hamlets, Newham, and Waltham Forest
**3. What involvement and consultation has been done in relation to this intervention?** *(e.g., with relevant groups and stakeholders)*	
**4. Who is affected by the intervention?**	Infants (aged<24 months)<br>Mothers<br>Pregnant women<br>Carers- Grandmother etc.
**5. What are the arrangements for monitoring and reviewing the actual impact of the intervention?**	

Protected Characteristic Group	Is there a potential for positive or negative impact?	Please explain and give examples of any evidence/data used	Action to address negative impact (e.g. adjustment to the intervention)
Disability			
Gender reassignment			
Marriage or civil partnership			
Pregnancy and maternity			
Race			
Religion or belief			
Sexual orientation			
Sex (gender)			
Age			

For peer review only - http://bmjopen.bmj.com/site/about/guidelines.xhtml

Evaluation:

Question	Explanation / justification
Is it possible the proposed intervention could discriminate or unfairly disadvantage people?	

Final Decision:	Tick the relevant box	Include any explanation / justification required
1. No barriers identified, therefore activity will **proceed**.		
2. You can decide to **stop** the intervention at some point because the data shows bias towards one or more groups		
3. You can **adapt or change** the intervention in a way which you think will eliminate the bias		
4. Barriers and impact identified, however having considered all available options carefully, there appear to be no other proportionate ways to achieve the aim of the intervention (e.g., in extreme cases or where positive action is taken). Therefore, you are going to **proceed with caution** with this intervention knowing that it may favour some people less than others, providing justification for this decision.		

**Date completed:**	
**Completed by whom (Borough/individual):**	

Staff time questionnaire

Measurement of Time Use for staff involved in *NEON*

This questionnaire is for collecting information on how staff from the study team who are contributing to the Nurture Early for Optimal Nutrition (*NEON*) divide their time between the different trial components. We aim to collect this information from all our partners staff involved in *NEON* over the duration of the 3-year of the study.

The information you provide will only be used for research purposes only. It ***will not*** be used to monitor performance or to plan future activities. The information you provide will help us to divide the total *NEON* cost into the following components: intervention, M&E, process evaluation, and research. This will allow us to report the cost effectiveness of the *NEON* intervention. We also want to separate out the setting up cost from the implementation cost, to inform others who may wish to implement these interventions.

Your name will not be used in the analysis but you will be identifiable through your answers. If you do not want to proceed, or wish to stop at any time, you can do so. If you have any questions, please discuss with Shereen Al Laham (*s.laham@ucl.ac.uk*).

==

Name of Staff Member: ....

Job title / role: ....

Institution: ....

Date: ....

---

1. When did your involvement with *NEON* first begin? .... (Month/Year)
2. Since you started, have you been involved in any project other than *NEON*?
   - ☐ Yes
   - ☐ No. **Go to question 6.**
3. If Yes, how many days did you spend on *NEON* in the last 30 days?
   .... Days OR .... Percent of time
4. Was this a typical month for you, since you started work on *NEON*?
   - ☐ Yes. **Go to question 6.**
   - ☐ No
5. If No, please tell me how you have divided your time between *NEON* and any other project since you first started on *NEON*.
   ....

....

(E.g: On average, 2 days per week from January to May, then full-time since June. Answer can be in percentage, days, or weeks – whatever you think most accurately represents your actual time use.)

6. Thinking now about the **last 30 days**, and only those days you worked on *NEON*, how have you in practice divided your time between the following *NEON* activities?

Page **1** of **2**

For peer review only - http://bmjopen.bmj.com/site/about/guidelines.xhtml

a) I spent .... days OR .... percent time on Monitoring and Evaluation.
b) I spent .... days OR .... percent time on study delivery and implementation.
c) I spent .... days OR .... percent time on Research Work, that is, working on the study design, reading the literature, or writing up or presenting the research findings.
d) I spent .... days OR .... percent time on Joint Work, that is, it is not possible to say it was one of the above. For example, general management or support functions.

7. Is this how you usually divide your time?

☐ Yes. **Go to question 9.**

☐ No

8. If No, please tell me how you have divided your time between the different *NEON* activities since you first started on *NEON*. *(Use the categories in question 6.)*

....

....

....

(E.g: 50% Joint work for the first 6 months, then 1 day per week Joint work but otherwise only NEON. Answer can be in percentage, days, or weeks – whatever you think most accurately represents your actual time use.)

9. Finally, I want to ask you about the division of time between **setting up** and **implementation** of the *NEON* study. Examples of setting up activities are: recruiting and training; developing and testing the intervention material. These activities may still be ongoing.
Please could you tell us when you start to work on implementation, and do setting up activities still continue

	Activity	Start date of implementation<br>*(Month/Year)*	Time spend on SETTING UP activities since start date<br>*(days per week or percentage of time)*
a	NEON intervention implementation		
b	Monitoring and Evaluation implementation		
c	Process Evaluation implementation		

== Thank You ==

SPIRIT
STANDARD PROTOCOL ITEMS: RECOMMENDATIONS FOR INTERVENTIONAL TRIALS

SPIRIT 2013 Checklist: Recommended items to address in a clinical trial protocol and related documents*

Section/ item	Item No	Description	Page
**Administrative information**			
Title	1	Descriptive title identifying the study design, population, interventions, and, if applicable, trial acronym	1
Trial registration	2a	Trial identifier and registry name. If not yet registered, name of intended registry	3, 17, 18
	2b	All items from the World Health Organization Trial Registration Data Set	N/A
Protocol version	3	Date and version identifier	17, 18
Funding	4	Sources and types of financial, material, and other support	17
Roles and responsibilities	5a	Names, affiliations, and roles of protocol contributors	1, 17
	5b	Name and contact information for the trial sponsor	17
	5c	Role of study sponsor and funders, if any, in study design; collection, management, analysis, and interpretation of data; writing of the report; and the decision to submit the report for publication, including whether they will have ultimate authority over any of these activities	17, 18
	5d	Composition, roles, and responsibilities of the coordinating centre, steering committee, endpoint adjudication committee, data management team, and other individuals or groups overseeing the trial, if applicable (see Item 21a for data monitoring committee)	11, 16
**Introduction**			
Background and rationale	6a	Description of research question and justification for undertaking the trial, including summary of relevant studies (published and unpublished) examining benefits and harms for each intervention	3, 5
	6b	Explanation for choice of comparators	6, 7
Objectives	7	Specific objectives or hypotheses	6
Trial design	8	Description of trial design including type of trial (eg, parallel group, crossover, factorial, single group), allocation ratio, and framework (eg, superiority, equivalence, noninferiority, exploratory)	6, 7

For peer review only

Methods: Participants, interventions, and outcomes

Study setting	9	Description of study settings (eg, community clinic, academic hospital) and list of countries where data will be collected. Reference to where list of study sites can be obtained	7
Eligibility criteria	10	Inclusion and exclusion criteria for participants. If applicable, eligibility criteria for study centres and individuals who will perform the interventions (eg, surgeons, psychotherapists)	7, 8
Interventions	11a	Interventions for each group with sufficient detail to allow replication, including how and when they will be administered	6, 7, 10
	11b	Criteria for discontinuing or modifying allocated interventions for a given trial participant (eg, drug dose change in response to harms, participant request, or improving/worsening disease)	N/A
	11c	Strategies to improve adherence to intervention protocols, and any procedures for monitoring adherence (eg, drug tablet return, laboratory tests)	N/A
	11d	Relevant concomitant care and interventions that are permitted or prohibited during the trial	N/A
Outcomes	12	Primary, secondary, and other outcomes, including the specific measurement variable (eg, systolic blood pressure), analysis metric (eg, change from baseline, final value, time to event), method of aggregation (eg, median, proportion), and time point for each outcome. Explanation of the clinical relevance of chosen efficacy and harm outcomes is strongly recommended	11, 12
Participant timeline	13	Time schedule of enrolment, interventions (including any run-ins and washouts), assessments, and visits for participants. A schematic diagram is highly recommended (see Figure)	9
Sample size	14	Estimated number of participants needed to achieve study objectives and how it was determined, including clinical and statistical assumptions supporting any sample size calculations	8
Recruitment	15	Strategies for achieving adequate participant enrolment to reach target sample size	8, 9

Methods: Assignment of interventions (for controlled trials)

Allocation:

Sequence generation	16a	Method of generating the allocation sequence (eg, computer-generated random numbers), and list of any factors for stratification. To reduce	9, 10

For peer review only - http://bmjopen.bmj.com/site/about/guidelines.xhtml

		predictability of a random sequence, details of any planned restriction (eg, blocking) should be provided in a separate document that is unavailable to those who enrol participants or assign interventions	
Allocation concealment mechanism	16b	Mechanism of implementing the allocation sequence (eg, central telephone; sequentially numbered, opaque, sealed envelopes), describing any steps to conceal the sequence until interventions are assigned	9, 10
Implementation	16c	Who will generate the allocation sequence, who will enrol participants, and who will assign participants to interventions	9, 10
Blinding (masking)	17a	Who will be blinded after assignment to interventions (eg, trial participants, care providers, outcome assessors, data analysts), and how	9, 10
	17b	If blinded, circumstances under which unblinding is permissible, and procedure for revealing a participant's allocated intervention during the trial	9, 10
**Methods: Data collection, management, and analysis**			
Data collection methods	18a	Plans for assessment and collection of outcome, baseline, and other trial data, including any related processes to promote data quality (eg, duplicate measurements, training of assessors) and a description of study instruments (eg, questionnaires, laboratory tests) along with their reliability and validity, if known. Reference to where data collection forms can be found, if not in the protocol	11, 12, 13
	18b	Plans to promote participant retention and complete follow-up, including list of any outcome data to be collected for participants who discontinue or deviate from intervention protocols	14, 16
Data management	19	Plans for data entry, coding, security, and storage, including any related processes to promote data quality (eg, double data entry; range checks for data values). Reference to where details of data management procedures can be found, if not in the protocol	14
Statistical methods	20a	Statistical methods for analysing primary and secondary outcomes. Reference to where other details of the statistical analysis plan can be found, if not in the protocol	14, 15
	20b	Methods for any additional analyses (eg, subgroup and adjusted analyses)	N/A
	20c	Definition of analysis population relating to protocol non-adherence (eg, as randomised analysis), and any statistical methods to handle missing data (eg, multiple imputation)	N/A
**Methods: Monitoring**			
Data monitoring	21a	Composition of data monitoring committee (DMC); summary of its role and reporting structure; statement of whether it is independent from the sponsor and competing interests; and reference to where further details	16

		about its charter can be found, if not in the protocol. Alternatively, an explanation of why a DMC is not needed	
	21b	Description of any interim analyses and stopping guidelines, including who will have access to these interim results and make the final decision to terminate the trial	16
/Harms	22	Plans for collecting, assessing, reporting, and managing solicited and spontaneously reported adverse events and other unintended effects of trial interventions or trial conduct	N/A
Auditing	23	Frequency and procedures for auditing trial conduct, if any, and whether the process will be independent from investigators and the sponsor	N/A
**Ethics and dissemination**			
Research ethics approval	24	Plans for seeking research ethics committee/institutional review board (REC/IRB) approval	2, 16, 18
Protocol amendments	25	Plans for communicating important protocol modifications (eg, changes to eligibility criteria, outcomes, analyses) to relevant parties (eg, investigators, REC/IRBs, trial participants, trial registries, journals, regulators)	16, 17
Consent or assent	26a	Who will obtain informed consent or assent from potential trial participants or authorised surrogates, and how (see Item 32)	18
	26b	Additional consent provisions for collection and use of participant data and biological specimens in ancillary studies, if applicable	N/A
Confidentiality	27	How personal information about potential and enrolled participants will be collected, shared, and maintained in order to protect confidentiality before, during, and after the trial	17, 18
Declaration of interests	28	Financial and other competing interests for principal investigators for the overall trial and each study site	17, 18
Access to data	29	Statement of who will have access to the final trial dataset, and disclosure of contractual agreements that limit such access for investigators	18
Ancillary and post-trial care	30	Provisions, if any, for ancillary and post-trial care, and for compensation to those who suffer harm from trial participation	N/A
Dissemination policy	31a	Plans for investigators and sponsor to communicate trial results to participants, healthcare professionals, the public, and other relevant groups (eg, via publication, reporting in results databases, or other data sharing arrangements), including any publication restrictions	18
	31b	Authorship eligibility guidelines and any intended use of professional writers	N/A

For peer review only - http://bmjopen.bmj.com/site/about/guidelines.xhtml

	31c	Plans, if any, for granting public access to the full protocol, participant-level dataset, and statistical code	18
**Appendices**			
Informed consent materials	32	Model consent form and other related documentation given to participants and authorised surrogates	Suppl. material
Biological specimens	33	Plans for collection, laboratory evaluation, and storage of biological specimens for genetic or molecular analysis in the current trial and for future use in ancillary studies, if applicable	N/A

*It is strongly recommended that this checklist be read in conjunction with the SPIRIT 2013 Explanation & Elaboration for important clarification on the items. Amendments to the protocol should be tracked and dated. The SPIRIT checklist is copyrighted by the SPIRIT Group under the Creative Commons "Attribution-NonCommercial-NoDerivs 3.0 Unported" license.

For peer review only

For peer review only - http://bmjopen.bmj.com/site/about/guidelines.xhtml
